# Analysis of the long non-coding RNA LINC01614 in non-small cell lung cancer

**DOI:** 10.1097/MD.0000000000016437

**Published:** 2019-07-26

**Authors:** Yan Sun, Chunhua Ling

**Affiliations:** Department of Respiratory Diseases, The First Affiliated Hospital of Soochow University, Soochow, China.

**Keywords:** expression profile, LINC01614, long non-coding RNA, non-small cell lung cancer

## Abstract

The aim of this study was toexplore the long non-coding RNA (lncRNA) expression pattern of non-small cell lung cancer (NSCLC) on a genome-wide scale and investigate their potential biological function in NSCLC.

LncRNAs were investigated in 6 pairs of NSCLC and matched adjacent non-tumor lung tissues (NTL) by microarray. A validation cohort was obtained from The Cancer Genome Atlas (TCGA) database and the effect of LINC01614 on diagnosis and prognosis in NSCLC was analyzed. Gene set enrichment analysis (GSEA) was used to predict the potential molecular mechanism of LINC01614, one identified lncRNA.

A total of 1392 differentially expressed lncRNAs were identified. LINC01614 was the most aberrantly expressed lncRNA in NSCLC compared with NTL. We confirmed the significantly upregulated LINC01614 in NSCLC patients from TCGA database. Furthermore, in TCGA database, LINC01614 was significantly upregulated in both adenocarcinoma and squamous cell carcinoma. And high expression of LINC01614 indicated poor overall survival of NSCLC patients. A sensitivity of 93% was calculated conditional on a high specificity of 95% for the discrimination of NSCLC tissues from normal tissues. Furthermore, the expression levels of LINC01614 were associated with the stage of tumor, but had no relationship with age and sex. Additionally, GSEA found that LINC01614 might be involved in TGF-β-, P53-, IGF-IR-mediated, Wnt and RTK/Ras/MAPK signaling pathways.

lncRNAs may play key roles in the development of NSCLC. LINC01614 is the most aberrantly expressed lncRNA in NSCLC tissues in our experiment and is also significantly differentially expressed in NSCLC patients from TCGA database. LINC01614 could be a prognostic indicator and has the potential to be a diagnostic biomarker of NSCLC.

## Abstract 2

Supplemental Digital Content is available in the text

## 1 Introduction

Lung cancer is the leading cause of cancer death worldwide.^[1]^ Non-small cell lung cancer (NSCLC) which mainly consists of adenocarcinoma and squamous cell carcinoma is a predominant form of lung cancer, accounting for approximately 80% of all lung cancers. Despite recent advances in the treatment of NSCLC, the prognosis of NSCLC is still unfavorable, with a 5-year overall survival (OS) of 15.9%.^[2]^ Thus, we still need a deep study to have a better understanding of the mechanisms of occurrence, development, and progression of NSCLC to improve its prognosis.

Long non-coding RNAs (lncRNAs) are RNA molecules of ≥200 nucleotides in length.^[3]^ They have limited or no protein-coding capacity and mainly locate within nucleus or cytosolic compartment.^[3]^ LncRNAs are categorized into 5 categories according to their relationship with protein-coding genes^[4]^: sense, antisense, divergent, intronic, and intergenic. They can act as decoys, scaffolds, signals, sponges, and guides and participate in a wide range of cellular processes, including dosage compensation, imprinting, transcription, mRNA splicing, translation, nuclear and cytoplasmic trafficking, and cellular localization.^[5]^ Thus, they are involved in epigenetic regulation, transcriptional regulation, and post-transcriptional processing.^[5]^

Growing evidence indicates that lncRNAs play critical roles in tumor initiation, progression, and metastasis by modulating oncogenic and tumor-suppressing pathways.^[6]^ A number of specific lncRNAs have been found to be differentially expressed in a variety of cancers.^[7]^ As specific lncRNAs are involved in tumorigenesis, they are becoming attractive treatment targets.^[8–10]^ Previous studies showed that lncRNAs were also involved in the pathogenesis of NSCLC and these studies provide new insights into the biology of NSCLC.^[3,8,11]^

In this study, we characterized the lncRNA expression profile in NSCLC by microarray analysis and identified the most aberrantly expressed lncRNA, LINC01614. Then, we confirmed the significantly upregulated LINC01614 in NSCLC patients from The Cancer Genome Atlas (TCGA) database. Using bioinformatics analyses, we found that high expression of LINC01614 indicated poor OS and the expression levels of LINC01614 detected in NSCLC and normal tissues showed a good diagnostic potential. We might expect the diagnostic potential of circulating LINC01614 and suppose it could serve as a biomarker for the diagnosis of NSCLC. In addition, Gene set enrichment analysis (GSEA) found that LINC01614 might be associated with the TGF-β, P53, IGF-IR-mediated, Wnt and RTK/Ras/MAPK signaling pathways.

## 2 Materials and methods

### 2.1 Patients and tissue samples

The study was approved by the Ethics Committee of the First Affiliated Hospital of Soochow University. Written informed consent was obtained from each patient. A total of 6 pairs of primary NSCLC tissues and matched adjacent normal tissues were collected from patients who underwent surgery at the Department of Thoracic Surgery of the First Affiliated Hospital of Soochow University in November 2016. After removal, the samples were collected immediately into EP tubes filled with RNAlater (Qiagen, Venlo, The Netherlands), stored at 4°C overnight so that RNAlater could fully penetrate into the samples and then stored at −20°C until use. All tumor specimens and paired normal tissues were confirmed by experienced pathologists. The clinical and pathological characteristics of each patient were also collected (Table 1).

**Table 1:**
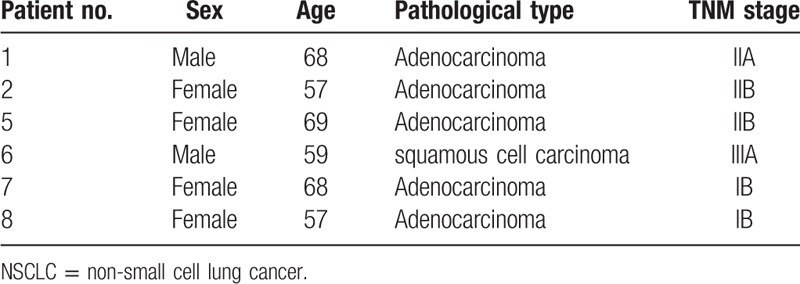
Clinical and pathological characteristics of 6 NSCLC patients.

### 2.2 NSCLC expression microarrays and data analysis

**Figure 1:**
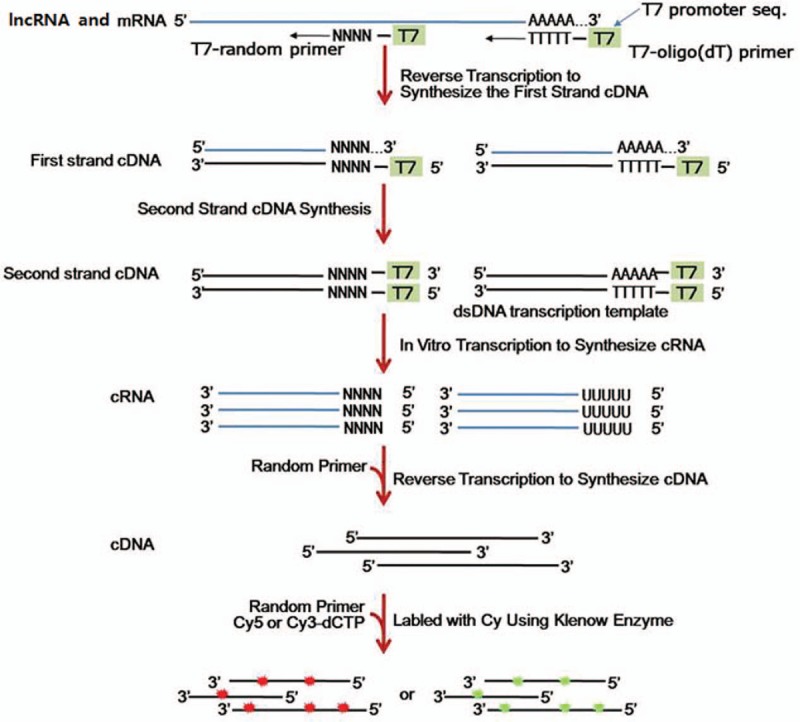
The procedure of total RNA transforming into single-strand DNA labeled with Cy3-dCTP or Cy5-dCTP. lncRNAs = long non-coding RNAs.

The experiment was performed in the laboratory of the CapitalBio Corp (Beijing, China). In brief, total RNA was first extracted by Trizol reagent (Invitrogen, Carlsbad, CA) and then purified by the NucleoSpin RNA Clean-up Kit (Macherey-Na-gel, Dϋren, Germany). The purity and concentration of extracted RNA were determined from OD260/280 readings on a NanoDrop ND-1000 spectrophotometer (Thermo Fisher Scientific, Waltham, MA) and the integrity was evaluated by 1% formaldehyde denaturing gel electrophoresis. 1ug total RNA was used to be transformed into single-strand DNA labeled with Cy3-dCTP or Cy5-dCTP (GE Healthcare, Piscataway, NJ) through various steps (The procedure was depicted in Fig. 1).The final products were hybridized to the CapitalBio Jingxin lncRNA&mRNA Human 4 × 180K Gene Expression Microarray V4.0 (CapitalBio Corp., Beijing, China), which contains 40,916 lncRNA detection probes and 34,235 mRNA detection probes. The data were extracted using the Agilent Feature Extraction and were summarized, normalized, and quality-controlled using the GeneSpring GX software (Agilent Technologies, Santa Clara, CA). To select the significantly differentially expressed lncRNAs and mRNAs between NSCLC and normal tissue samples, we used a threshold value of ≥2 or ≤-2 fold change and a Student *t* test *P*-value ≤.05. The data of the significantly differentially expressed lncRNAs were analyzed with hierarchical clustering using the Cluster 3.0 software (Human Genome Center, Tokyo, Japan) and the heatmap was performed using the Java Treeview software (Stanford University of Medicine, Stanford, CA). The volcano plots of significantly differentially expressed lncRNAs were performed by R × 64 3.2.4 Revised and the ggplot2 package. Based on the significantly differentially expressed mRNAs between NSCLC and normal tissue samples, the gene ontology (GO) and pathways enriched in the BioCyc, KEGG PATHWAY, Panther, and Reactome databases were analyzed using the KEGG Orthology Based Annotation System (KOBAS) software. The GO analysis was divided into 3 parts including molecular function, biological process, and cellular component. *P* values <.05 were considered statistically significant. In addition, the co-expression of lncRNA-mRNA pairs was determined by calculating the Pearson correlation coefficients (PCC) of each dysregulated lncRNA and mRNA probe. The PCC <−0.90 or >0.90 and *P* values <.05 were considered statistically significant.

### 2.3 The Quantitative real-time polymerase chain reaction validation experiments for the microarray analyses results

We selected the most dysregulated lncRNA LINC01614 and randomly selected 3 significantly differentially expressed lncRNAs from the microarray analyses results for the validation experiments. Total RNA of 6 pairs of primary NSCLC tissues and matched adjacent normal tissues was respectively extracted by Trizol reagent (Invitrogen, Carlsbad, CA) according to the manufacturer's instructions. Around 1 μg total RNA was reverse-transcribed in a final volume of 20 μL using M-MLV Reverse Transcriptase. Then, we used 2 μL of the complementary DNA (cDNA) for quantitative real-time polymerase chain reaction (qRT-PCR). And β-actin was used as a reference gene for normalization. The qRT-PCR was performed in a total reaction volume of 20 μL on a Roche real-time PCR kit. The qRT-PCR included an initial denaturation step of 10 minutes at 95°C and 39 cycles of 10 seconds at 95°C, 15 seconds at 60°C, and 20 seconds at 72°C. All the qRT-PCR experiments were performed in triplicate. The sequences of primers used for qRT-PCR experiments were as follows: 5′-ACC AAG TGA GAA ACT GAA GAC CAG-3′ (forward) and 5′-TCC TCA TGG AGG GCT AGG TTG-3′ (reverse) for FENDRR-005; 5′-GAG CTC CTT GGA GAA TCG GC-3′ (forward) and 5′-ACA CGA AAG GCT GGA AGT GTC-3′ (reverse) for LINC00968-007; 5′-TGC CGT TCT CCA GCG C-3′ (forward) and 5′-CCT CAG GTG TCC TCA TCT GGT AA-3′ (reverse) for DLEU1; 5′-TGT CAA CCA AGA GCG AAG CC-3′ (forward) and 5′-CTT GGA CAC AGA CCC TAG CAC-3′ (reverse) for LINC01614; 5′-ACA GGG GAG GTG ATA GCA TT-3′ (forward) and 5′-GAC CAA AAG CCT TCA TAC ATC TC-3′ (reverse) for β-actin. Relative expression values of the 4 lncRNAs were calculated by using 2^−ΔΔCt^. Next, we compared the expression levels of the 4 lncRNAs between NSCLC tissues and matched adjacent normal tissues and represented the results through the bar charts by using GraphPad Prism 7.01 (GraphPad Software Inc., San Diego, CA).

### 2.4 Gene expression profiles of NSCLC patients in the TCGA database and data analysis

The gene expression datasets of NSCLC patients including 594 adenocarcinoma patients providing 535 tumor tissues and 59 normal tissues and 551 squamous cell carcinoma patients providing 502 tumor tissues and 49 normal tissues were downloaded from the TCGA database (http://cancergenome.nih.gov/) by using the GDC Data Transfer Tool. Then, the datasets were processed using ActivePerl 5.24.1 and Perl package JSON2.90. We analyzed the differential expression of the genes from the processed datasets through using R × 64 3.2.4 Revised and edgeR package. We also used threshold values of ≥2 or ≤-2 fold change and *P* values ≤.05 to determine the significantly differentially expressed lncRNAs and mRNAs between NSCLC and normal tissues. We explored the correlation between low/high LINC01614 expression level (based on the median expression level of LINC01614) and NSCLC patients’ OS through using R × 64 3.2.4 Revised and its hash and survival packages. *P* values <.05 were considered statistically significant. Next, we randomly selected 100 adenocarcinoma patients’ tissues, 100 squamous cell carcinoma patients’ tissues, and 100 patients’ tumor adjacent normal tissues from the TCGA database and their clinical characteristics and LINC01614 expression level in the tissues were listed below (Table S1-3). Then we drew the receiver-operating characteristic (ROC) curve using SPSS 22.0 (IBM, Armonk, NY) to evaluate the diagnostic sensitivity and specificity of LINC01614. The area under the curve (AUC) was calculated from the ROC curve and the best cutoff point was determined using the Youden's index. Additionally, we extracted the clinical information (including age, sex, and the stage of tumor) from Table S1 and Table S2 to explore whether the expression levels of LINC01614 in NSCLC tissues were affected by clinical factors through using GraphPad Prism 7.01 (GraphPad Software Inc.,San Diego, CA. *P* values <.05 were considered statistically significant. Finally, we tried to investigate the potential molecular mechanisms of LINC01614 in NSCLC and gene set enrichment analysis (GSEA) was conducted, which is a widely used method for predicting biological function of unknown genes.^[12,13]^

## 3 Results

### 3.1 NSCLC expression microarrays and data analysis

**Figure 2:**
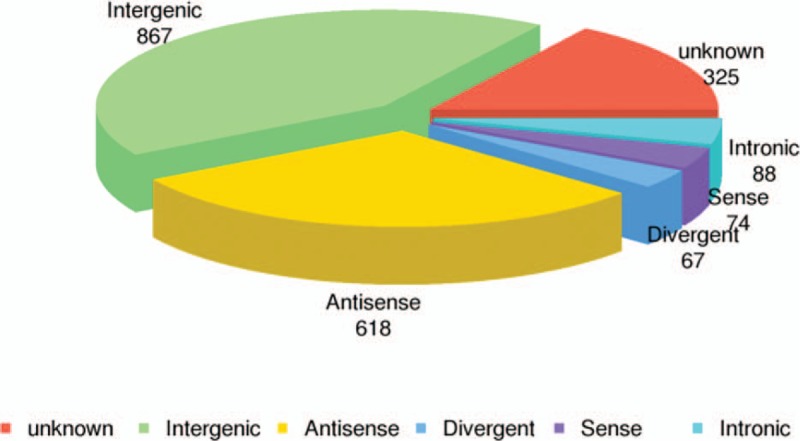
Distribution of differentially expressed lncRNAs in non-small cell lung cancer tissues.

**Figure 3:**
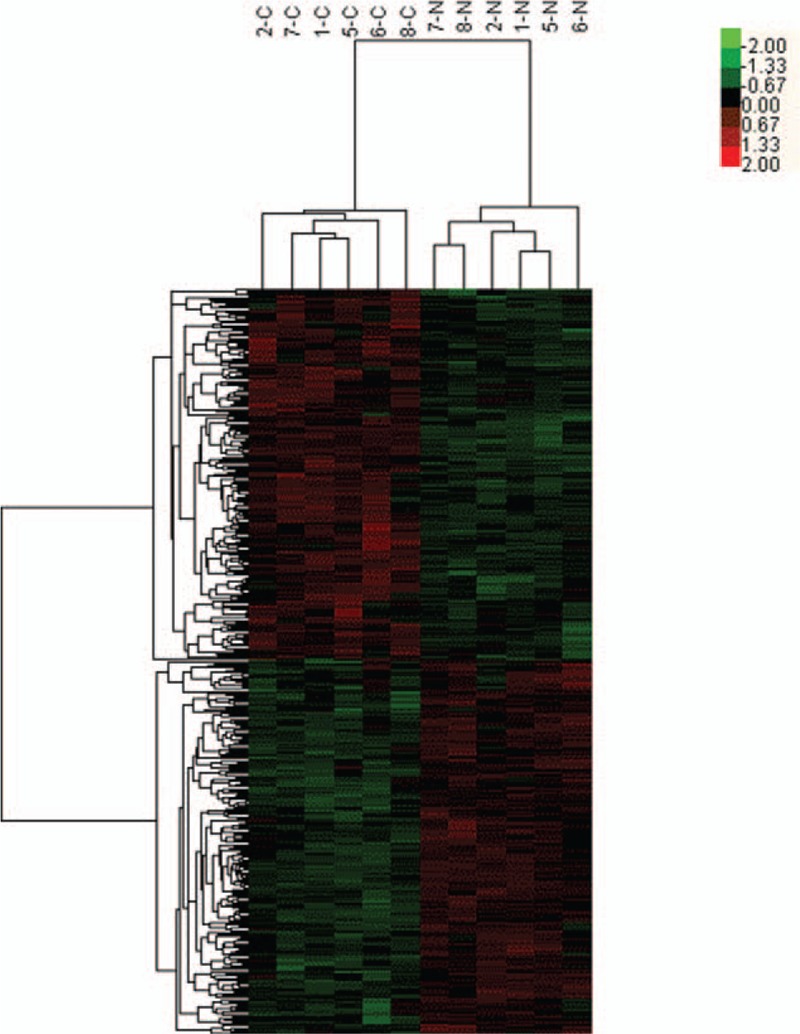
Hierarchical clustering shows a distinguishable lncRNA expression profile (including 2039 —long non-coding RNAs) between non-small cell lung cancer (NSCLC) tissues and paired adjacent normal tissues. 1-C represents NSCLC tissues of patient no. 1; 1-N represents paired adjacent normal tissues of patient No. 1 and so do 2-C, 2-N, 5-C, 5-N, 6-C, 6-N, 7-C, 7-N, 8-C, and 8-N. And see the detailed information of patient no. 1, patient no. 2, patient no. 5, patient no. 6, patient no. 7, and patient no. 8 in Table 1.

**Figure 4:**
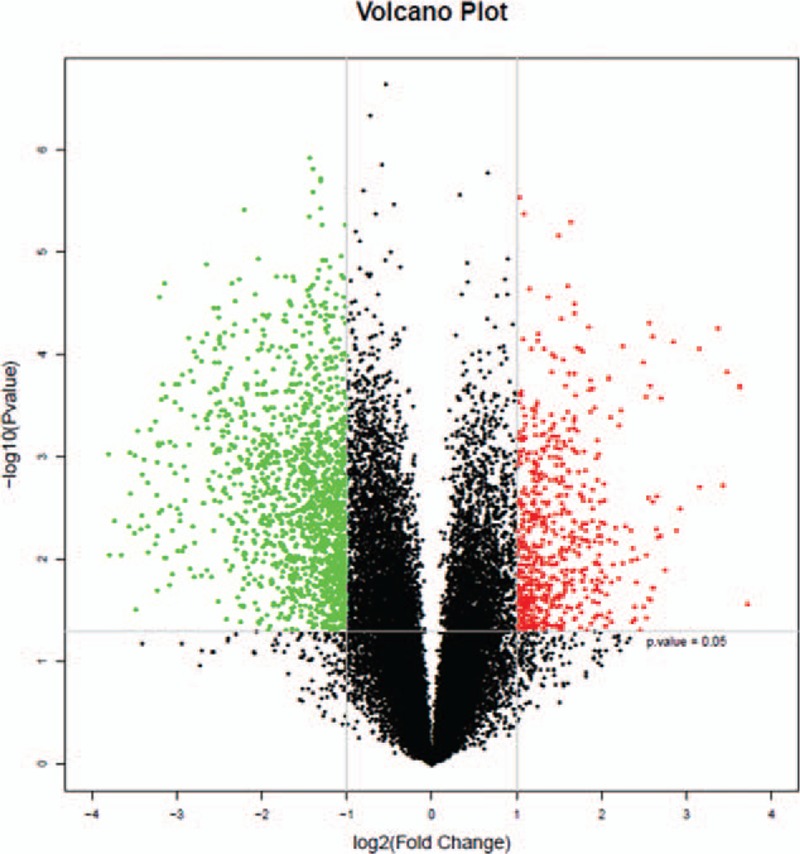
Volcano plots of the differentially expressed long non-coding RNAs (lncRNAs). The red and green points in the plot represent the significantly differentially expressed lncRNAs (including 2039 lncRNAs).

Based on the criteria of *P* values ≤.05 and absolute fold change ≥2, we identified 2039 significantly differentially expressed lncRNA probes and 3261 significantly differentially expressed mRNA probes between NSCLC and paired adjacent normal tissues (Table 2). We also analyzed the distinctive lncRNAs based on their categorizations and the results are presented in Figure 2. The categorization of most of these lncRNAs was intergenic. Then we performed these significantly differentially expressed lncRNAs through heatmap (Fig. 3) and volcano plot (Fig. 4). Through heatmap and volcano plot, we could see these significantly differentially expressed lncRNAs in NSCLC more directly and visually.

**Table 2:**
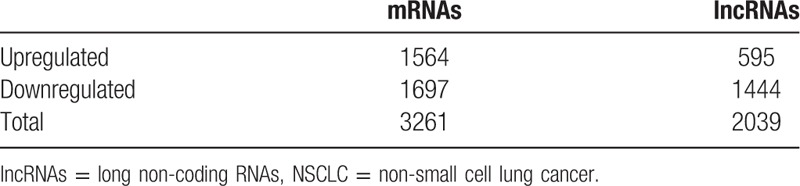
Dysregulated lncRNAs and mRNAs in NSCLC tissues.

**Figure 5:**
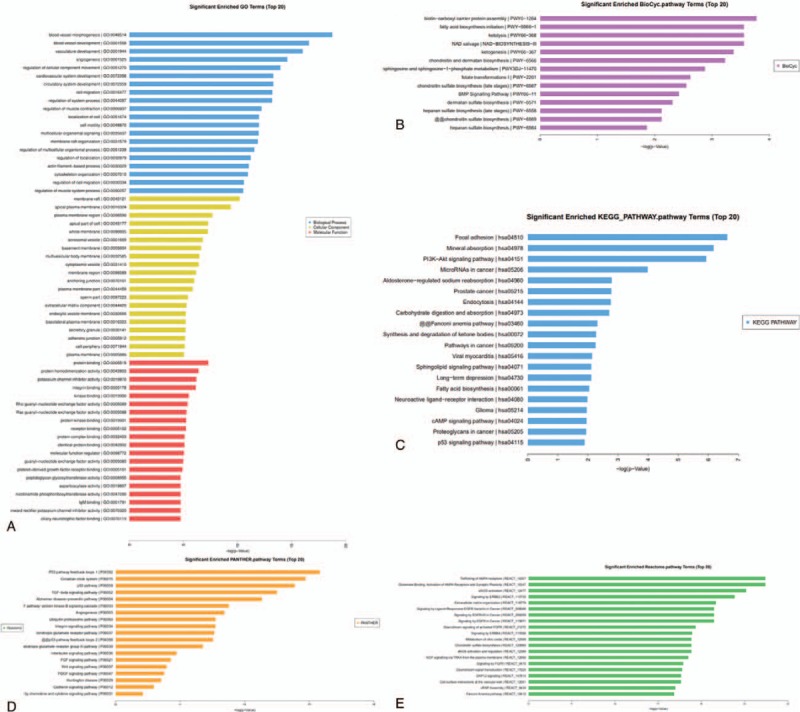
Gene ontology (GO) and pathway analyses of long non-coding RNAs co-expressed mRNAs. (A) The top 20 significantly enriched GO terms in biological processes, cellular components, and molecular functions, repectively. (B) The top 20 significantly enriched BioCyc pathway terms. (C) The top 20 significantly enriched KEGG pathway terms. (D) The top 20 significantly enriched Panther pathway terms. (E) The top 20 significantly enriched Reactome pathway terms.

To further explore the functions of lncRNAs in NSCLC, we subjected the results of the lncRNA and mRNA chip analyses to the PCC analysis, in which coexpression was considered at *P* > .90 or P < –.90. The function of lncRNAs was annotated using the GO and the BioCyc, KEGG, Panther and Reactome pathway analyses (Fig. 5A–E). From the GO and pathway analyses of lncRNA co-expressed mRNAs, we could see that these lncRNAs were associated with angiogenesis, cell motility, cell migration, cell localization, and cell adhesion, and participated in pathways of PI3K-AKT, P53, EGFR, FGFR, VEGF, and microRNAs in cancers. The results of the GO and pathway analyses confirmed that lncRNAs might play important roles in the occurrence and development of NSCLC.

Among the significantly differentially expressed lncRNAs between NSCLC and paired adjacent normal tissues, we found a most dysregulated lncRNA, LINC01614. Compared with paired adjacent normal tissues, LINC01614 was upregulated in NSCLC with an average increased fold of 22.92. LINC01614 is a 648-nt intergenic lncRNA and its gene is located in the 2q35 region. LINC01614 had not ever been characterized and its functional roles were unknown. We tried to determine the functional roles of LINC01614 through the co-expressed protein-coding genes of LINC01614 in NSCLC (Table S4). We found that among these genes, *ADAM12*^[14]^ and *BMP5*^[15]^ were associated with TGF-β signaling pathway, STEAP3^[16]^ and NEDD9^[17]^ were involved in P53 pathway, Arl4C^[18]^ was associated with Wnt signaling pathway and growth factor-Ras signaling pathway, and GPRC5A^[19]^ is a lung tumor suppressor gene and inhibits the activation of the oncogene EGFR of NSCLC. TGF-β signaling pathway, P53, Wnt, and growth factor-Ras signaling pathways are all classical pathways in the pathogenesis of NSCLC.

### 3.2 The qRT-PCR validation experiments’ results

**Figure 6:**
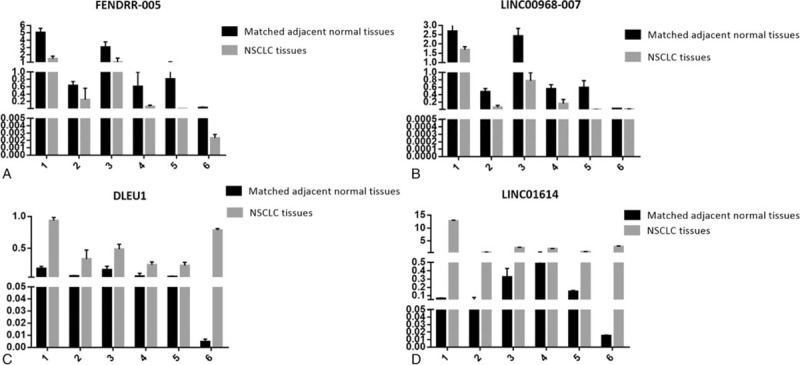
(A) Downregulation of FENDRR-005 in NSCLC compared with matched adjacent normal tissues. (B) Downregulation of LINC00968-007 in NSCLC compared with matched adjacent normal tissues. (C) Upregulation of DLEU1 in NSCLC compared with matched adjacent normal tissues. (D) Upregulation of LINC01614 in NSCLC compared with matched adjacent normal tissues. NSCLC = non-small cell lung cancer.

To validate the microarray analyses results, 4 lncRNAs (FENDRR-005, LINC00968-007, DLEU1 and LINC01614) were selected and their expression levels were obtained by qRT-PCR. As shown in Figure 6A–D, FENDRR-005 and LINC00968-007 were downregulated in NSCLC compared with matched adjacent normal tissues, and DLEU1 and LINC01614 were upregulated in NSCLC compared with matched adjacent normal tissues. Our validation experiments’ results were in accordance with the microarray analyses results.

### 3.3 Gene expression profile of NSCLC patients in the TCGA database and data analysis

**Figure 7:**
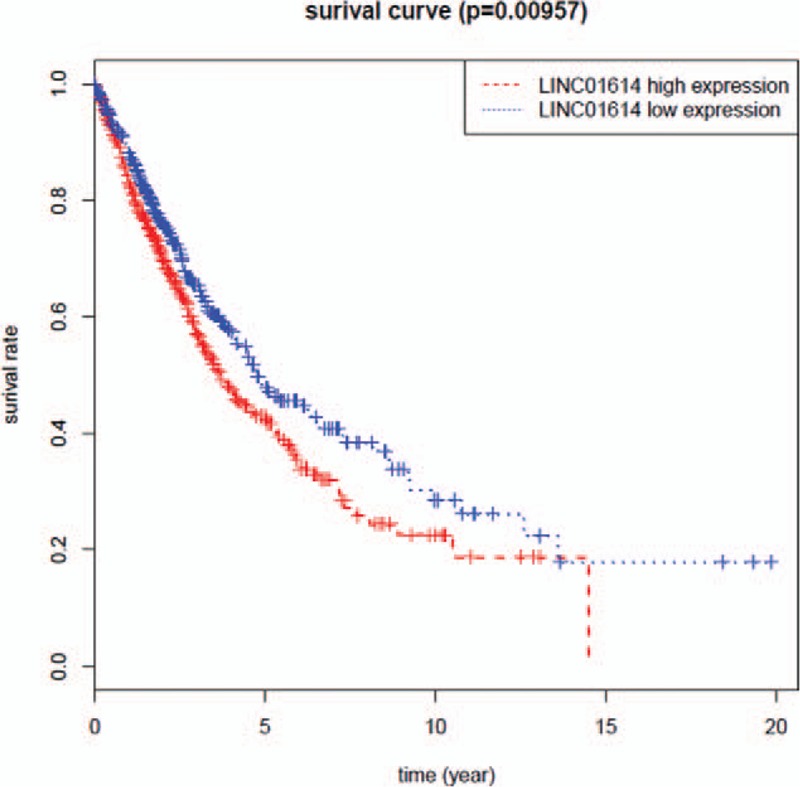
High expression of LINC01614 (based on the median) indicated poor overall survival. The threshold of “high” and “low” expression of LINC01614 was based on the median expression level of LINC01614. We defined “high” expression of LINC01614 when the expression level of LINC01614 above the median and defined “low” expression of LINC01614 when the expression level of LINC01614 below the median.

Through the usage of the TCGA database, we amplified the quantity of NSCLC patients to validate whether LINC01614 was still significantly differentially expressed in NSCLC. Then we found that compared with tumor adjacent normal tissues, LINC01614 was also significantly upregulated in NSCLC with an average increased fold of 3.21 (Table S5). Furthermore, we found that LINC01614 was significantly upregulated in both lung adenocarcinoma tissues and lung squamous cell carcinoma tissues, with an average increased fold of 3.72 (Table S6) and 2.95 (Table S7) respectively. Next, we investigated the prognostic value of LINC01614 with a large dataset of NSCLC patients from the TCGA database. High expression level of LINC01614 was significantly associated with poor OS (Fig. 7).

**Figure 8:**
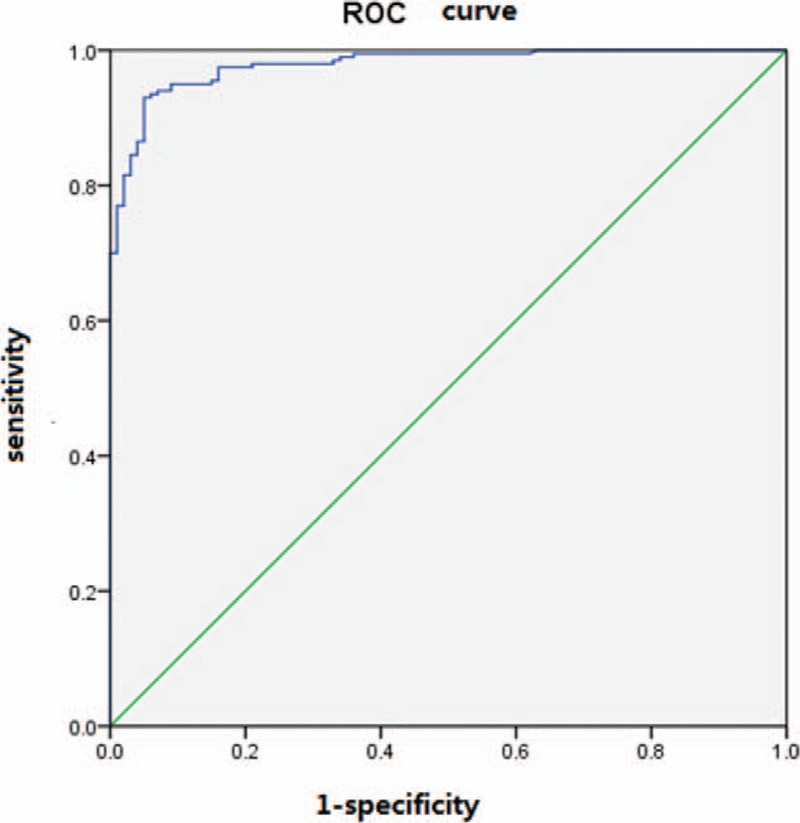
ROC curves of normalized LINC01614. The best cutoff point, according to the Youden Index, was 20.96 (fpkm value). ROC = receiver-operating characteristic.

**Figure 9:**
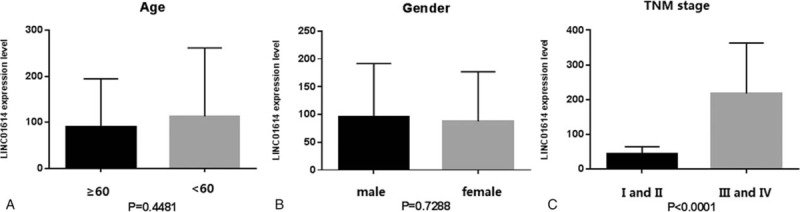
Expression level of LINC01614 between different age groups (including 187 ≥60 patients and 13 <60 patients) (A), sexes (including 118 male patients and 82 female patients) (B) and TNM stages (including 147 I and II tumor stage patients and 53 III and IV tumor stage patients) (C) in 100 lung adenocarcinoma patients and 100 lung squamous cell carcinoma patients from the TCGA database. *P* values were calculated by unpaired 2-tailed t test. Error bars represented mean ± SEM (standard error of mean). TCGA = the Cancer Genome Atlas.

To explore the diagnostic value of LINC01614, we randomly selected 100 lung adenocarcinoma patients, 100 lung squamous cell carcinoma patients, and 100 controls from the TCGA database (Table S1-3). Using ROC analysis, an AUC of 0.98 (95% confidence interval: 0.97–0.99) was obtained for NSCLC patients and controls (Fig. 8). Using the maximum Youden Index, a best cutoff point of LINC01614 value (20.96 [fpkm value]) was obtained with an optimal diagnostic value of 93% sensitivity and 95% specificity. Moreover, the expression levels of LINC01614 were positively correlated with the stage of tumor (Fig. 9C), but had no relationship with age and sex (Fig. 9A and B), suggesting that LINC01614 could be a prognostic biomarker for NSCLC. The information of NSCLC patients’ smoking status was incomplete in the TCGA database, so here we did not discuss whether the expression levels of LINC01614 were correlated with smoking status of NSCLC patients.

**Figure 10:**
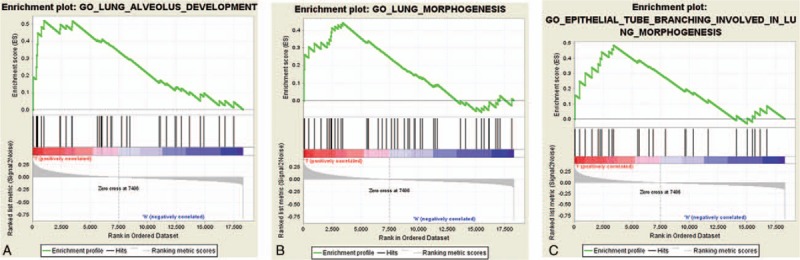
Gene set enrichment analyses of LINC01614 in NSCLC (enrichment plot). The green line represented the enrichment score (ES) for the gene-set enrichment analyses. The ranked list metric was generated by calculating the signal-to-noise ratio, which was based on the difference of means scaled according to the standard deviation. The larger the signal-to-noise ratio, the more distinct the gene expression was for each phenotype. The corresponding heat maps (Figure S1-3) showed the enrichment of genes in the gene sets. (A) Gene ontology (GO) enrichment plot of alveolus development. (B) GO enrichment analysis of lung morphogenesis. (C) GO enrichment analysis of epithelial tube branching involved in lung morphogenesis.

We then tried to further investigate the potential molecular mechanisms of LINC01614 and GSEA was conducted. As shown in Figure 10A–C, Table 3A–C and Figure S1-3, TGF-β, P53, IGF-IR, Wnt, and RTK/Ras/MAPK pathway genes were significantly enriched. And this result was in accord with the result of previous analysis in this article, suggesting LINC01614 may exert vital functions in NSCLC via modulating TGF-β, P53, IGF-IR, Wnt, and RTK/Ras/MAPK pathways.

**Table 3:**
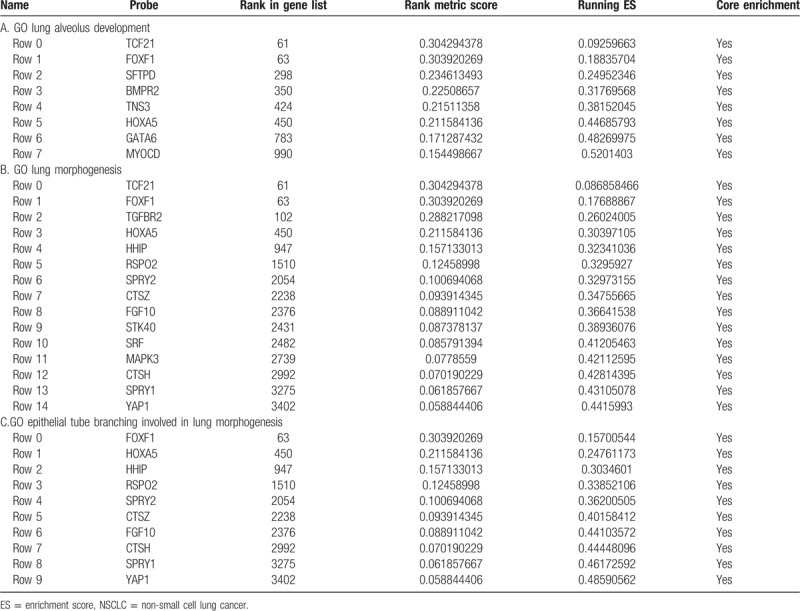
The details of gene set enrichment analyses of LINC01614 in NSCLC.

## 4 Discussion

Recent studies have revealed the contribution of lncRNAs as proto-oncogenes and tumor suppressor genes in tumorigenesis.^[20,21]^ For instance, lncRNA-PVT1 is increased in gastric cancer and promotes cancer cell proliferation by modulating the P15 and P16 signal pathways.^[22]^ MALAT1 is overexpressed in many cancers, especially in colorectal cancer, and it promotes tumor growth and metastasis through binding to SFPQ and releasing the oncogene PTBP2 from the SFPQ/PTBP2 complex.^[23]^ However, research of lncRNAs involvement in NSCLC is in its infancy and data about NSCLC-associated lncRNAs are limited. Therefore, the identification of additional NSCLC-associated lncRNAs is of great importance. They may serve as new diagnostic and prognostic tools, even new treatment targets.

With the development of high-throughput technologies, large amounts of microarray and RNA sequencing data have been obtained, but few studies have characterized lncRNA expression profile in NSCLC. In our study, we characterized the expression profile of lncRNAs in NSCLC by microarray analysis and identified 2039 significantly differentially expressed lncRNAs and 3261 significantly differentially expressed mRNAs between NSCLC and normal lung tissues, as supported by Xu et al.^[24]^ From the GO and pathway analyses of lncRNAs co-expressed mRNAs, we found that these significantly differentially expressed lncRNAs were associated with angiogenesis, cell motility, cell migration, cell localization, cell adhesion, and microRNAs in cancers and participated in PI3K-AKT, P53, EGFR, FGFR, and VEGF pathways. The results imply that they might play important roles in NSCLC.

Among these significantly differentially expressed lncRNAs, we observed that in the training set the most dysregulated lncRNA was LINC01614. Then, we confirmed the upregulation of LINC01614 in a validation cohort of NSCLC patients from the TCGA database. We investigated the prognostic value of LINC01614 using a large dataset of NSCLC patients from the TCGA database. We found that high expression of LINC01614 was significantly associated with poor OS. And the expression level of LINC01614 was positively correlated with the stage of tumor and had no relationship with age and sex. Because the smoking status data were incomplete in the TCGA database, we could not examine whether the expression level of LINC01614 was associated with smoking. Nevertheless, these data still suggest that LINC01614 has the potential to be a prognostic biomarker for NSCLC. In addition, ROC curve analysis showed that LINC01614 could provide an effective screening method for NSCLC tissues from normal tissues. As we know, many NSCLC patients are diagnosed by percutaneous transthoracic needle aspiration biopsy or bronchoscopic biopsy, but these methods obtain relatively small amounts of tumor tissues and sometimes tumor cells are crushed through these biopsy methods. Consequently, the pathologicomorphological change of tumor tissues could be not typical and difficult to be identified for the pathologists. In these cases, detecting LINC01614 expression level of biopsy tissues could be helpful for determining the nature of the lesion.

Some studies demonstrated that cell-free nucleic acids, such as DNA, microRNA, and lncRNA, are detectable in plasma and serum of cancer patients.^[25,26]^ More importantly, it has been shown that lncRNAs can remain stable in blood circulation even when subjected to hard conditions such as extreme pH and RNase A digestion.^[27]^ And the release of non-coding RNAs into the blood is thought to be associated with apoptosis and necrosis of tumor cells from the tumor microenvironment and is also the result of secretion.^[27]^ So it can be assumed that the content level of lncRNAs in tumor tissues is paralell with the content in patients’ blood. These features make lncRNAs have the potential to become ideal noninvasive biomarkers for cancer diagnosis and prognosis. For example, Tong et al^[6]^ reported that the plasma tumor-derived lncRNA POU3F3 could serve as a biomarker for the diagnosis of esophageal squamous cell carcinoma. And a recent study has also reported that detection of the lncRNA H19 in the plasma could be used to detect gastric cancer.^[28]^ Whereas LINC01614 showed excellent diagnostic performance in NSCLC tissues, we will next make further efforts to validate the favorable diagnostic efficiency of LINC01614 in patients’ blood.

Through bioinformatics methods, we found that LINC01614 might exert vital biological functions in NSCLC via modulating the TGF-β, P53, IGF-IR, Wnt, and RTK/Ras/MAPK pathways. Nevertheless, the biological functions of LINC01614 were based on bioinformatics prediction and further experiments are needed to validate these hypotheses and to investigate the more exact underlying molecular mechanisms. Based on that LINC01614 may be associated with several signaling pathways involved in the genesis and development of NSCLC, LINC01614 has the prospect of becoming a new therapeutic target for NSCLC.

## 5 Conclusions

In conclusion, our study ascertained a set of lncRNAs was differentially expressed in NSCLC compared with normal tissues using microarray analysis. And the results of the GO and pathway analyses showed that these lncRNAs might play key roles in the development of NSCLC. Moreover, in these lncRNAs, we found a most dysregulated lncRNA-LINC01614 in NSCLC. It could be used as a prognostic biomarker, and has the potential to be a diagnostic biomarker and a new therapeutic target for NSCLC. Although our findings are preliminary, we may lay the foundation for further diagnostic, prognostic, therapeutic, and functional research of lncRNAs in NSCLC.

## Acknowledgments

The authors thank all of the donors who donated to the Microarray Service at the laboratory of CapitalBio Corporation (Beijing, China).

## Author contributions

**Conceptualization:** Yan Sun.

**Data curation:** Yan Sun.

**Formal analysis:** Yan Sun, Chunhua Ling.

**Investigation:** Yan Sun.

**Methodology:** Chunhua Ling.

**Project administration:** Chunhua Ling.

**Resources:** Yan Sun.

**Software:** Chunhua Ling.

**Writing – original draft:** Yan Sun.

**Writing – review & editing:** Chunhua Ling.

## Supplementary Material

Supplemental Digital Content
