# Supplementary material for: Analysis of the long non-coding RNA LINC01614 in non-small cell lung cancer
Source: Medicine (Baltimore). 2019 Jul 26;98(30):e16437. doi: 10.1097/MD.0000000000016437 (PMC6708815; doi:10.1097/MD.0000000000016437)
Supplement: Supplemental Digital Content [file medi-98-e16437-s001.docx]

**Supplementary data**

Table S1. Clinical characteristics of 100 lung adenocarcinoma patients and the expression values of LINC01614 in the tumor tissues

| iPatients' No. in TCGA databaseen Patients' No. in TCGA databasets' No. in TCGA database | Age (the birth year) | GSexENDER | TNM stageTNM | Expression level of LINC01614 (fpkm value) |
| --- | --- | --- | --- | --- |
| TCGA-86-7953-01A-11R-2187-07 | ≥60（1942） | female | IA | 82.59 |
| TCGA-86-8280-01A-11R-2287-07 | ≥60（1957） | female | IIA | 43.26 |
| TCGA-44-A47G-01A-21R-A24H-07 | ≥60（1939） | female | IA | 91.61 |
| TCGA-64-1679-01A-21R-2066-07 | ≥60（1949） | female | IIIA | 782.95 |
| TCGA-J2-A4AG-01A-11R-A24H-07 | ≥60（1946） | female | IA | 26.89 |
| TCGA-MP-A4SW-01A-21R-A24X-07 | ≥60（1949） | male | IIB | 32.01 |
| TCGA-44-6147-01B-06R-A277-07 | ≥60（1943） | female | IA | 21.56 |
| TCGA-55-8301-01A-11R-2287-07 | ≥60（1954） | male | IB | 85.57 |
| TCGA-J2-A4HD-01A-11R-A24H-07 | ≥60（1951） | female | IA | 59.97 |
| TCGA-44-3396-01A-01R-1206-07 | ≥60（1935） | female | IIIA | 589.44 |
| TCGA-55-8092-01A-11R-2241-07 | ≥60（1937） | male | IIB | 60.06 |
| TCGA-91-6848-01A-11R-1949-07 | ≥60（1950） | male | IIIA | 204.48 |
| TCGA-78-7159-01A-11R-2039-07 | ≥60（1948） | female | IA | 73.44 |
| TCGA-49-6761-01A-31R-1949-07 | ≥60（1941） | female | IIIA | 122.17 |
| TCGA-44-2659-01A-01R-0946-07 | ≥60（1944） | female | IIB | 78.78 |
| TCGA-97-8547-01A-11R-2403-07 | ≥60（1934） | female | IIIA | 196.00 |
| TCGA-55-6985-01A-11R-1949-07 | ≥60（1946） | female | IB | 58.73 |
| TCGA-35-3615-01A-01R-0946-07 | ≥60（1950） | male | IB | 45.66 |
| TCGA-78-7152-01A-11R-2039-07 | ≥60（1938） | male | IB | 24.24 |
| TCGA-97-A4LX-01A-11R-A24X-07 | ≥60（1931） | male | IB | 50.35 |
| TCGA-86-8673-01A-11R-2403-07 | ≥60（1950） | male | IB | 31.53 |
| TCGA-55-1596-01A-01R-0946-07 | ≥60（1949） | male | IIB | 21.08 |
| TCGA-44-2656-01A-02R-A278-07 | ≥60（1950） | male | IB | 45.57 |
| TCGA-50-8457-01A-11R-2326-07 | ≥60（1948） | female | IA | 41.56 |
| TCGA-55-8614-01A-11R-2403-07 | ≥60（1935） | male | IB | 65.93 |
| TCGA-53-A4EZ-01A-12R-A24X-07 | ≥60（1949） | male | IIA | 22.97 |
| TCGA-97-A4M3-01A-11R-A24X-07 | ≥60（1943） | female | IA | 21.37 |
| TCGA-44-5644-01A-21R-2039-07 | <60（1959） | female | IB | 22.15 |
| TCGA-55-7914-01A-11R-2170-07 | ≥60（1940） | female | IIA | 77.58 |
| TCGA-86-A456-01A-11R-A24H-07 | ≥60（1934） | female | IA | 13.94 |
| TCGA-73-4668-01A-01R-1206-07 | ≥60（1943） | female | IIB | 98.97 |
| TCGA-95-7947-01A-11R-2187-07 | ≥60（1944） | male | IA | 29.50 |
| TCGA-64-5779-01A-01R-1628-07 | ≥60（1948） | male | IIIA | 74.41 |
| TCGA-55-6984-01A-11R-1949-07 | ≥60（1935） | female | IIB | 19.10 |
| TCGA-44-6776-01A-11R-1858-07 | ≥60（1945） | female | IA | 9.69 |
| TCGA-05-4418-01A-01R-1206-07 | ≥60（1939） | male | IIIA | 139.04 |
| TCGA-MP-A4TA-01A-21R-A24X-07 | ≥60（1931） | female | IA | 24.69 |
| TCGA-69-7761-01A-11R-2170-07 | ≥60（1927） | male | IB | 32.67 |
| TCGA-38-4625-01A-01R-1206-07 | ≥60（1936） | female | IB | 50.70 |
| TCGA-55-6968-01A-11R-1949-07 | ≥60（1943） | male | IV | 93.26 |
| TCGA-95-8494-01A-11R-2326-07 | ≥60（1945） | male | IIA | 31.06 |
| TCGA-44-3917-01B-02R-A277-07 | <60（1976） | female | IB | 30.59 |
| TCGA-86-8076-01A-31R-2241-07 | <60（1969） | male | IA | 41.28 |
| TCGA-L9-A443-01A-12R-A24H-07 | ≥60（1949） | female | IA | 43.51 |
| TCGA-49-4506-01A-01R-1206-07 | ≥60（1929） | female | IIB | 66.99 |
| TCGA-49-6745-01A-11R-1858-07 | ≥60（1929） | male | IIIA | 249.16 |
| TCGA-55-A492-01A-11R-A24H-07 | ≥60（1942） | female | IA | 4.59 |
| TCGA-67-3773-01A-01R-0946-07 | ≥60（1925） | female | IB | 64.27 |
| TCGA-55-8507-01A-11R-2403-07 | <60（1959） | male | IA | 41.28 |
| TCGA-55-7573-01A-11R-2039-07 | ≥60（1939） | female | IA | 27.73 |
| TCGA-95-7944-01A-11R-2187-07 | ≥60（1940） | male | IA | 26.03 |
| TCGA-97-8175-01A-11R-2287-07 | ≥60（1956） | female | IB | 51.78 |
| TCGA-44-4112-01B-06R-A277-07 | ≥60（1950） | female | IB | 25.14 |
| TCGA-50-6594-01A-11R-1755-07 | ≥60（1930） | female | IIIA | 133.00 |
| TCGA-55-6971-01A-11R-1949-07 | ≥60（1951） | female | IB | 47.04 |
| TCGA-50-5066-02A-11R-2090-07 | ≥60（1937） | male | IB | 18.03 |
| TCGA-50-5072-01A-21R-1858-07 | ≥60（1935） | male | IIIA | 219.32 |
| TCGA-86-8668-01A-11R-2403-07 | ≥60（1951） | female | IA | 36.49 |
| TCGA-86-8281-01A-11R-2287-07 | ≥60（1936） | male | IA | 39.56 |
| TCGA-49-4512-01A-21R-1858-07 | ≥60（1938） | female | IIIA | 167.12 |
| TCGA-50-6595-01A-12R-1858-07 | ≥60（1935） | female | IIIA | 296.50 |
| TCGA-05-4426-01A-01R-1206-07 | ≥60（1937） | male | IB | 35.89 |
| TCGA-78-7145-01A-11R-2039-07 | ≥60（1948） | female | IV | 132.77 |
| TCGA-35-5375-01A-01R-1628-07 | ≥60（1949） | male | IIIA | 135.29 |
| TCGA-91-6830-01A-11R-1949-07 | ≥60（1937） | female | IIA | 50.26 |
| TCGA-55-7994-01A-11R-2187-07 | ≥60（1930） | male | IIB | 54.52 |
| TCGA-97-7554-01A-11R-2039-07 | ≥60（1927） | female | IIIA | 283.92 |
| TCGA-95-7039-01A-11R-1949-07 | ≥60（1957） | female | IIB | 84.81 |
| TCGA-78-8660-01A-11R-2403-07 | ≥60（1936） | male | IIB | 47.58 |
| TCGA-MP-A4T8-01A-11R-A24X-07 | ≥60（1938） | male | IIIA | 94.30 |
| TCGA-91-A4BC-01A-11R-A24H-07 | ≥60（1953） | male | IIA | 47.92 |
| TCGA-44-6147-01A-11R-1755-07 | ≥60（1943） | female | IA | 48.14 |
| TCGA-55-8085-01A-11R-2241-07 | ≥60（1947） | male | IA | 40.12 |
| TCGA-4B-A93V-01B-11R-A39D-07 | <60（1961） | female | IA | 59.87 |
| TCGA-55-6712-01A-11R-1858-07 | ≥60（1939） | male | IIA | 76.93 |
| TCGA-05-4398-01A-01R-1206-07 | <60（1959） | female | IIIB | 99.57 |
| TCGA-86-6851-01A-11R-1949-07 | ≥60（1938） | female | IIA | 51.55 |
| TCGA-44-A47A-01A-21R-A24H-07 | ≥60（1934） | female | IB | 41.53 |
| TCGA-55-6983-01A-11R-1949-07 | ≥60（1924） | male | IIB | 65.75 |
| TCGA-44-3919-01A-02R-1107-07 | ≥60（1939） | female | IA | 41.02 |
| TCGA-55-7907-01A-11R-2170-07 | ≥60（1934） | male | IIA | 47.76 |
| TCGA-55-7911-01A-11R-2170-07 | ≥60（1941） | female | IA | 24.46 |
| TCGA-97-8179-01A-11R-2287-07 | ≥60（1940） | male | IA | 15.44 |
| TCGA-55-A491-01A-11R-A24H-07 | ≥60（1931） | female | IA | 43.62 |
| TCGA-99-AA5R-01A-11R-A39D-07 | ≥60（1943） | female | IA | 14.57 |
| TCGA-05-4420-01A-01R-1206-07 | <60（1967） | male | IB | 30.30 |
| TCGA-05-5420-01A-01R-1628-07 | ≥60（1941） | male | IIIA | 188.45 |
| TCGA-73-4666-01A-01R-1206-07 | ≥60（1957） | female | IV | 126.91 |
| TCGA-86-8073-01A-11R-2241-07 | ≥60（1953） | male | IB | 46.04 |
| TCGA-73-4670-01A-01R-1206-07 | ≥60（1941） | female | IV | 438.98 |
| TCGA-44-7659-01A-11R-2066-07 | ≥60（1940） | male | IA | 12.18 |
| TCGA-38-6178-01A-11R-1755-07 | ≥60（1940） | female | IIIA | 110.17 |
| TCGA-97-A4M5-01A-11R-A24X-07 | ≥60（1929） | male | IA | 37.38 |
| TCGA-95-7948-01A-11R-2187-07 | <60（1969） | female | IB | 40.43 |
| TCGA-64-1681-01A-11R-2066-07 | ≥60（1947） | female | IA | 28.84 |
| TCGA-60-2724-01A-01R-0851-07 | <60（1961） | male | IIIA | 557.88 |
| TCGA-99-8025-01A-11R-2241-07 | ≥60（1938） | female | IIIA | 116.75 |
| TCGA-62-8398-01A-11R-2326-07 | ≥60（1952） | male | IIIA | 86.04 |
| TCGA-44-8120-01A-11R-2241-07 | ≥60（1953） | male | IB | 41.96 |
| TCGA-44-2656-01A-02R-0946-07 | ≥60（1950） | male | IB | 57.73 |

Table S2. Clinical characteristics of 100 lung squamous cell carcinoma patients and the expression values of LINC01614 in the tumor tissues

| iPatients' No. in TCGA databasee | Age (the birth year) | SexE | TNM stageT | Expression level of LINC01614(fpkm value) |
| --- | --- | --- | --- | --- |
| TCGA-22-1002-01A-01R-0692-07 | ≥60（1932） | male | IA | 45.29 |
| TCGA-43-A56U-01A-11R-A26W-07 | ≥60（1936） | female | IA | 51.22 |
| TCGA-98-8022-01A-11R-2247-07 | ≥60（1950） | male | IA | 9.87 |
| TCGA-85-7710-01A-11R-2125-07 | ≥60（1952） | female | IA | 35.67 |
| TCGA-77-7138-01A-41R-2045-07 | ≥60（1932） | male | IB | 21.72 |
| TCGA-77-A5G8-01B-11R-A27Q-07 | ≥60（1939） | male | IIB | 59.29 |
| TCGA-77-A5GF-01A-21R-A27Q-07 | ≥60（1941） | male | IIA | 39.02 |
| TCGA-22-1011-01A-01R-0692-07 | ≥60（1928） | male | IB | 47.92 |
| TCGA-22-5477-01A-01R-1635-07 | ≥60（1939） | male | IA | 38.51 |
| TCGA-33-4583-01A-01R-1443-07 | ≥60（1926） | male | IA | 18.44 |
| TCGA-77-8009-01A-11R-2187-07 | ≥60（1939） | male | IIB | 63.47 |
| TCGA-21-1079-01A-01R-0692-07 | ≥60（1924） | male | IIIA | 239.52 |
| TCGA-21-A5DI-01A-31R-A26W-07 | ≥60（1929） | male | IA | 21.44 |
| TCGA-21-1080-01A-01R-0692-07 | ≥60（1930） | male | IB | 62.59 |
| TCGA-56-A4ZJ-01A-11R-A262-07 | ≥60（1937） | female | IA | 58.23 |
| TCGA-56-7823-01B-11R-2247-07 | ≥60（1953） | female | IIA | 14.03 |
| TCGA-77-7140-01A-41R-2045-07 | ≥60（1933） | female | IIB | 41.15 |
| TCGA-77-6842-01A-11R-1949-07 | ≥60（1921） | male | IIB | 59.80 |
| TCGA-98-A539-01A-31R-A262-07 | ≥60（1948） | male | IIB | 44.81 |
| TCGA-66-2771-01A-01R-0980-07 | ≥60（1947） | male | IIB | 89.46 |
| TCGA-46-6025-01A-11R-1820-07 | ≥60（1939） | male | IIB | 60.10 |
| TCGA-94-7943-01A-11R-2187-07 | ≥60（1931） | male | IA | 36.51 |
| TCGA-98-A53C-01A-11R-A262-07 | ≥60（1935） | female | IA | 14.49 |
| TCGA-33-4582-01A-01R-1443-07 | ≥60（1945） | male | IA | 21.65 |
| TCGA-77-8146-01A-11R-2247-07 | ≥60（1933） | male | IA | 36.00 |
| TCGA-33-4533-01A-01R-1201-07 | ≥60（1925） | female | IB | 27.62 |
| TCGA-77-8154-01A-11R-2247-07 | ≥60（1941） | male | IA | 9.00 |
| TCGA-92-8063-01A-11R-2247-07 | <60（1959） | male | IIIA | 85.64 |
| TCGA-85-8072-01A-31R-2247-07 | ≥60（1937） | male | IA | 23.99 |
| TCGA-96-7545-01A-21R-2045-07 | ≥60（1935） | male | IA | 20.56 |
| TCGA-85-6798-01A-11R-1949-07 | ≥60（1954） | male | IIIA | 121.74 |
| TCGA-34-2596-01A-01R-0851-07 | ≥60（1934） | male | IIB | 55.88 |
| TCGA-22-4594-01A-01R-1201-07 | ≥60（1942） | female | IIIA | 248.56 |
| TCGA-66-2800-01A-01R-1201-07 | ≥60（1934） | male | IIIB | 93.52 |
| TCGA-66-2790-01A-01R-0980-07 | ≥60（1936） | male | IIB | 55.29 |
| TCGA-77-A5G1-01A-11R-A27Q-07 | ≥60（1927） | male | IIIA | 103.96 |
| TCGA-68-7755-01A-11R-2125-07 | ≥60（1951） | female | IIA | 62.17 |
| TCGA-43-7657-01A-31R-2125-07 | ≥60（1943） | female | IA | 58.82 |
| TCGA-94-A4VJ-01A-11R-A24Z-07 | ≥60（1941） | female | IA | 55.52 |
| TCGA-L3-A4E7-01A-11R-A24Z-07 | ≥60（1941） | male | IB | 24.12 |
| TCGA-58-8387-01A-11R-2296-07 | ≥60（1950） | male | IIA | 55.85 |
| TCGA-96-8170-01A-11R-2296-07 | ≥60（1936） | female | IIA | 42.89 |
| TCGA-94-A5I6-01A-21R-A27Q-07 | ≥60（1950） | male | IIB | 55.26 |
| TCGA-22-4595-01A-01R-1201-07 | ≥60（1945） | male | IIIA | 291.39 |
| TCGA-18-3417-01A-01R-1443-07 | ≥60（1941） | male | IV | 566.21 |
| TCGA-77-A5GH-01A-11R-A27Q-07 | ≥60（1930） | male | IB | 55.14 |
| TCGA-37-3789-01A-01R-0980-07 | ≥60（1941） | male | IB | 50.79 |
| TCGA-39-5030-01A-01R-1443-07 | ≥60（1925） | female | IIIA | 129.69 |
| TCGA-22-0944-01A-01R-0692-07 | ≥60（1939） | male | IB | 39.46 |
| TCGA-37-3783-01A-01R-1201-07 | ≥60（1955） | male | IIIA | 157.83 |
| TCGA-66-2795-01A-02R-0980-07 | ≥60（1940） | male | IIIB | 364.78 |
| TCGA-43-8116-01A-11R-2247-07 | ≥60（1938） | male | IA | 45.02 |
| TCGA-77-A5GB-01B-11R-A27Q-07 | ≥60（1920） | male | IB | 65.18 |
| TCGA-NC-A5HE-01A-11R-A26W-07 | ≥60（1948） | male | IIB | 63.83 |
| TCGA-43-3394-01A-01R-0980-07 | ≥60（1957） | male | IB | 68.09 |
| TCGA-21-5784-01A-01R-1635-07 | ≥60（1928） | female | IB | 33.09 |
| TCGA-58-8388-01A-11R-2326-07 | ≥60（1950） | male | IB | 35.73 |
| TCGA-22-5472-01A-01R-1635-07 | ≥60（1938） | male | IB | 21.73 |
| TCGA-18-3410-01A-01R-0980-07 | ≥60（1923） | male | IIB | 61.87 |
| TCGA-68-7756-01A-11R-2125-07 | ≥60（1927） | male | IIIA | 175.44 |
| TCGA-22-4605-01A-21R-2125-07 | ≥60（1925） | female | IB | 60.07 |
| TCGA-85-8276-01A-11R-2296-07 | ≥60（1949） | male | IIA | 82.97 |
| TCGA-58-8390-01A-11R-2326-07 | ≥60（1940） | male | IIA | 78.19 |
| TCGA-90-7964-01A-21R-2187-07 | ≥60（1941） | male | IB | 34.20 |
| TCGA-85-8353-01A-21R-2296-07 | ≥60（1939） | male | IIIA | 212.40 |
| TCGA-37-4132-01A-01R-1100-07 | ≥60（1949） | female | IV | 106.68 |
| TCGA-33-6737-01A-11R-1820-07 | ≥60（1937） | male | IIIA | 90.91 |
| TCGA-22-5479-01A-31R-1949-07 | ≥60（1940） | male | IB | 45.53 |
| TCGA-34-5927-01A-11R-1820-07 | ≥60（1939） | female | IA | 28.38 |
| TCGA-34-8455-01A-11R-2326-07 | ≥60（1944） | male | IV | 229.10 |
| TCGA-77-7465-01A-11R-2045-07 | ≥60（1952） | male | IIA | 61.83 |
| TCGA-52-7810-01A-11R-2125-07 | ≥60（1948） | female | IIB | 32.86 |
| TCGA-77-7335-01A-11R-2045-07 | ≥60（1934） | female | IIIB | 415.06 |
| TCGA-70-6722-01A-11R-1820-07 | <60（1963） | male | IIIA | 240.42 |
| TCGA-85-8355-01A-11R-2296-07 | ≥60（1948） | male | IA | 25.61 |
| TCGA-56-8628-01A-11R-2403-07 | ≥60（1934） | male | IA | 55.63 |
| TCGA-21-5787-01A-01R-1635-07 | ≥60（1944） | male | IIIA | 110.29 |
| TCGA-18-3411-01A-01R-0980-07 | ≥60（1942） | female | IIIA | 191.15 |
| TCGA-33-AASD-01A-11R-A405-07 | ≥60（1918） | male | IA | 38.42 |
| TCGA-77-A5G3-01A-31R-A27Q-07 | ≥60（1939） | male | IIB | 61.50 |
| TCGA-39-5028-01A-01R-1443-07 | ≥60（1931） | male | IIIA | 242.23 |
| TCGA-90-A59Q-01A-11R-A26W-07 | ≥60（1951） | female | IIA | 70.27 |
| TCGA-NC-A5HP-01A-11R-A26W-07 | ≥60（1942） | male | IV | 276.15 |
| TCGA-85-8351-01A-11R-2296-07 | ≥60（1939） | male | IIA | 86.31 |
| TCGA-56-7222-01A-11R-2045-07 | ≥60（1951） | male | IB | 64.25 |
| TCGA-22-5471-01A-01R-1635-07 | ≥60（1930） | male | IB | 56.89 |
| TCGA-85-A53L-01A-21R-A26W-07 | ≥60（1949） | male | IIA | 61.11 |
| TCGA-96-A4JK-01A-11R-A24Z-07 | ≥60（1947） | male | IIA | 41.43 |
| TCGA-77-7142-01A-11R-2045-07 | ≥60（1948） | female | IB | 53.89 |
| TCGA-33-4587-01A-11R-2125-07 | ≥60（1940） | female | IB | 62.46 |
| TCGA-66-2786-01A-01R-0851-07 | ≥60（1940） | female | IA | 66.85 |
| TCGA-18-5595-01A-01R-1635-07 | <60（1958） | male | IB | 45.10 |
| TCGA-43-2581-01A-01R-0851-07 | <60（1962） | female | IIIA | 175.52 |
| TCGA-56-8309-01A-11R-2296-07 | ≥60（1946） | male | IA | 61.94 |
| TCGA-21-5782-01A-01R-1635-07 | ≥60（1936） | female | IB | 42.16 |
| TCGA-77-8145-01A-11R-2247-07 | ≥60（1927） | male | IIIB | 238.37 |
| TCGA-77-8128-01A-11R-2247-07 | ≥60（1939） | male | IIIA | 217.35 |
| TCGA-46-3768-01A-01R-0980-07 | ≥60（1952） | male | IIIA | 210.33 |
| TCGA-85-7699-01A-11R-2125-07 | ≥60（1938） | male | IIIA | 204.37 |
| TCGA-43-2576-01A-01R-A32Z-07 | ≥60（1947） | female | IIIA | 194.30 |

Table S3. The expression values of LINC01614 in 100 patients’ tumor adjacent normal tissues

| Patients' No. in TCGA database | expression level of LINC01614 (fpkm value) |
| --- | --- |
| TCGA-56-7823-11A-01R-2247-07 | 7.29 |
| TCGA-55-6982-11A-01R-1949-07 | 6.96 |
| TCGA-33-4587-11A-01R-2125-07 | 9.09 |
| TCGA-56-8309-11A-01R-2296-07 | 4.58 |
| TCGA-43-6143-11A-01R-1820-07 | 13.36 |
| TCGA-44-6145-11A-01R-1858-07 | 7.89 |
| TCGA-55-6970-11A-01R-1949-07 | 2.88 |
| TCGA-44-6146-11A-01R-1858-07 | 5.08 |
| TCGA-56-7579-11A-01R-2045-07 | 12.61 |
| TCGA-50-5930-11A-01R-1755-07 | 0.16 |
| TCGA-55-6975-11A-01R-1949-07 | 6.59 |
| TCGA-22-5481-11A-01R-1949-07 | 4.82 |
| TCGA-91-6829-11A-01R-1858-07 | 2.51 |
| TCGA-50-5935-11A-01R-1858-07 | 15.75 |
| TCGA-43-5670-11A-01R-2125-07 | 12.90 |
| TCGA-44-6144-11A-01R-1755-07 | 0.09 |
| TCGA-50-5932-11A-01R-1755-07 | 10.84 |
| TCGA-44-3398-11B-01R-1758-07 | 1.70 |
| TCGA-55-6969-11A-01R-1949-07 | 0.05 |
| TCGA-38-4627-11A-01R-1758-07 | 18.55 |
| TCGA-49-6744-11A-01R-1858-07 | 11.91 |
| TCGA-34-8454-11A-01R-2326-07 | 10.09 |
| TCGA-44-6777-11A-01R-1858-07 | 6.03 |
| TCGA-38-4626-11A-01R-1758-07 | 1.92 |
| TCGA-51-4079-11A-01R-1758-07 | 5.16 |
| TCGA-49-4490-11A-01R-1858-07 | 16.12 |
| TCGA-56-8623-11A-01R-A28V-07 | 15.97 |
| TCGA-49-6745-11A-01R-1858-07 | 10.55 |
| TCGA-55-6979-11A-01R-1949-07 | 11.46 |
| TCGA-22-5491-11A-01R-1858-07 | 4.52 |
| TCGA-44-2665-11A-01R-1758-07 | 18.94 |
| TCGA-22-5482-11A-01R-1635-07 | 20.15 |
| TCGA-22-4609-11A-01R-2125-07 | 2.67 |
| TCGA-49-6761-11A-01R-1949-07 | 6.06 |
| TCGA-55-6981-11A-01R-1949-07 | 5.42 |
| TCGA-55-6978-11A-01R-1949-07 | 1.56 |
| TCGA-22-5489-11A-01R-1635-07 | 6.85 |
| TCGA-91-6828-11A-01R-1858-07 | 11.37 |
| TCGA-33-6737-11A-01R-1820-07 | 12.54 |
| TCGA-39-5040-11A-01R-2125-07 | 2.00 |
| TCGA-50-5939-11A-01R-1628-07 | 8.41 |
| TCGA-55-6971-11A-01R-1949-07 | 4.19 |
| TCGA-50-5933-11A-01R-1755-07 | 4.33 |
| TCGA-60-2709-11A-01R-1820-07 | 4.81 |
| TCGA-77-7335-11A-01R-2045-07 | 26.17 |
| TCGA-56-8201-11A-01R-2247-07 | 10.26 |
| TCGA-43-7658-11A-01R-2125-07 | 3.29 |
| TCGA-55-6985-11A-01R-1949-07 | 4.23 |
| TCGA-77-7338-11A-01R-2045-07 | 16.57 |
| TCGA-51-4081-11A-01R-1758-07 | 11.87 |
| TCGA-50-6595-11A-01R-1858-07 | 3.99 |
| TCGA-44-6776-11A-01R-1858-07 | 5.87 |
| TCGA-91-6849-11A-01R-1949-07 | 2.98 |
| TCGA-55-6972-11A-01R-1949-07 | 4.34 |
| TCGA-44-2657-11A-01R-1758-07 | 5.42 |
| TCGA-22-5483-11A-11R-1820-07 | 9.13 |
| TCGA-22-5478-11A-11R-1635-07 | 9.77 |
| TCGA-50-5936-11A-01R-1628-07 | 3.45 |
| TCGA-77-7142-11A-01R-2045-07 | 4.26 |
| TCGA-56-7731-11A-01R-2125-07 | 7.07 |
| TCGA-43-6647-11A-01R-1820-07 | 3.41 |
| TCGA-56-7582-11A-01R-2045-07 | 15.09 |
| TCGA-90-7767-11A-01R-2125-07 | 7.47 |
| TCGA-44-2655-11A-01R-1758-07 | 12.50 |
| TCGA-22-5472-11A-01R-1635-07 | 24.57 |
| TCGA-77-8008-11A-01R-2187-07 | 40.68 |
| TCGA-50-5931-11A-01R-1858-07 | 3.71 |
| TCGA-91-6836-11A-01R-1858-07 | 4.59 |
| TCGA-91-6831-11A-02R-1858-07 | 10.44 |
| TCGA-90-6837-11A-01R-1949-07 | 20.84 |
| TCGA-56-7580-11A-01R-2045-07 | 30.15 |
| TCGA-44-6148-11A-01R-1858-07 | 5.06 |
| TCGA-56-7730-11A-01R-2125-07 | 2.82 |
| TCGA-44-2668-11A-01R-1758-07 | 4.34 |
| TCGA-77-7337-11A-01R-2045-07 | 4.45 |
| TCGA-43-6771-11A-01R-1820-07 | 3.71 |
| TCGA-38-4625-11A-01R-1758-07 | 6.31 |
| TCGA-55-6968-11A-01R-1949-07 | 7.81 |
| TCGA-56-8082-11A-01R-2247-07 | 3.47 |
| TCGA-44-3396-11A-01R-1758-07 | 10.46 |
| TCGA-44-5645-11A-01R-1628-07 | 2.15 |
| TCGA-77-7138-11A-01R-2045-07 | 1.45 |
| TCGA-43-7657-11A-01R-2125-07 | 2.57 |
| TCGA-91-6835-11A-01R-1858-07 | 15.61 |
| TCGA-73-4676-11A-01R-1755-07 | 17.10 |
| TCGA-38-4632-11A-01R-1755-07 | 6.96 |
| TCGA-55-6980-11A-01R-1949-07 | 6.00 |
| TCGA-85-7710-11A-01R-2125-07 | 4.56 |
| TCGA-91-6847-11A-01R-1949-07 | 1.69 |
| TCGA-49-4512-11A-01R-1858-07 | 9.91 |
| TCGA-58-8386-11A-01R-2296-07 | 12.10 |
| TCGA-44-6147-11A-01R-1858-07 | 7.99 |
| TCGA-44-2662-11A-01R-1758-07 | 4.96 |
| TCGA-55-6986-11A-01R-1949-07 | 1.34 |
| TCGA-92-7340-11A-01R-2045-07 | 4.16 |
| TCGA-44-6778-11A-01R-1858-07 | 6.62 |
| TCGA-22-5471-11A-01R-1635-07 | 3.50 |
| TCGA-56-8083-11A-01R-2247-07 | 6.83 |
| TCGA-55-6984-11A-01R-1949-07 | 1.50 |
| TCGA-22-4593-11A-01R-1820-07 | 34.48 |

# Table S4. The co-expressed protein-coding genes of LINC01614 in NSCLC

| co-expressed protein-coding genes | the Pearson correlation coefficient | P-value |
| --- | --- | --- |
| ADAM12 | 0.94 | 0.004 |
| ALG1 | -0.95 | 0.003 |
| STEAP3 | -0.93 | 0.006 |
| AATK | -0.92 | 0.010 |
| MPG | -0.93 | 0.008 |
| TBX2 | -0.96 | 0.003 |
| FAM105A | -0.95 | 0.004 |
| GPRC5A | -0.95 | 0.004 |
| FCRL6 | -0.91 | 0.011 |
| KIR2DL5A | -0.90 | 0.014 |
| CELF2 | -0.91 | 0.011 |
| ROBO2 | -0.98 | 0.000 |
| NEDD9 | -0.96 | 0.002 |
| KDELC1 | 0.93 | 0.008 |
| SOGA3 | -0.97 | 0.001 |
| GPR180 | 0.92 | 0.008 |
| MARCO | -0.97 | 0.002 |
| FAP | 0.95 | 0.003 |
| MCEMP1 | -0.97 | 0.001 |
| KRT10 | 0.94 | 0.006 |
| SLC11A1 | -0.92 | 0.010 |
| ARL4C | 0.96 | 0.003 |
| NPAS2 | 0.93 | 0.008 |
| FNDC1 | 0.92 | 0.010 |
| RANBP3L | -0.91 | 0.012 |
| CACNA1D | -0.96 | 0.003 |
| MMP7 | -0.98 | 0.001 |
| IL18R1 | -0.94 | 0.005 |
| NREP | 0.90 | 0.014 |
| MYOZ1 | -0.96 | 0.003 |
| SRD5A3 | -0.90 | 0.014 |
| PALD1 | -0.94 | 0.005 |
| RBMS2 | -0.98 | 0.001 |
| ATOH8 | -0.92 | 0.010 |
| TBX4 | -0.96 | 0.003 |
| FAM189A2 | -0.97 | 0.002 |
| BPHL | 0.91 | 0.012 |
| ENTPD7 | 0.92 | 0.009 |
| CDH24 | -0.94 | 0.004 |
| C16orf13 | -0.98 | 0.000 |
| PAPSS2 | -0.91 | 0.011 |
| PLOD2 | 0.92 | 0.008 |
| HCN3 | 0.95 | 0.003 |
| BMP5 | -0.95 | 0.004 |
| KIT | -0.91 | 0.011 |
| INMT | -0.93 | 0.008 |
| KCNJ8 | 1.00 | 0.000 |
| EFCC1 | -0.96 | 0.002 |
| DSP | 0.92 | 0.010 |

Table S5. The significantly dysregulated lncRNAs in NSCLC in the TCGA database

| LncRNA | logFC | logCPM | P-value | FDR |
| --- | --- | --- | --- | --- |
| RP11-371A19.2 | -5.35 | 5.67 | 0 | 0 |
| LANCL1-AS1 | -3.96 | 6.71 | 0 | 0 |
| AC093110.3 | -3.62 | 7.77 | 0 | 0 |
| HSPC324 | -4.03 | 5.81 | 2.54E-316 | 5.87E-313 |
| LINC00968 | -3.98 | 7.18 | 2.42E-303 | 4.46E-300 |
| AP001189.4 | -3.56 | 6.13 | 2.32E-295 | 3.56E-292 |
| FENDRR | -4.06 | 9.94 | 2.61E-292 | 3.44E-289 |
| RP5-826L7.1 | -3.82 | 3.49 | 3.61E-269 | 4.16E-266 |
| MIR3945HG | -3.88 | 6.57 | 6.24E-257 | 6.40E-254 |
| LINC01082 | -4.37 | 3.98 | 4.86E-255 | 4.49E-252 |
| RP11-805I24.3 | -4.53 | 4.75 | 6.88E-245 | 5.78E-242 |
| HID1-AS1 | -3.35 | 4.67 | 4.13E-241 | 3.18E-238 |
| RP4-575N6.5 | -3.17 | 3.75 | 1.46E-239 | 1.03E-236 |
| RP11-354P11.2 | -4.36 | 3.99 | 1.04E-224 | 6.86E-222 |
| AC018647.3 | -3.19 | 5.19 | 1.67E-212 | 1.03E-209 |
| LINC02016 | -5.51 | 5.39 | 1.65E-208 | 9.52E-206 |
| RP11-613D13.8 | -3.30 | 5.68 | 5.47E-207 | 2.97E-204 |
| RP4-575N6.4 | -2.83 | 4.94 | 4.01E-201 | 2.06E-198 |
| RP1-78O14.1 | -3.60 | 7.63 | 2.01E-199 | 9.79E-197 |
| PCAT19 | -2.70 | 9.38 | 3.41E-198 | 1.58E-195 |
| RP11-598F7.3 | -3.78 | 6.57 | 1.27E-188 | 5.59E-186 |
| RP11-672A2.4 | -2.87 | 6.13 | 2.56E-182 | 1.07E-179 |
| ADAMTS9-AS1 | -3.31 | 6.85 | 2.74E-182 | 1.10E-179 |
| SMIM25 | -2.74 | 10.51 | 2.16E-176 | 8.30E-174 |
| LINC01290 | -2.38 | 4.88 | 4.15E-176 | 1.53E-173 |
| AC011899.9 | -2.70 | 8.40 | 2.98E-175 | 1.06E-172 |
| LINC01996 | -4.71 | 5.77 | 1.36E-171 | 4.65E-169 |
| RP11-287F9.2 | -4.73 | 3.76 | 5.59E-171 | 1.84E-168 |
| LINC00656 | -3.48 | 4.30 | 1.62E-169 | 4.99E-167 |
| AC006273.4 | -2.82 | 4.63 | 4.36E-163 | 1.30E-160 |
| RP11-286H15.1 | -3.36 | 5.26 | 3.23E-162 | 9.31E-160 |
| RP1-18D14.7 | -2.98 | 4.75 | 3.11E-160 | 8.60E-158 |
| RP11-354P11.4 | -3.90 | 3.42 | 3.17E-160 | 8.60E-158 |
| RP11-389C8.2 | -2.18 | 8.40 | 5.24E-159 | 1.38E-156 |
| CTD-2369P2.8 | -3.24 | 7.87 | 1.16E-157 | 2.99E-155 |
| RP11-51B23.3 | -2.59 | 3.78 | 2.09E-150 | 5.21E-148 |
| LINC01936 | -2.90 | 8.45 | 8.82E-148 | 2.14E-145 |
| RP11-1024P17.1 | -2.33 | 6.79 | 2.45E-147 | 5.79E-145 |
| RP11-475O23.2 | -3.84 | 3.46 | 1.97E-146 | 4.43E-144 |
| RP11-434D9.1 | -3.14 | 6.00 | 1.58E-145 | 3.46E-143 |
| LINC01197 | -2.40 | 6.23 | 2.75E-145 | 5.91E-143 |
| AP000438.2 | -3.14 | 3.67 | 3.85E-145 | 8.08E-143 |
| RP11-100L22.1 | -3.44 | 3.41 | 1.52E-144 | 3.12E-142 |
| MED4-AS1 | -2.36 | 4.41 | 3.82E-143 | 7.66E-141 |
| RP11-295M18.6 | -2.71 | 4.02 | 2.32E-142 | 4.56E-140 |
| RP11-136H19.1 | -2.49 | 4.03 | 1.27E-140 | 2.45E-138 |
| LINC00702 | -2.68 | 7.46 | 3.14E-140 | 5.92E-138 |
| PACRG-AS3 | -4.17 | 3.68 | 6.07E-139 | 1.12E-136 |
| LINC01352 | -2.59 | 4.71 | 3.07E-137 | 5.56E-135 |
| RP11-2N1.3 | -4.10 | 2.71 | 1.42E-136 | 2.52E-134 |
| AC004947.2 | -3.52 | 5.43 | 4.33E-136 | 7.55E-134 |
| LINC00163 | -3.59 | 4.41 | 1.09E-134 | 1.83E-132 |
| RP11-264B14.1 | -3.05 | 3.68 | 2.24E-134 | 3.69E-132 |
| RP11-79H23.3 | -2.76 | 6.79 | 4.98E-132 | 8.07E-130 |
| RP11-352D13.6 | -3.19 | 6.24 | 2.18E-130 | 3.47E-128 |
| RP11-4B16.3 | -2.76 | 3.65 | 2.99E-125 | 4.68E-123 |
| LINC00891 | -2.87 | 4.92 | 3.44E-124 | 5.30E-122 |
| RP11-335L23.5 | -2.55 | 3.76 | 1.76E-122 | 2.66E-120 |
| RP13-1016M1.2 | -2.50 | 4.37 | 3.18E-120 | 4.74E-118 |
| RP4-564M11.2 | -2.55 | 4.61 | 1.66E-119 | 2.43E-117 |
| RP11-714G18.1 | -2.58 | 4.68 | 5.66E-118 | 8.04E-116 |
| TBX5-AS1 | -2.23 | 9.27 | 1.46E-114 | 2.04E-112 |
| RP11-35J10.7 | -3.21 | 3.68 | 3.37E-113 | 4.64E-111 |
| LINC01572 | 3.05 | 6.58 | 1.44E-110 | 1.89E-108 |
| LINC00511 | 3.61 | 11.73 | 2.65E-110 | 3.45E-108 |
| LINC00551 | -2.98 | 5.60 | 2.99E-110 | 3.83E-108 |
| RP11-452C13.1 | -2.45 | 4.65 | 4.80E-110 | 6.08E-108 |
| RP11-35J10.6 | -3.27 | 3.35 | 2.90E-108 | 3.57E-106 |
| RP11-544M22.1 | -3.70 | 5.20 | 7.62E-108 | 9.26E-106 |
| LINC01863 | -3.75 | 4.30 | 5.74E-106 | 6.71E-104 |
| C1orf140 | -2.96 | 3.21 | 1.75E-105 | 2.02E-103 |
| LINC01645 | -3.20 | 3.82 | 3.44E-105 | 3.92E-103 |
| MGC27382 | -3.52 | 6.05 | 1.05E-102 | 1.18E-100 |
| C5orf64 | -2.51 | 3.32 | 6.93E-101 | 7.71E-99 |
| AC090616.2 | -2.68 | 7.69 | 1.10E-100 | 1.21E-98 |
| AP002856.5 | -4.78 | 5.22 | 1.55E-100 | 1.69E-98 |
| DDX11-AS1 | 2.66 | 6.74 | 5.36E-100 | 5.69E-98 |
| RP11-325L12.6 | -2.06 | 4.72 | 8.47E-98 | 8.88E-96 |
| RP11-594N15.3 | -2.34 | 5.36 | 1.19E-97 | 1.24E-95 |
| PCAT6 | 2.88 | 9.08 | 6.46E-97 | 6.55E-95 |
| RP11-439L18.1 | -2.36 | 3.90 | 1.30E-96 | 1.31E-94 |
| LL22NC03-104C7.1 | -3.49 | 3.07 | 1.99E-96 | 1.98E-94 |
| ADAMTS9-AS2 | -2.43 | 6.62 | 8.29E-96 | 8.15E-94 |
| RP11-532F6.3 | -2.07 | 7.06 | 5.22E-95 | 5.07E-93 |
| RP11-356N1.2 | -2.46 | 4.27 | 1.14E-94 | 1.10E-92 |
| CTC-296K1.3 | -2.26 | 4.04 | 1.41E-94 | 1.34E-92 |
| AC007128.1 | 5.90 | 6.81 | 4.06E-94 | 3.82E-92 |
| RP11-830F9.6 | -2.20 | 3.49 | 7.58E-94 | 7.07E-92 |
| FOXD3-AS1 | 7.06 | 7.16 | 1.07E-92 | 9.88E-91 |
| AC133785.1 | 7.13 | 6.56 | 2.94E-92 | 2.69E-90 |
| PVT1 | 2.51 | 10.62 | 5.78E-92 | 5.23E-90 |
| LINC00472 | -2.23 | 6.33 | 1.43E-91 | 1.28E-89 |
| RP11-627G18.1 | -2.46 | 4.33 | 2.59E-91 | 2.27E-89 |
| RP11-236L14.2 | -2.05 | 4.83 | 7.06E-91 | 6.15E-89 |
| TFAP2A-AS1 | 3.18 | 6.47 | 9.73E-91 | 8.40E-89 |
| LINC01070 | -3.86 | 2.57 | 1.38E-90 | 1.18E-88 |
| CTD-2515H24.2 | -2.73 | 4.62 | 2.19E-90 | 1.83E-88 |
| RP11-27M24.2 | -2.58 | 4.50 | 2.57E-90 | 2.13E-88 |
| AC006129.1 | -2.05 | 5.10 | 4.80E-90 | 3.95E-88 |
| RP11-352D13.5 | -2.70 | 4.02 | 7.73E-90 | 6.31E-88 |
| AC002398.12 | -2.91 | 2.84 | 1.21E-89 | 9.81E-88 |
| HLX-AS1 | -2.54 | 3.31 | 2.07E-89 | 1.66E-87 |
| CTD-2527I21.15 | 6.17 | 7.30 | 1.17E-88 | 9.26E-87 |
| CADM3-AS1 | -2.71 | 5.90 | 2.67E-88 | 2.07E-86 |
| RP11-246K15.1 | -3.34 | 4.09 | 4.12E-87 | 3.12E-85 |
| LINC00844 | -3.30 | 2.97 | 1.94E-86 | 1.46E-84 |
| RP1-251M9.3 | -3.97 | 3.56 | 4.36E-86 | 3.25E-84 |
| BBOX1-AS1 | 6.40 | 8.75 | 1.11E-85 | 8.16E-84 |
| LINC01977 | 4.44 | 6.66 | 3.87E-85 | 2.81E-83 |
| FAM83A-AS1 | 6.44 | 8.51 | 5.87E-85 | 4.24E-83 |
| LINC01412 | -2.52 | 3.00 | 2.66E-84 | 1.90E-82 |
| RP11-783K16.5 | 3.55 | 7.55 | 8.79E-84 | 6.24E-82 |
| RP4-594A5.1 | 6.62 | 5.34 | 1.15E-83 | 8.13E-82 |
| RP11-77A13.1 | -4.04 | 7.01 | 4.19E-83 | 2.93E-81 |
| RP5-839B4.8 | -3.25 | 7.01 | 3.12E-82 | 2.15E-80 |
| LINC01624 | -2.36 | 4.02 | 6.31E-82 | 4.31E-80 |
| VPS9D1-AS1 | 3.39 | 9.68 | 9.98E-82 | 6.78E-80 |
| RP11-49G2.3 | -2.32 | 4.10 | 1.13E-81 | 7.61E-80 |
| PGM5-AS1 | -2.97 | 3.51 | 1.62E-81 | 1.08E-79 |
| LINC01165 | -2.62 | 3.24 | 3.22E-81 | 2.12E-79 |
| RP11-672A2.5 | -2.51 | 2.80 | 3.33E-81 | 2.18E-79 |
| LINC01985 | -2.95 | 2.97 | 6.22E-81 | 4.05E-79 |
| LINC02104 | -2.33 | 3.92 | 1.12E-80 | 7.24E-79 |
| RP11-95I16.2 | -3.22 | 4.27 | 1.21E-80 | 7.74E-79 |
| RP11-108L7.15 | 2.58 | 5.86 | 1.30E-79 | 8.15E-78 |
| KCNMB2-AS1 | 6.82 | 9.18 | 2.36E-79 | 1.47E-77 |
| FEZF1-AS1 | 5.99 | 9.49 | 6.52E-79 | 4.04E-77 |
| AC004540.4 | -2.28 | 6.04 | 5.05E-78 | 3.11E-76 |
| RP11-64B16.4 | -3.04 | 3.17 | 2.77E-77 | 1.68E-75 |
| RUNDC3A-AS1 | 3.49 | 7.29 | 3.93E-77 | 2.37E-75 |
| CTD-3010D24.3 | 5.04 | 7.10 | 8.74E-77 | 5.24E-75 |
| RP11-89B16.1 | -2.33 | 3.89 | 9.67E-77 | 5.76E-75 |
| CTD-2373J6.1 | -2.04 | 3.78 | 8.86E-76 | 5.25E-74 |
| ZFPM2-AS1 | 5.22 | 8.67 | 1.15E-75 | 6.78E-74 |
| RP11-863P13.4 | -2.56 | 4.16 | 2.27E-75 | 1.33E-73 |
| CTD-2510F5.4 | 2.80 | 8.15 | 2.88E-75 | 1.67E-73 |
| RP11-384F7.2 | -3.46 | 4.18 | 6.47E-75 | 3.74E-73 |
| RP6-65G23.3 | 2.70 | 8.48 | 7.39E-75 | 4.24E-73 |
| LINC01506 | -2.38 | 4.27 | 5.70E-74 | 3.23E-72 |
| NAV2-AS2 | -2.73 | 4.25 | 1.06E-73 | 5.95E-72 |
| LINC02014 | 3.27 | 6.40 | 2.33E-72 | 1.26E-70 |
| RP11-677M14.3 | -2.04 | 6.69 | 1.02E-71 | 5.47E-70 |
| CTC-296K1.4 | -2.27 | 3.64 | 1.32E-71 | 6.97E-70 |
| RP11-429J17.7 | 2.56 | 6.68 | 1.50E-71 | 7.85E-70 |
| CTD-2530N21.5 | -3.44 | 3.86 | 2.35E-71 | 1.23E-69 |
| LINC01703 | 2.72 | 6.68 | 2.70E-71 | 1.40E-69 |
| LLNLR-470E3.1 | -2.01 | 5.96 | 3.69E-71 | 1.90E-69 |
| RP11-796E10.1 | 5.09 | 5.39 | 4.17E-71 | 2.14E-69 |
| RP11-244M2.1 | 4.38 | 7.27 | 5.19E-71 | 2.65E-69 |
| SNHG4 | 2.39 | 8.40 | 6.22E-71 | 3.16E-69 |
| NPSR1-AS1 | 6.14 | 5.41 | 1.11E-70 | 5.58E-69 |
| RP11-111E14.1 | -2.18 | 4.70 | 1.38E-70 | 6.92E-69 |
| RP11-312J18.6 | -3.36 | 3.92 | 2.09E-70 | 1.04E-68 |
| RP5-1103B4.3 | -3.63 | 2.69 | 3.53E-70 | 1.75E-68 |
| RP11-546J1.1 | 2.25 | 5.26 | 1.47E-69 | 7.22E-68 |
| LINC01836 | -2.07 | 6.76 | 1.77E-69 | 8.67E-68 |
| RP11-494M8.4 | -2.63 | 4.96 | 2.25E-69 | 1.09E-67 |
| LINC00211 | -2.52 | 3.46 | 3.19E-69 | 1.53E-67 |
| LINC02185 | -2.51 | 5.84 | 3.34E-69 | 1.60E-67 |
| AC135178.7 | -2.35 | 2.95 | 5.65E-69 | 2.69E-67 |
| TYMSOS | 2.85 | 6.84 | 7.37E-69 | 3.49E-67 |
| SLC2A1-AS1 | 2.95 | 7.32 | 4.72E-68 | 2.20E-66 |
| RP4-724E16.2 | 2.10 | 7.35 | 5.52E-68 | 2.56E-66 |
| AC007743.1 | -2.06 | 7.50 | 2.60E-67 | 1.19E-65 |
| RP11-650L12.2 | 3.58 | 7.22 | 3.56E-67 | 1.63E-65 |
| RP5-965F6.2 | -2.06 | 3.56 | 3.74E-67 | 1.69E-65 |
| RP11-738B7.1 | 3.58 | 4.26 | 7.00E-67 | 3.14E-65 |
| RP13-463N16.6 | 5.74 | 6.38 | 7.24E-67 | 3.23E-65 |
| AC079630.4 | -2.63 | 9.47 | 1.06E-66 | 4.70E-65 |
| RP11-44B19.1 | -2.53 | 3.20 | 6.94E-66 | 3.07E-64 |
| LHFPL3-AS2 | -2.89 | 9.27 | 9.11E-66 | 4.01E-64 |
| RP1-140C12.2 | -2.11 | 3.14 | 1.05E-65 | 4.58E-64 |
| PGM5P4-AS1 | -2.55 | 3.71 | 9.50E-65 | 4.08E-63 |
| RP11-8L2.1 | 7.16 | 6.62 | 1.06E-64 | 4.52E-63 |
| RP3-332B22.1 | -3.03 | 3.10 | 1.09E-64 | 4.63E-63 |
| RP5-908M14.10 | 2.10 | 6.50 | 1.16E-64 | 4.90E-63 |
| TMPO-AS1 | 2.04 | 8.51 | 1.47E-64 | 6.13E-63 |
| RP11-540A21.2 | 2.44 | 6.68 | 1.69E-63 | 6.94E-62 |
| RP11-370I10.2 | -2.72 | 3.34 | 1.80E-63 | 7.34E-62 |
| LINC00670 | -2.69 | 3.03 | 2.70E-63 | 1.09E-61 |
| MNX1-AS1 | 5.00 | 7.22 | 7.33E-63 | 2.94E-61 |
| RP1-15D23.2 | -2.53 | 3.06 | 1.02E-62 | 4.06E-61 |
| CYP4A22-AS1 | 2.72 | 5.33 | 1.27E-62 | 5.05E-61 |
| AP000251.3 | 4.08 | 5.79 | 1.35E-62 | 5.32E-61 |
| HOXC-AS2 | 5.36 | 6.35 | 2.54E-62 | 9.99E-61 |
| OGFRP1 | 2.15 | 6.81 | 4.67E-62 | 1.83E-60 |
| RP11-742B18.1 | 4.75 | 7.33 | 5.19E-62 | 2.02E-60 |
| LINC02147 | -2.28 | 3.25 | 8.45E-62 | 3.28E-60 |
| RP11-157F20.3 | 3.29 | 3.93 | 1.57E-61 | 6.07E-60 |
| CASC9 | 6.73 | 9.72 | 2.08E-61 | 7.97E-60 |
| RP11-968A15.2 | 2.18 | 5.88 | 4.00E-61 | 1.51E-59 |
| LINC01031 | -2.16 | 3.31 | 7.07E-61 | 2.67E-59 |
| CTD-2531D15.4 | -2.67 | 4.33 | 9.80E-61 | 3.66E-59 |
| LINC02163 | 6.80 | 5.49 | 1.98E-60 | 7.34E-59 |
| RP11-125O18.1 | -2.46 | 4.07 | 2.10E-60 | 7.76E-59 |
| RP11-501J20.5 | -2.42 | 3.44 | 2.22E-60 | 8.16E-59 |
| RP11-12G12.7 | 2.04 | 9.54 | 6.59E-60 | 2.41E-58 |
| KB-1460A1.1 | 2.83 | 5.58 | 7.03E-60 | 2.55E-58 |
| CTD-3224I3.3 | -2.29 | 2.84 | 1.05E-59 | 3.80E-58 |
| AC002066.1 | -2.25 | 5.36 | 1.14E-59 | 4.11E-58 |
| RP11-544L8__B.4 | -2.42 | 3.93 | 1.50E-59 | 5.38E-58 |
| CALML3-AS1 | 5.29 | 8.40 | 2.86E-59 | 1.02E-57 |
| KB-1448A5.1 | -2.53 | 2.97 | 3.61E-59 | 1.28E-57 |
| RP11-386G11.5 | 2.20 | 6.40 | 5.67E-59 | 1.99E-57 |
| RP3-523K23.2 | 5.75 | 10.52 | 9.23E-59 | 3.22E-57 |
| RP11-480A16.1 | 2.36 | 7.97 | 9.62E-59 | 3.34E-57 |
| RP11-493L12.5 | 3.51 | 5.06 | 1.71E-58 | 5.91E-57 |
| RP11-539E17.5 | 5.23 | 4.65 | 1.73E-58 | 5.95E-57 |
| RP11-357D18.1 | -2.99 | 6.86 | 2.38E-58 | 8.18E-57 |
| CTB-66B24.1 | -2.24 | 2.75 | 2.62E-58 | 8.95E-57 |
| RP11-25H12.1 | 6.50 | 4.90 | 2.92E-58 | 9.94E-57 |
| RP3-512B11.3 | 2.74 | 7.98 | 3.62E-58 | 1.23E-56 |
| SFTA1P | -2.64 | 10.78 | 4.23E-58 | 1.43E-56 |
| AC015849.16 | 3.55 | 5.91 | 4.53E-58 | 1.53E-56 |
| RP11-336A10.5 | 4.48 | 5.45 | 5.94E-58 | 1.98E-56 |
| RP11-108K3.2 | 5.57 | 4.85 | 1.23E-57 | 4.03E-56 |
| LINC00607 | -2.04 | 6.91 | 1.53E-57 | 5.00E-56 |
| MELTF-AS1 | 2.43 | 8.49 | 1.68E-57 | 5.48E-56 |
| GATA6-AS1 | -2.22 | 6.44 | 2.15E-57 | 6.93E-56 |
| Z83851.4 | 2.14 | 7.86 | 3.89E-57 | 1.24E-55 |
| RP11-635O16.2 | -3.22 | 6.16 | 5.10E-57 | 1.62E-55 |
| RP11-161I6.2 | 5.16 | 6.72 | 7.18E-57 | 2.26E-55 |
| CTD-2227E11.1 | 2.56 | 6.50 | 9.07E-57 | 2.84E-55 |
| RP11-98G7.1 | 3.95 | 5.39 | 1.05E-56 | 3.26E-55 |
| RP11-108K3.1 | 4.99 | 5.15 | 1.52E-56 | 4.71E-55 |
| AC006159.5 | -2.56 | 3.01 | 1.75E-56 | 5.40E-55 |
| RP11-779O18.1 | -2.14 | 2.90 | 1.90E-56 | 5.81E-55 |
| RP11-474G23.3 | 2.17 | 5.28 | 6.99E-56 | 2.12E-54 |
| RP11-624L4.1 | 3.20 | 7.63 | 1.02E-55 | 3.05E-54 |
| RP11-390F4.3 | 2.96 | 7.91 | 1.65E-55 | 4.93E-54 |
| RP11-932O9.10 | 2.32 | 5.10 | 3.85E-55 | 1.14E-53 |
| HOXC13-AS | 7.24 | 6.71 | 4.66E-55 | 1.37E-53 |
| AC011286.1 | -2.84 | 3.53 | 7.89E-55 | 2.29E-53 |
| RP11-57A1.1 | 3.27 | 4.07 | 9.51E-55 | 2.75E-53 |
| RP11-560J1.2 | 2.25 | 5.99 | 1.23E-54 | 3.53E-53 |
| SLC12A9-AS1 | 2.26 | 5.77 | 1.38E-54 | 3.94E-53 |
| AC123886.2 | -2.49 | 2.80 | 1.45E-54 | 4.13E-53 |
| C2orf48 | 2.86 | 6.25 | 1.56E-54 | 4.43E-53 |
| CTD-2523D13.2 | 3.80 | 6.47 | 2.95E-54 | 8.31E-53 |
| LINC01711 | 3.63 | 5.08 | 6.22E-54 | 1.74E-52 |
| RP11-2N1.2 | -3.16 | 4.79 | 1.50E-53 | 4.17E-52 |
| RP6-191P20.4 | 4.81 | 5.04 | 1.63E-53 | 4.50E-52 |
| AFAP1-AS1 | 5.76 | 12.20 | 1.80E-53 | 4.95E-52 |
| CTD-2562J17.7 | -2.16 | 3.80 | 1.88E-53 | 5.16E-52 |
| BLACAT1 | 2.84 | 8.90 | 2.14E-53 | 5.86E-52 |
| PCAT7 | 3.32 | 6.87 | 2.92E-53 | 7.98E-52 |
| AC007182.6 | -2.47 | 3.97 | 4.36E-53 | 1.18E-51 |
| SPRY4-IT1 | -2.10 | 5.04 | 8.58E-53 | 2.32E-51 |
| CTC-480C2.1 | 6.91 | 5.29 | 1.12E-52 | 3.00E-51 |
| AC009262.2 | 4.96 | 3.61 | 1.48E-52 | 3.96E-51 |
| RP11-191L9.4 | 7.35 | 6.11 | 1.52E-52 | 4.07E-51 |
| RP11-174G6.1 | 4.04 | 6.28 | 2.53E-52 | 6.71E-51 |
| LINC00887 | 3.97 | 6.03 | 2.98E-52 | 7.88E-51 |
| DLX6-AS1 | 6.09 | 8.76 | 4.04E-52 | 1.06E-50 |
| RP1-27K12.4 | 4.31 | 6.51 | 4.55E-52 | 1.19E-50 |
| AC195454.1 | -2.25 | 4.45 | 4.78E-52 | 1.25E-50 |
| LINC01748 | 5.45 | 7.48 | 7.92E-52 | 2.05E-50 |
| RP11-485G7.6 | 2.65 | 4.72 | 9.87E-52 | 2.54E-50 |
| CTD-2033A16.3 | 2.91 | 5.20 | 1.10E-51 | 2.83E-50 |
| LINC00519 | 5.24 | 8.09 | 1.62E-51 | 4.14E-50 |
| CTD-3247F14.2 | -2.09 | 4.84 | 2.86E-51 | 7.20E-50 |
| LINC00491 | 6.67 | 6.91 | 4.45E-51 | 1.11E-49 |
| RP11-295G20.2 | 2.74 | 9.88 | 5.05E-51 | 1.26E-49 |
| RP11-387H17.6 | -2.32 | 3.13 | 5.71E-51 | 1.42E-49 |
| LINC01633 | 6.19 | 4.49 | 9.30E-51 | 2.30E-49 |
| RP11-203H2.2 | -2.85 | 2.86 | 1.09E-50 | 2.68E-49 |
| POU6F2-AS2 | 6.87 | 5.96 | 1.15E-50 | 2.81E-49 |
| RP11-328K4.1 | 6.16 | 5.84 | 1.49E-50 | 3.64E-49 |
| RP11-794G24.1 | 3.13 | 5.95 | 1.64E-50 | 4.00E-49 |
| RP11-346D19.1 | 6.51 | 4.84 | 2.46E-50 | 5.93E-49 |
| CTA-384D8.35 | 2.46 | 8.50 | 3.34E-50 | 7.98E-49 |
| HAGLROS | 3.72 | 7.48 | 6.94E-50 | 1.65E-48 |
| RP11-238K6.1 | -2.84 | 6.74 | 1.35E-49 | 3.19E-48 |
| CASC8 | 4.07 | 7.46 | 2.22E-49 | 5.25E-48 |
| LINC00896 | 2.89 | 5.92 | 2.36E-49 | 5.57E-48 |
| RP11-10N16.3 | 3.26 | 4.30 | 2.79E-49 | 6.56E-48 |
| CTD-3216D2.5 | 2.12 | 4.89 | 3.46E-49 | 8.12E-48 |
| RP13-577H12.2 | -3.37 | 3.28 | 4.12E-49 | 9.60E-48 |
| CTD-2319I12.5 | -2.22 | 3.09 | 5.03E-49 | 1.16E-47 |
| RP5-1056H1.2 | 2.71 | 6.93 | 7.87E-49 | 1.81E-47 |
| FIRRE | 3.30 | 7.43 | 1.10E-48 | 2.52E-47 |
| AC005537.2 | 4.55 | 7.05 | 1.20E-48 | 2.73E-47 |
| CTC-499J9.1 | 6.16 | 6.27 | 1.43E-48 | 3.24E-47 |
| DUXAP8 | 3.02 | 9.06 | 1.78E-48 | 4.03E-47 |
| RP13-497K6.1 | -2.72 | 3.04 | 3.06E-48 | 6.89E-47 |
| TRPM2-AS | 4.14 | 7.66 | 4.24E-48 | 9.51E-47 |
| AC069513.4 | 2.82 | 4.70 | 4.84E-48 | 1.08E-46 |
| RP11-470P21.2 | 4.68 | 4.55 | 9.78E-48 | 2.17E-46 |
| CTD-2023N9.1 | -2.57 | 2.82 | 1.08E-47 | 2.38E-46 |
| AF127577.8 | 6.91 | 5.69 | 1.16E-47 | 2.56E-46 |
| KDM4A-AS1 | 2.04 | 7.58 | 1.24E-47 | 2.71E-46 |
| AC079630.2 | -2.79 | 6.51 | 1.41E-47 | 3.06E-46 |
| RP1-244F24.1 | 2.02 | 5.66 | 1.52E-47 | 3.30E-46 |
| MYO16-AS1 | -2.71 | 5.93 | 1.86E-47 | 4.02E-46 |
| CTA-989H11.1 | 2.01 | 6.51 | 2.12E-47 | 4.57E-46 |
| RP4-694A7.2 | 4.85 | 4.62 | 3.20E-47 | 6.81E-46 |
| RP5-836N17.4 | 3.04 | 4.16 | 3.32E-47 | 7.04E-46 |
| RP11-496D24.2 | 4.38 | 4.04 | 3.34E-47 | 7.07E-46 |
| LINC00942 | 6.86 | 10.27 | 3.77E-47 | 7.97E-46 |
| AC145343.2 | 2.50 | 6.49 | 4.26E-47 | 8.96E-46 |
| CTA-280A3.2 | 5.97 | 4.49 | 5.18E-47 | 1.09E-45 |
| LINC01208 | 4.65 | 3.99 | 2.35E-46 | 4.84E-45 |
| AC013264.2 | -2.39 | 6.47 | 3.68E-46 | 7.56E-45 |
| LINC01460 | 3.43 | 6.69 | 5.04E-46 | 1.03E-44 |
| RP11-964E11.2 | 3.38 | 7.03 | 5.57E-46 | 1.14E-44 |
| LINC01833 | 6.74 | 7.22 | 5.73E-46 | 1.17E-44 |
| LINC01614 | 3.21 | 7.76 | 7.83E-46 | 1.59E-44 |
| HHIP-AS1 | -2.04 | 8.36 | 8.24E-46 | 1.67E-44 |
| RP5-1063M23.2 | 3.02 | 5.26 | 8.80E-46 | 1.78E-44 |
| RP11-59D5__B.2 | 3.90 | 8.23 | 8.92E-46 | 1.80E-44 |
| MYOSLID | 3.35 | 7.79 | 1.16E-45 | 2.33E-44 |
| CTC-327F10.4 | 4.77 | 4.01 | 1.24E-45 | 2.47E-44 |
| RP11-512N21.3 | -2.22 | 3.77 | 1.76E-45 | 3.50E-44 |
| RP11-114B7.6 | 5.19 | 4.81 | 2.07E-45 | 4.10E-44 |
| LINC01980 | 8.07 | 8.16 | 2.08E-45 | 4.12E-44 |
| LINC01705 | 3.73 | 5.54 | 2.33E-45 | 4.61E-44 |
| RP11-657O9.1 | 4.99 | 6.41 | 2.69E-45 | 5.31E-44 |
| RP11-21L23.3 | 2.61 | 5.73 | 2.72E-45 | 5.35E-44 |
| CTD-2532K18.2 | 4.47 | 4.53 | 2.85E-45 | 5.59E-44 |
| RP11-336K24.5 | -2.53 | 4.72 | 2.93E-45 | 5.74E-44 |
| RP4-539M6.14 | -2.51 | 6.19 | 3.31E-45 | 6.45E-44 |
| RP11-211G23.2 | 5.88 | 6.13 | 3.38E-45 | 6.58E-44 |
| RP11-277P12.20 | 3.27 | 9.41 | 7.66E-45 | 1.47E-43 |
| AC005324.6 | -3.08 | 2.89 | 7.75E-45 | 1.48E-43 |
| LINC02156 | 3.31 | 3.72 | 9.58E-45 | 1.83E-43 |
| RP11-909N17.2 | 4.67 | 5.72 | 9.84E-45 | 1.87E-43 |
| RP11-445O3.2 | 6.75 | 5.48 | 1.08E-44 | 2.04E-43 |
| RP11-44F21.5 | 2.94 | 8.99 | 1.40E-44 | 2.64E-43 |
| RP11-739B23.1 | 2.42 | 4.27 | 1.42E-44 | 2.67E-43 |
| RP11-57A19.2 | 3.18 | 6.47 | 1.47E-44 | 2.76E-43 |
| AC004221.2 | 3.23 | 3.72 | 1.54E-44 | 2.90E-43 |
| RP3-323A16.1 | 3.48 | 7.89 | 2.74E-44 | 5.10E-43 |
| FGF10-AS1 | -2.82 | 2.89 | 2.81E-44 | 5.23E-43 |
| AC005256.1 | 6.17 | 4.59 | 3.42E-44 | 6.35E-43 |
| WASIR2 | 3.00 | 5.48 | 3.81E-44 | 7.05E-43 |
| LINC02159 | 3.83 | 7.41 | 4.70E-44 | 8.65E-43 |
| RP11-254F7.4 | 2.95 | 3.64 | 4.91E-44 | 9.02E-43 |
| CTD-2129N1.1 | 3.15 | 3.49 | 6.13E-44 | 1.12E-42 |
| RP11-366L20.2 | 2.84 | 6.31 | 7.46E-44 | 1.36E-42 |
| RP11-284F21.10 | 4.08 | 10.35 | 8.03E-44 | 1.45E-42 |
| BARX1-AS1 | 6.68 | 5.70 | 1.03E-43 | 1.85E-42 |
| AC002076.10 | 3.78 | 4.60 | 1.26E-43 | 2.26E-42 |
| RP11-254F19.2 | -2.41 | 2.85 | 1.79E-43 | 3.21E-42 |
| RP11-81H3.2 | 6.26 | 6.27 | 1.83E-43 | 3.26E-42 |
| RP11-673E11.2 | -2.73 | 2.82 | 1.88E-43 | 3.34E-42 |
| CTD-2591A6.2 | 6.84 | 5.11 | 2.27E-43 | 4.04E-42 |
| RP11-713C5.1 | 4.00 | 5.88 | 3.11E-43 | 5.52E-42 |
| RP11-123B3.2 | 4.61 | 4.83 | 3.31E-43 | 5.87E-42 |
| RP11-231D20.2 | 3.88 | 5.69 | 3.44E-43 | 6.09E-42 |
| RP11-445O3.1 | 5.65 | 4.16 | 3.47E-43 | 6.12E-42 |
| RP11-17A4.2 | -2.42 | 3.61 | 4.70E-43 | 8.26E-42 |
| KB-1440D3.13 | 2.57 | 4.66 | 5.34E-43 | 9.37E-42 |
| CTD-2021H9.3 | 5.87 | 7.52 | 5.48E-43 | 9.60E-42 |
| CCAT1 | 7.40 | 9.43 | 6.71E-43 | 1.17E-41 |
| DGCR9 | 2.65 | 7.13 | 6.79E-43 | 1.18E-41 |
| UMODL1-AS1 | -2.47 | 5.88 | 8.26E-43 | 1.43E-41 |
| LINC02003 | 3.86 | 3.89 | 8.93E-43 | 1.55E-41 |
| LINC00958 | 4.90 | 10.17 | 9.73E-43 | 1.68E-41 |
| LINC01607 | 2.20 | 6.33 | 1.06E-42 | 1.82E-41 |
| LINC01305 | 6.22 | 5.92 | 1.06E-42 | 1.83E-41 |
| RP11-284F21.9 | 4.78 | 8.81 | 1.26E-42 | 2.15E-41 |
| RP11-304L19.1 | 3.15 | 7.11 | 1.93E-42 | 3.29E-41 |
| RP11-676J12.6 | -2.26 | 3.76 | 1.98E-42 | 3.36E-41 |
| RP11-758M4.4 | 7.02 | 7.99 | 3.42E-42 | 5.77E-41 |
| HOXA11-AS | 5.30 | 6.34 | 3.70E-42 | 6.24E-41 |
| RP11-304L19.3 | 3.28 | 6.97 | 3.98E-42 | 6.67E-41 |
| RP5-823G15.5 | 3.52 | 5.05 | 4.31E-42 | 7.21E-41 |
| CTD-2256P15.1 | 2.58 | 3.38 | 4.38E-42 | 7.31E-41 |
| RP11-145A3.1 | 3.06 | 6.10 | 4.95E-42 | 8.23E-41 |
| RP11-1103G16.1 | 7.52 | 6.55 | 5.37E-42 | 8.90E-41 |
| SATB2-AS1 | 3.11 | 4.66 | 5.72E-42 | 9.47E-41 |
| CTD-2139B15.5 | 7.25 | 5.54 | 6.65E-42 | 1.10E-40 |
| RP11-30P6.6 | 3.87 | 5.69 | 7.27E-42 | 1.20E-40 |
| RP11-132A1.4 | 2.68 | 8.15 | 9.91E-42 | 1.62E-40 |
| LINC01234 | 6.66 | 8.58 | 1.23E-41 | 2.00E-40 |
| LINC01765 | -2.80 | 5.14 | 1.38E-41 | 2.24E-40 |
| LINC00337 | 2.68 | 6.01 | 1.41E-41 | 2.29E-40 |
| LINC00626 | 6.88 | 7.04 | 1.61E-41 | 2.60E-40 |
| AC114803.3 | 4.90 | 3.45 | 1.96E-41 | 3.13E-40 |
| RP11-102G14.1 | 2.29 | 4.98 | 2.13E-41 | 3.39E-40 |
| RP11-96H17.1 | 5.22 | 5.32 | 2.23E-41 | 3.54E-40 |
| LINC00862 | 3.15 | 5.59 | 2.50E-41 | 3.97E-40 |
| RP11-1C8.4 | 5.58 | 5.31 | 2.85E-41 | 4.51E-40 |
| RP11-1C8.7 | 5.59 | 4.82 | 3.69E-41 | 5.83E-40 |
| FAM83C-AS1 | 2.46 | 3.76 | 3.73E-41 | 5.88E-40 |
| RP11-734K21.2 | 3.43 | 4.86 | 5.84E-41 | 9.14E-40 |
| PKP4-AS1 | 2.67 | 7.42 | 6.52E-41 | 1.02E-39 |
| HOTAIR | 6.64 | 6.87 | 7.63E-41 | 1.19E-39 |
| AL163953.2 | 5.82 | 4.44 | 8.83E-41 | 1.37E-39 |
| RP11-734K21.5 | 3.60 | 6.52 | 9.65E-41 | 1.49E-39 |
| RP11-357H14.17 | 4.95 | 7.60 | 1.00E-40 | 1.54E-39 |
| RP11-616M22.7 | 5.30 | 4.98 | 1.02E-40 | 1.57E-39 |
| RP6-114E22.1 | 5.01 | 5.70 | 1.11E-40 | 1.70E-39 |
| LINC00898 | 6.58 | 5.71 | 1.46E-40 | 2.24E-39 |
| LINC01807 | 6.49 | 6.00 | 1.63E-40 | 2.48E-39 |
| LINC02043 | 3.86 | 4.95 | 1.74E-40 | 2.65E-39 |
| RP11-476D10.1 | -2.55 | 7.53 | 1.98E-40 | 3.00E-39 |
| LINC01827 | -2.36 | 3.78 | 2.46E-40 | 3.74E-39 |
| CTD-2228K2.7 | 2.30 | 10.63 | 2.57E-40 | 3.88E-39 |
| CTC-441N14.2 | -2.09 | 3.91 | 2.97E-40 | 4.48E-39 |
| NAALADL2-AS2 | 5.53 | 5.90 | 3.06E-40 | 4.61E-39 |
| AC078942.1 | -2.17 | 3.07 | 3.25E-40 | 4.89E-39 |
| RP4-735C1.4 | -2.25 | 3.25 | 3.71E-40 | 5.56E-39 |
| RP11-284F21.7 | 3.46 | 7.77 | 3.97E-40 | 5.93E-39 |
| RP11-204P2.3 | -2.02 | 2.65 | 6.33E-40 | 9.45E-39 |
| CTC-276P9.4 | 4.81 | 4.45 | 6.61E-40 | 9.84E-39 |
| RP11-417E7.2 | 3.00 | 5.92 | 7.11E-40 | 1.05E-38 |
| F11-AS1 | -2.35 | 5.29 | 7.44E-40 | 1.10E-38 |
| CTD-2292P10.4 | 2.22 | 6.47 | 8.99E-40 | 1.33E-38 |
| LINC00461 | 4.67 | 5.01 | 9.15E-40 | 1.35E-38 |
| RP5-1120P11.1 | 2.69 | 8.62 | 1.04E-39 | 1.53E-38 |
| LINC01468 | 6.82 | 6.47 | 1.19E-39 | 1.74E-38 |
| MIR2052HG | 4.39 | 5.68 | 1.46E-39 | 2.13E-38 |
| MIR205HG | 5.22 | 12.42 | 1.51E-39 | 2.20E-38 |
| MAFA-AS1 | 4.49 | 5.24 | 1.63E-39 | 2.36E-38 |
| AC069277.2 | 4.88 | 5.79 | 1.73E-39 | 2.51E-38 |
| LINC01559 | 6.16 | 8.34 | 1.82E-39 | 2.63E-38 |
| RP11-122M14.1 | -2.06 | 5.50 | 2.55E-39 | 3.67E-38 |
| RP11-397A16.1 | 6.63 | 6.63 | 2.89E-39 | 4.15E-38 |
| RP11-434D9.2 | 4.37 | 4.15 | 2.94E-39 | 4.21E-38 |
| RP11-245D16.4 | 2.14 | 6.17 | 3.32E-39 | 4.73E-38 |
| CTD-2555C10.3 | 3.07 | 6.27 | 4.71E-39 | 6.72E-38 |
| LDLRAD4-AS1 | -2.50 | 3.57 | 5.85E-39 | 8.31E-38 |
| LINC01625 | -2.08 | 3.30 | 7.27E-39 | 1.03E-37 |
| RP11-434H14.1 | -2.30 | 2.91 | 7.98E-39 | 1.13E-37 |
| RP3-340N1.2 | 5.19 | 9.15 | 8.06E-39 | 1.13E-37 |
| UCA1 | 5.26 | 9.05 | 9.85E-39 | 1.38E-37 |
| LINC01842 | 3.71 | 5.90 | 1.48E-38 | 2.06E-37 |
| LINC00858 | 4.83 | 6.47 | 1.50E-38 | 2.09E-37 |
| RP11-432I5.2 | 2.65 | 3.77 | 1.55E-38 | 2.15E-37 |
| AC010148.1 | 2.39 | 5.86 | 1.62E-38 | 2.24E-37 |
| MGAT3-AS1 | -2.25 | 3.74 | 2.01E-38 | 2.78E-37 |
| AC011738.4 | 3.81 | 4.98 | 2.16E-38 | 2.98E-37 |
| RP11-180I4.4 | -2.50 | 3.26 | 2.37E-38 | 3.27E-37 |
| LINC01564 | 3.64 | 7.07 | 2.51E-38 | 3.46E-37 |
| AC009264.1 | 6.59 | 7.14 | 2.62E-38 | 3.61E-37 |
| RP11-90L1.8 | 2.53 | 7.02 | 3.26E-38 | 4.48E-37 |
| LINC01967 | 5.52 | 4.55 | 3.31E-38 | 4.54E-37 |
| AC108676.1 | 4.19 | 7.61 | 4.96E-38 | 6.76E-37 |
| RP11-519M16.1 | 5.95 | 4.60 | 5.22E-38 | 7.10E-37 |
| CTD-2340E1.2 | 4.85 | 3.91 | 5.36E-38 | 7.27E-37 |
| LINC01166 | -2.66 | 3.42 | 5.98E-38 | 8.11E-37 |
| PGM5P3-AS1 | -2.22 | 3.95 | 6.02E-38 | 8.15E-37 |
| RP11-108K3.3 | 3.87 | 2.87 | 7.03E-38 | 9.50E-37 |
| RP11-21L23.2 | 2.29 | 8.77 | 7.69E-38 | 1.04E-36 |
| RP11-95M15.1 | 4.91 | 5.42 | 7.93E-38 | 1.07E-36 |
| U47924.29 | 2.25 | 3.73 | 7.98E-38 | 1.07E-36 |
| RP11-380J14.1 | 5.19 | 5.84 | 8.23E-38 | 1.11E-36 |
| CTD-2330J20.2 | -2.41 | 2.63 | 1.19E-37 | 1.59E-36 |
| RP11-10A14.5 | 4.06 | 6.70 | 1.23E-37 | 1.63E-36 |
| LINC01096 | 4.80 | 4.96 | 1.23E-37 | 1.64E-36 |
| RP11-445O3.3 | 5.85 | 5.61 | 1.43E-37 | 1.89E-36 |
| RP11-159F24.6 | 3.40 | 6.23 | 1.52E-37 | 2.01E-36 |
| RP4-616B8.5 | 2.23 | 5.03 | 1.53E-37 | 2.02E-36 |
| RP11-671P2.1 | -2.16 | 3.43 | 1.93E-37 | 2.55E-36 |
| C5orf66-AS1 | 6.77 | 6.56 | 2.53E-37 | 3.30E-36 |
| RP11-314A20.2 | 2.13 | 4.62 | 2.57E-37 | 3.35E-36 |
| RMST | -2.57 | 5.16 | 2.84E-37 | 3.70E-36 |
| CTC-321K16.1 | 4.26 | 4.74 | 3.01E-37 | 3.92E-36 |
| RP11-524H19.2 | 6.04 | 6.49 | 4.03E-37 | 5.22E-36 |
| RP11-661A12.9 | 2.55 | 5.23 | 4.59E-37 | 5.91E-36 |
| CTD-2619J13.13 | 2.65 | 7.05 | 4.74E-37 | 6.09E-36 |
| RP11-368L12.1 | 4.85 | 5.26 | 5.18E-37 | 6.64E-36 |
| LINC00629 | 2.74 | 4.27 | 5.92E-37 | 7.57E-36 |
| LINC01968 | 3.09 | 3.75 | 6.07E-37 | 7.75E-36 |
| LINC00668 | 6.35 | 9.01 | 6.12E-37 | 7.80E-36 |
| HOXA10-AS | 5.13 | 5.03 | 6.19E-37 | 7.89E-36 |
| LINC01270 | 2.15 | 7.81 | 6.30E-37 | 8.01E-36 |
| RP11-124O11.1 | -2.26 | 3.13 | 6.71E-37 | 8.51E-36 |
| LL22NC03-N64E9.1 | 3.90 | 4.02 | 6.77E-37 | 8.57E-36 |
| AC123023.1 | -2.54 | 3.67 | 7.36E-37 | 9.30E-36 |
| LINC01168 | -2.51 | 3.04 | 9.88E-37 | 1.24E-35 |
| RP11-169F17.1 | 6.82 | 9.23 | 1.08E-36 | 1.35E-35 |
| RP11-492E3.2 | 3.45 | 7.71 | 1.09E-36 | 1.36E-35 |
| RP11-895M11.3 | 2.30 | 3.59 | 1.12E-36 | 1.40E-35 |
| HOXC-AS3 | 6.06 | 5.53 | 1.20E-36 | 1.49E-35 |
| RP5-940J5.3 | 2.02 | 4.29 | 1.33E-36 | 1.65E-35 |
| C10orf91 | 2.69 | 6.94 | 1.49E-36 | 1.85E-35 |
| RP11-3B12.5 | 5.33 | 5.47 | 1.54E-36 | 1.90E-35 |
| AC068831.16 | 3.74 | 4.58 | 1.78E-36 | 2.20E-35 |
| AC011288.2 | 5.85 | 6.03 | 1.79E-36 | 2.21E-35 |
| CTD-3035D6.2 | 2.70 | 5.01 | 1.86E-36 | 2.28E-35 |
| LINC01561 | 3.51 | 4.83 | 1.86E-36 | 2.28E-35 |
| MIR9-3HG | 3.04 | 9.26 | 2.28E-36 | 2.80E-35 |
| RP11-80H5.2 | 3.49 | 3.36 | 2.71E-36 | 3.30E-35 |
| LINC00665 | 2.22 | 11.21 | 2.75E-36 | 3.35E-35 |
| RP11-353N14.1 | 3.93 | 3.46 | 2.82E-36 | 3.42E-35 |
| LINC01518 | 7.31 | 5.62 | 2.93E-36 | 3.55E-35 |
| LINC02081 | 2.62 | 6.98 | 2.98E-36 | 3.61E-35 |
| STEAP2-AS1 | 2.86 | 3.81 | 3.01E-36 | 3.63E-35 |
| CTD-3080P12.3 | -2.91 | 4.28 | 3.13E-36 | 3.77E-35 |
| LINC01873 | 4.18 | 4.02 | 3.85E-36 | 4.64E-35 |
| DEPDC1-AS1 | 2.98 | 3.58 | 4.42E-36 | 5.32E-35 |
| AE000662.93 | 2.84 | 3.86 | 4.43E-36 | 5.33E-35 |
| AC006262.6 | 3.18 | 6.64 | 4.87E-36 | 5.85E-35 |
| LINC01206 | 9.03 | 10.76 | 5.55E-36 | 6.65E-35 |
| SOX21-AS1 | 3.49 | 9.28 | 5.61E-36 | 6.72E-35 |
| HOXC-AS1 | 3.66 | 5.28 | 5.93E-36 | 7.09E-35 |
| RP11-54O7.18 | 3.16 | 5.04 | 8.30E-36 | 9.88E-35 |
| PTGES2-AS1 | 2.17 | 4.56 | 8.60E-36 | 1.02E-34 |
| AC012501.2 | 5.65 | 4.16 | 9.95E-36 | 1.18E-34 |
| RP11-290F5.1 | 2.17 | 7.98 | 1.22E-35 | 1.44E-34 |
| DSG1-AS1 | 6.39 | 5.90 | 1.59E-35 | 1.87E-34 |
| RP11-63N8.3 | -2.07 | 2.58 | 1.74E-35 | 2.04E-34 |
| LL22NC03-63E9.3 | 4.13 | 4.30 | 1.97E-35 | 2.31E-34 |
| LINC01605 | 3.80 | 7.93 | 2.84E-35 | 3.32E-34 |
| RP11-573D15.8 | 2.61 | 5.04 | 3.11E-35 | 3.63E-34 |
| RP11-84D1.1 | 2.15 | 4.56 | 3.74E-35 | 4.34E-34 |
| HHATL-AS1 | -2.45 | 3.80 | 3.74E-35 | 4.34E-34 |
| RP11-1038A11.3 | 4.14 | 7.74 | 4.23E-35 | 4.88E-34 |
| CASC19 | 5.96 | 6.41 | 5.66E-35 | 6.51E-34 |
| CTD-2147F2.1 | 6.02 | 7.09 | 6.07E-35 | 6.97E-34 |
| SOX2-OT | 4.40 | 10.29 | 6.20E-35 | 7.11E-34 |
| SRGAP3-AS2 | -2.74 | 7.96 | 7.61E-35 | 8.67E-34 |
| RP11-54O7.1 | 3.13 | 5.44 | 7.80E-35 | 8.88E-34 |
| LINC01271 | 2.04 | 5.62 | 9.37E-35 | 1.06E-33 |
| CTC-441N14.1 | -2.09 | 4.17 | 1.01E-34 | 1.15E-33 |
| LINC01971 | 3.24 | 3.78 | 1.41E-34 | 1.60E-33 |
| RP11-525G13.2 | 2.06 | 5.42 | 1.54E-34 | 1.74E-33 |
| RP11-1007I13.4 | 4.51 | 4.19 | 1.57E-34 | 1.77E-33 |
| RP11-499O7.7 | 3.86 | 6.50 | 2.07E-34 | 2.32E-33 |
| CTD-2008P7.8 | 6.48 | 5.60 | 2.49E-34 | 2.79E-33 |
| LINC00941 | 3.55 | 7.66 | 2.57E-34 | 2.87E-33 |
| AC011294.3 | 3.82 | 6.50 | 3.54E-34 | 3.95E-33 |
| LINC01615 | 3.10 | 6.58 | 3.58E-34 | 3.99E-33 |
| RP11-218E20.3 | 3.99 | 5.83 | 3.76E-34 | 4.18E-33 |
| LINC00466 | 4.30 | 3.64 | 4.03E-34 | 4.48E-33 |
| RP11-408B11.2 | 5.82 | 6.21 | 4.61E-34 | 5.10E-33 |
| RP11-802D6.1 | 2.25 | 4.54 | 5.89E-34 | 6.49E-33 |
| LINC01616 | -2.91 | 3.13 | 7.13E-34 | 7.83E-33 |
| RP11-646E18.4 | 5.93 | 5.33 | 7.17E-34 | 7.86E-33 |
| RP11-863P13.3 | 2.52 | 5.85 | 7.85E-34 | 8.58E-33 |
| RP11-429J17.5 | 2.81 | 4.46 | 8.67E-34 | 9.45E-33 |
| LINC01385 | 5.47 | 4.04 | 9.30E-34 | 1.01E-32 |
| LINC01876 | 2.22 | 7.01 | 9.36E-34 | 1.02E-32 |
| RP11-417L19.2 | 3.86 | 3.90 | 1.04E-33 | 1.12E-32 |
| RP11-268F1.3 | -2.63 | 4.58 | 1.04E-33 | 1.13E-32 |
| RP11-32K4.1 | 6.29 | 5.09 | 1.34E-33 | 1.45E-32 |
| RP11-411K7.1 | 4.05 | 7.69 | 1.55E-33 | 1.66E-32 |
| LINC01611 | 5.97 | 4.60 | 1.75E-33 | 1.87E-32 |
| LINC01348 | 2.25 | 7.53 | 1.81E-33 | 1.93E-32 |
| RP11-1038A11.1 | 3.83 | 5.60 | 3.17E-33 | 3.36E-32 |
| RP5-965G21.4 | 2.05 | 6.77 | 3.35E-33 | 3.54E-32 |
| RP11-1069G10.2 | 3.81 | 4.02 | 3.44E-33 | 3.64E-32 |
| RP11-356K23.2 | -2.23 | 3.35 | 4.13E-33 | 4.34E-32 |
| LA16c-321D4.2 | 2.42 | 5.31 | 6.14E-33 | 6.44E-32 |
| AC105053.3 | -2.07 | 4.33 | 6.27E-33 | 6.56E-32 |
| RP11-65D17.1 | 3.86 | 4.68 | 6.40E-33 | 6.70E-32 |
| RP1-272L16.1 | 5.74 | 5.03 | 6.82E-33 | 7.12E-32 |
| AC012531.25 | 3.50 | 5.08 | 8.67E-33 | 9.02E-32 |
| RP11-332J15.4 | 2.96 | 4.31 | 8.72E-33 | 9.07E-32 |
| LINC01981 | 4.58 | 3.27 | 8.89E-33 | 9.23E-32 |
| AC114812.8 | 4.97 | 4.40 | 9.00E-33 | 9.33E-32 |
| LINC01775 | 2.36 | 3.59 | 1.09E-32 | 1.13E-31 |
| RP11-274B18.4 | -2.27 | 2.55 | 1.11E-32 | 1.15E-31 |
| RP11-483F11.7 | 2.24 | 4.91 | 1.34E-32 | 1.38E-31 |
| RP11-395G23.3 | 2.03 | 8.18 | 1.37E-32 | 1.41E-31 |
| RP11-10A14.4 | 2.30 | 5.82 | 1.65E-32 | 1.69E-31 |
| RP11-881M11.4 | 2.82 | 4.18 | 1.69E-32 | 1.73E-31 |
| CTD-2066L21.3 | 4.79 | 5.06 | 1.76E-32 | 1.80E-31 |
| CTA-384D8.31 | 3.86 | 7.11 | 2.18E-32 | 2.22E-31 |
| LINC01393 | 2.11 | 5.31 | 2.56E-32 | 2.60E-31 |
| LINC02012 | 2.49 | 6.55 | 2.60E-32 | 2.63E-31 |
| RP11-397A16.3 | 4.77 | 3.27 | 2.85E-32 | 2.88E-31 |
| AC053503.4 | 2.22 | 4.49 | 3.72E-32 | 3.75E-31 |
| LINC02086 | 3.49 | 6.82 | 4.16E-32 | 4.17E-31 |
| RP11-66B24.7 | 2.66 | 6.99 | 4.64E-32 | 4.65E-31 |
| AC008268.1 | -2.99 | 9.02 | 5.73E-32 | 5.72E-31 |
| KB-1615E4.2 | 3.31 | 4.04 | 5.78E-32 | 5.76E-31 |
| RP1-27K12.2 | 7.01 | 10.81 | 6.50E-32 | 6.47E-31 |
| RP11-542G1.1 | 5.20 | 4.60 | 7.32E-32 | 7.27E-31 |
| RP11-546O6.4 | 3.83 | 3.87 | 8.25E-32 | 8.18E-31 |
| RP11-1134I14.8 | 2.09 | 6.75 | 8.33E-32 | 8.25E-31 |
| LINC00525 | 2.73 | 5.35 | 8.83E-32 | 8.74E-31 |
| RP11-150C16.1 | 2.83 | 5.37 | 9.17E-32 | 9.07E-31 |
| CTD-2194D22.3 | 6.67 | 4.90 | 9.53E-32 | 9.41E-31 |
| ARNTL2-AS1 | 3.35 | 3.56 | 1.04E-31 | 1.03E-30 |
| RP5-984P4.6 | 6.02 | 5.06 | 1.05E-31 | 1.03E-30 |
| MIR2117HG | 4.58 | 5.18 | 1.11E-31 | 1.09E-30 |
| RP11-44N12.5 | 2.65 | 4.64 | 1.32E-31 | 1.29E-30 |
| RP11-356K23.1 | -2.21 | 6.87 | 1.33E-31 | 1.30E-30 |
| LINC02109 | 5.33 | 5.16 | 1.39E-31 | 1.36E-30 |
| LINP1 | 4.25 | 7.09 | 2.32E-31 | 2.25E-30 |
| CTB-186G2.1 | 4.09 | 5.22 | 2.42E-31 | 2.34E-30 |
| RP11-391H12.8 | 2.37 | 4.25 | 2.75E-31 | 2.66E-30 |
| AC093390.1 | -2.25 | 3.07 | 3.34E-31 | 3.23E-30 |
| LINC01513 | -2.18 | 4.11 | 3.81E-31 | 3.67E-30 |
| RP11-310P5.1 | 3.10 | 3.07 | 3.85E-31 | 3.71E-30 |
| RP11-13E5.2 | 4.52 | 3.27 | 3.97E-31 | 3.81E-30 |
| RP11-114H23.1 | 3.35 | 5.59 | 4.07E-31 | 3.91E-30 |
| AP000696.2 | 4.48 | 3.82 | 4.85E-31 | 4.64E-30 |
| LINC01395 | 4.03 | 4.61 | 5.18E-31 | 4.94E-30 |
| RP11-519G16.5 | 3.20 | 7.87 | 6.50E-31 | 6.17E-30 |
| RP11-286H14.8 | 2.67 | 4.51 | 6.59E-31 | 6.24E-30 |
| LINC01524 | 4.17 | 4.31 | 6.69E-31 | 6.33E-30 |
| RP3-340B19.3 | -2.10 | 5.73 | 7.93E-31 | 7.48E-30 |
| RP11-133K1.11 | 2.95 | 3.18 | 9.10E-31 | 8.55E-30 |
| ABCA9-AS1 | 4.09 | 5.76 | 9.29E-31 | 8.71E-30 |
| LINC02187 | 5.16 | 5.07 | 1.01E-30 | 9.43E-30 |
| LINC00393 | 6.79 | 5.73 | 1.10E-30 | 1.03E-29 |
| SALRNA1 | 2.47 | 4.98 | 1.10E-30 | 1.03E-29 |
| SH3PXD2A-AS1 | 3.19 | 8.23 | 1.16E-30 | 1.08E-29 |
| RP13-455A7.1 | 4.02 | 3.02 | 1.19E-30 | 1.10E-29 |
| LINC01249 | 7.38 | 6.26 | 1.30E-30 | 1.21E-29 |
| C8orf34-AS1 | -2.02 | 9.09 | 1.39E-30 | 1.29E-29 |
| IGFL2-AS1 | 5.25 | 8.36 | 1.56E-30 | 1.44E-29 |
| RP11-146I2.1 | 2.49 | 4.18 | 1.58E-30 | 1.45E-29 |
| RP11-1055B8.9 | 2.31 | 4.44 | 1.62E-30 | 1.49E-29 |
| RP11-108M12.3 | 2.86 | 5.18 | 1.84E-30 | 1.69E-29 |
| RP11-677M24.1 | 4.63 | 3.52 | 1.86E-30 | 1.71E-29 |
| CTD-2529O21.1 | 2.64 | 3.48 | 1.98E-30 | 1.81E-29 |
| CTC-327F10.5 | 3.59 | 3.32 | 2.00E-30 | 1.84E-29 |
| CTB-113P19.4 | 4.58 | 7.64 | 2.24E-30 | 2.06E-29 |
| RP11-96B2.1 | 4.26 | 4.31 | 2.68E-30 | 2.45E-29 |
| RP11-1M18.1 | 2.63 | 4.32 | 2.93E-30 | 2.68E-29 |
| RP11-193M21.1 | -2.06 | 5.19 | 2.95E-30 | 2.69E-29 |
| RP4-669H2.1 | 2.61 | 5.38 | 3.39E-30 | 3.07E-29 |
| RP11-567N4.3 | 5.40 | 4.49 | 3.62E-30 | 3.28E-29 |
| RP5-827C21.6 | 2.45 | 5.04 | 3.83E-30 | 3.46E-29 |
| RP11-159F24.5 | 2.61 | 5.35 | 3.91E-30 | 3.52E-29 |
| RP11-424M24.5 | -2.53 | 4.69 | 4.05E-30 | 3.64E-29 |
| AC112721.2 | 2.81 | 4.49 | 4.13E-30 | 3.71E-29 |
| RP11-124N19.3 | 3.21 | 4.64 | 5.35E-30 | 4.79E-29 |
| AC104088.1 | 4.13 | 4.91 | 5.45E-30 | 4.87E-29 |
| RP4-760C5.3 | 2.05 | 4.22 | 5.55E-30 | 4.96E-29 |
| AC018890.6 | 3.64 | 6.99 | 5.80E-30 | 5.17E-29 |
| MIR137HG | 5.66 | 5.46 | 6.41E-30 | 5.71E-29 |
| APCDD1L-AS1 | 3.49 | 6.45 | 6.46E-30 | 5.75E-29 |
| LINC01451 | 3.34 | 7.87 | 7.03E-30 | 6.25E-29 |
| RP5-1158E12.3 | 2.55 | 4.98 | 7.99E-30 | 7.08E-29 |
| LINC00592 | 2.52 | 5.28 | 8.56E-30 | 7.57E-29 |
| ELFN1-AS1 | 3.80 | 6.46 | 8.87E-30 | 7.84E-29 |
| FLJ12825 | 2.02 | 5.18 | 9.20E-30 | 8.12E-29 |
| RP11-78F17.1 | 3.86 | 4.63 | 9.80E-30 | 8.63E-29 |
| RP11-553A10.1 | 3.54 | 7.22 | 1.18E-29 | 1.04E-28 |
| CTC-537E7.2 | 6.27 | 5.47 | 1.25E-29 | 1.09E-28 |
| RP11-818F20.5 | 3.78 | 5.96 | 1.30E-29 | 1.14E-28 |
| RP11-96H17.3 | 4.06 | 3.68 | 1.34E-29 | 1.17E-28 |
| LINC01698 | 5.60 | 3.92 | 1.57E-29 | 1.37E-28 |
| LL22NC03-N14H11.1 | 2.29 | 5.03 | 1.64E-29 | 1.42E-28 |
| LINC00355 | 5.61 | 6.21 | 1.66E-29 | 1.45E-28 |
| RP11-527N22.2 | 4.02 | 5.70 | 1.67E-29 | 1.45E-28 |
| RP5-968D22.3 | 2.98 | 3.24 | 1.67E-29 | 1.45E-28 |
| LSAMP-AS1 | 4.29 | 4.85 | 1.70E-29 | 1.48E-28 |
| RP11-215P8.4 | 5.07 | 5.52 | 1.92E-29 | 1.65E-28 |
| NOVA1-AS1 | 5.57 | 5.85 | 2.04E-29 | 1.76E-28 |
| CTD-2008L17.2 | 4.10 | 7.19 | 2.23E-29 | 1.92E-28 |
| RP11-230B22.1 | 2.01 | 4.26 | 2.47E-29 | 2.12E-28 |
| RP11-187E13.1 | 4.03 | 4.44 | 2.52E-29 | 2.16E-28 |
| RP1-251M9.2 | -2.23 | 2.64 | 2.65E-29 | 2.27E-28 |
| CH507-42P11.6 | 3.02 | 3.58 | 3.33E-29 | 2.82E-28 |
| RP4-660H19.1 | 4.96 | 5.79 | 3.38E-29 | 2.86E-28 |
| LINC00501 | 4.91 | 5.46 | 3.54E-29 | 2.99E-28 |
| KC6 | 4.10 | 7.89 | 3.68E-29 | 3.11E-28 |
| DLX2-AS1 | 4.65 | 3.39 | 4.17E-29 | 3.51E-28 |
| AC011298.2 | 6.40 | 6.10 | 4.59E-29 | 3.85E-28 |
| CTD-2184D3.3 | 5.43 | 4.47 | 5.30E-29 | 4.44E-28 |
| CTC-239J10.1 | -2.01 | 2.51 | 6.98E-29 | 5.82E-28 |
| CTD-2337I7.1 | 3.46 | 4.89 | 8.49E-29 | 7.05E-28 |
| RP11-120K18.2 | 2.51 | 4.46 | 8.51E-29 | 7.07E-28 |
| CTA-246H3.12 | 3.27 | 3.36 | 9.05E-29 | 7.49E-28 |
| RP11-123K3.9 | 2.38 | 3.25 | 9.31E-29 | 7.70E-28 |
| RP11-286B14.2 | 5.19 | 3.86 | 1.04E-28 | 8.59E-28 |
| LINC00536 | 5.13 | 4.10 | 1.34E-28 | 1.11E-27 |
| RP11-429B14.4 | 4.41 | 3.53 | 1.42E-28 | 1.17E-27 |
| RP11-635N19.3 | 4.66 | 3.86 | 1.62E-28 | 1.33E-27 |
| RP11-190J1.3 | 5.19 | 4.63 | 1.80E-28 | 1.47E-27 |
| RP11-138H10.2 | -2.09 | 2.63 | 1.89E-28 | 1.54E-27 |
| RP11-366F6.2 | 7.73 | 6.84 | 1.94E-28 | 1.58E-27 |
| LINC01214 | 5.29 | 4.76 | 1.96E-28 | 1.60E-27 |
| RP11-66D17.3 | 2.73 | 3.24 | 2.08E-28 | 1.69E-27 |
| LINC02178 | 6.15 | 5.06 | 2.36E-28 | 1.91E-27 |
| RP11-38M8.1 | 2.28 | 6.21 | 2.72E-28 | 2.19E-27 |
| FAM222A-AS1 | 2.56 | 5.36 | 2.74E-28 | 2.21E-27 |
| RP11-114G22.1 | 4.95 | 5.36 | 2.96E-28 | 2.39E-27 |
| LINC00707 | 3.81 | 7.07 | 3.04E-28 | 2.45E-27 |
| TM4SF1-AS1 | 2.28 | 6.71 | 3.10E-28 | 2.49E-27 |
| CTD-2083E4.7 | 3.77 | 4.11 | 3.49E-28 | 2.80E-27 |
| HOTTIP | 4.79 | 4.28 | 3.66E-28 | 2.94E-27 |
| RP11-104H15.10 | 2.43 | 3.12 | 4.17E-28 | 3.32E-27 |
| RP11-390N6.1 | 3.08 | 3.65 | 4.38E-28 | 3.49E-27 |
| RP11-462L8.1 | 3.13 | 5.80 | 5.51E-28 | 4.37E-27 |
| AL109761.5 | 2.48 | 4.47 | 5.88E-28 | 4.66E-27 |
| RP11-495P10.5 | 3.31 | 3.56 | 5.93E-28 | 4.69E-27 |
| AC114765.1 | 3.17 | 3.03 | 6.31E-28 | 4.98E-27 |
| AC003958.2 | 5.29 | 4.84 | 6.75E-28 | 5.32E-27 |
| LINC01297 | 5.24 | 3.99 | 6.98E-28 | 5.50E-27 |
| CTC-575I10.1 | 3.11 | 3.52 | 7.47E-28 | 5.87E-27 |
| CTD-2196E14.5 | 2.14 | 3.18 | 8.41E-28 | 6.61E-27 |
| LINC01287 | 6.21 | 7.96 | 8.60E-28 | 6.75E-27 |
| LINC01447 | -2.21 | 3.98 | 9.97E-28 | 7.80E-27 |
| RP4-616B8.6 | 2.56 | 3.28 | 1.13E-27 | 8.85E-27 |
| RP11-308D13.3 | 4.31 | 4.08 | 1.24E-27 | 9.64E-27 |
| LINC00880 | 2.70 | 5.39 | 1.42E-27 | 1.10E-26 |
| RP11-561P12.5 | -2.29 | 2.59 | 1.43E-27 | 1.11E-26 |
| RP11-254F7.1 | 5.33 | 4.29 | 1.63E-27 | 1.26E-26 |
| LINC01456 | 5.81 | 5.04 | 1.64E-27 | 1.27E-26 |
| RP11-167H9.4 | 4.46 | 4.46 | 2.34E-27 | 1.80E-26 |
| RP11-644C3.1 | 3.23 | 3.09 | 2.36E-27 | 1.82E-26 |
| MIR31HG | 3.91 | 6.14 | 2.37E-27 | 1.83E-26 |
| HOXB-AS4 | 4.64 | 4.82 | 2.40E-27 | 1.85E-26 |
| LINC00460 | 3.82 | 7.83 | 2.49E-27 | 1.92E-26 |
| AP000697.6 | 3.75 | 2.83 | 2.62E-27 | 2.01E-26 |
| CTD-2314B22.1 | 5.49 | 3.75 | 2.74E-27 | 2.10E-26 |
| CTB-50L17.5 | 2.41 | 3.19 | 2.97E-27 | 2.27E-26 |
| RP11-335K5.2 | 3.35 | 3.04 | 3.56E-27 | 2.71E-26 |
| RP4-536B24.4 | 2.69 | 3.34 | 3.57E-27 | 2.71E-26 |
| CTD-2309O5.3 | 4.12 | 3.51 | 3.68E-27 | 2.80E-26 |
| RP11-104E19.1 | 4.71 | 5.82 | 3.79E-27 | 2.88E-26 |
| RP11-54O7.16 | 2.61 | 3.93 | 3.81E-27 | 2.89E-26 |
| AC092484.1 | 5.13 | 5.01 | 4.09E-27 | 3.10E-26 |
| RP11-568J23.8 | 2.45 | 5.04 | 4.58E-27 | 3.46E-26 |
| AC005330.2 | 2.59 | 5.09 | 5.43E-27 | 4.09E-26 |
| RP11-25L3.3 | 3.60 | 3.77 | 5.81E-27 | 4.37E-26 |
| LINC01979 | 2.43 | 5.30 | 6.09E-27 | 4.57E-26 |
| LINC00628 | 3.16 | 3.63 | 6.21E-27 | 4.65E-26 |
| RP11-829H16.3 | 2.49 | 4.71 | 7.17E-27 | 5.35E-26 |
| RP11-277P12.9 | 2.84 | 4.35 | 8.37E-27 | 6.22E-26 |
| CLDN10-AS1 | 5.64 | 6.58 | 8.66E-27 | 6.42E-26 |
| RP11-13P5.2 | 2.01 | 4.93 | 9.28E-27 | 6.86E-26 |
| RP11-680H20.2 | 3.73 | 5.68 | 9.46E-27 | 6.99E-26 |
| SMCR2 | 2.08 | 3.21 | 1.01E-26 | 7.42E-26 |
| RP4-781K5.5 | 4.87 | 3.81 | 1.02E-26 | 7.50E-26 |
| RP11-1012E15.2 | 3.64 | 5.19 | 1.07E-26 | 7.88E-26 |
| RP11-360L9.7 | 2.72 | 2.94 | 1.20E-26 | 8.79E-26 |
| FSIP2-AS1 | 2.15 | 4.50 | 1.33E-26 | 9.72E-26 |
| RP11-771K4.1 | 3.24 | 4.59 | 1.47E-26 | 1.08E-25 |
| LHFPL3-AS1 | -2.10 | 4.07 | 1.51E-26 | 1.10E-25 |
| RP11-360O19.4 | 2.32 | 4.47 | 1.53E-26 | 1.12E-25 |
| RP4-785G19.5 | 2.01 | 4.40 | 1.65E-26 | 1.20E-25 |
| LINC00518 | 4.80 | 3.93 | 1.65E-26 | 1.20E-25 |
| RP11-486O13.2 | -2.14 | 2.63 | 1.77E-26 | 1.29E-25 |
| AF127936.3 | 2.19 | 4.80 | 1.87E-26 | 1.35E-25 |
| RP11-337N6.1 | 4.30 | 6.58 | 1.89E-26 | 1.37E-25 |
| RP11-481J2.3 | 2.31 | 4.58 | 2.09E-26 | 1.51E-25 |
| MUC2 | 5.81 | 9.22 | 2.29E-26 | 1.65E-25 |
| CTD-2066L21.2 | 4.79 | 3.65 | 2.34E-26 | 1.69E-25 |
| KCNQ5-IT1 | 3.74 | 3.56 | 2.48E-26 | 1.79E-25 |
| RP11-6N13.1 | 4.60 | 3.22 | 2.80E-26 | 2.01E-25 |
| AC091801.1 | 4.94 | 4.28 | 3.18E-26 | 2.28E-25 |
| CTD-2566J3.1 | 5.68 | 6.04 | 4.46E-26 | 3.17E-25 |
| LINC01116 | 2.46 | 8.40 | 4.47E-26 | 3.18E-25 |
| AC024592.9 | 2.24 | 5.14 | 4.54E-26 | 3.22E-25 |
| RP11-499F3.2 | 3.29 | 5.63 | 4.55E-26 | 3.22E-25 |
| RP11-785D18.3 | 4.27 | 7.27 | 4.89E-26 | 3.46E-25 |
| LINC01202 | 5.34 | 3.73 | 5.31E-26 | 3.75E-25 |
| RP11-197K6.1 | 7.10 | 6.96 | 6.60E-26 | 4.65E-25 |
| RP11-10A14.9 | 2.43 | 3.90 | 7.53E-26 | 5.30E-25 |
| RP11-493L12.3 | 3.03 | 4.44 | 7.61E-26 | 5.35E-25 |
| RP4-784A16.3 | 3.02 | 2.88 | 7.76E-26 | 5.45E-25 |
| LINC01587 | 2.92 | 4.80 | 7.81E-26 | 5.48E-25 |
| RP11-874J12.4 | 3.45 | 5.59 | 1.02E-25 | 7.11E-25 |
| RP11-54H7.4 | 4.54 | 10.21 | 1.06E-25 | 7.40E-25 |
| LINC00165 | -2.50 | 3.89 | 1.09E-25 | 7.55E-25 |
| AC073316.2 | 2.97 | 4.47 | 1.20E-25 | 8.30E-25 |
| LINC01629 | 4.13 | 5.60 | 1.25E-25 | 8.69E-25 |
| AC024560.2 | 2.71 | 4.71 | 1.26E-25 | 8.70E-25 |
| MIR548XHG | 7.19 | 5.84 | 1.31E-25 | 9.04E-25 |
| LINC00615 | 5.77 | 4.36 | 1.37E-25 | 9.46E-25 |
| RP11-608O21.1 | 4.83 | 4.21 | 1.41E-25 | 9.74E-25 |
| RP11-319E16.1 | 3.33 | 3.10 | 1.53E-25 | 1.05E-24 |
| LINC01194 | 6.94 | 6.40 | 1.72E-25 | 1.18E-24 |
| AF003625.3 | 6.77 | 5.74 | 2.06E-25 | 1.41E-24 |
| RP11-47I22.2 | 2.02 | 5.97 | 2.07E-25 | 1.42E-24 |
| RP11-309M7.1 | 5.30 | 4.20 | 2.33E-25 | 1.59E-24 |
| RP11-177F15.1 | 4.21 | 4.36 | 2.36E-25 | 1.61E-24 |
| AP003900.6 | 5.99 | 4.26 | 2.61E-25 | 1.78E-24 |
| CTD-2544H17.1 | -2.45 | 4.44 | 2.67E-25 | 1.81E-24 |
| RP4-753D10.3 | -2.36 | 2.66 | 2.79E-25 | 1.90E-24 |
| RP11-434I12.3 | 3.99 | 6.88 | 2.95E-25 | 2.00E-24 |
| GDNF-AS1 | 3.25 | 5.82 | 2.99E-25 | 2.02E-24 |
| LINC00871 | 4.84 | 5.23 | 3.17E-25 | 2.14E-24 |
| RP11-276H19.2 | 3.35 | 8.66 | 3.21E-25 | 2.17E-24 |
| LINC01811 | -2.29 | 3.12 | 3.57E-25 | 2.41E-24 |
| GACAT2 | 3.28 | 3.81 | 3.86E-25 | 2.60E-24 |
| RP11-395E19.6 | -2.50 | 3.69 | 4.03E-25 | 2.71E-24 |
| LINC01993 | 2.04 | 5.41 | 4.61E-25 | 3.10E-24 |
| CTD-2034I4.2 | 2.50 | 3.93 | 4.79E-25 | 3.22E-24 |
| LINC01583 | 2.76 | 4.11 | 4.86E-25 | 3.26E-24 |
| RP5-827C21.2 | 2.30 | 4.54 | 5.12E-25 | 3.43E-24 |
| RP11-143E21.3 | 6.05 | 6.11 | 5.17E-25 | 3.45E-24 |
| LINC00392 | 8.70 | 6.97 | 5.70E-25 | 3.80E-24 |
| RP11-367F23.2 | 4.65 | 4.29 | 5.73E-25 | 3.82E-24 |
| CTD-2162K18.4 | 2.81 | 5.78 | 5.81E-25 | 3.88E-24 |
| LINC01429 | 3.07 | 3.22 | 6.14E-25 | 4.09E-24 |
| RP11-46A10.2 | 2.36 | 4.02 | 6.20E-25 | 4.13E-24 |
| LINC02076 | 2.35 | 4.00 | 6.47E-25 | 4.30E-24 |
| AC007249.3 | 2.25 | 5.50 | 6.71E-25 | 4.45E-24 |
| RP11-351I24.1 | 2.13 | 4.81 | 7.64E-25 | 5.06E-24 |
| RP11-685G9.4 | 3.19 | 2.86 | 7.67E-25 | 5.08E-24 |
| IL20RB-AS1 | 3.86 | 3.44 | 7.75E-25 | 5.13E-24 |
| RP11-384O8.1 | 2.05 | 7.16 | 9.08E-25 | 5.99E-24 |
| RP11-408E5.5 | 5.29 | 4.19 | 9.25E-25 | 6.10E-24 |
| RP11-367G18.1 | 2.64 | 8.03 | 1.08E-24 | 7.07E-24 |
| AF131216.5 | 4.40 | 5.25 | 1.12E-24 | 7.35E-24 |
| CTD-2008L17.1 | 4.14 | 6.19 | 1.12E-24 | 7.37E-24 |
| RP11-148B3.1 | 5.04 | 3.73 | 1.19E-24 | 7.82E-24 |
| LINC00184 | 2.29 | 4.90 | 1.20E-24 | 7.83E-24 |
| LINC00928 | 3.85 | 2.88 | 1.23E-24 | 8.01E-24 |
| RP11-297P16.3 | 5.41 | 4.06 | 1.31E-24 | 8.54E-24 |
| LVCAT1 | 3.69 | 5.82 | 1.33E-24 | 8.65E-24 |
| RP11-545D19.1 | 4.76 | 3.46 | 1.34E-24 | 8.71E-24 |
| RP11-159H10.3 | 2.29 | 4.95 | 1.40E-24 | 9.10E-24 |
| LINC00973 | 5.55 | 6.83 | 1.46E-24 | 9.48E-24 |
| CASC20 | 4.60 | 5.61 | 2.06E-24 | 1.34E-23 |
| C20orf197 | 2.56 | 7.55 | 2.25E-24 | 1.45E-23 |
| SMILR | 3.47 | 4.56 | 2.31E-24 | 1.49E-23 |
| AC006946.16 | 2.38 | 6.15 | 2.52E-24 | 1.63E-23 |
| RP1-170O19.14 | 4.06 | 3.11 | 2.59E-24 | 1.67E-23 |
| RP11-332J15.3 | 2.19 | 3.80 | 2.81E-24 | 1.80E-23 |
| LINC01391 | 4.84 | 4.09 | 3.04E-24 | 1.95E-23 |
| AC007389.3 | 3.66 | 3.94 | 3.64E-24 | 2.33E-23 |
| LINC01752 | 3.34 | 6.04 | 4.06E-24 | 2.59E-23 |
| LINC01105 | -2.26 | 5.52 | 4.17E-24 | 2.66E-23 |
| RP11-157I4.4 | 3.69 | 3.10 | 4.45E-24 | 2.84E-23 |
| RP11-1042B17.3 | 3.43 | 3.84 | 5.07E-24 | 3.22E-23 |
| RP11-1038A11.2 | 3.50 | 3.16 | 5.11E-24 | 3.24E-23 |
| POU6F2-AS1 | 4.09 | 2.98 | 5.32E-24 | 3.37E-23 |
| LINC02041 | 2.24 | 6.74 | 5.60E-24 | 3.54E-23 |
| RP1-35C21.2 | 3.77 | 3.91 | 5.68E-24 | 3.59E-23 |
| LINC01133 | 3.55 | 10.06 | 6.86E-24 | 4.32E-23 |
| MIAT | 2.01 | 11.42 | 7.11E-24 | 4.47E-23 |
| AC007405.8 | 3.06 | 3.19 | 7.23E-24 | 4.54E-23 |
| AC006159.4 | -2.25 | 2.62 | 7.52E-24 | 4.71E-23 |
| LINC01213 | 3.57 | 3.85 | 8.49E-24 | 5.31E-23 |
| RP4-547N15.3 | -2.39 | 5.15 | 9.11E-24 | 5.69E-23 |
| AC007193.6 | 3.95 | 4.85 | 9.79E-24 | 6.10E-23 |
| FAM181A-AS1 | -2.00 | 5.13 | 9.91E-24 | 6.16E-23 |
| AC016723.4 | 5.13 | 4.65 | 1.06E-23 | 6.56E-23 |
| RP11-366L20.3 | 3.49 | 3.28 | 1.14E-23 | 7.09E-23 |
| RP11-63E9.1 | 3.91 | 5.60 | 1.17E-23 | 7.22E-23 |
| RP11-445F12.1 | 5.90 | 5.01 | 1.20E-23 | 7.39E-23 |
| RP11-642C5.1 | 3.27 | 3.64 | 1.20E-23 | 7.41E-23 |
| RP11-685G9.2 | 2.77 | 3.23 | 1.34E-23 | 8.26E-23 |
| RP11-352B15.2 | 5.65 | 4.25 | 1.41E-23 | 8.68E-23 |
| ZFHX4-AS1 | 4.82 | 5.11 | 1.41E-23 | 8.68E-23 |
| RP11-83M16.6 | 3.70 | 5.01 | 1.42E-23 | 8.75E-23 |
| RP11-3J1.1 | 4.70 | 3.94 | 1.46E-23 | 8.94E-23 |
| CTD-2535I10.1 | 4.78 | 4.16 | 1.48E-23 | 9.09E-23 |
| AC097713.3 | 5.78 | 5.34 | 1.48E-23 | 9.09E-23 |
| AC004870.4 | 4.03 | 5.38 | 1.53E-23 | 9.35E-23 |
| RP11-107M16.2 | 3.35 | 3.78 | 1.68E-23 | 1.03E-22 |
| RP11-831A10.1 | 7.08 | 5.60 | 1.70E-23 | 1.04E-22 |
| LINC02029 | 2.94 | 3.83 | 1.74E-23 | 1.06E-22 |
| LINC01983 | 3.10 | 5.63 | 1.75E-23 | 1.07E-22 |
| CTD-2013M15.1 | 4.32 | 4.18 | 2.26E-23 | 1.37E-22 |
| LEMD1-AS1 | 2.08 | 4.55 | 2.47E-23 | 1.49E-22 |
| CASC11 | 2.43 | 4.82 | 2.49E-23 | 1.51E-22 |
| RP11-1000B6.2 | 2.47 | 2.71 | 3.19E-23 | 1.93E-22 |
| AF015262.2 | 3.47 | 3.13 | 3.24E-23 | 1.95E-22 |
| LINC00645 | 4.04 | 3.50 | 3.84E-23 | 2.31E-22 |
| CTA-390C10.9 | 3.33 | 2.90 | 4.52E-23 | 2.71E-22 |
| RP11-445P17.8 | 2.86 | 6.75 | 5.05E-23 | 3.02E-22 |
| SSTR5-AS1 | 4.71 | 5.42 | 5.15E-23 | 3.08E-22 |
| CTC-431G16.2 | 3.41 | 5.43 | 5.32E-23 | 3.18E-22 |
| RP11-478J18.2 | 3.32 | 4.20 | 5.50E-23 | 3.28E-22 |
| RP11-314N14.1 | 6.78 | 4.79 | 5.62E-23 | 3.35E-22 |
| LINC01687 | 5.07 | 3.53 | 5.65E-23 | 3.37E-22 |
| ERVH48-1 | 4.09 | 8.40 | 6.82E-23 | 4.05E-22 |
| LA16c-312E8.2 | 2.08 | 3.12 | 7.10E-23 | 4.22E-22 |
| RP11-21L23.4 | 3.77 | 5.58 | 7.88E-23 | 4.66E-22 |
| RP11-379K22.3 | 3.27 | 4.86 | 8.44E-23 | 4.98E-22 |
| RP11-416I2.1 | 2.49 | 5.85 | 8.82E-23 | 5.20E-22 |
| RP11-376O6.2 | 2.91 | 2.74 | 9.04E-23 | 5.32E-22 |
| RP11-567M16.1 | 2.53 | 7.23 | 9.09E-23 | 5.35E-22 |
| LINC01992 | 5.93 | 5.25 | 9.44E-23 | 5.55E-22 |
| RP11-114H21.2 | 5.32 | 3.89 | 9.74E-23 | 5.72E-22 |
| LINC00640 | 2.75 | 6.15 | 1.00E-22 | 5.90E-22 |
| HOXD-AS2 | 2.76 | 5.80 | 1.08E-22 | 6.32E-22 |
| RP11-209K10.2 | 5.02 | 4.19 | 1.08E-22 | 6.33E-22 |
| LINC00470 | 3.77 | 7.05 | 1.12E-22 | 6.57E-22 |
| RP11-383J24.1 | 3.61 | 3.84 | 1.14E-22 | 6.63E-22 |
| RP11-268G12.1 | 4.01 | 4.25 | 1.19E-22 | 6.97E-22 |
| RP11-348J24.2 | 3.19 | 4.58 | 1.27E-22 | 7.39E-22 |
| RP11-280K24.4 | 3.36 | 4.33 | 1.27E-22 | 7.39E-22 |
| MKRN3-AS1 | 3.29 | 3.48 | 1.38E-22 | 8.03E-22 |
| KB-1991G8.1 | 2.39 | 5.42 | 1.42E-22 | 8.22E-22 |
| RP11-150O12.6 | 2.44 | 6.79 | 1.47E-22 | 8.54E-22 |
| RP3-522D1.1 | 2.76 | 4.40 | 1.49E-22 | 8.64E-22 |
| LINC01269 | 2.34 | 5.91 | 1.54E-22 | 8.92E-22 |
| AC008088.4 | 3.15 | 3.18 | 1.80E-22 | 1.03E-21 |
| RP11-412P11.1 | 5.41 | 4.11 | 1.80E-22 | 1.04E-21 |
| RP11-285C1.2 | 3.78 | 3.44 | 1.81E-22 | 1.04E-21 |
| RP11-734K21.3 | 3.50 | 3.26 | 1.85E-22 | 1.06E-21 |
| CTB-60B18.12 | 2.18 | 3.44 | 1.91E-22 | 1.10E-21 |
| RP11-675F6.3 | 3.90 | 4.40 | 2.45E-22 | 1.40E-21 |
| CYP4F26P | 2.74 | 6.03 | 2.48E-22 | 1.41E-21 |
| RP11-962G15.1 | 6.13 | 4.45 | 2.50E-22 | 1.42E-21 |
| RP11-476K15.1 | 4.83 | 5.34 | 2.54E-22 | 1.45E-21 |
| AC005863.1 | 4.61 | 4.53 | 2.57E-22 | 1.46E-21 |
| CTD-2008P7.1 | 2.58 | 3.39 | 2.60E-22 | 1.48E-21 |
| RP11-144A16.8 | -2.44 | 3.49 | 2.70E-22 | 1.53E-21 |
| RP11-70F11.7 | 5.14 | 4.19 | 2.75E-22 | 1.56E-21 |
| TBL1XR1-AS1 | 2.59 | 3.77 | 2.95E-22 | 1.67E-21 |
| XX-C2158C6.3 | 3.22 | 3.37 | 3.06E-22 | 1.73E-21 |
| LINC01697 | 4.05 | 6.41 | 3.43E-22 | 1.94E-21 |
| AC112721.1 | 2.38 | 3.91 | 3.57E-22 | 2.02E-21 |
| RP5-1097P24.1 | 6.07 | 5.43 | 3.71E-22 | 2.09E-21 |
| CTB-49A3.2 | 2.25 | 4.23 | 3.74E-22 | 2.11E-21 |
| LINC01571 | -2.39 | 4.25 | 3.90E-22 | 2.19E-21 |
| FLJ16779 | 2.59 | 6.05 | 3.91E-22 | 2.20E-21 |
| AC073321.4 | 3.09 | 2.98 | 4.42E-22 | 2.48E-21 |
| LUCAT1 | 2.23 | 8.44 | 4.71E-22 | 2.64E-21 |
| TTLL11-IT1 | 2.65 | 4.31 | 4.81E-22 | 2.69E-21 |
| RP11-557H15.3 | 3.46 | 7.65 | 4.84E-22 | 2.71E-21 |
| RP11-259O2.2 | 4.33 | 3.72 | 6.02E-22 | 3.35E-21 |
| LINC02208 | 3.22 | 3.68 | 6.08E-22 | 3.38E-21 |
| RP11-62L18.3 | 4.07 | 2.91 | 6.27E-22 | 3.49E-21 |
| RP13-631K18.3 | 2.49 | 3.85 | 6.27E-22 | 3.49E-21 |
| KCNH1-IT1 | 4.50 | 3.54 | 6.76E-22 | 3.75E-21 |
| RP11-101E5.1 | -2.40 | 3.20 | 6.77E-22 | 3.75E-21 |
| LINC01956 | 4.67 | 4.42 | 6.85E-22 | 3.80E-21 |
| RP11-503C24.6 | 4.24 | 4.87 | 6.88E-22 | 3.81E-21 |
| XXbac-BPG308K3.5 | 2.38 | 4.11 | 7.08E-22 | 3.92E-21 |
| RP11-675F6.4 | 3.89 | 4.15 | 7.21E-22 | 3.99E-21 |
| RP11-669N7.2 | 6.97 | 5.81 | 7.45E-22 | 4.12E-21 |
| RP11-436D23.1 | 4.05 | 3.30 | 7.97E-22 | 4.40E-21 |
| RP11-706C16.8 | 3.80 | 3.10 | 8.17E-22 | 4.51E-21 |
| RP11-311F12.1 | 3.17 | 6.34 | 8.34E-22 | 4.60E-21 |
| RP11-225N10.1 | 3.22 | 4.01 | 8.39E-22 | 4.62E-21 |
| LINC01792 | 3.05 | 3.37 | 9.18E-22 | 5.05E-21 |
| CTD-2620I22.3 | 3.02 | 4.39 | 9.63E-22 | 5.29E-21 |
| LINC01511 | 5.42 | 6.49 | 9.85E-22 | 5.41E-21 |
| RP11-486L19.2 | 3.59 | 5.42 | 9.91E-22 | 5.44E-21 |
| RP11-499F3.1 | 2.89 | 4.39 | 1.00E-21 | 5.49E-21 |
| AC092669.6 | 3.50 | 2.84 | 1.05E-21 | 5.78E-21 |
| LINC00682 | 5.21 | 3.87 | 1.13E-21 | 6.17E-21 |
| LINC01224 | 2.99 | 7.83 | 1.32E-21 | 7.21E-21 |
| LINC01419 | 7.89 | 7.75 | 1.33E-21 | 7.28E-21 |
| RP5-1011O1.2 | 2.98 | 4.81 | 1.44E-21 | 7.84E-21 |
| RP11-329E24.6 | 4.34 | 3.35 | 1.49E-21 | 8.12E-21 |
| RP11-576D8.4 | 2.56 | 3.91 | 1.63E-21 | 8.86E-21 |
| AC068580.7 | 3.21 | 3.30 | 1.65E-21 | 8.93E-21 |
| RP11-114H23.2 | 2.71 | 4.46 | 1.66E-21 | 8.98E-21 |
| SPATA3-AS1 | 2.26 | 3.84 | 1.67E-21 | 9.03E-21 |
| SAMMSON | 2.73 | 3.50 | 1.69E-21 | 9.12E-21 |
| LINC00659 | 3.43 | 4.35 | 1.98E-21 | 1.07E-20 |
| LINC01361 | 2.42 | 3.83 | 1.98E-21 | 1.07E-20 |
| XXYLT1-AS1 | 2.36 | 3.58 | 2.11E-21 | 1.14E-20 |
| FNDC1-IT1 | 2.28 | 3.06 | 2.11E-21 | 1.14E-20 |
| RP11-300E4.2 | 2.89 | 3.26 | 2.18E-21 | 1.17E-20 |
| CDKN2A-AS1 | 2.99 | 3.63 | 2.32E-21 | 1.24E-20 |
| RP1-202O8.2 | 2.42 | 3.81 | 2.42E-21 | 1.30E-20 |
| RP11-1084E5.1 | 2.23 | 4.78 | 2.53E-21 | 1.35E-20 |
| XXyac-YM21GA2.3 | -2.50 | 3.53 | 2.60E-21 | 1.39E-20 |
| AC003986.6 | 2.52 | 3.32 | 2.61E-21 | 1.40E-20 |
| AC114765.2 | 3.06 | 2.89 | 2.71E-21 | 1.44E-20 |
| RP11-336A10.4 | 3.81 | 5.85 | 2.74E-21 | 1.46E-20 |
| LINC01356 | 2.43 | 5.05 | 3.11E-21 | 1.65E-20 |
| PART1 | 3.40 | 8.32 | 3.33E-21 | 1.76E-20 |
| LINC01322 | 3.75 | 5.14 | 3.33E-21 | 1.76E-20 |
| RP11-13K12.2 | 4.27 | 5.01 | 3.86E-21 | 2.04E-20 |
| CTD-2587M23.1 | 3.93 | 3.99 | 4.19E-21 | 2.21E-20 |
| LINC00601 | 3.82 | 3.88 | 4.23E-21 | 2.23E-20 |
| RP11-418I22.2 | -2.17 | 2.67 | 4.29E-21 | 2.26E-20 |
| CTD-2021H9.1 | 3.40 | 3.14 | 4.42E-21 | 2.33E-20 |
| RP4-781K5.9 | 3.56 | 3.02 | 4.42E-21 | 2.33E-20 |
| RP11-1070A24.2 | 3.32 | 2.93 | 4.45E-21 | 2.34E-20 |
| CTD-2091N23.1 | 2.63 | 3.90 | 5.33E-21 | 2.80E-20 |
| LINC01280 | 3.65 | 3.01 | 5.36E-21 | 2.81E-20 |
| RP1-101G11.3 | 4.90 | 4.55 | 5.51E-21 | 2.89E-20 |
| AP000679.2 | 2.51 | 3.63 | 5.55E-21 | 2.90E-20 |
| LINC00704 | 3.06 | 6.42 | 5.61E-21 | 2.94E-20 |
| RP11-123O10.4 | 2.44 | 3.37 | 5.62E-21 | 2.94E-20 |
| RP11-789C1.2 | -2.16 | 3.03 | 5.84E-21 | 3.05E-20 |
| RP11-146E13.4 | 2.73 | 3.36 | 5.88E-21 | 3.07E-20 |
| RP11-400N13.2 | 4.05 | 6.84 | 5.95E-21 | 3.10E-20 |
| RP3-495K2.2 | 2.52 | 3.11 | 6.09E-21 | 3.17E-20 |
| RP11-587P21.2 | 6.49 | 5.62 | 6.33E-21 | 3.29E-20 |
| LINC01592 | 3.04 | 3.60 | 6.48E-21 | 3.37E-20 |
| RP11-227H15.4 | 3.57 | 4.62 | 6.90E-21 | 3.59E-20 |
| RP11-474D1.3 | 7.06 | 7.51 | 7.89E-21 | 4.09E-20 |
| AC068138.1 | 5.40 | 4.11 | 7.99E-21 | 4.14E-20 |
| RP11-78C3.1 | 3.96 | 3.80 | 8.06E-21 | 4.17E-20 |
| AC091814.3 | 2.76 | 4.84 | 8.10E-21 | 4.19E-20 |
| RP4-738P15.1 | 3.15 | 4.53 | 8.12E-21 | 4.20E-20 |
| EWSAT1 | 2.59 | 6.20 | 8.89E-21 | 4.59E-20 |
| RP1-313L4.3 | 2.25 | 4.46 | 8.92E-21 | 4.60E-20 |
| RP11-237N19.3 | 3.27 | 3.25 | 9.22E-21 | 4.75E-20 |
| RP11-414H23.3 | 3.19 | 3.14 | 9.42E-21 | 4.84E-20 |
| RP11-220C2.1 | 2.71 | 3.47 | 1.04E-20 | 5.34E-20 |
| PTCSC2 | 3.56 | 4.12 | 1.07E-20 | 5.51E-20 |
| CTD-2547H18.1 | 3.08 | 6.00 | 1.13E-20 | 5.79E-20 |
| RP11-275I4.2 | 2.18 | 3.54 | 1.18E-20 | 6.06E-20 |
| BCAR4 | 5.73 | 5.81 | 1.26E-20 | 6.42E-20 |
| RP11-404J23.1 | 5.08 | 3.52 | 1.36E-20 | 6.96E-20 |
| LINC02167 | 7.10 | 5.53 | 1.37E-20 | 7.00E-20 |
| RP11-133K1.7 | 2.27 | 2.59 | 1.37E-20 | 7.00E-20 |
| RP11-308B16.2 | 5.74 | 4.63 | 1.38E-20 | 7.04E-20 |
| LINC01854 | 6.41 | 4.89 | 1.41E-20 | 7.16E-20 |
| RP11-422J15.1 | 3.25 | 2.59 | 1.46E-20 | 7.41E-20 |
| RP11-1020M18.10 | 4.66 | 3.51 | 1.54E-20 | 7.82E-20 |
| RP11-110A12.2 | 5.20 | 5.92 | 1.58E-20 | 7.98E-20 |
| RP11-235G24.3 | 4.86 | 3.50 | 1.65E-20 | 8.35E-20 |
| AF131217.1 | 2.82 | 8.86 | 1.86E-20 | 9.39E-20 |
| RP4-712E4.1 | 3.23 | 3.13 | 1.98E-20 | 9.97E-20 |
| RP11-297P16.4 | 7.62 | 7.76 | 2.19E-20 | 1.10E-19 |
| RP1-118J21.5 | 2.35 | 3.47 | 2.30E-20 | 1.15E-19 |
| RP11-69G7.1 | 5.62 | 5.71 | 2.38E-20 | 1.20E-19 |
| RP11-454P21.1 | 3.55 | 3.85 | 2.49E-20 | 1.25E-19 |
| CTD-3060P21.1 | 2.29 | 5.24 | 2.51E-20 | 1.26E-19 |
| RP1-46F2.3 | 4.71 | 4.75 | 2.55E-20 | 1.28E-19 |
| AC007966.1 | 2.03 | 4.61 | 2.59E-20 | 1.30E-19 |
| RP11-467L19.16 | 4.02 | 5.06 | 2.84E-20 | 1.42E-19 |
| RP11-129M6.1 | 3.14 | 6.86 | 2.91E-20 | 1.45E-19 |
| FLJ42969 | 2.25 | 4.76 | 2.99E-20 | 1.49E-19 |
| AC006548.28 | 2.44 | 5.30 | 3.10E-20 | 1.54E-19 |
| LINC01494 | 2.68 | 2.59 | 3.14E-20 | 1.56E-19 |
| LINC00866 | 2.21 | 3.70 | 3.20E-20 | 1.59E-19 |
| AC079610.1 | 2.57 | 4.01 | 3.22E-20 | 1.60E-19 |
| ATP6V1B1-AS1 | 2.43 | 3.76 | 3.63E-20 | 1.81E-19 |
| RP11-283G6.5 | 3.55 | 4.08 | 3.91E-20 | 1.94E-19 |
| LINC01901 | 4.01 | 5.37 | 4.44E-20 | 2.20E-19 |
| H19 | 3.36 | 13.74 | 4.46E-20 | 2.21E-19 |
| ELDR | 3.57 | 4.96 | 4.50E-20 | 2.22E-19 |
| LINC02141 | 4.62 | 3.76 | 4.52E-20 | 2.23E-19 |
| RMDN2-AS1 | 2.00 | 5.17 | 4.55E-20 | 2.25E-19 |
| RP11-221N13.3 | 3.08 | 4.95 | 4.60E-20 | 2.27E-19 |
| LINC01908 | -2.11 | 3.75 | 4.61E-20 | 2.28E-19 |
| RP11-353N14.3 | 3.00 | 2.57 | 4.90E-20 | 2.41E-19 |
| ATP13A4-AS1 | -2.00 | 6.08 | 5.08E-20 | 2.50E-19 |
| RP11-401O9.3 | 3.12 | 4.11 | 5.08E-20 | 2.50E-19 |
| RP11-180M15.3 | 3.14 | 3.06 | 5.14E-20 | 2.52E-19 |
| LINC02122 | 3.75 | 4.57 | 5.37E-20 | 2.64E-19 |
| RAPGEF4-AS1 | 2.78 | 3.18 | 5.46E-20 | 2.68E-19 |
| CTD-2265O21.3 | 3.28 | 2.77 | 5.70E-20 | 2.79E-19 |
| CTD-2066L21.1 | 4.10 | 3.27 | 5.80E-20 | 2.84E-19 |
| RP11-1070N10.5 | 4.35 | 4.82 | 6.29E-20 | 3.07E-19 |
| CTB-33O18.1 | 4.61 | 3.87 | 6.31E-20 | 3.08E-19 |
| FER1L6-AS2 | 4.99 | 4.07 | 6.34E-20 | 3.09E-19 |
| AC003092.1 | 4.76 | 5.38 | 7.26E-20 | 3.53E-19 |
| CTD-2501M5.1 | -2.13 | 3.69 | 7.57E-20 | 3.67E-19 |
| ERVMER61-1 | 5.80 | 4.17 | 7.71E-20 | 3.74E-19 |
| AC141930.2 | 2.75 | 3.26 | 7.89E-20 | 3.82E-19 |
| DRAIC | 3.03 | 9.09 | 9.61E-20 | 4.64E-19 |
| LINC01344 | 2.35 | 3.98 | 1.02E-19 | 4.94E-19 |
| RP11-319E16.2 | 3.16 | 2.67 | 1.03E-19 | 4.97E-19 |
| RP4-799P18.2 | 2.40 | 4.23 | 1.03E-19 | 4.98E-19 |
| RP11-625L16.3 | 2.36 | 3.40 | 1.14E-19 | 5.49E-19 |
| LINC02133 | 3.47 | 4.78 | 1.20E-19 | 5.75E-19 |
| RP11-255G12.3 | 3.78 | 3.50 | 1.27E-19 | 6.08E-19 |
| LINC00462 | 4.13 | 4.76 | 1.37E-19 | 6.56E-19 |
| RP11-471M2.3 | 5.19 | 3.83 | 1.45E-19 | 6.94E-19 |
| RP5-1121A15.3 | 4.14 | 3.39 | 1.47E-19 | 7.05E-19 |
| TLX1NB | 4.27 | 3.15 | 1.48E-19 | 7.06E-19 |
| RP11-538I12.3 | 3.24 | 3.55 | 1.50E-19 | 7.19E-19 |
| GRM5-AS1 | 2.84 | 3.51 | 1.53E-19 | 7.30E-19 |
| MNX1-AS2 | 2.25 | 4.69 | 1.56E-19 | 7.42E-19 |
| LINC01926 | 2.55 | 3.12 | 1.57E-19 | 7.48E-19 |
| LINC01117 | 2.13 | 4.99 | 1.67E-19 | 7.96E-19 |
| LINC01399 | 2.87 | 3.14 | 1.77E-19 | 8.39E-19 |
| RP11-1070N10.7 | 4.41 | 4.23 | 1.80E-19 | 8.53E-19 |
| CTD-2616J11.16 | 2.31 | 3.18 | 1.88E-19 | 8.90E-19 |
| AF121898.3 | 4.38 | 3.43 | 1.89E-19 | 8.94E-19 |
| RP11-398G24.2 | 3.87 | 3.01 | 2.05E-19 | 9.68E-19 |
| CH507-154B10.2 | 2.02 | 3.22 | 2.17E-19 | 1.02E-18 |
| AC004158.3 | 3.88 | 3.81 | 2.24E-19 | 1.06E-18 |
| AC084149.2 | 3.60 | 2.72 | 2.40E-19 | 1.13E-18 |
| RP13-192B19.2 | 3.01 | 2.97 | 2.41E-19 | 1.14E-18 |
| RP11-217E22.5 | 3.95 | 3.15 | 2.73E-19 | 1.28E-18 |
| RP11-109M17.2 | 3.72 | 5.00 | 2.77E-19 | 1.30E-18 |
| RP11-354I13.1 | 4.25 | 3.09 | 2.86E-19 | 1.34E-18 |
| RP11-314D7.1 | 3.75 | 2.82 | 3.19E-19 | 1.49E-18 |
| RP11-565A3.2 | 5.60 | 4.00 | 3.33E-19 | 1.56E-18 |
| RP11-103J17.2 | 5.84 | 5.15 | 3.37E-19 | 1.57E-18 |
| RP11-153K16.1 | 4.45 | 3.87 | 3.64E-19 | 1.70E-18 |
| RP4-734C18.1 | 4.81 | 4.43 | 4.12E-19 | 1.92E-18 |
| RP11-390F4.6 | 2.39 | 4.73 | 4.30E-19 | 2.00E-18 |
| RP11-135A1.3 | 3.81 | 3.29 | 4.32E-19 | 2.01E-18 |
| LINC01896 | 6.13 | 4.92 | 4.37E-19 | 2.03E-18 |
| RP11-626P14.2 | 4.23 | 3.80 | 4.84E-19 | 2.25E-18 |
| LINC01527 | 4.13 | 5.37 | 5.04E-19 | 2.34E-18 |
| LINC02065 | 2.82 | 3.22 | 5.12E-19 | 2.37E-18 |
| LINC00160 | 2.83 | 4.52 | 5.15E-19 | 2.39E-18 |
| LINC01416 | 3.14 | 2.86 | 5.30E-19 | 2.45E-18 |
| RP11-338H14.1 | 3.53 | 4.51 | 5.55E-19 | 2.56E-18 |
| AC022201.4 | 4.23 | 3.46 | 5.85E-19 | 2.70E-18 |
| RP11-320G10.1 | 4.32 | 4.67 | 5.86E-19 | 2.70E-18 |
| LINC02048 | 3.21 | 3.61 | 6.09E-19 | 2.80E-18 |
| AC004920.3 | 3.23 | 2.60 | 6.97E-19 | 3.20E-18 |
| RP11-114M1.2 | 3.60 | 3.18 | 7.18E-19 | 3.29E-18 |
| RP11-314D7.2 | 3.63 | 3.00 | 7.21E-19 | 3.30E-18 |
| CTC-420A11.2 | 3.05 | 4.34 | 7.25E-19 | 3.32E-18 |
| LINC01597 | 2.97 | 7.11 | 7.59E-19 | 3.47E-18 |
| AC093911.1 | -2.36 | 2.52 | 7.68E-19 | 3.51E-18 |
| RP1-40E16.9 | 5.93 | 4.97 | 7.74E-19 | 3.53E-18 |
| RP11-85O21.2 | 3.89 | 2.94 | 7.84E-19 | 3.58E-18 |
| AC093702.1 | 2.90 | 3.99 | 8.61E-19 | 3.91E-18 |
| RP4-631H13.2 | 2.64 | 2.53 | 9.10E-19 | 4.13E-18 |
| RP11-395N3.1 | 2.43 | 4.07 | 9.40E-19 | 4.26E-18 |
| MLIP-IT1 | 2.73 | 3.64 | 1.09E-18 | 4.88E-18 |
| PLUT | 4.65 | 3.39 | 1.26E-18 | 5.66E-18 |
| FLJ37505 | 5.51 | 4.86 | 1.43E-18 | 6.39E-18 |
| AC010731.2 | 3.81 | 4.64 | 1.46E-18 | 6.52E-18 |
| CTD-2021H9.2 | 2.97 | 2.87 | 1.60E-18 | 7.15E-18 |
| RP11-320M16.2 | 3.00 | 3.34 | 1.62E-18 | 7.21E-18 |
| TTTY16 | -2.19 | 3.01 | 1.86E-18 | 8.26E-18 |
| CTC-338M12.9 | 2.93 | 4.63 | 1.99E-18 | 8.84E-18 |
| LVCAT5 | 5.90 | 4.69 | 2.01E-18 | 8.93E-18 |
| RP11-681L8.1 | 3.52 | 3.40 | 2.45E-18 | 1.08E-17 |
| RP11-416N4.1 | 3.01 | 2.92 | 2.51E-18 | 1.11E-17 |
| RP11-395B7.2 | 2.83 | 4.25 | 2.57E-18 | 1.13E-17 |
| CTB-189B5.3 | 2.37 | 3.02 | 2.61E-18 | 1.15E-17 |
| RP11-401O9.4 | 2.78 | 4.44 | 2.69E-18 | 1.19E-17 |
| AC005042.5 | 3.57 | 2.81 | 2.82E-18 | 1.24E-17 |
| LINC00648 | 3.61 | 7.16 | 2.90E-18 | 1.27E-17 |
| RP11-680F20.10 | 2.57 | 3.47 | 2.96E-18 | 1.30E-17 |
| AC025016.1 | 4.99 | 3.72 | 3.07E-18 | 1.35E-17 |
| AP001626.2 | 2.03 | 4.45 | 3.14E-18 | 1.38E-17 |
| RP11-317N12.1 | 5.77 | 5.02 | 3.30E-18 | 1.44E-17 |
| LINC01543 | 3.27 | 3.25 | 3.37E-18 | 1.47E-17 |
| RP3-333B15.5 | 2.89 | 3.03 | 3.66E-18 | 1.60E-17 |
| AC073957.15 | 2.97 | 3.77 | 3.85E-18 | 1.68E-17 |
| RP11-445P17.3 | 2.22 | 3.45 | 3.94E-18 | 1.72E-17 |
| RP11-728G15.1 | 3.48 | 3.32 | 3.99E-18 | 1.74E-17 |
| RP11-498M15.1 | 3.40 | 3.15 | 4.11E-18 | 1.79E-17 |
| RP11-311F12.2 | 3.75 | 4.75 | 4.18E-18 | 1.82E-17 |
| AC006000.5 | 3.12 | 3.35 | 4.40E-18 | 1.91E-17 |
| LINC00567 | 2.85 | 4.87 | 4.51E-18 | 1.96E-17 |
| RP11-145G20.1 | 6.06 | 4.73 | 4.71E-18 | 2.04E-17 |
| RP11-260A9.6 | 5.75 | 4.16 | 4.83E-18 | 2.09E-17 |
| FAM230C | 5.94 | 4.82 | 5.00E-18 | 2.16E-17 |
| RP11-817J15.2 | 3.39 | 5.43 | 5.03E-18 | 2.17E-17 |
| ESRG | 6.59 | 8.13 | 5.08E-18 | 2.19E-17 |
| RP11-180C1.1 | 5.59 | 4.10 | 5.14E-18 | 2.22E-17 |
| RP1-97J1.2 | 3.68 | 4.34 | 5.21E-18 | 2.25E-17 |
| RP5-978I12.1 | -2.12 | 3.95 | 5.25E-18 | 2.26E-17 |
| RP11-662I13.2 | 2.45 | 4.00 | 5.43E-18 | 2.34E-17 |
| RP11-440G9.1 | 3.18 | 3.62 | 5.69E-18 | 2.44E-17 |
| FLJ22447 | 2.23 | 8.08 | 6.15E-18 | 2.64E-17 |
| RP11-17E2.2 | 2.19 | 4.07 | 6.93E-18 | 2.97E-17 |
| TDRG1 | 4.60 | 3.83 | 6.95E-18 | 2.98E-17 |
| CHODL-AS1 | 3.10 | 2.87 | 7.68E-18 | 3.28E-17 |
| LINC00391 | 3.11 | 2.90 | 8.20E-18 | 3.50E-17 |
| AC013463.2 | 2.28 | 6.84 | 8.46E-18 | 3.61E-17 |
| RP11-565P22.2 | 4.67 | 4.54 | 9.12E-18 | 3.88E-17 |
| RP3-395M20.2 | 2.25 | 3.93 | 9.46E-18 | 4.02E-17 |
| RP11-659E9.4 | 2.55 | 2.60 | 9.64E-18 | 4.09E-17 |
| LINC01549 | 3.93 | 4.06 | 1.02E-17 | 4.34E-17 |
| RP11-488P3.1 | 2.37 | 7.28 | 1.08E-17 | 4.59E-17 |
| WI2-85898F10.1 | 2.44 | 3.87 | 1.11E-17 | 4.71E-17 |
| LINC00698 | 2.39 | 2.94 | 1.12E-17 | 4.76E-17 |
| RP11-548L20.1 | 5.89 | 4.10 | 1.15E-17 | 4.88E-17 |
| RP11-120K24.5 | 2.06 | 4.91 | 1.20E-17 | 5.07E-17 |
| RP11-449P1.1 | 3.96 | 3.55 | 1.26E-17 | 5.30E-17 |
| RP11-615J4.3 | 5.94 | 4.18 | 1.39E-17 | 5.86E-17 |
| RP11-398B16.2 | 6.84 | 4.99 | 1.49E-17 | 6.26E-17 |
| LINC01910 | 2.79 | 3.51 | 1.50E-17 | 6.29E-17 |
| SAMSN1-AS1 | 2.58 | 3.73 | 1.52E-17 | 6.37E-17 |
| GS1-24F4.2 | 2.30 | 3.94 | 1.65E-17 | 6.90E-17 |
| RP11-65M17.3 | 2.82 | 3.96 | 1.65E-17 | 6.92E-17 |
| RP11-290L1.5 | 3.16 | 2.65 | 1.68E-17 | 7.02E-17 |
| AC009955.8 | 2.44 | 2.53 | 1.70E-17 | 7.09E-17 |
| LINC00319 | 2.58 | 3.15 | 1.71E-17 | 7.13E-17 |
| RP4-797C5.2 | 2.93 | 3.18 | 1.75E-17 | 7.29E-17 |
| CTD-2354A18.1 | 6.12 | 6.40 | 1.78E-17 | 7.41E-17 |
| LINC02137 | 2.74 | 4.92 | 1.79E-17 | 7.46E-17 |
| RP11-502M1.2 | 2.81 | 3.31 | 2.04E-17 | 8.46E-17 |
| RP11-359E19.2 | 3.72 | 7.18 | 2.07E-17 | 8.58E-17 |
| RP11-67K19.3 | 3.77 | 4.66 | 2.11E-17 | 8.75E-17 |
| RP11-687D19.1 | -2.32 | 2.54 | 2.14E-17 | 8.85E-17 |
| FLJ36000 | 5.70 | 4.57 | 2.23E-17 | 9.21E-17 |
| PANCR | 4.80 | 3.49 | 2.34E-17 | 9.69E-17 |
| RP11-116O18.1 | 4.07 | 8.58 | 2.35E-17 | 9.72E-17 |
| RP3-395M20.3 | 2.28 | 3.96 | 2.43E-17 | 1.00E-16 |
| FRGCA | 2.84 | 4.52 | 2.44E-17 | 1.01E-16 |
| CTA-392C11.1 | 7.48 | 6.30 | 2.49E-17 | 1.03E-16 |
| RP11-370A5.2 | 2.27 | 4.13 | 2.50E-17 | 1.03E-16 |
| AL121578.2 | 3.94 | 2.92 | 2.60E-17 | 1.07E-16 |
| RP11-142A23.1 | 2.04 | 3.43 | 2.66E-17 | 1.10E-16 |
| RP11-663N22.1 | 2.23 | 4.81 | 2.70E-17 | 1.11E-16 |
| RP11-60A8.1 | 4.23 | 4.84 | 2.78E-17 | 1.14E-16 |
| OTX2-AS1 | 3.67 | 3.94 | 2.86E-17 | 1.18E-16 |
| RP4-781K5.4 | 2.58 | 5.72 | 2.90E-17 | 1.19E-16 |
| CTB-107G13.1 | 2.77 | 5.63 | 2.95E-17 | 1.21E-16 |
| RP4-712E4.2 | 3.06 | 2.57 | 3.13E-17 | 1.29E-16 |
| LINC01127 | 2.72 | 8.16 | 3.50E-17 | 1.43E-16 |
| CASC16 | 3.29 | 3.94 | 3.65E-17 | 1.49E-16 |
| MIR5689HG | 2.92 | 2.92 | 3.68E-17 | 1.51E-16 |
| RP11-305F18.1 | 3.84 | 4.47 | 3.69E-17 | 1.51E-16 |
| KB-1930G5.4 | 2.78 | 2.73 | 3.73E-17 | 1.52E-16 |
| RP11-3L21.2 | 3.16 | 4.04 | 3.79E-17 | 1.55E-16 |
| CTC-261N6.1 | 4.11 | 2.96 | 3.90E-17 | 1.59E-16 |
| RP11-346D14.1 | 2.72 | 2.91 | 4.03E-17 | 1.64E-16 |
| AC009236.2 | 2.58 | 2.69 | 4.48E-17 | 1.82E-16 |
| RP11-897M7.1 | 3.58 | 3.30 | 4.61E-17 | 1.87E-16 |
| LINC00483 | 3.53 | 3.77 | 4.85E-17 | 1.96E-16 |
| RP11-838N2.5 | 3.06 | 4.61 | 5.03E-17 | 2.03E-16 |
| RP11-398J5.1 | 4.61 | 3.30 | 5.07E-17 | 2.05E-16 |
| RP11-269F21.3 | 4.70 | 4.92 | 5.08E-17 | 2.05E-16 |
| RP11-466P24.6 | -2.13 | 3.16 | 5.14E-17 | 2.08E-16 |
| RP11-209E8.1 | 4.40 | 3.13 | 5.21E-17 | 2.10E-16 |
| RP1-296L11.1 | 2.77 | 2.72 | 5.39E-17 | 2.17E-16 |
| RP11-332K15.1 | 2.91 | 3.39 | 5.50E-17 | 2.21E-16 |
| SCHLAP1 | 5.14 | 3.72 | 5.59E-17 | 2.25E-16 |
| LINC02111 | 3.14 | 3.05 | 5.98E-17 | 2.40E-16 |
| RP11-484N16.1 | 2.41 | 4.02 | 6.07E-17 | 2.44E-16 |
| RP11-817J15.3 | 3.50 | 4.91 | 6.45E-17 | 2.59E-16 |
| FOXCUT | 2.20 | 4.05 | 6.46E-17 | 2.59E-16 |
| RP6-24A23.7 | 4.32 | 8.63 | 7.39E-17 | 2.95E-16 |
| RP11-187E13.2 | 2.60 | 3.21 | 7.43E-17 | 2.97E-16 |
| RP11-485F13.1 | 5.45 | 4.15 | 7.53E-17 | 3.00E-16 |
| CTD-2210P24.1 | 4.11 | 3.04 | 7.70E-17 | 3.07E-16 |
| CTD-3064H18.4 | 2.57 | 5.21 | 8.01E-17 | 3.18E-16 |
| KB-1615E4.3 | 2.12 | 3.08 | 8.26E-17 | 3.29E-16 |
| LINC01448 | 4.15 | 3.08 | 8.39E-17 | 3.34E-16 |
| RP11-221N13.4 | 4.26 | 3.14 | 8.40E-17 | 3.34E-16 |
| RP11-256I23.3 | 2.67 | 3.00 | 8.58E-17 | 3.40E-16 |
| CTD-3194G12.2 | 2.20 | 3.35 | 9.13E-17 | 3.61E-16 |
| LINC01250 | 2.89 | 3.21 | 9.20E-17 | 3.64E-16 |
| RP11-279N8.1 | 2.61 | 3.09 | 9.31E-17 | 3.68E-16 |
| LINC01436 | 2.41 | 9.06 | 9.44E-17 | 3.72E-16 |
| LINC01667 | 5.80 | 6.14 | 9.50E-17 | 3.74E-16 |
| AC005162.5 | 2.44 | 3.51 | 1.03E-16 | 4.04E-16 |
| RP11-479J7.2 | 2.15 | 2.93 | 1.05E-16 | 4.12E-16 |
| RP11-136I14.5 | 3.51 | 3.00 | 1.05E-16 | 4.14E-16 |
| CTA-392E5.1 | 4.97 | 5.18 | 1.12E-16 | 4.41E-16 |
| CTC-353G13.1 | 4.40 | 3.42 | 1.13E-16 | 4.42E-16 |
| RP11-431M3.1 | 3.91 | 2.84 | 1.15E-16 | 4.49E-16 |
| DPP10-AS1 | 2.91 | 6.95 | 1.19E-16 | 4.66E-16 |
| IGF2BP2-AS1 | 2.40 | 4.73 | 1.38E-16 | 5.38E-16 |
| RP11-259O2.1 | 2.56 | 6.30 | 1.59E-16 | 6.17E-16 |
| LINC02152 | 4.11 | 3.72 | 1.63E-16 | 6.34E-16 |
| LINC01995 | 3.54 | 3.65 | 1.63E-16 | 6.35E-16 |
| RP11-332J15.2 | 5.05 | 3.95 | 1.71E-16 | 6.64E-16 |
| RP4-813D12.3 | 2.79 | 4.46 | 1.78E-16 | 6.88E-16 |
| RP11-108K14.12 | 2.13 | 4.02 | 1.80E-16 | 6.97E-16 |
| CTD-2555A7.2 | 2.63 | 3.35 | 2.06E-16 | 7.95E-16 |
| RP11-272B17.2 | 4.17 | 3.02 | 2.16E-16 | 8.36E-16 |
| RP11-573J24.1 | 3.47 | 2.60 | 2.29E-16 | 8.83E-16 |
| LINC00308 | 3.68 | 2.69 | 2.39E-16 | 9.19E-16 |
| LINC00676 | 9.14 | 7.78 | 2.40E-16 | 9.21E-16 |
| RP11-573D15.3 | 2.91 | 2.79 | 2.50E-16 | 9.59E-16 |
| AJ003147.9 | 3.18 | 2.75 | 2.73E-16 | 1.05E-15 |
| LINC00383 | 5.21 | 3.60 | 2.89E-16 | 1.11E-15 |
| LINC00051 | 3.82 | 3.91 | 3.13E-16 | 1.20E-15 |
| MYCNUT | 3.95 | 2.88 | 3.56E-16 | 1.36E-15 |
| RP11-689C9.1 | 2.74 | 3.72 | 3.56E-16 | 1.36E-15 |
| CTD-2251F13.1 | 3.51 | 3.16 | 3.81E-16 | 1.45E-15 |
| CHL1-AS1 | 2.10 | 3.64 | 3.87E-16 | 1.47E-15 |
| RP4-583K8.1 | 3.31 | 2.54 | 3.99E-16 | 1.51E-15 |
| RP11-109E24.1 | 2.02 | 3.27 | 4.05E-16 | 1.53E-15 |
| THRA1/BTR | 3.48 | 4.16 | 4.38E-16 | 1.66E-15 |
| RP11-802F5.1 | 3.60 | 3.45 | 4.43E-16 | 1.68E-15 |
| RP11-78A19.4 | 3.04 | 3.24 | 4.45E-16 | 1.68E-15 |
| RP11-776A13.1 | 3.90 | 3.01 | 4.51E-16 | 1.70E-15 |
| RP11-326N17.2 | 2.20 | 3.01 | 4.60E-16 | 1.73E-15 |
| RP11-14C10.6 | 3.07 | 3.45 | 4.84E-16 | 1.82E-15 |
| ERVK-28 | 2.81 | 2.86 | 5.58E-16 | 2.10E-15 |
| RP11-119K6.6 | 2.19 | 2.70 | 6.30E-16 | 2.36E-15 |
| LINC01098 | 3.09 | 3.34 | 6.94E-16 | 2.59E-15 |
| AC015933.2 | 3.29 | 2.97 | 6.99E-16 | 2.61E-15 |
| CTB-49A3.4 | 2.37 | 3.66 | 7.00E-16 | 2.61E-15 |
| GRM7-AS3 | 2.95 | 2.48 | 7.17E-16 | 2.67E-15 |
| RP4-676L2.1 | 2.64 | 3.62 | 7.26E-16 | 2.70E-15 |
| DSCAM-AS1 | 7.52 | 9.56 | 7.28E-16 | 2.71E-15 |
| RP11-78L16.1 | 5.16 | 3.65 | 7.61E-16 | 2.83E-15 |
| RP11-1109M24.5 | 5.40 | 4.46 | 8.17E-16 | 3.03E-15 |
| KB-1460A1.2 | 2.14 | 2.55 | 8.22E-16 | 3.05E-15 |
| LINC01598 | 2.03 | 3.18 | 8.60E-16 | 3.18E-15 |
| RP11-20G13.1 | 2.49 | 4.37 | 9.54E-16 | 3.52E-15 |
| RP11-254I22.3 | 2.74 | 5.89 | 9.54E-16 | 3.52E-15 |
| RP11-497G19.7 | 2.26 | 3.60 | 9.55E-16 | 3.52E-15 |
| LINC00974 | 2.56 | 2.66 | 1.03E-15 | 3.78E-15 |
| RP11-297L17.2 | 4.86 | 3.71 | 1.03E-15 | 3.79E-15 |
| RPPH1 | 5.19 | 7.10 | 1.04E-15 | 3.84E-15 |
| RP11-13K12.1 | 2.59 | 6.37 | 1.11E-15 | 4.08E-15 |
| RP11-211C9.1 | 2.71 | 5.11 | 1.18E-15 | 4.32E-15 |
| LINC00221 | 5.46 | 7.04 | 1.21E-15 | 4.43E-15 |
| RP11-11N5.1 | 3.65 | 3.96 | 1.22E-15 | 4.45E-15 |
| CTD-2377D24.4 | 4.71 | 3.73 | 1.40E-15 | 5.10E-15 |
| RP11-68I3.7 | 2.30 | 2.56 | 1.44E-15 | 5.23E-15 |
| RP11-1260E13.1 | 2.85 | 6.32 | 1.57E-15 | 5.69E-15 |
| LINC01143 | 2.84 | 3.91 | 1.58E-15 | 5.72E-15 |
| AC106875.1 | 4.78 | 5.52 | 1.61E-15 | 5.85E-15 |
| LINC01370 | 6.62 | 5.06 | 1.68E-15 | 6.08E-15 |
| RP11-378I6.1 | 2.63 | 3.66 | 1.69E-15 | 6.13E-15 |
| RP11-625H11.2 | 3.70 | 3.17 | 1.77E-15 | 6.39E-15 |
| RP3-359N14.2 | 2.61 | 2.75 | 1.80E-15 | 6.51E-15 |
| RP11-556I14.2 | 2.06 | 3.02 | 2.04E-15 | 7.32E-15 |
| RP11-94M14.2 | 3.51 | 2.94 | 2.07E-15 | 7.45E-15 |
| RP11-186F10.2 | 2.52 | 3.26 | 2.19E-15 | 7.84E-15 |
| LINC01639 | 4.41 | 3.41 | 2.25E-15 | 8.03E-15 |
| RBAKDN | 2.38 | 4.21 | 2.25E-15 | 8.03E-15 |
| CTC-501O10.1 | 2.15 | 3.37 | 2.32E-15 | 8.29E-15 |
| CTD-2128A3.2 | 3.48 | 3.20 | 2.40E-15 | 8.57E-15 |
| RP11-501C14.5 | 4.03 | 2.99 | 2.51E-15 | 8.94E-15 |
| RP5-1119A7.14 | 2.18 | 4.55 | 2.55E-15 | 9.10E-15 |
| C3orf67-AS1 | 2.68 | 2.51 | 2.61E-15 | 9.30E-15 |
| RP4-715N11.2 | 2.73 | 2.92 | 2.63E-15 | 9.38E-15 |
| RP11-722M1.1 | 4.17 | 3.62 | 2.64E-15 | 9.41E-15 |
| RP11-167H9.5 | 3.80 | 3.00 | 2.93E-15 | 1.04E-14 |
| LINC01804 | 4.77 | 4.35 | 3.13E-15 | 1.11E-14 |
| AC096669.1 | 3.68 | 2.76 | 3.37E-15 | 1.20E-14 |
| AC079466.1 | 5.47 | 5.88 | 3.45E-15 | 1.22E-14 |
| bP-2171C21.3 | 3.71 | 3.28 | 3.64E-15 | 1.29E-14 |
| RP11-490G2.2 | 3.34 | 2.81 | 3.76E-15 | 1.33E-14 |
| AC027119.1 | 3.89 | 3.07 | 3.87E-15 | 1.37E-14 |
| PCAT2 | 2.46 | 3.00 | 3.98E-15 | 1.40E-14 |
| TBC1D3P1-DHX40P1 | 3.97 | 2.83 | 4.12E-15 | 1.45E-14 |
| RP11-488I20.8 | 4.60 | 3.96 | 4.15E-15 | 1.47E-14 |
| LINC02188 | 2.65 | 6.85 | 4.23E-15 | 1.49E-14 |
| CTD-2311M21.3 | 2.14 | 4.60 | 4.23E-15 | 1.49E-14 |
| RP11-586K2.1 | 2.26 | 3.51 | 4.35E-15 | 1.53E-14 |
| RP11-154H12.2 | 2.24 | 3.25 | 4.68E-15 | 1.65E-14 |
| RP11-26L20.3 | 2.70 | 3.41 | 4.82E-15 | 1.69E-14 |
| RP11-302F12.3 | 3.93 | 4.22 | 5.07E-15 | 1.78E-14 |
| ATP13A5-AS1 | 2.56 | 2.70 | 5.30E-15 | 1.86E-14 |
| LINC02170 | 2.03 | 5.21 | 5.36E-15 | 1.88E-14 |
| SLC7A11-AS1 | 2.04 | 6.18 | 5.73E-15 | 2.00E-14 |
| LINC01446 | 4.30 | 6.25 | 5.79E-15 | 2.02E-14 |
| LA16c-352F7.1 | 2.07 | 3.77 | 6.14E-15 | 2.14E-14 |
| U95743.1 | 3.85 | 2.82 | 6.30E-15 | 2.19E-14 |
| LINC02105 | 3.14 | 2.88 | 6.40E-15 | 2.23E-14 |
| RP11-314P15.2 | 3.17 | 2.58 | 6.62E-15 | 2.30E-14 |
| AC113617.1 | 3.46 | 2.73 | 6.66E-15 | 2.32E-14 |
| RP11-286B14.1 | 3.10 | 3.49 | 6.93E-15 | 2.41E-14 |
| RP4-737E23.2 | 2.50 | 7.41 | 7.10E-15 | 2.47E-14 |
| LINC01905 | 2.80 | 4.36 | 7.22E-15 | 2.51E-14 |
| AC074389.5 | 4.42 | 4.46 | 7.28E-15 | 2.52E-14 |
| MIR194-2HG | 2.16 | 4.46 | 7.59E-15 | 2.63E-14 |
| RP11-457M11.5 | 4.22 | 4.78 | 8.38E-15 | 2.90E-14 |
| LINC00524 | 2.84 | 4.13 | 9.20E-15 | 3.17E-14 |
| RP11-94P11.4 | 3.51 | 2.70 | 9.31E-15 | 3.21E-14 |
| LINC02128 | 3.16 | 3.54 | 9.33E-15 | 3.22E-14 |
| LINC01812 | 2.47 | 2.81 | 9.62E-15 | 3.31E-14 |
| AJ003147.8 | 3.12 | 2.63 | 9.69E-15 | 3.33E-14 |
| RP11-21C17.1 | 3.01 | 2.66 | 1.05E-14 | 3.60E-14 |
| AC137723.5 | 2.02 | 2.59 | 1.06E-14 | 3.63E-14 |
| RP11-196H14.2 | 2.20 | 2.49 | 1.06E-14 | 3.63E-14 |
| RP11-555J4.3 | 3.65 | 2.83 | 1.07E-14 | 3.66E-14 |
| RP11-156K13.2 | 2.99 | 2.61 | 1.11E-14 | 3.81E-14 |
| RP3-428L16.1 | 3.11 | 3.68 | 1.17E-14 | 4.01E-14 |
| RP11-284G10.1 | 4.66 | 3.44 | 1.19E-14 | 4.06E-14 |
| LINC01918 | 2.04 | 3.39 | 1.19E-14 | 4.08E-14 |
| AC008278.3 | 3.06 | 2.88 | 1.22E-14 | 4.15E-14 |
| CTD-2194D22.4 | 3.40 | 2.71 | 1.24E-14 | 4.23E-14 |
| LINC01959 | 2.85 | 2.48 | 1.25E-14 | 4.25E-14 |
| RP11-16L21.7 | 2.00 | 3.23 | 1.27E-14 | 4.34E-14 |
| RP11-386B13.4 | 3.81 | 3.27 | 1.33E-14 | 4.51E-14 |
| FGF12-AS2 | 2.37 | 3.47 | 1.48E-14 | 5.01E-14 |
| AC008060.7 | 4.16 | 3.21 | 1.53E-14 | 5.20E-14 |
| CTD-2374C24.1 | 4.13 | 3.25 | 1.59E-14 | 5.36E-14 |
| RP3-410C9.2 | 3.28 | 4.69 | 1.63E-14 | 5.49E-14 |
| RP11-274M17.3 | 3.38 | 2.67 | 1.64E-14 | 5.54E-14 |
| RP11-234O6.2 | 5.28 | 3.75 | 1.74E-14 | 5.86E-14 |
| RP4-745K6.1 | 3.15 | 2.51 | 1.77E-14 | 5.96E-14 |
| RP11-61J19.2 | 2.56 | 2.98 | 1.78E-14 | 6.00E-14 |
| RP11-746B8.1 | 2.57 | 2.56 | 1.79E-14 | 6.02E-14 |
| RP11-402J6.1 | 3.01 | 2.77 | 1.80E-14 | 6.06E-14 |
| RP11-79E3.3 | 4.99 | 3.64 | 1.82E-14 | 6.12E-14 |
| RP11-649A16.1 | 3.95 | 2.90 | 1.85E-14 | 6.21E-14 |
| RP11-202K23.1 | 4.29 | 2.98 | 1.87E-14 | 6.27E-14 |
| RP11-497G19.1 | 3.91 | 6.62 | 1.88E-14 | 6.30E-14 |
| RP13-60M5.2 | 4.29 | 2.99 | 1.91E-14 | 6.40E-14 |
| LINC01994 | 2.97 | 3.29 | 1.96E-14 | 6.55E-14 |
| RP11-430H10.2 | 3.81 | 4.26 | 2.03E-14 | 6.79E-14 |
| RP1-269M15.3 | 4.10 | 5.16 | 2.17E-14 | 7.24E-14 |
| LINC00824 | 3.18 | 5.45 | 2.19E-14 | 7.31E-14 |
| LA16c-60D12.2 | 4.11 | 3.22 | 2.21E-14 | 7.35E-14 |
| RP11-279F6.2 | 4.61 | 5.44 | 2.47E-14 | 8.22E-14 |
| LINC01608 | 5.54 | 4.11 | 2.58E-14 | 8.57E-14 |
| RP11-705O24.1 | 2.34 | 2.93 | 2.68E-14 | 8.90E-14 |
| RP11-14C10.5 | 2.27 | 2.83 | 2.71E-14 | 8.97E-14 |
| FGF12-AS3 | 2.55 | 3.01 | 2.81E-14 | 9.28E-14 |
| LINC01192 | 3.52 | 3.16 | 3.05E-14 | 1.01E-13 |
| RP4-784A16.4 | 2.24 | 2.73 | 3.15E-14 | 1.04E-13 |
| RP11-400D2.2 | 4.67 | 3.27 | 3.16E-14 | 1.04E-13 |
| AC006145.4 | 3.93 | 3.49 | 3.29E-14 | 1.08E-13 |
| RP11-230G5.2 | 3.23 | 6.23 | 3.32E-14 | 1.09E-13 |
| RP11-173C1.1 | 2.04 | 6.15 | 3.45E-14 | 1.13E-13 |
| LINC01345 | 4.85 | 3.65 | 3.69E-14 | 1.21E-13 |
| RP11-552M14.1 | 3.44 | 2.72 | 3.78E-14 | 1.24E-13 |
| RP1-290I10.5 | 3.34 | 3.08 | 3.84E-14 | 1.26E-13 |
| ST8SIA6-AS1 | 3.17 | 7.01 | 3.86E-14 | 1.26E-13 |
| GS1-72M22.1 | 3.05 | 4.68 | 3.94E-14 | 1.29E-13 |
| RP11-269F21.2 | 3.54 | 2.91 | 3.95E-14 | 1.29E-13 |
| LINC00705 | 2.40 | 3.38 | 4.06E-14 | 1.33E-13 |
| RP11-259O2.3 | 2.86 | 3.01 | 4.08E-14 | 1.33E-13 |
| LINC01731 | 2.53 | 3.11 | 4.15E-14 | 1.36E-13 |
| RP11-337A23.6 | 4.09 | 2.99 | 4.30E-14 | 1.40E-13 |
| RP11-3B12.2 | 2.44 | 3.51 | 4.72E-14 | 1.54E-13 |
| RP11-496N12.6 | 2.33 | 4.91 | 4.77E-14 | 1.55E-13 |
| RP11-865I6.2 | 3.05 | 4.35 | 4.88E-14 | 1.58E-13 |
| RP11-474D1.4 | 5.15 | 3.60 | 4.97E-14 | 1.61E-13 |
| CTD-2147F2.2 | 2.53 | 3.30 | 5.02E-14 | 1.63E-13 |
| AE000661.50 | 2.12 | 3.33 | 5.39E-14 | 1.74E-13 |
| RP11-430H10.3 | 3.58 | 2.95 | 5.47E-14 | 1.77E-13 |
| RP11-115C10.1 | 2.61 | 3.99 | 5.74E-14 | 1.85E-13 |
| LINC01510 | 2.85 | 3.59 | 6.23E-14 | 2.00E-13 |
| RP11-52L5.6 | 2.92 | 2.71 | 6.42E-14 | 2.06E-13 |
| RP4-753M9.1 | 2.92 | 2.61 | 6.56E-14 | 2.11E-13 |
| LINC00251 | 3.27 | 2.56 | 7.13E-14 | 2.28E-13 |
| LINC01021 | 3.10 | 5.56 | 7.19E-14 | 2.30E-13 |
| AC016710.1 | 4.76 | 3.30 | 7.56E-14 | 2.41E-13 |
| CTD-2384A14.1 | 4.65 | 4.53 | 7.66E-14 | 2.44E-13 |
| AC012363.4 | 3.99 | 3.20 | 8.12E-14 | 2.59E-13 |
| RP11-323H21.3 | 5.96 | 5.04 | 8.17E-14 | 2.60E-13 |
| RP11-80F22.15 | 3.92 | 2.80 | 8.35E-14 | 2.65E-13 |
| AC092625.1 | 3.26 | 2.55 | 8.47E-14 | 2.69E-13 |
| CTD-2210P24.3 | 2.71 | 2.59 | 8.50E-14 | 2.70E-13 |
| RP11-527D7.1 | 2.91 | 3.77 | 8.70E-14 | 2.76E-13 |
| RP11-282A11.3 | 2.77 | 4.48 | 8.87E-14 | 2.81E-13 |
| RP11-184I16.4 | 2.78 | 4.08 | 9.18E-14 | 2.91E-13 |
| LINC02196 | 3.39 | 2.98 | 9.30E-14 | 2.94E-13 |
| RP11-416N4.4 | 2.36 | 2.60 | 9.38E-14 | 2.97E-13 |
| LINC01964 | 3.60 | 3.63 | 9.70E-14 | 3.06E-13 |
| RP11-624C23.1 | 2.01 | 4.29 | 1.00E-13 | 3.17E-13 |
| AC093627.7 | 3.11 | 3.08 | 1.08E-13 | 3.40E-13 |
| RP11-380D23.1 | 3.38 | 3.15 | 1.17E-13 | 3.69E-13 |
| RP11-324L17.1 | 2.33 | 3.73 | 1.19E-13 | 3.74E-13 |
| AF003626.1 | 5.34 | 4.18 | 1.19E-13 | 3.74E-13 |
| RP11-488I20.9 | 5.38 | 4.71 | 1.22E-13 | 3.83E-13 |
| MIR3681HG | 2.04 | 4.73 | 1.23E-13 | 3.86E-13 |
| RP11-89M20.2 | 3.82 | 2.75 | 1.27E-13 | 3.98E-13 |
| RP5-1185I7.1 | 2.09 | 6.93 | 1.42E-13 | 4.43E-13 |
| LINC00557 | 2.90 | 3.13 | 1.46E-13 | 4.56E-13 |
| AC079135.1 | 2.91 | 2.66 | 1.57E-13 | 4.90E-13 |
| SC22CB-56B3.1 | 4.55 | 3.19 | 1.57E-13 | 4.90E-13 |
| LINC01681 | 3.11 | 2.91 | 1.61E-13 | 5.01E-13 |
| LINC01776 | 3.33 | 3.17 | 1.71E-13 | 5.32E-13 |
| LINC01036 | 3.06 | 3.43 | 1.79E-13 | 5.58E-13 |
| LINC01087 | 3.25 | 3.74 | 1.94E-13 | 6.01E-13 |
| RP11-319F12.2 | 4.29 | 3.45 | 2.01E-13 | 6.23E-13 |
| RP11-435D7.3 | 2.17 | 2.64 | 2.04E-13 | 6.31E-13 |
| RP11-734I18.1 | 3.87 | 5.14 | 2.05E-13 | 6.36E-13 |
| RP11-406A9.2 | 3.56 | 4.10 | 2.08E-13 | 6.43E-13 |
| RP11-74M11.2 | -2.03 | 2.80 | 2.23E-13 | 6.92E-13 |
| AC084193.1 | 4.18 | 2.93 | 2.26E-13 | 7.01E-13 |
| RP11-15M15.1 | 2.61 | 2.62 | 2.63E-13 | 8.12E-13 |
| RP11-445P19.3 | 3.00 | 2.69 | 2.69E-13 | 8.30E-13 |
| RP5-916L7.2 | 2.61 | 2.86 | 3.06E-13 | 9.39E-13 |
| RP11-476M19.2 | 2.58 | 3.30 | 3.08E-13 | 9.47E-13 |
| RP11-330A16.1 | 2.08 | 3.70 | 3.21E-13 | 9.83E-13 |
| RP11-407A16.3 | 3.46 | 3.28 | 3.33E-13 | 1.02E-12 |
| RP11-805L22.3 | 3.16 | 2.52 | 3.38E-13 | 1.03E-12 |
| CTC-255N20.1 | 2.20 | 6.07 | 3.53E-13 | 1.07E-12 |
| B3GALT5-AS1 | 2.60 | 6.08 | 3.59E-13 | 1.09E-12 |
| RP1-32I10.10 | 2.67 | 3.03 | 3.60E-13 | 1.10E-12 |
| RP11-227D2.3 | 3.42 | 2.58 | 3.75E-13 | 1.14E-12 |
| LINC01139 | 2.23 | 8.00 | 3.88E-13 | 1.18E-12 |
| RP6-24A23.3 | 2.96 | 5.84 | 4.02E-13 | 1.22E-12 |
| AL773572.7 | 3.56 | 4.89 | 4.04E-13 | 1.22E-12 |
| RP11-1263C18.1 | 3.54 | 2.80 | 4.09E-13 | 1.24E-12 |
| RP11-804N13.1 | 3.12 | 3.78 | 4.10E-13 | 1.24E-12 |
| CTD-2314G24.2 | 2.41 | 5.59 | 4.34E-13 | 1.31E-12 |
| CTC-340I23.2 | 2.09 | 2.85 | 4.34E-13 | 1.31E-12 |
| RP11-831A10.2 | 3.75 | 3.16 | 4.39E-13 | 1.32E-12 |
| AC010745.2 | 3.27 | 2.62 | 4.81E-13 | 1.45E-12 |
| CTD-2587H24.5 | 2.17 | 3.48 | 4.88E-13 | 1.47E-12 |
| LINC01885 | 3.43 | 2.59 | 4.93E-13 | 1.48E-12 |
| AC016730.1 | 3.88 | 2.89 | 5.20E-13 | 1.56E-12 |
| RP3-326I13.1 | 4.03 | 3.31 | 5.26E-13 | 1.58E-12 |
| CTA-520D8.2 | 3.58 | 3.85 | 5.51E-13 | 1.65E-12 |
| CTB-1I21.1 | 3.53 | 3.36 | 5.76E-13 | 1.72E-12 |
| CTC-458G6.4 | 4.49 | 3.40 | 5.77E-13 | 1.72E-12 |
| LINC01411 | 2.90 | 2.97 | 5.86E-13 | 1.75E-12 |
| RP11-396O20.2 | 3.28 | 3.53 | 5.90E-13 | 1.76E-12 |
| RP11-503C24.4 | 2.60 | 3.55 | 5.96E-13 | 1.78E-12 |
| LINC01299 | 3.78 | 3.38 | 5.98E-13 | 1.78E-12 |
| UG0898H09 | 3.11 | 5.58 | 6.16E-13 | 1.83E-12 |
| RP11-718O11.1 | 2.31 | 2.78 | 6.41E-13 | 1.91E-12 |
| LINC00302 | 3.57 | 2.68 | 6.85E-13 | 2.04E-12 |
| OSTM1-AS1 | 3.46 | 3.25 | 6.90E-13 | 2.05E-12 |
| GPR1-AS | 4.26 | 4.67 | 7.21E-13 | 2.14E-12 |
| C11orf44 | 2.42 | 3.20 | 7.29E-13 | 2.16E-12 |
| RP4-555L14.4 | 2.35 | 3.30 | 7.82E-13 | 2.31E-12 |
| RP11-115I9.1 | 3.50 | 2.62 | 7.99E-13 | 2.36E-12 |
| RP11-676J15.1 | 2.36 | 2.56 | 8.26E-13 | 2.44E-12 |
| RP11-374A4.1 | 2.17 | 2.86 | 8.31E-13 | 2.46E-12 |
| KCNIP4-IT1 | 2.87 | 2.50 | 8.76E-13 | 2.59E-12 |
| EGLN3-AS1 | 2.79 | 2.72 | 8.91E-13 | 2.63E-12 |
| RP11-438B23.2 | 2.42 | 4.52 | 9.88E-13 | 2.91E-12 |
| RP11-1081M5.1 | 2.89 | 4.33 | 9.92E-13 | 2.92E-12 |
| LINC01193 | 4.53 | 3.34 | 9.99E-13 | 2.94E-12 |
| AC000032.2 | 3.09 | 3.25 | 1.02E-12 | 3.01E-12 |
| RP11-66B24.1 | 2.59 | 3.15 | 1.03E-12 | 3.04E-12 |
| RP11-110H1.8 | 3.56 | 3.68 | 1.05E-12 | 3.09E-12 |
| RP11-268G12.3 | 2.73 | 3.53 | 1.06E-12 | 3.10E-12 |
| LINC01701 | 2.26 | 2.81 | 1.08E-12 | 3.16E-12 |
| RP1-90K10.4 | 2.79 | 2.84 | 1.10E-12 | 3.22E-12 |
| AC010967.2 | 3.28 | 2.94 | 1.13E-12 | 3.30E-12 |
| LNX1-AS1 | 2.54 | 2.80 | 1.16E-12 | 3.38E-12 |
| C15orf59-AS1 | 2.59 | 4.53 | 1.24E-12 | 3.63E-12 |
| AL078471.5 | 4.13 | 2.98 | 1.29E-12 | 3.77E-12 |
| AP000998.2 | 2.65 | 2.49 | 1.31E-12 | 3.82E-12 |
| CTD-2210P24.2 | 3.73 | 2.80 | 1.34E-12 | 3.92E-12 |
| CTB-147C22.9 | 2.72 | 2.86 | 1.34E-12 | 3.92E-12 |
| LINC01924 | 4.13 | 3.27 | 1.35E-12 | 3.94E-12 |
| RP11-749H20.1 | 3.34 | 2.65 | 1.44E-12 | 4.19E-12 |
| AC114730.5 | 2.04 | 3.29 | 1.45E-12 | 4.20E-12 |
| RP11-315A17.1 | 5.33 | 4.88 | 1.45E-12 | 4.22E-12 |
| PAQR9-AS1 | 3.01 | 3.52 | 1.46E-12 | 4.25E-12 |
| OVAAL | 2.99 | 4.16 | 1.51E-12 | 4.38E-12 |
| RP11-315F22.1 | 4.98 | 3.75 | 1.64E-12 | 4.75E-12 |
| RP11-809H16.5 | 2.97 | 2.70 | 1.67E-12 | 4.82E-12 |
| RP1-154K9.2 | 2.22 | 4.61 | 1.70E-12 | 4.90E-12 |
| RP1-261G23.5 | 2.56 | 2.95 | 1.77E-12 | 5.12E-12 |
| RP11-392O17.1 | 2.85 | 4.78 | 1.85E-12 | 5.34E-12 |
| RP11-348J12.5 | 2.21 | 2.90 | 1.86E-12 | 5.35E-12 |
| LINC01257 | 3.98 | 4.64 | 1.88E-12 | 5.43E-12 |
| AC142293.3 | 2.98 | 3.66 | 1.99E-12 | 5.71E-12 |
| AC002463.3 | 3.75 | 3.08 | 2.16E-12 | 6.20E-12 |
| RP11-95P13.2 | 4.30 | 3.14 | 2.36E-12 | 6.76E-12 |
| LINC01405 | 3.14 | 2.88 | 2.37E-12 | 6.78E-12 |
| RP11-290F24.3 | 2.09 | 3.11 | 2.41E-12 | 6.90E-12 |
| MIR4300HG | 2.95 | 3.14 | 2.41E-12 | 6.91E-12 |
| LINC01475 | 3.33 | 3.27 | 2.47E-12 | 7.08E-12 |
| RP11-809H16.4 | 2.99 | 2.64 | 2.48E-12 | 7.11E-12 |
| TUSC8 | 3.40 | 5.42 | 2.51E-12 | 7.19E-12 |
| AC073284.4 | 2.50 | 2.55 | 2.60E-12 | 7.42E-12 |
| AP000439.1 | 2.87 | 4.18 | 2.61E-12 | 7.47E-12 |
| RP11-120I21.2 | 3.85 | 3.16 | 2.63E-12 | 7.51E-12 |
| RP11-503C24.1 | 3.82 | 3.37 | 2.66E-12 | 7.61E-12 |
| LINC01343 | 2.94 | 2.94 | 3.31E-12 | 9.39E-12 |
| IGF2-AS | 2.50 | 4.66 | 3.44E-12 | 9.76E-12 |
| LINC01740 | 2.90 | 4.33 | 3.55E-12 | 1.01E-11 |
| C10orf126 | 4.19 | 3.98 | 3.64E-12 | 1.03E-11 |
| RP11-122C21.1 | 3.64 | 3.57 | 3.84E-12 | 1.08E-11 |
| LINC01037 | 2.94 | 3.06 | 3.88E-12 | 1.10E-11 |
| CPS1-IT1 | 3.84 | 2.81 | 4.26E-12 | 1.20E-11 |
| LINC01162 | 3.37 | 2.65 | 4.31E-12 | 1.21E-11 |
| AC006262.10 | 3.08 | 2.85 | 4.32E-12 | 1.22E-11 |
| LINC01749 | 2.27 | 2.60 | 4.42E-12 | 1.24E-11 |
| RP3-446N13.5 | 3.55 | 2.95 | 4.43E-12 | 1.24E-11 |
| RP11-631F7.1 | 2.50 | 2.97 | 4.57E-12 | 1.28E-11 |
| KIRREL3-AS1 | 3.17 | 3.23 | 4.67E-12 | 1.31E-11 |
| RP11-184M15.2 | 2.33 | 3.23 | 4.94E-12 | 1.38E-11 |
| AC104389.28 | 2.66 | 5.14 | 4.95E-12 | 1.39E-11 |
| RP11-482M8.3 | 3.26 | 2.82 | 5.19E-12 | 1.45E-11 |
| LINC01647 | 3.21 | 3.08 | 5.24E-12 | 1.46E-11 |
| CTD-3007L5.1 | 4.76 | 3.39 | 5.76E-12 | 1.61E-11 |
| LINC01470 | 2.42 | 2.79 | 5.81E-12 | 1.62E-11 |
| LINC01346 | 4.67 | 4.37 | 6.45E-12 | 1.79E-11 |
| AC006372.5 | 3.05 | 3.25 | 7.07E-12 | 1.96E-11 |
| AC005808.3 | 2.34 | 2.73 | 7.21E-12 | 1.99E-11 |
| RMRP | 4.80 | 5.47 | 8.48E-12 | 2.34E-11 |
| RP11-13K12.5 | 2.15 | 5.82 | 9.72E-12 | 2.67E-11 |
| LINC01205 | 3.68 | 3.65 | 9.99E-12 | 2.74E-11 |
| CTD-2315E11.1 | 2.42 | 3.20 | 1.08E-11 | 2.95E-11 |
| PCAT5 | 2.44 | 3.01 | 1.08E-11 | 2.96E-11 |
| RP11-302L19.1 | 3.01 | 3.19 | 1.10E-11 | 3.00E-11 |
| RP11-170M17.1 | 2.34 | 2.90 | 1.14E-11 | 3.11E-11 |
| RP3-438O4.4 | 3.34 | 3.51 | 1.19E-11 | 3.26E-11 |
| NRG1-IT1 | 3.10 | 2.59 | 1.24E-11 | 3.39E-11 |
| RP5-912I13.1 | 3.82 | 3.26 | 1.31E-11 | 3.56E-11 |
| GS1-594A7.3 | 2.06 | 3.73 | 1.32E-11 | 3.60E-11 |
| RP5-1011O1.3 | 2.03 | 2.92 | 1.34E-11 | 3.64E-11 |
| LINC01819 | 2.72 | 6.50 | 1.56E-11 | 4.21E-11 |
| RP11-244B22.11 | 4.84 | 3.38 | 1.58E-11 | 4.28E-11 |
| RP11-11N5.3 | 2.81 | 2.81 | 1.60E-11 | 4.32E-11 |
| RP11-462P6.1 | 2.05 | 2.61 | 1.83E-11 | 4.94E-11 |
| AC006262.4 | 2.31 | 3.24 | 1.96E-11 | 5.27E-11 |
| LINC00706 | 2.02 | 3.28 | 2.02E-11 | 5.42E-11 |
| RP11-664H17.1 | 2.32 | 4.06 | 2.03E-11 | 5.46E-11 |
| CTD-2311B13.1 | 3.08 | 2.46 | 2.06E-11 | 5.53E-11 |
| AC060834.3 | 4.20 | 3.46 | 2.09E-11 | 5.61E-11 |
| DIO2-AS1 | 2.63 | 2.63 | 2.10E-11 | 5.62E-11 |
| AL162759.1 | 2.27 | 4.09 | 2.14E-11 | 5.75E-11 |
| RP11-123K19.1 | 2.42 | 3.46 | 2.21E-11 | 5.93E-11 |
| RP11-315E17.1 | 3.66 | 3.36 | 2.30E-11 | 6.15E-11 |
| RP11-702B10.2 | 2.38 | 4.39 | 2.50E-11 | 6.68E-11 |
| RP11-344E13.4 | 3.05 | 3.34 | 2.54E-11 | 6.79E-11 |
| RP11-722P11.4 | 2.16 | 3.14 | 2.55E-11 | 6.81E-11 |
| RP11-1114I9.1 | 3.19 | 2.66 | 2.78E-11 | 7.40E-11 |
| CTD-2540B15.6 | 2.23 | 2.68 | 2.91E-11 | 7.75E-11 |
| RP11-462G2.1 | 2.45 | 8.31 | 2.92E-11 | 7.76E-11 |
| AC003088.1 | 2.02 | 3.14 | 3.05E-11 | 8.10E-11 |
| RP11-334A14.8 | 2.13 | 5.26 | 3.17E-11 | 8.40E-11 |
| RP11-1145L24.1 | 3.34 | 2.89 | 3.18E-11 | 8.42E-11 |
| RP11-774I5.1 | 3.35 | 2.62 | 3.24E-11 | 8.58E-11 |
| RP11-665I14.1 | 4.32 | 3.05 | 3.32E-11 | 8.78E-11 |
| RP11-578B16.1 | 3.00 | 3.01 | 3.35E-11 | 8.86E-11 |
| RP6-91H8.5 | 2.23 | 2.96 | 3.45E-11 | 9.11E-11 |
| ANO1-AS2 | 2.28 | 3.24 | 3.48E-11 | 9.20E-11 |
| RP11-897M7.4 | 3.03 | 2.85 | 3.50E-11 | 9.23E-11 |
| RP13-895J2.3 | 2.49 | 3.87 | 3.50E-11 | 9.24E-11 |
| LINC01666 | 2.22 | 3.53 | 3.51E-11 | 9.27E-11 |
| AC064834.1 | 4.77 | 4.93 | 3.53E-11 | 9.30E-11 |
| LINC00408 | 3.54 | 2.63 | 3.53E-11 | 9.31E-11 |
| CTC-241F20.4 | 2.02 | 3.30 | 3.60E-11 | 9.48E-11 |
| AC005307.1 | 3.26 | 3.72 | 3.73E-11 | 9.82E-11 |
| RP11-18A15.1 | 2.85 | 2.56 | 3.76E-11 | 9.90E-11 |
| AC093843.1 | 2.18 | 2.56 | 3.77E-11 | 9.91E-11 |
| LINC01326 | 3.67 | 2.78 | 3.96E-11 | 1.04E-10 |
| KIF25-AS1 | 2.35 | 6.74 | 4.10E-11 | 1.08E-10 |
| PCAT18 | 2.59 | 4.84 | 4.29E-11 | 1.12E-10 |
| MRGPRG-AS1 | 3.16 | 2.77 | 4.43E-11 | 1.16E-10 |
| CTA-992D9.6 | 4.82 | 3.58 | 4.58E-11 | 1.20E-10 |
| RP11-507B12.2 | 2.74 | 3.16 | 4.65E-11 | 1.22E-10 |
| RP11-486M23.2 | 2.18 | 2.81 | 4.67E-11 | 1.22E-10 |
| AC023481.1 | 2.02 | 2.64 | 4.74E-11 | 1.24E-10 |
| RP11-497G19.2 | 3.61 | 5.38 | 4.74E-11 | 1.24E-10 |
| LINC01976 | 2.43 | 2.72 | 5.20E-11 | 1.35E-10 |
| LINC02119 | 3.43 | 2.81 | 5.37E-11 | 1.40E-10 |
| RP11-2A4.4 | 4.36 | 3.96 | 5.56E-11 | 1.45E-10 |
| LINC02031 | 2.67 | 5.73 | 5.60E-11 | 1.46E-10 |
| RP11-394A14.2 | 2.43 | 2.52 | 5.62E-11 | 1.46E-10 |
| RP11-362K2.2 | 2.05 | 2.74 | 6.20E-11 | 1.61E-10 |
| LINC00403 | 3.83 | 3.55 | 6.54E-11 | 1.69E-10 |
| RP11-128P17.4 | 3.57 | 3.12 | 6.70E-11 | 1.73E-10 |
| RP11-11K13.1 | 3.41 | 2.58 | 6.78E-11 | 1.75E-10 |
| RP5-866L20.1 | 2.59 | 3.28 | 6.78E-11 | 1.75E-10 |
| RP11-231N3.1 | 2.22 | 2.95 | 6.94E-11 | 1.79E-10 |
| LINC00867 | 2.31 | 3.43 | 6.97E-11 | 1.80E-10 |
| RP11-449J1.1 | 2.63 | 2.98 | 7.14E-11 | 1.84E-10 |
| RP11-1085N6.3 | 2.83 | 2.71 | 7.18E-11 | 1.85E-10 |
| UPK1A-AS1 | 2.23 | 4.10 | 7.66E-11 | 1.97E-10 |
| U91319.1 | 3.34 | 3.15 | 7.85E-11 | 2.02E-10 |
| LINC01665 | 3.60 | 3.13 | 7.92E-11 | 2.04E-10 |
| RP11-503C24.2 | 3.10 | 3.67 | 8.62E-11 | 2.21E-10 |
| RP11-1041F24.1 | 3.78 | 2.87 | 8.99E-11 | 2.30E-10 |
| LINC01210 | 3.96 | 3.38 | 9.28E-11 | 2.38E-10 |
| RP11-435O5.6 | 2.16 | 3.18 | 9.48E-11 | 2.43E-10 |
| RP11-161D15.2 | 2.50 | 2.78 | 9.51E-11 | 2.43E-10 |
| C12orf77 | 2.52 | 3.47 | 9.57E-11 | 2.45E-10 |
| RP11-384F7.1 | 3.21 | 3.16 | 1.01E-10 | 2.58E-10 |
| CTD-2130O13.1 | 3.07 | 4.23 | 1.05E-10 | 2.68E-10 |
| RP11-619J20.1 | 2.22 | 2.51 | 1.06E-10 | 2.70E-10 |
| RP11-12K22.1 | 3.41 | 2.76 | 1.08E-10 | 2.75E-10 |
| CTD-2515C13.2 | 3.34 | 2.67 | 1.10E-10 | 2.81E-10 |
| LINC01831 | 4.21 | 2.91 | 1.17E-10 | 2.97E-10 |
| ATP11AUN | 2.95 | 5.13 | 1.19E-10 | 3.03E-10 |
| AP000997.2 | 2.89 | 2.83 | 1.23E-10 | 3.11E-10 |
| RP11-479O16.1 | 2.76 | 4.15 | 1.23E-10 | 3.12E-10 |
| RP11-555G19.1 | 3.67 | 3.39 | 1.23E-10 | 3.12E-10 |
| AC002511.2 | 2.11 | 4.24 | 1.24E-10 | 3.13E-10 |
| RP11-115J23.1 | 2.67 | 3.24 | 1.25E-10 | 3.18E-10 |
| LINC02192 | 2.48 | 2.81 | 1.27E-10 | 3.21E-10 |
| RP11-776A13.3 | 3.50 | 2.66 | 1.29E-10 | 3.26E-10 |
| LINC02046 | 3.33 | 3.19 | 1.32E-10 | 3.33E-10 |
| LINC00052 | 2.80 | 2.66 | 1.35E-10 | 3.42E-10 |
| TTTY20 | 4.60 | 3.17 | 1.46E-10 | 3.69E-10 |
| RP11-172F10.1 | 2.24 | 2.84 | 1.46E-10 | 3.69E-10 |
| LINC01574 | 2.57 | 2.77 | 1.48E-10 | 3.74E-10 |
| RP5-1139I1.1 | 2.46 | 3.26 | 1.52E-10 | 3.84E-10 |
| RP11-157E21.1 | 2.19 | 5.09 | 1.54E-10 | 3.89E-10 |
| AP000473.6 | 2.30 | 2.52 | 1.55E-10 | 3.89E-10 |
| RP5-827E24.1 | 2.57 | 3.08 | 1.56E-10 | 3.93E-10 |
| RP11-148E17.1 | 3.73 | 3.47 | 1.67E-10 | 4.20E-10 |
| RP11-206M11.7 | 3.74 | 4.82 | 1.70E-10 | 4.28E-10 |
| LINC01254 | 2.81 | 3.40 | 1.76E-10 | 4.42E-10 |
| RP11-554D15.3 | 2.46 | 3.09 | 1.77E-10 | 4.44E-10 |
| RP11-438D14.3 | 2.78 | 2.75 | 1.77E-10 | 4.44E-10 |
| LINC00488 | 3.47 | 3.24 | 1.80E-10 | 4.49E-10 |
| LINC01491 | 3.10 | 2.75 | 1.82E-10 | 4.54E-10 |
| AC108868.6 | 4.46 | 3.57 | 1.93E-10 | 4.81E-10 |
| RP11-193H5.1 | 3.34 | 2.86 | 2.02E-10 | 5.04E-10 |
| LINC01258 | 2.08 | 3.09 | 2.09E-10 | 5.22E-10 |
| PSG8-AS1 | 2.45 | 2.50 | 2.16E-10 | 5.38E-10 |
| AC007126.1 | 2.67 | 2.87 | 2.45E-10 | 6.09E-10 |
| RP11-431J17.1 | 4.09 | 2.93 | 2.48E-10 | 6.16E-10 |
| RP11-713M6.2 | 3.04 | 2.53 | 2.53E-10 | 6.29E-10 |
| LINC01179 | 2.75 | 3.14 | 2.63E-10 | 6.52E-10 |
| RP11-408N14.1 | 2.39 | 2.63 | 2.63E-10 | 6.52E-10 |
| AC012354.6 | 2.11 | 3.50 | 2.67E-10 | 6.62E-10 |
| RP11-875H7.5 | 3.34 | 5.33 | 2.72E-10 | 6.74E-10 |
| RP11-966I7.2 | 3.38 | 2.66 | 2.99E-10 | 7.39E-10 |
| RP11-124N3.2 | 3.79 | 2.84 | 3.00E-10 | 7.42E-10 |
| RP11-372H2.1 | 2.74 | 3.11 | 3.20E-10 | 7.89E-10 |
| ELOVL2-AS1 | 2.15 | 2.94 | 3.31E-10 | 8.15E-10 |
| RP11-53B5.1 | 3.04 | 4.88 | 3.32E-10 | 8.18E-10 |
| RP11-774D14.1 | 3.23 | 6.13 | 3.33E-10 | 8.18E-10 |
| AC021218.2 | 2.03 | 5.82 | 3.42E-10 | 8.42E-10 |
| AC004009.3 | 3.30 | 2.57 | 3.68E-10 | 9.03E-10 |
| LINC01312 | 2.68 | 3.04 | 4.21E-10 | 1.03E-09 |
| RP11-319E12.2 | 2.44 | 2.58 | 4.47E-10 | 1.09E-09 |
| RP11-19O2.2 | 3.06 | 2.58 | 4.57E-10 | 1.11E-09 |
| AC018685.1 | 2.53 | 2.68 | 4.60E-10 | 1.12E-09 |
| AC012506.2 | 2.55 | 2.57 | 4.71E-10 | 1.15E-09 |
| RP11-788H18.1 | 3.32 | 2.71 | 4.80E-10 | 1.17E-09 |
| RP11-998D10.4 | 3.08 | 2.73 | 4.80E-10 | 1.17E-09 |
| RP11-149B9.2 | 4.42 | 3.40 | 5.02E-10 | 1.22E-09 |
| RP11-648K4.2 | 2.99 | 2.54 | 5.07E-10 | 1.23E-09 |
| RP11-31K23.2 | 3.41 | 2.79 | 5.35E-10 | 1.30E-09 |
| RP11-124N3.3 | 3.97 | 3.04 | 5.56E-10 | 1.35E-09 |
| MIR7-3HG | 3.15 | 3.93 | 5.69E-10 | 1.38E-09 |
| LINC01425 | 3.76 | 3.17 | 5.72E-10 | 1.38E-09 |
| RP11-445N18.5 | 2.19 | 4.28 | 6.19E-10 | 1.49E-09 |
| TCF4-AS1 | 2.41 | 4.13 | 6.54E-10 | 1.58E-09 |
| RP11-184D12.1 | 3.49 | 2.89 | 7.15E-10 | 1.72E-09 |
| RP3-400B16.3 | 2.97 | 2.96 | 7.72E-10 | 1.85E-09 |
| LINC01886 | 3.47 | 3.82 | 7.80E-10 | 1.87E-09 |
| AC008063.3 | 2.09 | 2.84 | 7.94E-10 | 1.91E-09 |
| RP11-44K6.3 | 2.02 | 3.26 | 7.99E-10 | 1.92E-09 |
| RP11-65M17.1 | 2.18 | 2.60 | 8.32E-10 | 1.99E-09 |
| LINC02037 | 3.08 | 4.24 | 8.56E-10 | 2.05E-09 |
| AP000997.1 | 2.87 | 2.61 | 8.60E-10 | 2.06E-09 |
| RP11-117L5.4 | 2.41 | 2.61 | 9.44E-10 | 2.25E-09 |
| LINC01602 | 2.89 | 5.47 | 9.70E-10 | 2.31E-09 |
| RP11-775H9.3 | 4.88 | 4.66 | 1.02E-09 | 2.42E-09 |
| LINC02005 | 2.39 | 3.59 | 1.03E-09 | 2.44E-09 |
| RP11-614F17.2 | 3.15 | 3.24 | 1.03E-09 | 2.45E-09 |
| RP11-498B4.5 | 2.46 | 2.52 | 1.06E-09 | 2.52E-09 |
| SC22CB-1D7.1 | 2.35 | 3.13 | 1.06E-09 | 2.52E-09 |
| RP11-560A15.3 | 2.57 | 2.66 | 1.08E-09 | 2.57E-09 |
| DANT1 | 3.01 | 2.83 | 1.09E-09 | 2.57E-09 |
| LINC01551 | 3.44 | 3.27 | 1.18E-09 | 2.79E-09 |
| RP11-536K17.1 | 3.34 | 2.61 | 1.27E-09 | 2.99E-09 |
| RP11-148B3.2 | 2.87 | 5.26 | 1.31E-09 | 3.08E-09 |
| AC007091.1 | 2.66 | 2.57 | 1.44E-09 | 3.37E-09 |
| AC006380.3 | 3.72 | 3.26 | 1.48E-09 | 3.48E-09 |
| LINC01467 | 3.92 | 3.02 | 1.58E-09 | 3.70E-09 |
| RP11-141A19.1 | 2.40 | 2.51 | 1.74E-09 | 4.08E-09 |
| RP1-276N6.2 | 3.69 | 2.82 | 1.93E-09 | 4.50E-09 |
| RP13-895J2.6 | 2.88 | 3.64 | 1.95E-09 | 4.53E-09 |
| RP1-209A6.1 | 2.09 | 3.36 | 1.99E-09 | 4.64E-09 |
| RP11-627G23.1 | 2.15 | 8.53 | 2.07E-09 | 4.81E-09 |
| AC005150.1 | 3.20 | 2.78 | 2.09E-09 | 4.86E-09 |
| AC005307.4 | 2.47 | 4.52 | 2.18E-09 | 5.05E-09 |
| RP11-626P14.1 | 2.50 | 2.53 | 2.33E-09 | 5.39E-09 |
| LINC00661 | 2.83 | 2.89 | 2.39E-09 | 5.53E-09 |
| AC099342.1 | 2.06 | 2.66 | 2.39E-09 | 5.54E-09 |
| RP11-554A11.8 | 3.12 | 3.97 | 2.41E-09 | 5.56E-09 |
| GACAT3 | 3.53 | 3.42 | 2.46E-09 | 5.70E-09 |
| LINC00602 | 2.99 | 3.21 | 2.64E-09 | 6.08E-09 |
| RP11-307P5.1 | 2.45 | 4.18 | 2.81E-09 | 6.46E-09 |
| RP11-702F3.3 | 2.60 | 2.88 | 2.86E-09 | 6.57E-09 |
| PRKCA-AS1 | 2.53 | 3.62 | 3.00E-09 | 6.89E-09 |
| CTD-2552K11.2 | 2.17 | 2.54 | 3.23E-09 | 7.39E-09 |
| RP11-475A13.1 | 3.16 | 3.03 | 3.52E-09 | 8.03E-09 |
| LINC00404 | 3.34 | 3.12 | 3.56E-09 | 8.13E-09 |
| RP11-161D15.1 | 2.25 | 2.88 | 3.82E-09 | 8.69E-09 |
| LINC01788 | 2.50 | 2.60 | 3.90E-09 | 8.88E-09 |
| AP000688.29 | 2.14 | 5.44 | 4.19E-09 | 9.51E-09 |
| LINC01029 | 4.03 | 3.49 | 4.38E-09 | 9.93E-09 |
| RP5-944M2.3 | 2.09 | 3.31 | 4.45E-09 | 1.01E-08 |
| RP11-324D17.2 | 2.10 | 2.68 | 4.58E-09 | 1.04E-08 |
| MIR124-2HG | 3.00 | 2.48 | 4.62E-09 | 1.05E-08 |
| RP11-385M4.1 | 2.43 | 2.63 | 4.86E-09 | 1.10E-08 |
| RP11-280G9.1 | 2.27 | 3.79 | 4.91E-09 | 1.11E-08 |
| RP11-863K10.2 | 2.57 | 2.91 | 5.02E-09 | 1.13E-08 |
| EPHA5-AS1 | 2.63 | 3.47 | 5.07E-09 | 1.14E-08 |
| LINC02008 | 3.23 | 2.91 | 5.10E-09 | 1.15E-08 |
| RP5-1024C24.1 | 2.36 | 3.36 | 5.16E-09 | 1.16E-08 |
| ADARB2-AS1 | 2.11 | 3.80 | 5.33E-09 | 1.20E-08 |
| RP11-204E9.1 | 2.87 | 3.39 | 6.46E-09 | 1.45E-08 |
| AC019185.4 | 2.03 | 2.51 | 6.62E-09 | 1.48E-08 |
| PLCB1-IT1 | 2.75 | 2.38 | 6.63E-09 | 1.48E-08 |
| CTC-339O9.1 | 2.05 | 4.11 | 6.83E-09 | 1.53E-08 |
| DPYD-AS1 | 2.03 | 6.03 | 7.02E-09 | 1.57E-08 |
| RP1-135L22.1 | 3.76 | 2.98 | 7.09E-09 | 1.58E-08 |
| RP11-685A21.1 | 2.56 | 2.58 | 7.58E-09 | 1.69E-08 |
| RP11-162D9.3 | 2.39 | 3.28 | 7.75E-09 | 1.72E-08 |
| RP11-369C8.1 | 3.31 | 3.50 | 7.84E-09 | 1.74E-08 |
| RP1-200K18.1 | 2.73 | 2.55 | 7.92E-09 | 1.76E-08 |
| RP11-171N4.1 | 3.37 | 2.78 | 8.50E-09 | 1.88E-08 |
| LINC00485 | 2.03 | 2.79 | 8.58E-09 | 1.90E-08 |
| RP11-300M24.1 | 2.82 | 2.71 | 8.77E-09 | 1.94E-08 |
| RP11-108E14.1 | 3.84 | 2.82 | 9.13E-09 | 2.02E-08 |
| RP11-794A8.1 | 3.92 | 3.86 | 9.87E-09 | 2.18E-08 |
| RP11-420B22.1 | 2.17 | 2.65 | 9.97E-09 | 2.20E-08 |
| RP11-1C1.6 | 3.44 | 2.96 | 1.02E-08 | 2.25E-08 |
| XXyac-YR29IB3.1 | 2.61 | 2.57 | 1.28E-08 | 2.80E-08 |
| AP000439.3 | 2.71 | 4.21 | 1.28E-08 | 2.81E-08 |
| LINC00879 | 3.45 | 2.81 | 1.35E-08 | 2.95E-08 |
| RP11-526P6.1 | 2.67 | 2.35 | 1.38E-08 | 3.00E-08 |
| RP11-510C10.3 | 3.09 | 2.83 | 1.45E-08 | 3.16E-08 |
| RP11-384J4.2 | 2.18 | 3.18 | 1.56E-08 | 3.38E-08 |
| RP4-655C5.4 | 2.10 | 3.64 | 1.56E-08 | 3.38E-08 |
| NAALADL2-AS3 | 2.40 | 2.50 | 1.63E-08 | 3.54E-08 |
| LINC01850 | 2.10 | 2.66 | 1.67E-08 | 3.62E-08 |
| KB-68A7.2 | 2.17 | 3.82 | 1.74E-08 | 3.78E-08 |
| RP11-536I6.2 | 2.49 | 3.03 | 1.81E-08 | 3.90E-08 |
| LINC00836 | 3.15 | 2.71 | 2.27E-08 | 4.87E-08 |
| LINC01159 | 2.97 | 2.77 | 2.44E-08 | 5.23E-08 |
| CTD-2385L22.1 | 2.15 | 3.33 | 2.65E-08 | 5.67E-08 |
| RP11-643A5.2 | 3.21 | 2.70 | 2.88E-08 | 6.13E-08 |
| LINC01998 | 2.52 | 3.63 | 2.94E-08 | 6.25E-08 |
| RP11-61O11.1 | 3.48 | 3.19 | 2.96E-08 | 6.30E-08 |
| LINC00303 | 2.22 | 3.57 | 2.97E-08 | 6.32E-08 |
| AC116609.2 | 2.16 | 3.25 | 3.29E-08 | 6.99E-08 |
| RP11-760D2.5 | 2.54 | 3.81 | 3.46E-08 | 7.33E-08 |
| AC007731.1 | 2.31 | 2.71 | 3.58E-08 | 7.56E-08 |
| LINC00456 | 2.37 | 2.49 | 3.69E-08 | 7.79E-08 |
| CH507-528H12.1 | 2.03 | 2.94 | 3.71E-08 | 7.82E-08 |
| RP11-318G21.4 | 3.28 | 3.53 | 4.12E-08 | 8.66E-08 |
| LINC01502 | 2.15 | 4.91 | 4.15E-08 | 8.73E-08 |
| RP11-161I2.1 | 2.50 | 3.24 | 4.24E-08 | 8.89E-08 |
| LINC02055 | 2.21 | 5.17 | 4.99E-08 | 1.04E-07 |
| AC006019.3 | 3.32 | 2.84 | 5.11E-08 | 1.07E-07 |
| RP11-34C15.2 | 3.68 | 2.89 | 5.23E-08 | 1.09E-07 |
| RP11-88H10.2 | 2.56 | 3.13 | 5.25E-08 | 1.09E-07 |
| RP11-305B6.3 | 2.72 | 2.68 | 6.32E-08 | 1.31E-07 |
| MAPT-IT1 | 2.20 | 2.50 | 6.33E-08 | 1.31E-07 |
| LINC00581 | 2.44 | 3.02 | 6.41E-08 | 1.33E-07 |
| LINC00473 | 2.64 | 8.48 | 6.81E-08 | 1.41E-07 |
| RP11-1O10.1 | 2.18 | 3.12 | 6.97E-08 | 1.44E-07 |
| ZBTB46-AS1 | 2.12 | 3.27 | 7.03E-08 | 1.45E-07 |
| FER1L6-AS1 | 2.78 | 2.75 | 7.17E-08 | 1.48E-07 |
| CTD-3064C13.1 | 2.41 | 2.87 | 7.38E-08 | 1.52E-07 |
| RP1-140J1.1 | 2.16 | 3.85 | 7.96E-08 | 1.64E-07 |
| RP11-12K11.2 | 2.22 | 3.73 | 8.48E-08 | 1.74E-07 |
| AF178030.2 | 2.31 | 2.88 | 8.57E-08 | 1.76E-07 |
| PEX5L-AS1 | 3.67 | 4.18 | 8.68E-08 | 1.78E-07 |
| CTC-264K15.6 | 2.50 | 2.66 | 9.07E-08 | 1.85E-07 |
| LINC01079 | 2.75 | 3.46 | 1.00E-07 | 2.05E-07 |
| RP13-895J2.2 | 2.00 | 3.11 | 1.02E-07 | 2.08E-07 |
| AC091814.2 | 2.12 | 3.83 | 1.12E-07 | 2.27E-07 |
| LINC01228 | 2.60 | 3.93 | 1.21E-07 | 2.45E-07 |
| RP11-81K13.1 | 2.40 | 2.85 | 1.37E-07 | 2.78E-07 |
| RP4-704D23.1 | 2.07 | 2.63 | 1.62E-07 | 3.25E-07 |
| LINC00200 | 3.22 | 4.62 | 1.73E-07 | 3.46E-07 |
| LINC01444 | 2.88 | 3.48 | 1.82E-07 | 3.64E-07 |
| RP11-15G16.1 | 2.40 | 2.66 | 2.14E-07 | 4.26E-07 |
| CTD-2050N2.1 | 2.06 | 2.54 | 2.58E-07 | 5.11E-07 |
| RP11-433M22.2 | 2.31 | 3.71 | 2.61E-07 | 5.16E-07 |
| LINC01158 | 2.90 | 3.23 | 2.77E-07 | 5.48E-07 |
| AL132709.1 | 2.59 | 2.52 | 3.17E-07 | 6.23E-07 |
| RP11-142A22.4 | 2.03 | 4.10 | 3.26E-07 | 6.39E-07 |
| CTB-180C19.1 | 3.53 | 2.82 | 3.53E-07 | 6.89E-07 |
| LINC01320 | 2.25 | 4.31 | 3.54E-07 | 6.92E-07 |
| LINC02095 | 2.22 | 2.78 | 3.64E-07 | 7.11E-07 |
| RP1-310O13.13 | 2.44 | 2.96 | 3.75E-07 | 7.31E-07 |
| RP11-567C20.2 | 2.79 | 2.43 | 4.57E-07 | 8.85E-07 |
| RP11-2L8.2 | 2.99 | 2.81 | 4.60E-07 | 8.91E-07 |
| LINC01198 | 2.10 | 3.19 | 4.79E-07 | 9.27E-07 |
| RP11-818F20.4 | 2.45 | 3.27 | 6.27E-07 | 1.20E-06 |
| DSCR10 | 2.20 | 2.49 | 6.40E-07 | 1.23E-06 |
| RP11-731N10.1 | 2.67 | 2.61 | 6.99E-07 | 1.33E-06 |
| RP5-1119A7.17 | 2.64 | 2.81 | 7.52E-07 | 1.43E-06 |
| AC145123.2 | 2.43 | 2.56 | 7.64E-07 | 1.45E-06 |
| RP11-1105O14.1 | 2.24 | 3.45 | 8.07E-07 | 1.53E-06 |
| CH507-513H4.3 | 3.20 | 4.28 | 8.36E-07 | 1.58E-06 |
| C17orf77 | 2.13 | 2.85 | 9.83E-07 | 1.85E-06 |
| CTC-286N12.1 | 3.09 | 2.87 | 1.02E-06 | 1.92E-06 |
| RP11-52J3.3 | 2.37 | 2.87 | 1.03E-06 | 1.93E-06 |
| RP11-563N12.2 | 2.10 | 2.62 | 1.05E-06 | 1.96E-06 |
| RP11-21B23.3 | 2.73 | 2.40 | 1.11E-06 | 2.08E-06 |
| RP11-88H10.3 | 2.28 | 2.98 | 1.27E-06 | 2.37E-06 |
| RP11-70J12.1 | 2.42 | 2.99 | 1.31E-06 | 2.43E-06 |
| TUSC7 | 2.80 | 3.74 | 1.37E-06 | 2.55E-06 |
| CYMP-AS1 | 2.01 | 3.84 | 1.46E-06 | 2.70E-06 |
| RP11-10H3.1 | 2.33 | 3.98 | 1.47E-06 | 2.72E-06 |
| RP5-855F14.2 | 2.01 | 2.58 | 1.67E-06 | 3.08E-06 |
| RP11-569G13.3 | 2.09 | 4.59 | 1.74E-06 | 3.19E-06 |
| RP11-322J23.1 | 2.21 | 3.02 | 1.75E-06 | 3.22E-06 |
| RP11-366H4.1 | 2.94 | 3.34 | 1.86E-06 | 3.42E-06 |
| RP11-448G4.4 | 2.19 | 2.42 | 2.06E-06 | 3.78E-06 |
| RP11-704M14.1 | 2.32 | 3.17 | 2.14E-06 | 3.90E-06 |
| RP5-921G16.1 | 2.12 | 2.90 | 2.24E-06 | 4.09E-06 |
| RP11-527L4.6 | 2.17 | 3.21 | 2.25E-06 | 4.11E-06 |
| RP11-91J3.3 | 2.16 | 2.57 | 2.61E-06 | 4.74E-06 |
| LINC02050 | 2.26 | 2.92 | 2.93E-06 | 5.30E-06 |
| AC023347.1 | 2.43 | 3.02 | 3.08E-06 | 5.56E-06 |
| MIR3976HG | 2.64 | 2.50 | 3.37E-06 | 6.05E-06 |
| AC006050.2 | 2.68 | 3.66 | 3.41E-06 | 6.13E-06 |
| RP11-693J15.6 | 2.17 | 4.86 | 5.74E-06 | 1.01E-05 |
| RP11-459O1.2 | 2.22 | 3.36 | 5.81E-06 | 1.02E-05 |
| RP11-749H17.2 | 2.15 | 2.42 | 7.02E-06 | 1.23E-05 |
| CTB-61M7.1 | 2.71 | 2.80 | 7.32E-06 | 1.28E-05 |
| LINC02212 | 2.25 | 4.17 | 7.36E-06 | 1.28E-05 |
| LINC02200 | 2.15 | 2.48 | 7.90E-06 | 1.37E-05 |
| RP11-720L8.1 | 2.26 | 2.57 | 8.42E-06 | 1.46E-05 |
| LINC01324 | 2.24 | 3.19 | 8.84E-06 | 1.53E-05 |
| RP11-1336O20.2 | 2.01 | 3.24 | 8.88E-06 | 1.54E-05 |
| LINC02125 | 2.02 | 2.83 | 8.97E-06 | 1.55E-05 |
| RP11-25C19.3 | 2.02 | 2.73 | 9.82E-06 | 1.69E-05 |
| LINC00967 | 2.27 | 2.98 | 1.52E-05 | 2.58E-05 |
| LINC01793 | 2.55 | 3.47 | 1.63E-05 | 2.76E-05 |
| ADIPOQ-AS1 | 2.27 | 2.50 | 2.83E-05 | 4.71E-05 |
| LINC00379 | 2.33 | 2.94 | 5.18E-05 | 8.43E-05 |
| RP11-232D9.3 | 2.38 | 2.83 | 8.23E-05 | 0.00013187 |
| RP11-436H11.5 | 2.16 | 2.44 | 8.38E-05 | 0.000134212 |
| CTC-546K23.1 | 2.07 | 2.70 | 0.000105499 | 0.000167741 |
| AP000462.1 | 2.12 | 2.87 | 0.000176736 | 0.000275503 |

Table S6. The significantly dysregulated lncRNAs in lung adenocarcinoma in the TCGA database

| LncRNA | logFC | logCPM | P-value | FDR |
| --- | --- | --- | --- | --- |
| AC093110.3 | -3.37 | 7.93 | 1.11E-213 | 9.96E-210 |
| RP11-371A19.2 | -5.21 | 5.74 | 1.47E-181 | 6.61E-178 |
| HSPC324 | -3.71 | 6.02 | 1.17E-167 | 3.52E-164 |
| FENDRR | -3.97 | 9.99 | 6.77E-154 | 1.52E-150 |
| LANCL1-AS1 | -3.74 | 6.93 | 1.81E-150 | 3.26E-147 |
| LINC00968 | -3.59 | 7.28 | 2.42E-139 | 3.63E-136 |
| AP001189.4 | -3.09 | 6.13 | 1.11E-127 | 1.43E-124 |
| RP4-575N6.5 | -2.90 | 3.92 | 2.43E-126 | 2.73E-123 |
| RP5-826L7.1 | -3.50 | 3.65 | 3.88E-126 | 3.89E-123 |
| LINC01082 | -4.19 | 4.15 | 1.49E-123 | 1.35E-120 |
| MIR3945HG | -3.54 | 6.72 | 1.26E-118 | 1.03E-115 |
| RP11-805I24.3 | -4.09 | 4.87 | 2.31E-118 | 1.74E-115 |
| HID1-AS1 | -2.79 | 4.82 | 5.85E-110 | 4.05E-107 |
| AC018647.3 | -2.80 | 5.40 | 3.05E-109 | 1.96E-106 |
| LINC02016 | -5.01 | 5.57 | 9.36E-104 | 5.62E-101 |
| RP11-287F9.2 | -4.56 | 3.79 | 3.16E-99 | 1.78E-96 |
| LINC01996 | -4.49 | 5.96 | 7.56E-98 | 4.01E-95 |
| LINC00656 | -3.49 | 4.23 | 2.48E-97 | 1.24E-94 |
| RP11-354P11.4 | -4.12 | 3.46 | 1.72E-93 | 8.15E-91 |
| PCAT19 | -2.42 | 9.50 | 1.88E-93 | 8.47E-91 |
| AC011899.9 | -2.40 | 8.63 | 5.78E-92 | 2.48E-89 |
| RP4-575N6.4 | -2.51 | 5.03 | 4.57E-90 | 1.87E-87 |
| RP11-354P11.2 | -4.09 | 4.10 | 1.03E-89 | 4.04E-87 |
| RP11-598F7.3 | -3.40 | 6.77 | 1.21E-89 | 4.55E-87 |
| AC128709.2 | -4.29 | 3.87 | 4.67E-89 | 1.68E-86 |
| AC011286.1 | -4.41 | 3.22 | 5.38E-88 | 1.87E-85 |
| RP11-613D13.8 | -2.76 | 5.72 | 7.37E-82 | 2.46E-79 |
| LINC00163 | -3.43 | 4.55 | 2.97E-80 | 9.22E-78 |
| LINC01290 | -2.27 | 4.98 | 3.10E-80 | 9.31E-78 |
| SMIM25 | -2.47 | 10.70 | 6.73E-77 | 1.96E-74 |
| RP11-286H15.1 | -3.10 | 5.36 | 1.30E-75 | 3.67E-73 |
| RP1-78O14.1 | -3.20 | 7.94 | 1.46E-75 | 3.97E-73 |
| RP11-672A2.4 | -2.44 | 6.12 | 6.62E-75 | 1.75E-72 |
| RP11-51B23.3 | -2.40 | 3.88 | 6.95E-75 | 1.79E-72 |
| RP1-18D14.7 | -2.64 | 4.89 | 8.00E-75 | 2.00E-72 |
| LINC01081 | -3.13 | 2.83 | 4.43E-74 | 1.08E-71 |
| AP000438.2 | -3.00 | 3.70 | 3.84E-72 | 9.11E-70 |
| ADAMTS9-AS1 | -2.74 | 7.05 | 4.58E-71 | 1.06E-68 |
| RP11-2N1.3 | -3.85 | 2.85 | 4.95E-71 | 1.11E-68 |
| RP11-403A3.3 | -2.95 | 4.83 | 2.56E-69 | 5.36E-67 |
| AC006273.4 | -2.61 | 4.77 | 5.06E-69 | 1.04E-66 |
| RP11-35J10.7 | -3.54 | 3.66 | 8.15E-68 | 1.63E-65 |
| LINC00551 | -3.08 | 5.81 | 2.05E-67 | 3.93E-65 |
| RP11-35J10.6 | -3.52 | 3.33 | 2.42E-67 | 4.53E-65 |
| RP11-253E3.3 | -2.09 | 8.48 | 1.26E-66 | 2.32E-64 |
| RP11-295M18.6 | -2.41 | 4.16 | 1.37E-66 | 2.47E-64 |
| LINC01936 | -2.43 | 8.69 | 6.78E-66 | 1.15E-63 |
| LINC01447 | -3.01 | 3.67 | 1.61E-65 | 2.68E-63 |
| AC004947.2 | -3.04 | 5.52 | 2.19E-65 | 3.58E-63 |
| TBX2-AS1 | -2.12 | 7.11 | 4.05E-65 | 6.51E-63 |
| RP11-475O23.2 | -3.53 | 3.57 | 2.68E-64 | 4.16E-62 |
| LINC01352 | -2.32 | 4.83 | 4.44E-64 | 6.78E-62 |
| CTD-2369P2.8 | -2.61 | 8.13 | 1.49E-63 | 2.24E-61 |
| LINC01977 | 4.79 | 7.17 | 2.30E-63 | 3.40E-61 |
| LINC01197 | -2.08 | 6.31 | 5.58E-63 | 8.11E-61 |
| MED4-AS1 | -2.28 | 4.52 | 7.18E-63 | 1.03E-60 |
| RP11-100L22.1 | -2.91 | 3.60 | 2.58E-62 | 3.63E-60 |
| RP11-452C13.1 | -2.22 | 4.90 | 9.81E-62 | 1.34E-59 |
| RP11-283G6.4 | -2.19 | 5.86 | 4.11E-61 | 5.53E-59 |
| RP11-544M22.1 | -3.99 | 5.20 | 3.21E-60 | 4.19E-58 |
| PACRG-AS3 | -3.87 | 3.82 | 1.11E-59 | 1.43E-57 |
| RP11-352D13.6 | -2.81 | 6.58 | 4.00E-59 | 5.08E-57 |
| FAM83A-AS1 | 6.55 | 8.67 | 5.57E-59 | 6.97E-57 |
| LINC01863 | -3.25 | 4.61 | 9.00E-59 | 1.11E-56 |
| RP11-494M8.4 | -3.35 | 4.70 | 1.55E-58 | 1.88E-56 |
| RP11-335L23.5 | -2.30 | 3.95 | 4.05E-58 | 4.87E-56 |
| RP11-594N15.3 | -2.39 | 5.31 | 6.00E-58 | 7.11E-56 |
| RP11-4B16.3 | -2.62 | 3.78 | 1.00E-57 | 1.18E-55 |
| LINC02014 | 3.98 | 6.59 | 1.15E-57 | 1.32E-55 |
| LL22NC03-104C7.1 | -3.45 | 3.16 | 9.57E-57 | 1.08E-54 |
| LINC00702 | -2.25 | 7.57 | 3.09E-55 | 3.44E-53 |
| PTPRD-AS1 | -2.30 | 5.51 | 5.34E-55 | 5.87E-53 |
| RP11-561I11.3 | -3.17 | 3.34 | 2.30E-53 | 2.47E-51 |
| RP4-564M11.2 | -2.39 | 4.78 | 4.39E-53 | 4.66E-51 |
| LINC01506 | -2.43 | 4.26 | 7.39E-52 | 7.74E-50 |
| LINC02154 | -3.30 | 7.13 | 2.19E-51 | 2.27E-49 |
| LINC01624 | -2.32 | 4.04 | 4.42E-51 | 4.52E-49 |
| AFAP1-AS1 | 6.36 | 12.84 | 2.13E-50 | 2.16E-48 |
| RP11-714G18.1 | -2.18 | 5.01 | 2.84E-50 | 2.84E-48 |
| CTD-2319I12.5 | -2.52 | 2.95 | 5.13E-50 | 5.08E-48 |
| LINC00891 | -2.44 | 5.17 | 5.91E-50 | 5.79E-48 |
| RP11-136H19.1 | -2.00 | 4.23 | 6.53E-50 | 6.32E-48 |
| RP5-1061H20.4 | 2.34 | 6.85 | 8.30E-50 | 7.96E-48 |
| PVT1 | 2.66 | 10.46 | 1.77E-49 | 1.68E-47 |
| MNX1-AS1 | 5.22 | 7.39 | 2.42E-49 | 2.27E-47 |
| LINC00511 | 3.13 | 11.23 | 4.04E-49 | 3.75E-47 |
| RP11-44B19.1 | -2.81 | 3.14 | 1.51E-48 | 1.38E-46 |
| MGC27382 | -3.12 | 6.23 | 2.27E-48 | 2.04E-46 |
| RP6-65G23.3 | 2.96 | 8.69 | 2.63E-47 | 2.34E-45 |
| RUNDC3A-AS1 | 3.74 | 7.33 | 3.55E-47 | 3.14E-45 |
| C1orf140 | -2.63 | 3.29 | 3.95E-47 | 3.46E-45 |
| RP11-434D9.1 | -2.70 | 6.30 | 5.35E-47 | 4.63E-45 |
| RP3-332B22.1 | -2.84 | 3.13 | 5.40E-47 | 4.63E-45 |
| RP11-783K16.5 | 3.47 | 7.25 | 3.39E-46 | 2.83E-44 |
| DDX11-AS1 | 2.43 | 6.38 | 3.47E-46 | 2.86E-44 |
| RP11-264B14.1 | -2.38 | 3.84 | 6.01E-46 | 4.81E-44 |
| CTD-2527I21.15 | 5.79 | 6.81 | 6.04E-46 | 4.81E-44 |
| ZFPM2-AS1 | 5.31 | 8.48 | 6.12E-46 | 4.84E-44 |
| FGF10-AS1 | -3.23 | 2.82 | 1.98E-45 | 1.54E-43 |
| LINC01572 | 2.88 | 6.19 | 2.84E-45 | 2.17E-43 |
| RP11-439L18.1 | -2.38 | 3.78 | 5.90E-45 | 4.46E-43 |
| RP3-340N1.2 | 6.02 | 10.00 | 1.61E-44 | 1.19E-42 |
| PCAT6 | 2.68 | 8.81 | 2.08E-44 | 1.52E-42 |
| LINC01270 | 2.86 | 8.33 | 4.39E-44 | 3.19E-42 |
| RP11-539E17.5 | 5.46 | 5.05 | 5.04E-44 | 3.63E-42 |
| RP5-839B4.8 | -2.97 | 7.22 | 9.98E-44 | 7.08E-42 |
| LINC01165 | -2.47 | 3.31 | 1.88E-43 | 1.32E-41 |
| AC090616.2 | -2.04 | 8.20 | 3.52E-43 | 2.44E-41 |
| FEZF1-AS1 | 6.12 | 9.48 | 3.97E-43 | 2.73E-41 |
| AC004540.4 | -2.05 | 6.16 | 4.15E-43 | 2.83E-41 |
| CTD-3010D24.3 | 5.31 | 7.42 | 4.26E-43 | 2.89E-41 |
| HLX-AS1 | -2.30 | 3.39 | 8.02E-43 | 5.35E-41 |
| CTD-2003C8.2 | -2.26 | 4.73 | 2.17E-42 | 1.44E-40 |
| LINC01070 | -3.44 | 2.67 | 2.74E-42 | 1.80E-40 |
| NAV2-AS2 | -2.75 | 4.39 | 3.35E-42 | 2.19E-40 |
| RP11-352D13.5 | -2.41 | 4.33 | 3.86E-42 | 2.50E-40 |
| RP11-27M24.2 | -2.36 | 4.79 | 3.92E-42 | 2.52E-40 |
| AC004490.1 | -2.13 | 4.22 | 5.42E-42 | 3.46E-40 |
| LINC00472 | -2.14 | 6.44 | 1.65E-41 | 1.04E-39 |
| RP13-1016M1.2 | -2.01 | 4.45 | 1.91E-41 | 1.20E-39 |
| FOXD3-AS1 | 6.92 | 6.93 | 2.31E-41 | 1.43E-39 |
| LINC01645 | -2.69 | 4.07 | 4.67E-41 | 2.86E-39 |
| C5orf64 | -2.12 | 3.43 | 6.64E-41 | 4.04E-39 |
| RP11-863P13.4 | -2.58 | 4.04 | 9.46E-41 | 5.68E-39 |
| CTD-2515H24.2 | -2.23 | 4.98 | 9.97E-41 | 5.91E-39 |
| AC007182.6 | -2.70 | 3.88 | 1.43E-40 | 8.34E-39 |
| RP11-493L12.5 | 4.22 | 5.49 | 1.67E-40 | 9.68E-39 |
| RP11-203H2.2 | -3.04 | 2.81 | 2.95E-40 | 1.70E-38 |
| XXbac-BPG27H4.8 | -3.41 | 3.66 | 3.13E-40 | 1.80E-38 |
| AP002856.5 | -4.15 | 5.32 | 3.74E-40 | 2.13E-38 |
| C1orf220 | 2.20 | 6.13 | 4.73E-40 | 2.68E-38 |
| LINC00844 | -2.90 | 3.09 | 1.46E-39 | 8.05E-38 |
| RP11-79H23.3 | -2.09 | 6.72 | 4.54E-39 | 2.47E-37 |
| TFAP2A-AS1 | 3.05 | 6.11 | 5.41E-39 | 2.92E-37 |
| RP11-356N1.2 | -2.13 | 4.45 | 9.31E-39 | 4.99E-37 |
| AC145343.2 | 3.04 | 6.91 | 1.08E-38 | 5.73E-37 |
| VPS9D1-AS1 | 2.93 | 8.97 | 1.49E-38 | 7.87E-37 |
| CADM3-AS1 | -2.23 | 6.07 | 1.66E-38 | 8.70E-37 |
| LINC02104 | -2.14 | 3.97 | 3.27E-38 | 1.69E-36 |
| RP11-246K15.1 | -2.88 | 4.21 | 4.98E-38 | 2.55E-36 |
| LINC01985 | -2.59 | 3.13 | 9.41E-38 | 4.76E-36 |
| AC011294.3 | 4.90 | 7.19 | 1.22E-37 | 6.13E-36 |
| AF131215.8 | -2.29 | 4.26 | 3.24E-37 | 1.61E-35 |
| RP11-386G11.5 | 2.59 | 6.65 | 5.18E-37 | 2.57E-35 |
| CTD-3247F14.2 | -2.35 | 4.59 | 6.48E-37 | 3.17E-35 |
| LINC01271 | 2.58 | 5.96 | 7.22E-37 | 3.50E-35 |
| LINC01703 | 2.36 | 6.21 | 7.94E-37 | 3.82E-35 |
| RP11-64B16.4 | -2.80 | 3.10 | 8.71E-37 | 4.17E-35 |
| RP1-251M9.3 | -3.35 | 3.78 | 9.09E-37 | 4.33E-35 |
| RP1-140C12.2 | -2.12 | 3.15 | 9.49E-37 | 4.50E-35 |
| RP11-81H3.2 | 6.84 | 6.63 | 1.00E-36 | 4.74E-35 |
| RP11-429J17.7 | 2.69 | 6.83 | 1.45E-36 | 6.80E-35 |
| CTA-384D8.35 | 2.85 | 8.54 | 2.08E-36 | 9.72E-35 |
| LINC00857 | 2.32 | 8.76 | 2.18E-36 | 1.01E-34 |
| BLACAT1 | 2.94 | 8.93 | 2.54E-36 | 1.17E-34 |
| CTD-2501M5.1 | -3.38 | 3.31 | 2.78E-36 | 1.28E-34 |
| LINC00211 | -2.30 | 3.46 | 3.58E-36 | 1.63E-34 |
| RP4-724E16.2 | 2.32 | 7.34 | 7.25E-36 | 3.28E-34 |
| AGAP11 | -2.07 | 4.04 | 8.31E-36 | 3.74E-34 |
| CTD-3193K9.11 | -2.24 | 3.83 | 1.54E-35 | 6.91E-34 |
| WASIR2 | 3.41 | 5.85 | 1.67E-35 | 7.45E-34 |
| CTD-3060P21.1 | 3.16 | 5.93 | 3.46E-35 | 1.53E-33 |
| RP11-794G24.1 | 3.52 | 6.25 | 3.49E-35 | 1.53E-33 |
| LINC01412 | -2.19 | 3.12 | 3.89E-35 | 1.70E-33 |
| CTD-3224I3.3 | -2.39 | 2.83 | 5.54E-35 | 2.41E-33 |
| AC133785.1 | 6.74 | 6.12 | 6.40E-35 | 2.76E-33 |
| RP11-95I16.2 | -2.82 | 4.60 | 1.78E-34 | 7.57E-33 |
| RP13-463N16.6 | 5.12 | 5.85 | 3.88E-34 | 1.62E-32 |
| LINC00896 | 3.24 | 6.30 | 5.48E-34 | 2.27E-32 |
| RP11-30P6.6 | 4.42 | 6.05 | 7.37E-34 | 3.05E-32 |
| AC007128.1 | 5.39 | 6.45 | 7.50E-34 | 3.07E-32 |
| SNHG4 | 2.15 | 7.94 | 1.32E-33 | 5.33E-32 |
| RP1-179N16.6 | 2.20 | 6.41 | 1.52E-33 | 6.10E-32 |
| TDRKH-AS1 | 2.01 | 6.83 | 1.58E-33 | 6.30E-32 |
| RP11-384F7.2 | -2.97 | 4.33 | 2.26E-33 | 8.97E-32 |
| RP11-336A10.5 | 4.45 | 5.58 | 2.55E-33 | 1.01E-31 |
| LINC01616 | -3.17 | 3.11 | 3.62E-33 | 1.42E-31 |
| RP11-304L19.1 | 3.90 | 7.69 | 6.78E-33 | 2.62E-31 |
| RP11-49G2.3 | -2.06 | 4.13 | 8.53E-33 | 3.26E-31 |
| RP11-492E3.2 | 4.37 | 8.41 | 8.57E-33 | 3.26E-31 |
| AC015849.16 | 3.64 | 5.80 | 9.76E-33 | 3.68E-31 |
| BBOX1-AS1 | 5.58 | 7.59 | 1.15E-32 | 4.29E-31 |
| RP11-546J1.1 | 2.07 | 5.10 | 1.51E-32 | 5.63E-31 |
| AC124789.1 | -2.37 | 6.91 | 1.83E-32 | 6.79E-31 |
| RP5-908M14.10 | 2.18 | 6.49 | 1.85E-32 | 6.79E-31 |
| RP13-497K6.1 | -2.84 | 3.17 | 3.00E-32 | 1.10E-30 |
| RP11-738B7.1 | 3.19 | 4.04 | 3.48E-32 | 1.27E-30 |
| RP11-304L19.3 | 4.00 | 7.57 | 4.80E-32 | 1.72E-30 |
| RP5-1103B4.3 | -3.15 | 2.85 | 1.26E-31 | 4.45E-30 |
| AE000662.93 | 3.90 | 4.10 | 1.29E-31 | 4.54E-30 |
| RP11-108L7.15 | 2.01 | 5.37 | 1.52E-31 | 5.31E-30 |
| RP11-572O17.1 | 2.00 | 5.99 | 1.83E-31 | 6.38E-30 |
| LINC01426 | 2.46 | 9.08 | 1.88E-31 | 6.52E-30 |
| RP11-244M2.1 | 3.85 | 6.51 | 3.26E-31 | 1.12E-29 |
| CTD-2227E11.1 | 2.81 | 6.64 | 3.62E-31 | 1.24E-29 |
| PGM5-AS1 | -2.34 | 3.74 | 3.67E-31 | 1.25E-29 |
| AC002398.12 | -2.43 | 2.86 | 3.94E-31 | 1.33E-29 |
| RP11-672A2.5 | -2.19 | 2.86 | 4.55E-31 | 1.53E-29 |
| LINC00862 | 3.75 | 6.13 | 1.13E-30 | 3.77E-29 |
| RP11-650L12.2 | 3.86 | 7.43 | 1.21E-30 | 4.01E-29 |
| LINC01614 | 3.72 | 7.66 | 1.22E-30 | 4.05E-29 |
| RP11-519G16.5 | 4.05 | 8.56 | 1.65E-30 | 5.39E-29 |
| RP11-89B16.1 | -2.04 | 3.97 | 1.74E-30 | 5.67E-29 |
| RP11-54O7.18 | 3.93 | 5.46 | 1.82E-30 | 5.91E-29 |
| NPSR1-AS1 | 5.70 | 5.06 | 2.04E-30 | 6.56E-29 |
| CLDN10-AS1 | 6.49 | 7.43 | 2.15E-30 | 6.89E-29 |
| RP11-59D5__B.2 | 4.03 | 8.15 | 3.13E-30 | 9.94E-29 |
| RP11-98G7.1 | 4.16 | 5.46 | 4.66E-30 | 1.47E-28 |
| SLC2A1-AS1 | 2.34 | 6.54 | 8.21E-30 | 2.57E-28 |
| RP11-284F21.10 | 4.49 | 10.35 | 1.26E-29 | 3.93E-28 |
| TYMSOS | 2.47 | 6.37 | 1.73E-29 | 5.35E-28 |
| RP11-127I20.5 | 2.53 | 5.65 | 1.79E-29 | 5.51E-28 |
| RP11-390F4.3 | 2.97 | 7.66 | 2.60E-29 | 8.01E-28 |
| CYP4A22-AS1 | 2.52 | 5.09 | 2.93E-29 | 8.96E-28 |
| RP11-211G23.2 | 6.05 | 6.30 | 3.04E-29 | 9.29E-28 |
| RP11-540A21.2 | 2.16 | 6.33 | 3.09E-29 | 9.39E-28 |
| RP11-57A19.2 | 3.13 | 6.34 | 3.47E-29 | 1.05E-27 |
| LINC01711 | 3.83 | 5.10 | 4.45E-29 | 1.34E-27 |
| RP11-180I4.4 | -2.43 | 3.27 | 4.97E-29 | 1.49E-27 |
| RP11-284F21.9 | 5.34 | 8.97 | 5.42E-29 | 1.62E-27 |
| RP11-544L8__B.4 | -2.35 | 3.85 | 6.29E-29 | 1.88E-27 |
| CTD-2033A16.3 | 2.82 | 5.09 | 1.15E-28 | 3.39E-27 |
| RP4-594A5.1 | 6.03 | 4.90 | 1.40E-28 | 4.12E-27 |
| RP1-244F24.1 | 2.15 | 5.55 | 1.52E-28 | 4.42E-27 |
| C2orf48 | 2.95 | 6.03 | 1.63E-28 | 4.74E-27 |
| CTD-2510F5.4 | 2.46 | 7.71 | 2.10E-28 | 6.05E-27 |
| RP11-204P2.3 | -2.36 | 2.63 | 2.15E-28 | 6.16E-27 |
| KB-1440D3.13 | 2.82 | 4.95 | 2.91E-28 | 8.33E-27 |
| LINC02159 | 4.17 | 7.56 | 4.66E-28 | 1.31E-26 |
| CH17-360D5.2 | -2.43 | 8.19 | 4.71E-28 | 1.32E-26 |
| CTD-2023N9.1 | -2.39 | 2.90 | 5.14E-28 | 1.43E-26 |
| LINC01460 | 3.85 | 6.93 | 6.20E-28 | 1.72E-26 |
| RP1-15D23.2 | -2.44 | 3.00 | 1.33E-27 | 3.64E-26 |
| MGAT3-AS1 | -2.44 | 3.62 | 1.46E-27 | 4.00E-26 |
| DGCR9 | 3.03 | 7.41 | 2.49E-27 | 6.78E-26 |
| RP11-57A1.1 | 3.53 | 4.14 | 3.09E-27 | 8.38E-26 |
| KB-1460A1.1 | 2.73 | 5.38 | 4.17E-27 | 1.12E-25 |
| RP11-290F5.1 | 2.57 | 7.93 | 4.42E-27 | 1.19E-25 |
| RP11-964E11.2 | 3.34 | 7.02 | 5.02E-27 | 1.34E-25 |
| RP11-295G20.2 | 2.83 | 9.71 | 5.22E-27 | 1.39E-25 |
| RP11-370I10.2 | -2.21 | 3.64 | 5.44E-27 | 1.44E-25 |
| SEMA6A-AS2 | -2.04 | 2.60 | 6.03E-27 | 1.59E-25 |
| LINC00942 | 6.58 | 9.74 | 6.99E-27 | 1.83E-25 |
| RP11-932O9.10 | 2.29 | 4.76 | 7.09E-27 | 1.85E-25 |
| RP11-157F20.3 | 3.06 | 3.95 | 8.17E-27 | 2.12E-25 |
| CTD-2531D15.4 | -2.46 | 4.49 | 9.73E-27 | 2.52E-25 |
| KCNMB2-AS1 | 5.51 | 7.69 | 1.01E-26 | 2.60E-25 |
| RP11-10A14.5 | 4.57 | 7.07 | 1.08E-26 | 2.77E-25 |
| RP11-123B3.2 | 5.28 | 5.03 | 1.35E-26 | 3.43E-25 |
| RP11-77A13.1 | -3.16 | 6.90 | 1.49E-26 | 3.80E-25 |
| RP11-312J18.6 | -2.87 | 4.19 | 1.66E-26 | 4.21E-25 |
| CTD-2536I1.3 | -2.16 | 2.58 | 2.00E-26 | 5.03E-25 |
| RP11-17A4.2 | -2.44 | 3.45 | 2.05E-26 | 5.13E-25 |
| RP11-284F21.7 | 3.80 | 7.72 | 2.08E-26 | 5.20E-25 |
| SLC12A9-AS1 | 2.18 | 5.59 | 2.47E-26 | 6.17E-25 |
| HOXC-AS2 | 5.07 | 5.99 | 2.61E-26 | 6.48E-25 |
| AC009005.2 | 2.12 | 7.97 | 3.60E-26 | 8.93E-25 |
| RP11-387H17.6 | -2.17 | 3.11 | 3.73E-26 | 9.20E-25 |
| RP5-963E22.6 | 2.03 | 5.64 | 4.68E-26 | 1.15E-24 |
| RP11-470P21.2 | 5.12 | 4.79 | 5.53E-26 | 1.35E-24 |
| LINC01833 | 6.66 | 7.41 | 5.75E-26 | 1.40E-24 |
| RP11-416I2.1 | 3.35 | 6.47 | 6.14E-26 | 1.49E-24 |
| LINC01705 | 3.86 | 5.21 | 6.53E-26 | 1.58E-24 |
| RP11-806K15.1 | -3.10 | 2.59 | 6.88E-26 | 1.66E-24 |
| U52111.14 | 2.26 | 6.04 | 7.13E-26 | 1.71E-24 |
| CTA-384D8.34 | 2.82 | 6.64 | 7.58E-26 | 1.81E-24 |
| RP11-496D24.2 | 4.13 | 4.10 | 9.72E-26 | 2.28E-24 |
| RP11-417E7.2 | 3.58 | 6.00 | 1.18E-25 | 2.76E-24 |
| CTD-2337I7.1 | 4.15 | 5.54 | 1.30E-25 | 3.04E-24 |
| AC005324.6 | -2.93 | 2.82 | 1.35E-25 | 3.13E-24 |
| LINC00629 | 3.46 | 4.51 | 1.60E-25 | 3.70E-24 |
| CTD-2530N21.5 | -2.91 | 4.20 | 2.26E-25 | 5.20E-24 |
| AL163953.2 | 5.25 | 4.09 | 2.36E-25 | 5.41E-24 |
| LINC01607 | 2.46 | 6.48 | 2.56E-25 | 5.86E-24 |
| CTC-431G16.2 | 4.45 | 6.23 | 9.16E-25 | 2.05E-23 |
| AC005256.1 | 6.79 | 4.82 | 1.24E-24 | 2.74E-23 |
| RP11-504P24.9 | 2.09 | 4.89 | 1.36E-24 | 2.99E-23 |
| LINC01395 | 4.92 | 5.13 | 1.40E-24 | 3.06E-23 |
| RP11-145A3.1 | 2.92 | 5.41 | 1.83E-24 | 3.96E-23 |
| LINC00628 | 3.60 | 3.91 | 1.96E-24 | 4.24E-23 |
| RP11-2N1.2 | -2.73 | 5.13 | 2.40E-24 | 5.15E-23 |
| AC007285.7 | 2.27 | 4.27 | 2.54E-24 | 5.42E-23 |
| RP11-734K21.5 | 3.79 | 6.71 | 2.55E-24 | 5.43E-23 |
| LINC02003 | 4.12 | 4.13 | 3.28E-24 | 6.94E-23 |
| LINC00887 | 3.97 | 5.88 | 3.56E-24 | 7.47E-23 |
| RP11-159H10.3 | 3.19 | 5.23 | 4.52E-24 | 9.39E-23 |
| PGM5P4-AS1 | -2.00 | 3.99 | 5.26E-24 | 1.09E-22 |
| LINC00624 | 2.24 | 7.04 | 5.54E-24 | 1.14E-22 |
| CTD-2547H18.1 | 4.09 | 6.78 | 5.75E-24 | 1.18E-22 |
| RP11-635O16.2 | -2.50 | 6.67 | 6.40E-24 | 1.31E-22 |
| RP11-400N13.2 | 4.74 | 7.67 | 6.72E-24 | 1.37E-22 |
| RP11-796E10.1 | 4.55 | 4.97 | 7.92E-24 | 1.60E-22 |
| LINC00461 | 4.05 | 4.51 | 8.51E-24 | 1.72E-22 |
| AC195454.1 | -2.05 | 4.69 | 8.71E-24 | 1.75E-22 |
| RP11-734K21.2 | 3.47 | 4.94 | 1.29E-23 | 2.55E-22 |
| LINC00880 | 3.38 | 6.00 | 1.39E-23 | 2.74E-22 |
| LINC00525 | 3.41 | 5.41 | 1.63E-23 | 3.19E-22 |
| KB-1615E4.2 | 3.78 | 4.16 | 1.89E-23 | 3.68E-22 |
| RP5-1120P11.1 | 2.93 | 8.77 | 2.11E-23 | 4.09E-22 |
| RP11-169F17.1 | 6.66 | 9.64 | 2.21E-23 | 4.27E-22 |
| F11-AS1 | -2.28 | 5.32 | 2.47E-23 | 4.76E-22 |
| RP11-676J12.6 | -2.08 | 3.71 | 2.71E-23 | 5.20E-22 |
| LINC01348 | 2.62 | 7.69 | 2.92E-23 | 5.60E-22 |
| RP11-359N11.1 | -2.25 | 3.35 | 3.21E-23 | 6.13E-22 |
| RP11-357D18.1 | -2.34 | 7.25 | 3.34E-23 | 6.37E-22 |
| RP4-735C1.4 | -2.28 | 3.16 | 5.99E-23 | 1.12E-21 |
| RP11-254I22.3 | 3.67 | 6.74 | 6.75E-23 | 1.25E-21 |
| RP11-366L20.2 | 3.17 | 6.23 | 6.98E-23 | 1.29E-21 |
| VAC14-AS1 | 2.53 | 5.76 | 7.42E-23 | 1.36E-21 |
| RP11-124O11.1 | -2.06 | 3.17 | 7.91E-23 | 1.45E-21 |
| RP11-616M22.7 | 5.59 | 5.03 | 1.28E-22 | 2.34E-21 |
| RP11-875O11.3 | 2.31 | 6.45 | 1.45E-22 | 2.63E-21 |
| MNX1-AS2 | 2.92 | 5.26 | 1.82E-22 | 3.27E-21 |
| CTC-327F10.4 | 4.75 | 3.80 | 1.85E-22 | 3.32E-21 |
| LINC01993 | 2.79 | 5.70 | 1.96E-22 | 3.51E-21 |
| AF127936.3 | 2.99 | 4.83 | 2.67E-22 | 4.72E-21 |
| RP1-27K12.4 | 4.16 | 6.00 | 2.83E-22 | 5.00E-21 |
| RP11-713C5.1 | 4.28 | 5.86 | 2.91E-22 | 5.14E-21 |
| LINC02081 | 2.94 | 7.10 | 3.23E-22 | 5.68E-21 |
| LINC00665 | 2.31 | 11.15 | 3.39E-22 | 5.94E-21 |
| RP11-434I12.3 | 4.40 | 7.09 | 3.39E-22 | 5.94E-21 |
| RMDN2-AS1 | 2.62 | 5.82 | 3.49E-22 | 6.10E-21 |
| LINC01983 | 3.77 | 6.29 | 4.00E-22 | 6.94E-21 |
| RP4-539M6.14 | -2.21 | 6.50 | 4.70E-22 | 8.13E-21 |
| MYOSLID | 3.43 | 7.59 | 5.28E-22 | 9.13E-21 |
| RP11-350J20.12 | 2.74 | 8.23 | 5.44E-22 | 9.39E-21 |
| RP11-742B18.1 | 3.69 | 6.16 | 7.04E-22 | 1.21E-20 |
| RP11-114H23.1 | 3.97 | 5.68 | 7.44E-22 | 1.27E-20 |
| RP5-1063M23.2 | 2.97 | 5.25 | 7.97E-22 | 1.36E-20 |
| RP11-238K6.1 | -2.46 | 7.05 | 8.05E-22 | 1.37E-20 |
| AP000251.3 | 3.42 | 5.25 | 9.64E-22 | 1.63E-20 |
| LINC00337 | 2.85 | 6.13 | 1.02E-21 | 1.72E-20 |
| RP11-211C9.1 | 3.82 | 5.86 | 1.03E-21 | 1.74E-20 |
| LINC01208 | 4.09 | 3.78 | 1.14E-21 | 1.92E-20 |
| CTC-255N20.1 | 3.06 | 6.81 | 1.28E-21 | 2.14E-20 |
| RP11-44N12.5 | 3.14 | 4.86 | 1.72E-21 | 2.85E-20 |
| AC069513.4 | 2.28 | 4.23 | 1.82E-21 | 3.01E-20 |
| TRIM31-AS1 | 2.41 | 6.04 | 1.93E-21 | 3.19E-20 |
| RP6-191P20.4 | 4.20 | 4.22 | 2.23E-21 | 3.65E-20 |
| FRGCA | 3.70 | 5.20 | 2.33E-21 | 3.82E-20 |
| RP4-694A7.2 | 4.97 | 4.32 | 2.53E-21 | 4.12E-20 |
| CTC-441N14.2 | -2.03 | 4.03 | 2.58E-21 | 4.20E-20 |
| RP11-474P2.6 | 2.19 | 5.62 | 2.64E-21 | 4.29E-20 |
| CASC16 | 4.24 | 4.65 | 2.79E-21 | 4.52E-20 |
| LINC01765 | -2.49 | 5.46 | 3.00E-21 | 4.85E-20 |
| AC079630.4 | -2.03 | 9.93 | 3.14E-21 | 5.06E-20 |
| RP4-760C5.3 | 2.43 | 4.45 | 3.17E-21 | 5.08E-20 |
| AC123886.2 | -2.10 | 2.96 | 3.17E-21 | 5.08E-20 |
| RP5-836N17.4 | 3.08 | 3.88 | 3.19E-21 | 5.09E-20 |
| RP11-118K6.3 | 2.24 | 5.21 | 3.58E-21 | 5.71E-20 |
| LHFPL3-AS2 | -2.20 | 9.71 | 4.49E-21 | 7.11E-20 |
| XXyac-YM21GA2.3 | -2.78 | 3.46 | 4.52E-21 | 7.15E-20 |
| UCA1 | 5.69 | 9.33 | 5.47E-21 | 8.56E-20 |
| CTD-2008P7.8 | 6.54 | 5.79 | 6.56E-21 | 1.02E-19 |
| AC013264.2 | -2.07 | 6.77 | 6.62E-21 | 1.03E-19 |
| LINC01827 | -2.02 | 4.00 | 8.68E-21 | 1.34E-19 |
| LINC00160 | 3.56 | 5.20 | 1.02E-20 | 1.57E-19 |
| CTD-2532K18.2 | 4.28 | 3.90 | 1.18E-20 | 1.81E-19 |
| RP11-895M11.3 | 2.44 | 3.54 | 1.40E-20 | 2.13E-19 |
| RP11-54O7.1 | 3.42 | 5.50 | 1.45E-20 | 2.21E-19 |
| PACRG-AS1 | -2.02 | 3.07 | 1.48E-20 | 2.26E-19 |
| AC007386.4 | 2.22 | 5.74 | 1.53E-20 | 2.32E-19 |
| LL21NC02-1C16.2 | 2.80 | 7.30 | 1.91E-20 | 2.88E-19 |
| RP4-712E4.1 | 3.88 | 3.63 | 2.40E-20 | 3.61E-19 |
| RP11-98D18.17 | 2.19 | 4.38 | 2.58E-20 | 3.86E-19 |
| LINC00866 | 2.80 | 4.12 | 3.16E-20 | 4.72E-19 |
| PTGES2-AS1 | 2.36 | 4.56 | 3.63E-20 | 5.41E-19 |
| AC007750.5 | 2.00 | 5.49 | 3.67E-20 | 5.47E-19 |
| RP3-512B11.3 | 2.11 | 7.30 | 4.40E-20 | 6.51E-19 |
| RP11-909N17.2 | 4.37 | 5.53 | 4.85E-20 | 7.17E-19 |
| CTD-2619J13.13 | 2.91 | 7.24 | 5.19E-20 | 7.63E-19 |
| RP11-94I2.4 | 2.54 | 4.10 | 5.91E-20 | 8.65E-19 |
| LINC01842 | 3.50 | 5.79 | 6.30E-20 | 9.21E-19 |
| LINC01559 | 6.54 | 8.20 | 7.42E-20 | 1.08E-18 |
| RP11-485G7.6 | 2.08 | 4.31 | 9.00E-20 | 1.30E-18 |
| RP11-66B24.7 | 2.97 | 7.29 | 9.01E-20 | 1.30E-18 |
| HOTAIR | 6.79 | 6.59 | 1.04E-19 | 1.50E-18 |
| RP11-1134I14.8 | 2.29 | 6.81 | 1.05E-19 | 1.52E-18 |
| AC006159.5 | -2.13 | 3.10 | 1.06E-19 | 1.52E-18 |
| RP11-462L8.1 | 3.93 | 6.42 | 1.23E-19 | 1.76E-18 |
| RP11-473C18.3 | 2.24 | 3.37 | 1.31E-19 | 1.88E-18 |
| AC004221.2 | 2.90 | 3.52 | 1.49E-19 | 2.12E-18 |
| LINC01168 | -2.39 | 3.07 | 1.57E-19 | 2.23E-18 |
| AC053503.4 | 2.30 | 4.65 | 1.91E-19 | 2.70E-18 |
| RP11-863P13.3 | 2.59 | 5.29 | 2.24E-19 | 3.14E-18 |
| CALML3-AS1 | 3.26 | 6.32 | 2.47E-19 | 3.44E-18 |
| RP5-940J5.3 | 2.05 | 4.11 | 2.51E-19 | 3.50E-18 |
| CTD-2529O21.1 | 2.96 | 3.62 | 2.55E-19 | 3.55E-18 |
| RP11-8L2.1 | 6.00 | 5.43 | 2.70E-19 | 3.74E-18 |
| STEAP2-AS1 | 3.15 | 3.82 | 2.82E-19 | 3.89E-18 |
| RP13-577H12.2 | -2.77 | 3.57 | 3.15E-19 | 4.32E-18 |
| RNF144A-AS1 | 2.21 | 7.43 | 3.26E-19 | 4.44E-18 |
| RP11-47I22.2 | 2.44 | 5.83 | 3.46E-19 | 4.70E-18 |
| LINC01166 | -2.47 | 3.30 | 3.61E-19 | 4.91E-18 |
| RP11-395B7.2 | 3.94 | 4.93 | 3.74E-19 | 5.06E-18 |
| LINC02086 | 3.99 | 6.83 | 3.81E-19 | 5.14E-18 |
| AC093390.1 | -2.13 | 3.03 | 3.97E-19 | 5.36E-18 |
| RP11-661A12.9 | 2.29 | 4.97 | 4.08E-19 | 5.50E-18 |
| AC024592.9 | 2.39 | 5.25 | 4.15E-19 | 5.58E-18 |
| BARX1-AS1 | 6.54 | 5.97 | 4.69E-19 | 6.28E-18 |
| RP11-10A14.4 | 2.55 | 5.97 | 4.73E-19 | 6.33E-18 |
| DUXAP8 | 2.59 | 8.59 | 5.01E-19 | 6.69E-18 |
| RP11-187E13.2 | 3.46 | 3.66 | 5.41E-19 | 7.21E-18 |
| LUCAT1 | 3.03 | 8.85 | 5.91E-19 | 7.82E-18 |
| CASC9 | 5.49 | 8.51 | 5.98E-19 | 7.89E-18 |
| RP5-1056H1.2 | 2.04 | 6.09 | 6.01E-19 | 7.92E-18 |
| RP5-965G21.4 | 2.04 | 6.82 | 6.07E-19 | 7.98E-18 |
| PCAT7 | 2.37 | 5.97 | 6.33E-19 | 8.30E-18 |
| PKP4-AS1 | 2.38 | 7.08 | 6.72E-19 | 8.80E-18 |
| C20orf197 | 3.04 | 8.00 | 7.74E-19 | 1.01E-17 |
| RP11-209K10.2 | 6.12 | 4.77 | 8.10E-19 | 1.06E-17 |
| RP11-818F20.5 | 4.25 | 5.89 | 8.50E-19 | 1.11E-17 |
| UMODL1-AS1 | -2.04 | 6.16 | 8.93E-19 | 1.16E-17 |
| LINC00460 | 4.44 | 8.34 | 8.94E-19 | 1.16E-17 |
| RP11-429J17.5 | 2.92 | 4.26 | 1.08E-18 | 1.39E-17 |
| AC002076.10 | 4.11 | 4.69 | 1.43E-18 | 1.82E-17 |
| HOXC13-AS | 5.97 | 5.50 | 1.52E-18 | 1.93E-17 |
| CTD-3194G12.2 | 3.01 | 3.76 | 1.52E-18 | 1.93E-17 |
| AC004870.4 | 4.55 | 5.74 | 1.54E-18 | 1.94E-17 |
| CTD-2555C10.3 | 2.99 | 6.18 | 1.56E-18 | 1.97E-17 |
| RP4-676L2.1 | 3.74 | 4.19 | 1.78E-18 | 2.24E-17 |
| LINC02163 | 6.03 | 4.91 | 1.91E-18 | 2.40E-17 |
| CTD-2139B15.5 | 7.27 | 5.83 | 2.01E-18 | 2.52E-17 |
| RP11-187E13.1 | 4.50 | 4.92 | 2.18E-18 | 2.71E-17 |
| RP11-132A1.4 | 2.28 | 7.74 | 2.23E-18 | 2.78E-17 |
| RP11-545P7.9 | 2.11 | 4.39 | 2.26E-18 | 2.81E-17 |
| AC112721.2 | 3.28 | 4.59 | 2.32E-18 | 2.89E-17 |
| RP11-626G11.4 | 2.22 | 5.83 | 2.55E-18 | 3.17E-17 |
| U47924.29 | 2.15 | 3.72 | 2.56E-18 | 3.17E-17 |
| TRPM2-AS | 3.45 | 6.94 | 2.95E-18 | 3.62E-17 |
| LINC02133 | 4.39 | 5.49 | 3.65E-18 | 4.43E-17 |
| FAM83C-AS1 | 2.27 | 3.46 | 4.16E-18 | 5.02E-17 |
| LINC02122 | 4.62 | 5.13 | 4.37E-18 | 5.24E-17 |
| LINC01597 | 4.14 | 7.89 | 4.54E-18 | 5.42E-17 |
| RP11-380J14.1 | 4.98 | 5.19 | 4.87E-18 | 5.80E-17 |
| LINC00858 | 5.12 | 6.93 | 4.99E-18 | 5.92E-17 |
| RP11-161I6.2 | 3.89 | 5.52 | 5.00E-18 | 5.93E-17 |
| RP11-150C16.1 | 3.17 | 5.49 | 5.57E-18 | 6.60E-17 |
| AC079630.2 | -2.31 | 6.84 | 5.65E-18 | 6.69E-17 |
| AP001626.2 | 2.65 | 4.96 | 6.05E-18 | 7.13E-17 |
| AC012360.6 | 2.17 | 6.16 | 6.33E-18 | 7.45E-17 |
| RP11-190J1.3 | 5.53 | 4.84 | 6.95E-18 | 8.13E-17 |
| SATB2-AS1 | 2.84 | 4.31 | 7.06E-18 | 8.24E-17 |
| LINC02029 | 3.99 | 4.23 | 7.19E-18 | 8.37E-17 |
| RP11-1069G10.2 | 3.83 | 4.05 | 7.68E-18 | 8.91E-17 |
| AC009262.2 | 4.27 | 3.16 | 7.72E-18 | 8.95E-17 |
| RP11-1084E5.1 | 2.76 | 4.73 | 8.21E-18 | 9.48E-17 |
| FIRRE | 2.61 | 6.66 | 9.43E-18 | 1.08E-16 |
| RP11-624L4.1 | 2.37 | 6.71 | 9.48E-18 | 1.09E-16 |
| RP11-1055B8.9 | 2.35 | 4.66 | 9.51E-18 | 1.09E-16 |
| LINC00501 | 5.30 | 5.75 | 1.07E-17 | 1.22E-16 |
| AC011298.2 | 7.02 | 6.77 | 1.30E-17 | 1.48E-16 |
| DRAIC | 3.72 | 9.86 | 1.40E-17 | 1.59E-16 |
| RP3-323A16.1 | 2.82 | 7.19 | 1.48E-17 | 1.67E-16 |
| RP11-357H14.17 | 4.41 | 7.07 | 1.57E-17 | 1.77E-16 |
| AC123023.1 | -2.25 | 3.73 | 1.59E-17 | 1.79E-16 |
| RP11-66D17.3 | 2.77 | 3.40 | 2.08E-17 | 2.32E-16 |
| CTD-2620I22.3 | 3.36 | 4.58 | 2.15E-17 | 2.39E-16 |
| RP11-10N16.3 | 2.38 | 3.72 | 2.23E-17 | 2.47E-16 |
| LINC02156 | 3.13 | 3.67 | 2.31E-17 | 2.56E-16 |
| RP11-103B5.4 | 2.39 | 5.79 | 2.35E-17 | 2.61E-16 |
| CASC8 | 3.27 | 6.71 | 2.37E-17 | 2.62E-16 |
| MYO16-AS1 | -2.38 | 6.18 | 2.64E-17 | 2.92E-16 |
| RP11-466P24.6 | -2.76 | 3.37 | 2.91E-17 | 3.20E-16 |
| RP11-268F1.3 | -2.32 | 4.87 | 3.03E-17 | 3.32E-16 |
| RP11-108K3.1 | 4.18 | 4.30 | 3.24E-17 | 3.55E-16 |
| LINC00707 | 4.60 | 7.37 | 3.34E-17 | 3.65E-16 |
| RP11-54O7.16 | 3.12 | 3.91 | 3.39E-17 | 3.70E-16 |
| LINC01970 | 2.06 | 4.72 | 3.64E-17 | 3.97E-16 |
| AC128709.3 | -2.34 | 3.35 | 3.67E-17 | 3.99E-16 |
| LINC00973 | 6.23 | 7.56 | 3.77E-17 | 4.09E-16 |
| RP11-391H12.8 | 2.05 | 4.10 | 3.96E-17 | 4.28E-16 |
| RP11-25H12.1 | 5.18 | 3.88 | 3.97E-17 | 4.29E-16 |
| LINC01748 | 4.87 | 6.45 | 4.01E-17 | 4.32E-16 |
| RP11-84D1.1 | 2.15 | 4.15 | 4.75E-17 | 5.11E-16 |
| RMST | -2.09 | 5.57 | 4.84E-17 | 5.18E-16 |
| MAFA-AS1 | 4.08 | 4.97 | 5.08E-17 | 5.43E-16 |
| RP11-768B22.2 | 2.41 | 4.92 | 5.86E-17 | 6.26E-16 |
| LINC01979 | 2.35 | 5.06 | 6.18E-17 | 6.57E-16 |
| RP11-29H23.4 | 2.23 | 3.30 | 6.39E-17 | 6.77E-16 |
| CTD-2591A6.2 | 6.46 | 4.71 | 6.78E-17 | 7.18E-16 |
| RP4-712E4.2 | 4.14 | 2.94 | 6.91E-17 | 7.30E-16 |
| AC006946.16 | 2.59 | 6.03 | 6.98E-17 | 7.36E-16 |
| RP11-108K3.2 | 4.39 | 4.09 | 7.07E-17 | 7.45E-16 |
| CTD-2256P15.1 | 2.24 | 3.30 | 7.23E-17 | 7.61E-16 |
| CTD-2008P7.1 | 3.09 | 3.49 | 7.68E-17 | 8.06E-16 |
| FLJ12825 | 2.06 | 5.25 | 7.78E-17 | 8.16E-16 |
| LINC01926 | 3.44 | 3.44 | 7.97E-17 | 8.34E-16 |
| RP11-167H9.4 | 4.73 | 4.61 | 8.12E-17 | 8.48E-16 |
| LA16c-321D4.2 | 2.45 | 5.25 | 8.36E-17 | 8.71E-16 |
| AC108868.5 | -2.03 | 2.99 | 1.02E-16 | 1.06E-15 |
| NAALADL2-AS2 | 5.10 | 5.50 | 1.33E-16 | 1.36E-15 |
| RP11-785D18.3 | 5.00 | 7.95 | 1.33E-16 | 1.36E-15 |
| AC104699.1 | 2.21 | 5.77 | 1.41E-16 | 1.44E-15 |
| SALRNA1 | 2.60 | 5.30 | 1.43E-16 | 1.46E-15 |
| NOVA1-AS1 | 5.84 | 6.31 | 1.44E-16 | 1.47E-15 |
| RP11-625L16.3 | 3.27 | 3.58 | 1.46E-16 | 1.49E-15 |
| STPG3-AS1 | 2.07 | 5.80 | 1.53E-16 | 1.56E-15 |
| AC108676.1 | 2.66 | 6.04 | 1.59E-16 | 1.61E-15 |
| LINC01169 | -2.03 | 4.44 | 1.63E-16 | 1.65E-15 |
| AC073316.2 | 3.84 | 4.85 | 1.68E-16 | 1.70E-15 |
| LINC00466 | 4.30 | 3.47 | 1.79E-16 | 1.81E-15 |
| RP11-254F7.4 | 2.50 | 3.40 | 1.83E-16 | 1.84E-15 |
| FAM222A-AS1 | 2.83 | 5.44 | 1.86E-16 | 1.87E-15 |
| CTD-2523D13.2 | 2.19 | 4.93 | 2.05E-16 | 2.05E-15 |
| DLX6-AS1 | 4.48 | 7.21 | 2.10E-16 | 2.10E-15 |
| RP11-124N19.3 | 3.33 | 4.49 | 2.11E-16 | 2.10E-15 |
| CTD-2196E14.5 | 2.57 | 3.25 | 2.36E-16 | 2.34E-15 |
| HOXC-AS3 | 5.81 | 5.54 | 2.40E-16 | 2.37E-15 |
| ELFN1-AS1 | 3.89 | 6.47 | 2.59E-16 | 2.56E-15 |
| LA16c-329F2.2 | 2.02 | 4.14 | 3.49E-16 | 3.41E-15 |
| RP11-63E9.1 | 5.00 | 6.33 | 3.75E-16 | 3.64E-15 |
| LINC01969 | 2.93 | 3.50 | 3.89E-16 | 3.77E-15 |
| LINC01214 | 5.76 | 5.27 | 3.97E-16 | 3.84E-15 |
| ERVH48-1 | 4.76 | 9.01 | 4.03E-16 | 3.90E-15 |
| LINC01561 | 3.19 | 4.50 | 4.47E-16 | 4.31E-15 |
| TTLL11-IT1 | 3.17 | 4.61 | 4.52E-16 | 4.35E-15 |
| RP11-414H23.3 | 3.96 | 3.52 | 5.31E-16 | 5.09E-15 |
| HAGLROS | 2.51 | 6.38 | 5.68E-16 | 5.43E-15 |
| RP11-14C10.6 | 3.69 | 4.01 | 6.02E-16 | 5.75E-15 |
| RP11-499O7.7 | 3.90 | 6.43 | 6.40E-16 | 6.08E-15 |
| LINC00519 | 2.45 | 5.18 | 6.75E-16 | 6.40E-15 |
| RP11-46A10.2 | 2.71 | 4.24 | 6.98E-16 | 6.59E-15 |
| RP11-320N21.2 | 2.42 | 3.13 | 8.28E-16 | 7.78E-15 |
| RP11-356K23.2 | -2.01 | 3.66 | 8.48E-16 | 7.96E-15 |
| LINP1 | 4.34 | 7.07 | 8.56E-16 | 8.02E-15 |
| LINC01436 | 3.21 | 9.74 | 9.71E-16 | 9.02E-15 |
| RP11-13P5.2 | 2.23 | 5.00 | 1.01E-15 | 9.34E-15 |
| RP11-38M8.1 | 2.44 | 6.10 | 1.04E-15 | 9.61E-15 |
| CTD-3035D6.2 | 2.55 | 4.86 | 1.31E-15 | 1.20E-14 |
| AL109761.5 | 2.55 | 4.51 | 1.33E-15 | 1.22E-14 |
| LINC01971 | 3.17 | 3.63 | 1.33E-15 | 1.22E-14 |
| RP11-302F12.3 | 5.01 | 4.94 | 1.38E-15 | 1.26E-14 |
| CH507-42P11.6 | 3.39 | 3.67 | 1.38E-15 | 1.26E-14 |
| RP11-346D19.1 | 5.18 | 3.86 | 1.66E-15 | 1.51E-14 |
| CTC-499J9.1 | 4.72 | 4.40 | 1.87E-15 | 1.68E-14 |
| LA16c-325D7.1 | 2.13 | 4.99 | 1.91E-15 | 1.72E-14 |
| CCAT1 | 5.64 | 7.39 | 1.98E-15 | 1.77E-14 |
| RP3-523K23.2 | 3.40 | 8.10 | 2.03E-15 | 1.82E-14 |
| RP11-80H5.2 | 3.08 | 3.01 | 2.04E-15 | 1.82E-14 |
| RP11-309M7.1 | 5.70 | 4.70 | 2.10E-15 | 1.88E-14 |
| RP11-644C3.1 | 3.44 | 2.99 | 2.34E-15 | 2.07E-14 |
| RP11-415J8.5 | 2.28 | 3.63 | 2.41E-15 | 2.14E-14 |
| RP11-44F21.5 | 2.28 | 8.30 | 2.42E-15 | 2.15E-14 |
| RP11-1C8.4 | 5.36 | 4.64 | 2.47E-15 | 2.19E-14 |
| CTD-2021H9.3 | 5.05 | 6.58 | 2.70E-15 | 2.38E-14 |
| RP11-383J24.1 | 4.48 | 3.98 | 2.79E-15 | 2.46E-14 |
| KB-1991G8.1 | 2.73 | 5.71 | 2.87E-15 | 2.53E-14 |
| LINC01468 | 5.90 | 5.55 | 2.88E-15 | 2.53E-14 |
| CTD-2509G16.2 | 2.68 | 3.53 | 2.93E-15 | 2.57E-14 |
| LINC01792 | 3.48 | 3.52 | 2.94E-15 | 2.58E-14 |
| LINC01978 | 2.15 | 4.76 | 3.20E-15 | 2.80E-14 |
| CTD-2330J20.2 | -2.12 | 2.73 | 3.22E-15 | 2.81E-14 |
| RP11-231I16.1 | 2.13 | 5.35 | 3.38E-15 | 2.94E-14 |
| LVCAT1 | 3.91 | 6.12 | 3.40E-15 | 2.95E-14 |
| RP11-657O9.1 | 3.89 | 5.26 | 3.53E-15 | 3.06E-14 |
| AC012501.2 | 5.12 | 3.53 | 3.62E-15 | 3.14E-14 |
| RP11-67K19.3 | 4.67 | 5.46 | 3.77E-15 | 3.26E-14 |
| XXbac-BPG308K3.5 | 2.69 | 4.31 | 3.95E-15 | 3.41E-14 |
| RP11-1260E13.1 | 3.62 | 7.17 | 4.29E-15 | 3.69E-14 |
| EGOT | 2.34 | 6.03 | 4.32E-15 | 3.71E-14 |
| LINC00491 | 5.61 | 5.82 | 4.50E-15 | 3.85E-14 |
| LINC02043 | 2.42 | 3.65 | 4.78E-15 | 4.09E-14 |
| LINC01587 | 2.90 | 4.79 | 4.98E-15 | 4.26E-14 |
| LINC01305 | 5.02 | 4.40 | 5.08E-15 | 4.34E-14 |
| AC144450.1 | 2.66 | 5.42 | 5.16E-15 | 4.40E-14 |
| RP11-191L9.4 | 5.71 | 4.45 | 5.17E-15 | 4.40E-14 |
| CH17-360D5.3 | -2.04 | 6.80 | 5.22E-15 | 4.44E-14 |
| LINC01775 | 2.35 | 3.33 | 5.54E-15 | 4.70E-14 |
| MIR137HG | 4.97 | 4.89 | 5.73E-15 | 4.85E-14 |
| AC006262.6 | 2.45 | 5.52 | 5.98E-15 | 5.04E-14 |
| LINC01234 | 5.51 | 7.11 | 6.18E-15 | 5.20E-14 |
| LINC01615 | 2.30 | 5.51 | 6.28E-15 | 5.28E-14 |
| CTC-480C2.1 | 5.69 | 4.31 | 6.72E-15 | 5.63E-14 |
| XX-C2158C6.3 | 3.95 | 3.71 | 6.80E-15 | 5.70E-14 |
| LINC01564 | 2.96 | 6.33 | 6.84E-15 | 5.72E-14 |
| RP11-95M15.1 | 4.22 | 4.61 | 6.94E-15 | 5.80E-14 |
| LINC00941 | 3.33 | 7.36 | 7.03E-15 | 5.87E-14 |
| RP11-114B7.6 | 4.01 | 3.91 | 7.49E-15 | 6.24E-14 |
| AC141930.2 | 3.16 | 3.52 | 7.52E-15 | 6.26E-14 |
| RP11-469A15.2 | 2.81 | 5.78 | 7.96E-15 | 6.59E-14 |
| MIR9-3HG | 2.72 | 8.76 | 8.10E-15 | 6.70E-14 |
| RP11-556E13.1 | 2.05 | 4.68 | 8.23E-15 | 6.80E-14 |
| RP11-114H23.2 | 3.21 | 4.59 | 8.23E-15 | 6.80E-14 |
| LINC00626 | 3.51 | 3.85 | 8.54E-15 | 7.04E-14 |
| RP11-353N14.1 | 3.41 | 3.04 | 8.81E-15 | 7.24E-14 |
| RP11-275I4.2 | 2.63 | 3.38 | 9.46E-15 | 7.74E-14 |
| LINC01287 | 5.76 | 7.40 | 9.61E-15 | 7.86E-14 |
| RP11-417L19.2 | 3.79 | 3.70 | 9.63E-15 | 7.87E-14 |
| AC005537.2 | 3.53 | 5.94 | 1.07E-14 | 8.71E-14 |
| LINC01393 | 2.07 | 5.24 | 1.07E-14 | 8.73E-14 |
| LINC02065 | 3.28 | 3.37 | 1.11E-14 | 9.05E-14 |
| RP11-474D1.3 | 7.52 | 8.26 | 1.13E-14 | 9.15E-14 |
| CTD-2566J3.1 | 5.45 | 6.24 | 1.13E-14 | 9.17E-14 |
| LINC01968 | 2.61 | 3.29 | 1.15E-14 | 9.31E-14 |
| AC112721.1 | 2.87 | 3.99 | 1.25E-14 | 1.01E-13 |
| RP11-297L17.2 | 5.38 | 4.31 | 1.28E-14 | 1.03E-13 |
| RP11-771K4.1 | 3.35 | 4.88 | 1.36E-14 | 1.10E-13 |
| CTD-2162K18.4 | 2.93 | 5.96 | 1.47E-14 | 1.18E-13 |
| SRGAP3-AS2 | -2.32 | 8.25 | 1.51E-14 | 1.21E-13 |
| AC144450.2 | 2.58 | 5.63 | 1.52E-14 | 1.22E-13 |
| POU6F2-AS2 | 6.19 | 5.18 | 1.67E-14 | 1.33E-13 |
| AC012531.25 | 3.43 | 4.69 | 1.75E-14 | 1.39E-13 |
| RP11-96H17.1 | 4.34 | 4.55 | 1.81E-14 | 1.43E-13 |
| RP11-627G23.1 | 2.94 | 9.43 | 1.83E-14 | 1.45E-13 |
| THRA1/BTR | 4.09 | 4.46 | 1.83E-14 | 1.45E-13 |
| CTD-2129N1.1 | 2.39 | 3.20 | 1.90E-14 | 1.49E-13 |
| LINC01843 | 2.36 | 7.04 | 1.91E-14 | 1.51E-13 |
| LINC01876 | 2.17 | 6.94 | 1.93E-14 | 1.52E-13 |
| LINC01213 | 3.83 | 3.90 | 2.07E-14 | 1.62E-13 |
| LINC00165 | -2.40 | 3.97 | 2.08E-14 | 1.62E-13 |
| CTA-384D8.31 | 2.77 | 5.79 | 2.17E-14 | 1.70E-13 |
| CTC-321K16.1 | 3.71 | 4.19 | 2.22E-14 | 1.73E-13 |
| RP11-3B12.5 | 4.92 | 5.14 | 2.24E-14 | 1.75E-13 |
| AC114803.3 | 4.17 | 2.95 | 2.31E-14 | 1.79E-13 |
| APCDD1L-AS1 | 3.22 | 6.24 | 2.36E-14 | 1.83E-13 |
| RP11-332J15.4 | 2.37 | 3.72 | 2.40E-14 | 1.86E-13 |
| RP11-65D17.1 | 3.28 | 4.54 | 2.49E-14 | 1.93E-13 |
| CTA-246H3.12 | 3.49 | 3.20 | 2.54E-14 | 1.97E-13 |
| HOXA11-AS | 4.35 | 5.31 | 2.57E-14 | 1.98E-13 |
| RP11-174G6.1 | 2.21 | 4.58 | 2.75E-14 | 2.12E-13 |
| FNDC1-IT1 | 2.97 | 3.10 | 2.88E-14 | 2.21E-13 |
| RP4-536B24.4 | 2.95 | 3.26 | 2.88E-14 | 2.21E-13 |
| LINC01518 | 5.98 | 4.61 | 2.98E-14 | 2.29E-13 |
| CTC-327F10.5 | 3.54 | 3.20 | 3.19E-14 | 2.44E-13 |
| RP11-547D24.1 | 2.03 | 4.30 | 3.22E-14 | 2.46E-13 |
| RP6-114E22.1 | 4.79 | 5.30 | 3.23E-14 | 2.46E-13 |
| CTD-2066L21.3 | 4.19 | 4.30 | 3.49E-14 | 2.64E-13 |
| RP11-497G19.1 | 4.74 | 7.34 | 3.66E-14 | 2.77E-13 |
| LINC02015 | 2.69 | 6.78 | 3.81E-14 | 2.88E-13 |
| RP11-664H17.1 | 3.27 | 4.70 | 4.24E-14 | 3.19E-13 |
| UG0898H09 | 4.66 | 6.26 | 4.45E-14 | 3.35E-13 |
| RP11-379K22.3 | 4.01 | 5.45 | 4.57E-14 | 3.44E-13 |
| MUC2 | 5.83 | 9.36 | 4.95E-14 | 3.71E-13 |
| RP11-334A14.8 | 3.31 | 6.05 | 4.97E-14 | 3.72E-13 |
| RP11-237N19.3 | 3.79 | 3.52 | 5.65E-14 | 4.22E-13 |
| HHATL-AS1 | -2.01 | 4.04 | 5.73E-14 | 4.27E-13 |
| AF127577.8 | 6.00 | 5.16 | 6.11E-14 | 4.53E-13 |
| LEMD1-AS1 | 2.21 | 4.72 | 6.30E-14 | 4.68E-13 |
| CTA-280A3.2 | 4.60 | 3.37 | 6.53E-14 | 4.83E-13 |
| IGFL2-AS1 | 4.14 | 5.67 | 6.70E-14 | 4.94E-13 |
| RP11-114M1.2 | 4.59 | 3.46 | 6.82E-14 | 5.03E-13 |
| LINC01611 | 5.68 | 4.08 | 7.53E-14 | 5.53E-13 |
| LINC00682 | 5.33 | 4.02 | 7.59E-14 | 5.56E-13 |
| RP11-673E11.2 | -2.03 | 3.02 | 7.72E-14 | 5.64E-13 |
| RP11-231D20.2 | 2.49 | 4.16 | 7.78E-14 | 5.68E-13 |
| RP4-738P15.1 | 3.38 | 4.87 | 7.96E-14 | 5.80E-13 |
| RP11-328K4.1 | 4.27 | 4.20 | 7.98E-14 | 5.81E-13 |
| LL22NC03-N64E9.1 | 3.17 | 3.42 | 8.48E-14 | 6.16E-13 |
| RP1-140K8.5 | 2.64 | 7.68 | 8.51E-14 | 6.18E-13 |
| RP11-376O6.2 | 3.52 | 2.86 | 8.74E-14 | 6.33E-13 |
| RP11-25L3.3 | 4.01 | 3.48 | 8.87E-14 | 6.41E-13 |
| RP11-1042B17.3 | 3.09 | 3.49 | 9.16E-14 | 6.62E-13 |
| RP11-424M24.5 | -2.10 | 5.05 | 1.01E-13 | 7.29E-13 |
| RP11-555J4.3 | 4.66 | 3.33 | 1.09E-13 | 7.85E-13 |
| LINC00518 | 4.48 | 3.27 | 1.14E-13 | 8.16E-13 |
| LINC00676 | 10.04 | 8.72 | 1.17E-13 | 8.38E-13 |
| AC092484.1 | 5.00 | 5.16 | 1.38E-13 | 9.82E-13 |
| RP11-1C8.7 | 4.85 | 4.30 | 1.43E-13 | 1.02E-12 |
| LL22NC03-63E9.3 | 3.32 | 3.43 | 1.68E-13 | 1.19E-12 |
| RP11-60A8.1 | 5.04 | 5.58 | 1.82E-13 | 1.28E-12 |
| CTB-55O6.4 | 2.69 | 6.06 | 1.95E-13 | 1.37E-12 |
| CTD-2083E4.7 | 3.11 | 3.64 | 2.17E-13 | 1.51E-12 |
| RP5-823G15.5 | 3.27 | 4.50 | 2.20E-13 | 1.53E-12 |
| CTD-3064H18.4 | 3.35 | 5.72 | 2.21E-13 | 1.54E-12 |
| RP11-730K11.1 | 2.09 | 3.81 | 2.25E-13 | 1.56E-12 |
| HOXC-AS1 | 3.21 | 4.78 | 2.45E-13 | 1.70E-12 |
| SYNPR-AS1 | 2.06 | 6.59 | 2.51E-13 | 1.74E-12 |
| RP11-527D7.1 | 4.01 | 4.22 | 2.56E-13 | 1.77E-12 |
| RP11-893F2.5 | 2.39 | 3.73 | 2.66E-13 | 1.84E-12 |
| RP11-359E19.2 | 4.47 | 7.76 | 2.66E-13 | 1.84E-12 |
| AC096579.15 | 2.13 | 5.29 | 2.76E-13 | 1.90E-12 |
| RP11-405A12.2 | 2.77 | 5.20 | 2.87E-13 | 1.97E-12 |
| AF015262.2 | 3.60 | 3.07 | 2.89E-13 | 1.99E-12 |
| C10orf91 | 2.01 | 6.16 | 2.91E-13 | 2.00E-12 |
| AC018890.6 | 3.15 | 6.31 | 2.98E-13 | 2.04E-12 |
| RP11-445O3.1 | 4.49 | 3.14 | 3.26E-13 | 2.22E-12 |
| LINC00567 | 3.79 | 5.48 | 3.33E-13 | 2.27E-12 |
| LINC01633 | 5.10 | 3.68 | 3.74E-13 | 2.53E-12 |
| RP11-326C3.2 | 2.12 | 10.12 | 4.03E-13 | 2.72E-12 |
| DPP10-AS1 | 3.19 | 7.28 | 4.28E-13 | 2.88E-12 |
| PANCR | 5.66 | 4.09 | 4.37E-13 | 2.94E-12 |
| RP11-90L1.8 | 2.09 | 6.51 | 4.50E-13 | 3.02E-12 |
| RP11-209E8.1 | 4.95 | 3.59 | 4.89E-13 | 3.26E-12 |
| AC024560.2 | 2.41 | 4.48 | 4.91E-13 | 3.28E-12 |
| LA16c-349E10.1 | 2.14 | 4.53 | 4.93E-13 | 3.28E-12 |
| RP11-156K13.2 | 3.79 | 2.97 | 5.28E-13 | 3.51E-12 |
| BCAR4 | 6.42 | 6.48 | 5.45E-13 | 3.62E-12 |
| LINC00483 | 4.25 | 4.38 | 5.68E-13 | 3.77E-12 |
| RP1-35C21.2 | 3.78 | 3.76 | 5.71E-13 | 3.78E-12 |
| SSTR5-AS1 | 5.28 | 5.68 | 6.13E-13 | 4.05E-12 |
| LINC01807 | 5.12 | 4.72 | 6.82E-13 | 4.47E-12 |
| RP11-432I5.2 | 2.06 | 3.50 | 7.23E-13 | 4.73E-12 |
| RP11-10A14.9 | 2.36 | 3.97 | 7.36E-13 | 4.81E-12 |
| AP003900.6 | 5.87 | 4.07 | 7.54E-13 | 4.92E-12 |
| RP11-815M8.1 | 2.16 | 8.00 | 7.58E-13 | 4.94E-12 |
| RP11-227H15.4 | 3.35 | 4.38 | 7.84E-13 | 5.11E-12 |
| AC005330.2 | 2.37 | 4.76 | 7.93E-13 | 5.16E-12 |
| CTD-2342N23.3 | 2.10 | 4.26 | 8.04E-13 | 5.21E-12 |
| RP11-242J7.1 | 3.20 | 5.18 | 8.10E-13 | 5.25E-12 |
| AC008268.1 | -2.14 | 9.62 | 8.47E-13 | 5.46E-12 |
| RP11-493L12.3 | 2.33 | 3.93 | 8.94E-13 | 5.75E-12 |
| RP11-445O3.2 | 5.15 | 4.11 | 9.62E-13 | 6.18E-12 |
| AC092535.3 | 2.23 | 4.85 | 9.62E-13 | 6.18E-12 |
| AC083884.8 | 2.23 | 5.61 | 1.12E-12 | 7.19E-12 |
| CTC-276P9.4 | 2.97 | 3.08 | 1.14E-12 | 7.25E-12 |
| GS1-594A7.3 | 2.80 | 4.21 | 1.20E-12 | 7.66E-12 |
| RP11-73M7.1 | 2.13 | 5.07 | 1.21E-12 | 7.69E-12 |
| LINC01511 | 5.75 | 6.99 | 1.24E-12 | 7.89E-12 |
| LINC01385 | 5.50 | 3.97 | 1.36E-12 | 8.62E-12 |
| RP11-829H16.3 | 2.31 | 4.43 | 1.40E-12 | 8.82E-12 |
| AC021218.2 | 3.14 | 6.57 | 1.41E-12 | 8.89E-12 |
| RP11-108K3.3 | 3.30 | 2.57 | 1.45E-12 | 9.14E-12 |
| AP000696.2 | 4.04 | 3.53 | 1.52E-12 | 9.54E-12 |
| FLJ22447 | 2.89 | 8.54 | 1.52E-12 | 9.57E-12 |
| RP5-984P4.6 | 5.51 | 4.26 | 1.53E-12 | 9.61E-12 |
| RP11-352B15.2 | 5.98 | 4.50 | 1.72E-12 | 1.08E-11 |
| RP11-855O10.2 | 2.31 | 5.36 | 1.80E-12 | 1.12E-11 |
| PP14571 | 2.01 | 7.43 | 1.89E-12 | 1.18E-11 |
| CTC-501O10.1 | 2.98 | 3.60 | 1.98E-12 | 1.23E-11 |
| RP11-116O18.1 | 4.87 | 9.37 | 2.24E-12 | 1.38E-11 |
| AC011738.4 | 2.18 | 3.71 | 2.35E-12 | 1.44E-11 |
| RP11-418I22.2 | -2.08 | 2.77 | 2.37E-12 | 1.46E-11 |
| MIR31HG | 3.54 | 5.63 | 2.50E-12 | 1.54E-11 |
| CTD-2576D5.4 | 2.59 | 6.22 | 2.55E-12 | 1.56E-11 |
| RP11-445O3.3 | 5.04 | 4.92 | 2.70E-12 | 1.65E-11 |
| POU6F2-AS1 | 4.18 | 3.17 | 2.71E-12 | 1.65E-11 |
| RP11-101E5.1 | -2.26 | 3.38 | 2.85E-12 | 1.74E-11 |
| RP11-310P5.1 | 2.81 | 2.68 | 2.89E-12 | 1.76E-11 |
| AC009236.2 | 3.56 | 2.67 | 2.95E-12 | 1.79E-11 |
| RP1-27K12.2 | 5.68 | 9.49 | 2.96E-12 | 1.80E-11 |
| RP11-503C24.6 | 4.47 | 5.18 | 3.26E-12 | 1.97E-11 |
| RP11-535A19.1 | 2.53 | 3.17 | 3.32E-12 | 2.01E-11 |
| LL22NC03-102D1.18 | 3.16 | 2.75 | 3.40E-12 | 2.05E-11 |
| RP11-395N3.1 | 2.74 | 4.20 | 3.40E-12 | 2.05E-11 |
| RP11-395E19.6 | -2.20 | 3.92 | 3.41E-12 | 2.05E-11 |
| MIR548XHG | 6.39 | 5.01 | 3.50E-12 | 2.10E-11 |
| CTD-2535I10.1 | 4.45 | 3.95 | 3.58E-12 | 2.14E-11 |
| AC079466.1 | 7.05 | 6.10 | 3.66E-12 | 2.19E-11 |
| LINC01804 | 5.01 | 4.91 | 3.69E-12 | 2.21E-11 |
| LINC00824 | 4.20 | 6.09 | 3.71E-12 | 2.22E-11 |
| KB-1615E4.3 | 2.29 | 3.32 | 3.84E-12 | 2.29E-11 |
| RP11-279F6.2 | 5.08 | 6.16 | 3.89E-12 | 2.32E-11 |
| RP11-180I4.2 | 2.44 | 3.08 | 4.02E-12 | 2.40E-11 |
| LINC00251 | 4.16 | 2.95 | 4.15E-12 | 2.47E-11 |
| RP5-1119A7.14 | 2.78 | 5.04 | 4.38E-12 | 2.60E-11 |
| MIR2052HG | 3.69 | 4.94 | 4.53E-12 | 2.68E-11 |
| LINC01687 | 4.59 | 3.20 | 4.62E-12 | 2.73E-11 |
| RP11-144A16.8 | -2.26 | 3.70 | 5.04E-12 | 2.97E-11 |
| HOXA10-AS | 4.21 | 3.83 | 5.37E-12 | 3.16E-11 |
| RP11-396O20.2 | 3.88 | 4.09 | 5.49E-12 | 3.23E-11 |
| LL22NC03-N14H11.1 | 2.01 | 4.72 | 5.58E-12 | 3.27E-11 |
| RP11-519M16.1 | 5.17 | 3.56 | 5.69E-12 | 3.33E-11 |
| RP11-800A18.4 | 2.68 | 4.60 | 5.73E-12 | 3.36E-11 |
| FLJ16779 | 2.58 | 5.85 | 5.84E-12 | 3.42E-11 |
| AC069277.2 | 4.17 | 4.96 | 5.95E-12 | 3.48E-11 |
| AC006548.28 | 2.54 | 5.21 | 6.00E-12 | 3.51E-11 |
| RP11-146I2.1 | 2.12 | 3.99 | 6.00E-12 | 3.51E-11 |
| AC008063.2 | 2.05 | 5.65 | 6.05E-12 | 3.53E-11 |
| RP11-495P10.5 | 2.93 | 3.28 | 6.41E-12 | 3.74E-11 |
| RP11-1007I13.4 | 3.36 | 3.53 | 6.47E-12 | 3.77E-11 |
| DSCAM-AS1 | 7.97 | 10.52 | 6.56E-12 | 3.81E-11 |
| LINC01021 | 4.05 | 5.93 | 6.74E-12 | 3.91E-11 |
| RP11-758M4.4 | 4.91 | 5.83 | 6.85E-12 | 3.96E-11 |
| RP4-555L14.4 | 3.08 | 3.80 | 6.88E-12 | 3.97E-11 |
| LINC01980 | 6.24 | 6.32 | 6.89E-12 | 3.98E-11 |
| LINC01194 | 6.17 | 6.06 | 7.09E-12 | 4.09E-11 |
| RP4-547N15.3 | -2.16 | 5.49 | 7.15E-12 | 4.12E-11 |
| RP11-576D8.4 | 2.11 | 3.63 | 7.52E-12 | 4.31E-11 |
| RP11-401O9.4 | 3.40 | 4.53 | 7.76E-12 | 4.44E-11 |
| RP11-103J17.2 | 6.08 | 5.24 | 8.18E-12 | 4.66E-11 |
| RP11-395N3.2 | 2.09 | 5.89 | 8.43E-12 | 4.80E-11 |
| RP5-968D22.3 | 3.43 | 2.88 | 9.32E-12 | 5.29E-11 |
| RP3-410C9.2 | 3.46 | 4.89 | 9.49E-12 | 5.38E-11 |
| LINC02178 | 5.87 | 4.24 | 9.77E-12 | 5.53E-11 |
| RP11-626P14.2 | 4.41 | 4.28 | 9.93E-12 | 5.62E-11 |
| RP11-390F4.6 | 2.47 | 4.73 | 1.00E-11 | 5.66E-11 |
| AC003092.1 | 5.26 | 5.82 | 1.08E-11 | 6.06E-11 |
| AC007405.8 | 2.80 | 3.20 | 1.08E-11 | 6.06E-11 |
| LINC02109 | 4.52 | 4.23 | 1.13E-11 | 6.34E-11 |
| RP11-78F17.1 | 3.56 | 4.13 | 1.19E-11 | 6.68E-11 |
| RP11-445F12.1 | 5.73 | 4.94 | 1.23E-11 | 6.87E-11 |
| RP11-380D23.1 | 4.45 | 3.68 | 1.24E-11 | 6.93E-11 |
| RP11-1038A11.1 | 3.53 | 4.95 | 1.25E-11 | 6.99E-11 |
| LINC02048 | 4.04 | 3.91 | 1.28E-11 | 7.09E-11 |
| RP11-368L12.1 | 3.46 | 4.00 | 1.28E-11 | 7.13E-11 |
| RP11-553A10.1 | 3.01 | 6.67 | 1.33E-11 | 7.37E-11 |
| LINC01269 | 2.15 | 5.78 | 1.40E-11 | 7.74E-11 |
| RP11-348J24.2 | 3.48 | 4.80 | 1.46E-11 | 8.02E-11 |
| RP11-136I14.5 | 3.33 | 3.05 | 1.48E-11 | 8.15E-11 |
| CTD-2147F2.1 | 5.11 | 6.00 | 1.62E-11 | 8.87E-11 |
| ERVK-28 | 3.28 | 3.16 | 1.78E-11 | 9.71E-11 |
| RP11-215P8.4 | 3.67 | 4.18 | 1.86E-11 | 1.02E-10 |
| RP11-148B3.2 | 3.61 | 6.11 | 2.08E-11 | 1.13E-10 |
| RP11-440G9.1 | 3.33 | 3.63 | 2.11E-11 | 1.15E-10 |
| SAMSN1-AS1 | 3.15 | 4.06 | 2.12E-11 | 1.15E-10 |
| LINC00659 | 3.78 | 4.22 | 2.19E-11 | 1.19E-10 |
| RP1-313L4.3 | 2.30 | 4.37 | 2.21E-11 | 1.20E-10 |
| AL122127.25 | 2.08 | 4.22 | 2.26E-11 | 1.22E-10 |
| H19 | 3.39 | 13.48 | 2.37E-11 | 1.28E-10 |
| RP1-40E16.9 | 5.65 | 4.47 | 2.38E-11 | 1.28E-10 |
| RP11-138A9.2 | 2.55 | 6.95 | 2.65E-11 | 1.41E-10 |
| RP11-478J18.2 | 3.14 | 4.11 | 2.82E-11 | 1.50E-10 |
| AC142293.3 | 3.86 | 4.24 | 2.83E-11 | 1.51E-10 |
| LINC01224 | 2.57 | 7.44 | 2.87E-11 | 1.52E-10 |
| RP11-503C24.1 | 4.55 | 3.98 | 2.96E-11 | 1.57E-10 |
| KB-173C10.2 | 2.31 | 4.01 | 3.02E-11 | 1.60E-10 |
| HOXB-AS4 | 4.14 | 4.35 | 3.02E-11 | 1.60E-10 |
| AL162759.1 | 2.85 | 4.46 | 3.05E-11 | 1.61E-10 |
| LINC01811 | -2.04 | 3.17 | 3.12E-11 | 1.64E-10 |
| LINC00898 | 4.56 | 3.85 | 3.20E-11 | 1.69E-10 |
| RP11-120K18.2 | 2.10 | 3.93 | 3.21E-11 | 1.69E-10 |
| RP11-154H12.2 | 2.93 | 3.33 | 3.41E-11 | 1.79E-10 |
| MIR194-2HG | 2.92 | 5.03 | 3.60E-11 | 1.89E-10 |
| CTD-2314B22.1 | 5.00 | 3.44 | 3.67E-11 | 1.92E-10 |
| RP11-462G2.1 | 3.18 | 8.96 | 3.78E-11 | 1.98E-10 |
| RP11-675F6.4 | 3.88 | 3.95 | 3.88E-11 | 2.02E-10 |
| RP11-718O11.1 | 3.03 | 3.05 | 3.91E-11 | 2.04E-10 |
| RP11-167H9.5 | 4.53 | 3.44 | 3.96E-11 | 2.06E-10 |
| RP11-13K12.1 | 3.16 | 6.52 | 4.14E-11 | 2.15E-10 |
| RP11-457M11.5 | 5.02 | 5.53 | 4.17E-11 | 2.17E-10 |
| RP11-479J7.2 | 2.16 | 3.04 | 4.17E-11 | 2.17E-10 |
| RP11-497G19.2 | 4.42 | 6.10 | 4.62E-11 | 2.39E-10 |
| RP11-123K3.9 | 2.18 | 2.98 | 4.78E-11 | 2.46E-10 |
| CASC19 | 4.47 | 4.84 | 4.84E-11 | 2.49E-10 |
| LINC01629 | 3.35 | 4.64 | 4.85E-11 | 2.50E-10 |
| LINC02187 | 4.44 | 4.21 | 4.88E-11 | 2.51E-10 |
| LINC00536 | 3.31 | 2.76 | 4.89E-11 | 2.51E-10 |
| RP1-32I10.10 | 3.02 | 3.38 | 4.90E-11 | 2.52E-10 |
| RP11-734K21.3 | 3.17 | 3.01 | 4.91E-11 | 2.52E-10 |
| LINC01583 | 2.23 | 3.64 | 5.08E-11 | 2.60E-10 |
| RP11-412P11.1 | 5.18 | 3.72 | 5.10E-11 | 2.61E-10 |
| RP11-93H12.4 | 2.65 | 4.00 | 5.21E-11 | 2.67E-10 |
| TBC1D3P1-DHX40P1 | 4.71 | 3.28 | 5.23E-11 | 2.67E-10 |
| AC091801.1 | 4.33 | 3.53 | 5.42E-11 | 2.77E-10 |
| PLUT | 4.99 | 3.77 | 5.46E-11 | 2.78E-10 |
| RP11-231N3.1 | 2.88 | 3.34 | 5.47E-11 | 2.79E-10 |
| RP11-454P21.1 | 3.66 | 3.70 | 5.88E-11 | 2.98E-10 |
| LINC00589 | 2.22 | 5.53 | 6.01E-11 | 3.05E-10 |
| AC114730.3 | 2.18 | 5.85 | 6.08E-11 | 3.08E-10 |
| RP11-642C5.1 | 3.26 | 3.63 | 6.10E-11 | 3.09E-10 |
| RP11-141M3.6 | 2.41 | 7.08 | 6.14E-11 | 3.10E-10 |
| LINC01344 | 2.41 | 3.87 | 6.24E-11 | 3.15E-10 |
| LINC01299 | 4.94 | 4.01 | 6.34E-11 | 3.20E-10 |
| RP11-299H22.6 | 2.69 | 4.72 | 6.92E-11 | 3.48E-10 |
| LINC01297 | 4.17 | 3.43 | 7.67E-11 | 3.85E-10 |
| RP11-897M7.1 | 4.12 | 3.58 | 7.67E-11 | 3.85E-10 |
| RP11-565A3.2 | 5.32 | 3.97 | 7.71E-11 | 3.86E-10 |
| LINC00184 | 2.01 | 4.79 | 7.85E-11 | 3.92E-10 |
| RP3-522D1.1 | 2.70 | 4.37 | 7.99E-11 | 3.99E-10 |
| RP3-495K2.2 | 2.38 | 2.96 | 8.31E-11 | 4.14E-10 |
| RP1-207H1.3 | 2.66 | 3.11 | 8.42E-11 | 4.19E-10 |
| AGBL1-AS1 | -2.07 | 2.78 | 8.64E-11 | 4.30E-10 |
| RP11-486M23.1 | 2.59 | 4.20 | 9.02E-11 | 4.48E-10 |
| SPATA3-AS1 | 2.32 | 3.77 | 9.48E-11 | 4.70E-10 |
| RP11-6N13.1 | 4.42 | 3.20 | 9.59E-11 | 4.76E-10 |
| LINC01571 | -2.07 | 4.52 | 9.75E-11 | 4.82E-10 |
| RP11-6J21.2 | 2.79 | 5.56 | 1.03E-10 | 5.09E-10 |
| ST7-OT4 | 2.06 | 5.03 | 1.04E-10 | 5.10E-10 |
| RP11-881M11.4 | 2.07 | 3.26 | 1.13E-10 | 5.53E-10 |
| LINC00524 | 3.40 | 4.60 | 1.18E-10 | 5.78E-10 |
| DEPDC1-AS1 | 2.19 | 3.10 | 1.19E-10 | 5.81E-10 |
| RP11-675F6.3 | 3.57 | 4.17 | 1.26E-10 | 6.18E-10 |
| LINC01206 | 3.68 | 4.63 | 1.39E-10 | 6.79E-10 |
| CHL1-AS1 | 2.39 | 3.73 | 1.45E-10 | 7.07E-10 |
| RP11-502M1.2 | 2.91 | 3.19 | 1.49E-10 | 7.21E-10 |
| RP11-538I12.3 | 3.15 | 3.41 | 1.50E-10 | 7.26E-10 |
| KIF25-AS1 | 3.35 | 7.27 | 1.55E-10 | 7.47E-10 |
| LINC01510 | 3.70 | 4.14 | 1.57E-10 | 7.56E-10 |
| RP11-241G9.3 | 2.39 | 3.46 | 1.57E-10 | 7.59E-10 |
| CTC-340I23.2 | 2.89 | 2.81 | 1.59E-10 | 7.66E-10 |
| CDKN2A-AS1 | 2.78 | 3.26 | 1.66E-10 | 7.97E-10 |
| ARNTL2-AS1 | 2.74 | 3.18 | 1.67E-10 | 8.02E-10 |
| RP11-13E5.2 | 3.72 | 2.74 | 1.69E-10 | 8.10E-10 |
| HNF1A-AS1 | 2.64 | 7.74 | 1.70E-10 | 8.18E-10 |
| C5orf66-AS1 | 4.03 | 4.14 | 1.76E-10 | 8.42E-10 |
| CTD-2066L21.2 | 4.50 | 3.40 | 1.78E-10 | 8.51E-10 |
| RP11-390N6.1 | 2.96 | 3.60 | 1.79E-10 | 8.55E-10 |
| LA16c-60D12.2 | 5.14 | 3.77 | 1.81E-10 | 8.66E-10 |
| SOX21-AS1 | 2.29 | 7.96 | 1.91E-10 | 9.09E-10 |
| LINC00958 | 2.54 | 7.74 | 1.93E-10 | 9.17E-10 |
| RP11-488P3.1 | 2.49 | 7.30 | 1.95E-10 | 9.26E-10 |
| RP11-372H2.1 | 3.50 | 3.69 | 1.99E-10 | 9.44E-10 |
| LINC01449 | 2.09 | 2.90 | 2.04E-10 | 9.64E-10 |
| RP11-677M24.1 | 3.94 | 3.08 | 2.08E-10 | 9.82E-10 |
| RP3-438O4.4 | 3.97 | 3.97 | 2.10E-10 | 9.90E-10 |
| AC073284.4 | 2.96 | 2.82 | 2.11E-10 | 9.97E-10 |
| RP11-21L23.4 | 3.32 | 5.20 | 2.16E-10 | 1.02E-09 |
| RP11-274M17.3 | 3.34 | 2.79 | 2.17E-10 | 1.02E-09 |
| MEG3 | 2.04 | 11.80 | 2.17E-10 | 1.02E-09 |
| RP11-180C1.1 | 5.86 | 4.27 | 2.19E-10 | 1.03E-09 |
| LINC01356 | 2.34 | 4.88 | 2.35E-10 | 1.10E-09 |
| LINC00704 | 3.15 | 5.81 | 2.38E-10 | 1.12E-09 |
| RP11-434D9.2 | 2.65 | 2.88 | 2.56E-10 | 1.19E-09 |
| RP11-96B2.1 | 3.64 | 3.60 | 2.58E-10 | 1.20E-09 |
| LINC00648 | 3.98 | 7.09 | 2.77E-10 | 1.29E-09 |
| RP11-1103G16.1 | 5.19 | 4.31 | 2.85E-10 | 1.32E-09 |
| RP11-115J23.1 | 3.22 | 3.67 | 2.90E-10 | 1.35E-09 |
| CTD-2377D24.4 | 5.06 | 4.09 | 3.05E-10 | 1.41E-09 |
| RP4-799P18.2 | 2.13 | 3.94 | 3.08E-10 | 1.42E-09 |
| LINC01429 | 2.82 | 3.10 | 3.10E-10 | 1.43E-09 |
| RP11-548L20.1 | 5.85 | 4.07 | 3.16E-10 | 1.46E-09 |
| ERVMER61-1 | 5.71 | 4.17 | 3.23E-10 | 1.49E-09 |
| RP11-320M16.2 | 3.00 | 3.34 | 3.24E-10 | 1.49E-09 |
| CTD-3080P12.3 | -2.09 | 4.41 | 3.28E-10 | 1.51E-09 |
| CTB-49A3.4 | 2.87 | 3.99 | 3.43E-10 | 1.57E-09 |
| RP11-314N14.1 | 5.75 | 3.97 | 3.54E-10 | 1.62E-09 |
| LINC01967 | 4.16 | 3.51 | 3.58E-10 | 1.64E-09 |
| AC006946.17 | 2.05 | 3.58 | 3.68E-10 | 1.68E-09 |
| SAMMSON | 2.57 | 3.41 | 3.75E-10 | 1.71E-09 |
| AC016723.4 | 4.42 | 3.83 | 3.94E-10 | 1.79E-09 |
| AC113617.1 | 3.98 | 3.07 | 4.07E-10 | 1.85E-09 |
| RP11-476K15.1 | 4.43 | 5.10 | 4.11E-10 | 1.87E-09 |
| CTD-2555A7.2 | 2.91 | 3.47 | 4.31E-10 | 1.95E-09 |
| ABCA9-AS1 | 3.18 | 4.67 | 4.51E-10 | 2.04E-09 |
| RP13-714J12.1 | 2.03 | 5.00 | 4.57E-10 | 2.07E-09 |
| AC092675.3 | 2.51 | 4.80 | 4.61E-10 | 2.08E-09 |
| RP11-96H17.3 | 3.29 | 2.99 | 4.63E-10 | 2.09E-09 |
| CTD-2340E1.2 | 3.73 | 3.18 | 4.64E-10 | 2.09E-09 |
| RP11-471M2.3 | 5.02 | 3.76 | 4.73E-10 | 2.13E-09 |
| LINC00355 | 4.38 | 5.70 | 4.80E-10 | 2.16E-09 |
| RP11-546K22.1 | 2.18 | 3.89 | 4.98E-10 | 2.24E-09 |
| RP11-133K1.11 | 2.19 | 2.79 | 4.98E-10 | 2.24E-09 |
| LINC00592 | 2.00 | 4.87 | 5.09E-10 | 2.28E-09 |
| LINC00601 | 3.70 | 3.69 | 5.29E-10 | 2.37E-09 |
| RPPH1 | 6.10 | 8.05 | 5.41E-10 | 2.42E-09 |
| LINC01524 | 3.29 | 3.52 | 5.50E-10 | 2.46E-09 |
| RP11-844P9.5 | 2.13 | 6.51 | 5.51E-10 | 2.46E-09 |
| LINC02208 | 2.81 | 3.34 | 5.77E-10 | 2.57E-09 |
| CTD-2265O21.3 | 3.13 | 2.84 | 5.83E-10 | 2.60E-09 |
| RP11-143E21.3 | 6.27 | 6.31 | 5.86E-10 | 2.61E-09 |
| RP11-525A16.4 | 2.09 | 4.02 | 5.94E-10 | 2.64E-09 |
| DPYD-AS1 | 2.90 | 6.85 | 6.14E-10 | 2.73E-09 |
| RP11-360L9.7 | 2.19 | 2.68 | 6.38E-10 | 2.83E-09 |
| LINC01116 | 2.03 | 7.96 | 6.53E-10 | 2.90E-09 |
| AF003626.1 | 6.52 | 4.96 | 6.56E-10 | 2.91E-09 |
| RP11-330A16.1 | 2.41 | 3.62 | 6.58E-10 | 2.92E-09 |
| RP11-264E20.1 | 2.04 | 2.79 | 6.69E-10 | 2.96E-09 |
| RP11-663N22.1 | 2.40 | 5.02 | 6.81E-10 | 3.01E-09 |
| ZFHX4-AS1 | 4.00 | 4.21 | 7.37E-10 | 3.25E-09 |
| RP11-834C11.6 | 2.62 | 3.39 | 7.50E-10 | 3.30E-09 |
| CTA-397H3.3 | 2.03 | 2.83 | 7.63E-10 | 3.35E-09 |
| RP11-308B16.2 | 5.40 | 4.46 | 7.81E-10 | 3.43E-09 |
| RP11-1000B6.2 | 2.30 | 2.58 | 7.86E-10 | 3.45E-09 |
| CTB-107G13.1 | 2.56 | 5.28 | 7.88E-10 | 3.46E-09 |
| RP11-180M15.3 | 3.31 | 3.26 | 8.06E-10 | 3.53E-09 |
| RP4-784A16.3 | 2.57 | 2.59 | 8.06E-10 | 3.53E-09 |
| FER1L6-AS2 | 4.26 | 3.33 | 8.20E-10 | 3.59E-09 |
| RP11-173C1.1 | 2.45 | 6.40 | 8.36E-10 | 3.65E-09 |
| RP11-323H21.3 | 6.90 | 5.85 | 8.57E-10 | 3.74E-09 |
| LINC01419 | 7.25 | 7.74 | 8.63E-10 | 3.76E-09 |
| CTB-49A3.2 | 2.08 | 4.10 | 8.79E-10 | 3.83E-09 |
| LINC00221 | 5.21 | 6.11 | 8.98E-10 | 3.91E-09 |
| RP11-680F20.10 | 2.93 | 3.60 | 9.08E-10 | 3.95E-09 |
| RP11-474D1.4 | 5.55 | 4.02 | 9.18E-10 | 3.98E-09 |
| CTC-343N3.1 | 2.04 | 5.16 | 9.45E-10 | 4.09E-09 |
| LINC02152 | 4.30 | 3.45 | 9.71E-10 | 4.20E-09 |
| ATP11AUN | 3.81 | 5.97 | 9.91E-10 | 4.28E-09 |
| KCNQ5-IT1 | 3.45 | 3.59 | 9.92E-10 | 4.29E-09 |
| LINC01776 | 3.71 | 3.35 | 1.05E-09 | 4.54E-09 |
| RP11-542G1.1 | 4.06 | 3.62 | 1.07E-09 | 4.63E-09 |
| RP11-436D23.1 | 3.96 | 3.10 | 1.09E-09 | 4.69E-09 |
| RP11-401O9.3 | 3.09 | 4.10 | 1.15E-09 | 4.96E-09 |
| LINC00668 | 3.99 | 6.48 | 1.18E-09 | 5.06E-09 |
| RP11-109E24.1 | 2.12 | 3.27 | 1.19E-09 | 5.11E-09 |
| LINC02192 | 3.47 | 3.21 | 1.23E-09 | 5.28E-09 |
| ZBTB20-AS1 | 2.03 | 4.48 | 1.24E-09 | 5.32E-09 |
| PRKCA-AS1 | 3.74 | 4.31 | 1.29E-09 | 5.50E-09 |
| KB-173C10.1 | 2.08 | 3.01 | 1.32E-09 | 5.65E-09 |
| RP11-874J12.4 | 2.89 | 5.04 | 1.34E-09 | 5.72E-09 |
| MIR181A1HG | 2.39 | 5.06 | 1.39E-09 | 5.92E-09 |
| RP11-1038A11.3 | 3.12 | 6.60 | 1.44E-09 | 6.10E-09 |
| CTD-2311M21.3 | 2.14 | 4.46 | 1.51E-09 | 6.41E-09 |
| LINC00319 | 2.62 | 3.32 | 1.52E-09 | 6.45E-09 |
| LINC01543 | 3.32 | 2.95 | 1.56E-09 | 6.60E-09 |
| RP4-669H2.1 | 2.09 | 4.89 | 1.59E-09 | 6.72E-09 |
| RP11-431M3.1 | 3.60 | 2.69 | 1.64E-09 | 6.92E-09 |
| RP11-567N4.3 | 4.60 | 3.77 | 1.69E-09 | 7.10E-09 |
| RP11-184M15.2 | 2.90 | 3.69 | 1.72E-09 | 7.22E-09 |
| CTD-2013M15.1 | 3.62 | 3.66 | 1.78E-09 | 7.46E-09 |
| RP11-337A23.6 | 4.67 | 3.42 | 1.91E-09 | 7.98E-09 |
| TBL1XR1-AS1 | 2.75 | 3.78 | 1.91E-09 | 7.98E-09 |
| HAR1B | 2.27 | 5.32 | 1.94E-09 | 8.08E-09 |
| RP11-284G10.1 | 5.11 | 3.65 | 1.94E-09 | 8.09E-09 |
| RP11-78L16.1 | 5.22 | 3.76 | 2.00E-09 | 8.32E-09 |
| RP11-587P21.2 | 5.46 | 4.93 | 2.08E-09 | 8.61E-09 |
| RP11-135A1.3 | 3.47 | 2.99 | 2.09E-09 | 8.64E-09 |
| RP3-326I13.1 | 4.66 | 3.54 | 2.17E-09 | 8.95E-09 |
| CTD-2616J11.16 | 2.05 | 3.07 | 2.17E-09 | 8.96E-09 |
| RP11-366F6.2 | 6.38 | 4.69 | 2.19E-09 | 9.01E-09 |
| LINC01605 | 2.01 | 5.94 | 2.20E-09 | 9.07E-09 |
| RP11-646E18.4 | 3.52 | 3.08 | 2.23E-09 | 9.18E-09 |
| RP11-486M23.2 | 2.90 | 3.13 | 2.26E-09 | 9.30E-09 |
| RP5-1011O1.3 | 2.82 | 3.05 | 2.27E-09 | 9.34E-09 |
| CTC-261N6.1 | 4.09 | 3.05 | 2.29E-09 | 9.40E-09 |
| RP11-366L20.3 | 3.36 | 3.03 | 2.31E-09 | 9.47E-09 |
| RP11-962G15.1 | 5.53 | 3.97 | 2.38E-09 | 9.72E-09 |
| RP11-608O21.1 | 4.11 | 3.56 | 2.39E-09 | 9.79E-09 |
| RP11-510M2.5 | 2.51 | 4.58 | 2.44E-09 | 9.95E-09 |
| TTTY16 | -2.17 | 2.75 | 2.44E-09 | 9.96E-09 |
| LVCAT5 | 5.70 | 4.74 | 2.45E-09 | 9.98E-09 |
| CTD-2130O13.1 | 3.47 | 4.27 | 2.59E-09 | 1.05E-08 |
| RP11-669N7.2 | 5.70 | 4.57 | 2.59E-09 | 1.05E-08 |
| RP11-146E13.4 | 2.58 | 3.21 | 2.59E-09 | 1.05E-08 |
| RP4-797C5.2 | 2.95 | 3.44 | 2.61E-09 | 1.06E-08 |
| RP11-507B12.2 | 3.42 | 3.51 | 2.68E-09 | 1.09E-08 |
| RP11-490G2.2 | 3.51 | 2.87 | 2.76E-09 | 1.12E-08 |
| AF121898.3 | 4.55 | 3.71 | 2.81E-09 | 1.14E-08 |
| RP11-159D12.11 | 2.04 | 2.98 | 2.84E-09 | 1.15E-08 |
| CTD-2297D10.2 | 2.06 | 3.27 | 2.86E-09 | 1.16E-08 |
| LINC01667 | 5.83 | 6.35 | 2.94E-09 | 1.19E-08 |
| AE000658.31 | 2.11 | 2.88 | 2.97E-09 | 1.20E-08 |
| AC015933.2 | 3.86 | 2.91 | 3.03E-09 | 1.22E-08 |
| AL773572.7 | 4.39 | 5.50 | 3.08E-09 | 1.24E-08 |
| RP5-1121A15.3 | 3.86 | 3.16 | 3.11E-09 | 1.25E-08 |
| RP1-272L16.1 | 3.55 | 3.02 | 3.13E-09 | 1.26E-08 |
| DSG1-AS1 | 4.73 | 3.93 | 3.15E-09 | 1.27E-08 |
| RP11-407A16.3 | 3.74 | 3.50 | 3.20E-09 | 1.28E-08 |
| TUSC8 | 3.81 | 6.03 | 3.23E-09 | 1.30E-08 |
| RP11-221N13.3 | 2.87 | 4.66 | 3.24E-09 | 1.30E-08 |
| LINC01873 | 2.56 | 2.89 | 3.27E-09 | 1.31E-08 |
| RP11-16D22.2 | 2.49 | 2.98 | 3.28E-09 | 1.31E-08 |
| LINC01913 | 2.78 | 5.30 | 3.30E-09 | 1.32E-08 |
| CPS1-IT1 | 4.67 | 3.24 | 3.33E-09 | 1.33E-08 |
| RP11-1070N10.5 | 4.13 | 4.30 | 3.38E-09 | 1.35E-08 |
| RP11-419J16.1 | 2.20 | 3.32 | 3.51E-09 | 1.40E-08 |
| RP11-53B5.1 | 3.49 | 5.50 | 3.54E-09 | 1.41E-08 |
| RP11-752D24.2 | 2.06 | 4.21 | 3.64E-09 | 1.45E-08 |
| XXyac-YM21GA2.7 | -2.01 | 5.09 | 3.69E-09 | 1.47E-08 |
| RP11-332J15.2 | 5.32 | 4.33 | 3.70E-09 | 1.47E-08 |
| CTB-186G2.1 | 2.25 | 3.81 | 3.70E-09 | 1.47E-08 |
| LINC00645 | 4.56 | 3.55 | 3.78E-09 | 1.50E-08 |
| AC003986.6 | 2.35 | 2.92 | 3.82E-09 | 1.51E-08 |
| RP11-138A9.1 | 2.72 | 5.78 | 3.83E-09 | 1.52E-08 |
| CTA-390C10.9 | 2.89 | 2.73 | 3.97E-09 | 1.57E-08 |
| RP11-18A15.1 | 3.61 | 2.89 | 3.98E-09 | 1.58E-08 |
| RP4-631H13.2 | 2.58 | 2.60 | 4.19E-09 | 1.65E-08 |
| LINC00393 | 4.81 | 4.11 | 4.39E-09 | 1.73E-08 |
| RP11-280K24.4 | 2.78 | 3.53 | 4.48E-09 | 1.76E-08 |
| RP11-344P13.4 | 2.81 | 2.57 | 4.77E-09 | 1.86E-08 |
| LINC01202 | 4.88 | 3.38 | 4.98E-09 | 1.94E-08 |
| RP11-145G20.1 | 5.52 | 4.32 | 5.00E-09 | 1.95E-08 |
| AC068858.1 | 2.07 | 2.71 | 5.25E-09 | 2.04E-08 |
| RP11-503C24.4 | 3.18 | 4.07 | 5.30E-09 | 2.06E-08 |
| LINC01257 | 4.68 | 5.30 | 5.43E-09 | 2.11E-08 |
| RP11-230G5.2 | 3.93 | 7.06 | 5.72E-09 | 2.22E-08 |
| LINC00543 | 2.03 | 6.33 | 5.82E-09 | 2.25E-08 |
| CTC-465D4.1 | 2.37 | 3.86 | 5.92E-09 | 2.29E-08 |
| LINC01249 | 4.80 | 3.99 | 6.13E-09 | 2.37E-08 |
| AC099342.1 | 2.74 | 2.74 | 6.23E-09 | 2.40E-08 |
| CTD-2021H9.1 | 3.49 | 3.00 | 6.35E-09 | 2.45E-08 |
| RP11-1O10.1 | 3.17 | 3.51 | 6.38E-09 | 2.46E-08 |
| RP3-333B15.5 | 3.01 | 3.03 | 6.48E-09 | 2.50E-08 |
| AC004158.3 | 3.01 | 3.10 | 6.70E-09 | 2.58E-08 |
| LINC02188 | 3.01 | 7.06 | 6.72E-09 | 2.59E-08 |
| LINC00330 | 2.14 | 3.75 | 6.76E-09 | 2.60E-08 |
| AC092198.1 | 2.51 | 5.27 | 7.03E-09 | 2.69E-08 |
| AC006380.3 | 4.94 | 3.88 | 7.21E-09 | 2.76E-08 |
| RP11-416N4.1 | 2.88 | 2.86 | 7.24E-09 | 2.77E-08 |
| GRM5-AS1 | 2.26 | 3.44 | 7.31E-09 | 2.79E-08 |
| LINC01446 | 4.46 | 6.11 | 7.35E-09 | 2.81E-08 |
| AC005808.3 | 2.85 | 2.74 | 7.60E-09 | 2.89E-08 |
| LINC01098 | 3.23 | 3.50 | 7.70E-09 | 2.93E-08 |
| LINC00383 | 4.76 | 3.30 | 7.91E-09 | 3.00E-08 |
| RP11-377G16.2 | 2.35 | 3.27 | 7.97E-09 | 3.02E-08 |
| RP11-114G22.1 | 3.31 | 3.94 | 8.07E-09 | 3.06E-08 |
| LINC01456 | 4.60 | 3.66 | 8.17E-09 | 3.09E-08 |
| LINC01345 | 5.22 | 4.05 | 8.27E-09 | 3.12E-08 |
| VCAN-AS1 | 2.56 | 4.03 | 8.99E-09 | 3.38E-08 |
| RP11-290L1.5 | 2.96 | 2.63 | 9.09E-09 | 3.42E-08 |
| LINC01924 | 4.54 | 3.71 | 9.57E-09 | 3.59E-08 |
| RP11-157E21.1 | 3.01 | 5.78 | 1.02E-08 | 3.81E-08 |
| AC006145.4 | 4.22 | 3.67 | 1.04E-08 | 3.90E-08 |
| CTD-2385L22.1 | 2.99 | 3.89 | 1.07E-08 | 3.97E-08 |
| LINC00698 | 2.24 | 2.75 | 1.07E-08 | 4.00E-08 |
| RP11-65J3.2 | 2.41 | 2.79 | 1.09E-08 | 4.05E-08 |
| AC114812.8 | 3.23 | 2.98 | 1.12E-08 | 4.18E-08 |
| LINC01659 | 2.05 | 5.52 | 1.13E-08 | 4.19E-08 |
| RP13-192B19.2 | 2.89 | 2.99 | 1.16E-08 | 4.32E-08 |
| RP11-13K12.5 | 2.62 | 5.98 | 1.17E-08 | 4.33E-08 |
| CTD-2540B15.6 | 2.64 | 2.91 | 1.20E-08 | 4.45E-08 |
| RP11-1070N10.7 | 4.16 | 3.82 | 1.24E-08 | 4.57E-08 |
| RP11-542K23.9 | 2.14 | 3.31 | 1.26E-08 | 4.65E-08 |
| RP11-221N13.4 | 4.55 | 3.28 | 1.28E-08 | 4.72E-08 |
| RP11-543G18.1 | 2.31 | 3.02 | 1.30E-08 | 4.79E-08 |
| RP11-586D19.2 | 2.47 | 2.87 | 1.32E-08 | 4.87E-08 |
| SLC8A1-AS1 | 2.01 | 5.63 | 1.36E-08 | 4.98E-08 |
| RP11-114H21.2 | 4.12 | 3.07 | 1.36E-08 | 5.00E-08 |
| RP11-705O24.1 | 2.42 | 3.12 | 1.39E-08 | 5.11E-08 |
| RP11-699A5.2 | 2.80 | 2.64 | 1.59E-08 | 5.81E-08 |
| AC005307.1 | 3.95 | 4.06 | 1.62E-08 | 5.91E-08 |
| LINC00392 | 5.79 | 3.99 | 1.63E-08 | 5.95E-08 |
| RP11-398B16.2 | 6.77 | 4.81 | 1.70E-08 | 6.19E-08 |
| LINC01701 | 2.58 | 2.84 | 1.71E-08 | 6.20E-08 |
| RP11-469H8.6 | 2.16 | 8.18 | 1.71E-08 | 6.22E-08 |
| CTD-2354A18.1 | 6.01 | 5.52 | 1.72E-08 | 6.24E-08 |
| RP11-485F13.1 | 5.21 | 3.94 | 1.75E-08 | 6.34E-08 |
| LA16c-352F7.1 | 2.17 | 3.91 | 1.82E-08 | 6.58E-08 |
| RP11-734I18.1 | 4.61 | 5.28 | 1.85E-08 | 6.68E-08 |
| RP11-1038A11.2 | 3.01 | 2.88 | 1.89E-08 | 6.84E-08 |
| LINC00470 | 3.10 | 6.06 | 1.90E-08 | 6.87E-08 |
| RP11-326N17.2 | 2.29 | 2.90 | 1.92E-08 | 6.94E-08 |
| RP11-94P11.4 | 3.49 | 2.74 | 1.93E-08 | 6.94E-08 |
| FAM230C | 5.34 | 4.63 | 1.99E-08 | 7.15E-08 |
| RP11-79E3.3 | 5.26 | 4.07 | 2.00E-08 | 7.18E-08 |
| AC007193.6 | 2.70 | 3.06 | 2.01E-08 | 7.23E-08 |
| AC027119.1 | 4.10 | 3.31 | 2.04E-08 | 7.32E-08 |
| LINC01399 | 2.82 | 2.77 | 2.09E-08 | 7.48E-08 |
| RP4-781K5.4 | 2.77 | 5.95 | 2.11E-08 | 7.54E-08 |
| RP11-329E24.6 | 3.68 | 2.93 | 2.14E-08 | 7.64E-08 |
| MIR5689HG | 3.08 | 3.04 | 2.15E-08 | 7.68E-08 |
| PROX1-AS1 | 2.06 | 5.28 | 2.16E-08 | 7.73E-08 |
| RP11-408B11.2 | 4.23 | 4.62 | 2.22E-08 | 7.94E-08 |
| AC009264.1 | 3.57 | 4.52 | 2.25E-08 | 8.02E-08 |
| RP11-332K15.1 | 2.32 | 3.07 | 2.37E-08 | 8.46E-08 |
| SC22CB-1D7.1 | 3.32 | 3.54 | 2.42E-08 | 8.60E-08 |
| RP11-706C16.8 | 3.27 | 2.81 | 2.47E-08 | 8.77E-08 |
| RP1-63G5.7 | 2.20 | 3.27 | 2.47E-08 | 8.79E-08 |
| RP11-21A7A.4 | 2.20 | 2.71 | 2.49E-08 | 8.84E-08 |
| LINC02037 | 3.97 | 4.85 | 2.60E-08 | 9.19E-08 |
| CTD-2314G24.2 | 2.66 | 5.61 | 2.62E-08 | 9.26E-08 |
| RP11-254F7.1 | 3.98 | 3.16 | 2.64E-08 | 9.33E-08 |
| RP11-502H18.2 | 2.58 | 2.62 | 2.67E-08 | 9.41E-08 |
| LINC00602 | 3.79 | 3.67 | 2.87E-08 | 1.01E-07 |
| RP11-279N8.1 | 2.56 | 2.88 | 2.89E-08 | 1.02E-07 |
| RP11-685G9.2 | 2.34 | 2.96 | 3.00E-08 | 1.05E-07 |
| RP11-476M19.2 | 2.83 | 3.57 | 3.02E-08 | 1.06E-07 |
| RP11-234O6.2 | 4.94 | 3.40 | 3.04E-08 | 1.07E-07 |
| RP11-32K4.1 | 4.16 | 3.39 | 3.16E-08 | 1.10E-07 |
| RP5-944M2.3 | 2.79 | 3.66 | 3.26E-08 | 1.14E-07 |
| RP11-119K6.6 | 2.34 | 2.77 | 3.26E-08 | 1.14E-07 |
| RP11-80I3.1 | 2.16 | 2.80 | 3.51E-08 | 1.22E-07 |
| RP11-408N14.1 | 2.93 | 2.88 | 3.52E-08 | 1.22E-07 |
| RP11-315E17.1 | 4.03 | 3.90 | 3.58E-08 | 1.24E-07 |
| LINC01096 | 2.60 | 3.52 | 3.63E-08 | 1.26E-07 |
| RP11-123O10.4 | 2.03 | 3.13 | 3.76E-08 | 1.30E-07 |
| LINC01956 | 3.77 | 3.51 | 3.76E-08 | 1.30E-07 |
| SMILR | 2.91 | 3.91 | 3.77E-08 | 1.30E-07 |
| RP4-536B24.2 | 2.17 | 2.60 | 3.78E-08 | 1.31E-07 |
| RP11-595B24.2 | 2.94 | 3.74 | 3.81E-08 | 1.31E-07 |
| RP11-317N12.1 | 4.85 | 4.19 | 3.88E-08 | 1.34E-07 |
| GACAT3 | 4.89 | 4.02 | 3.91E-08 | 1.35E-07 |
| RP11-390B4.3 | 2.23 | 4.00 | 3.94E-08 | 1.36E-07 |
| MKRN3-AS1 | 2.68 | 2.98 | 4.04E-08 | 1.39E-07 |
| RMRP | 5.99 | 6.38 | 4.27E-08 | 1.46E-07 |
| RP11-272B17.2 | 4.54 | 3.15 | 4.27E-08 | 1.46E-07 |
| MEG8 | 2.25 | 4.24 | 4.75E-08 | 1.62E-07 |
| RP11-445P19.3 | 3.26 | 2.69 | 4.91E-08 | 1.67E-07 |
| LSAMP-AS1 | 3.07 | 3.67 | 4.93E-08 | 1.68E-07 |
| RP13-487P22.1 | 2.15 | 3.66 | 5.00E-08 | 1.70E-07 |
| RP11-260A9.6 | 4.95 | 3.69 | 5.26E-08 | 1.79E-07 |
| RP11-416A14.1 | 2.06 | 3.52 | 5.44E-08 | 1.85E-07 |
| LINC00871 | 3.70 | 4.15 | 5.80E-08 | 1.97E-07 |
| AC068580.7 | 2.62 | 3.00 | 5.82E-08 | 1.97E-07 |
| RP11-897M7.4 | 3.58 | 3.23 | 6.02E-08 | 2.04E-07 |
| LINC01731 | 2.35 | 2.92 | 6.02E-08 | 2.04E-07 |
| RP11-364P22.1 | 2.48 | 3.10 | 6.05E-08 | 2.05E-07 |
| RP11-108M12.3 | 2.14 | 4.80 | 6.34E-08 | 2.14E-07 |
| RP4-781K5.5 | 3.71 | 2.93 | 6.39E-08 | 2.15E-07 |
| RP11-865I6.2 | 2.56 | 3.94 | 6.56E-08 | 2.21E-07 |
| ST8SIA6-AS1 | 3.26 | 7.10 | 6.87E-08 | 2.30E-07 |
| RP11-30K9.5 | 2.14 | 5.61 | 6.94E-08 | 2.33E-07 |
| RP11-809H16.4 | 3.36 | 2.86 | 7.04E-08 | 2.36E-07 |
| LINC00473 | 3.66 | 9.16 | 7.05E-08 | 2.36E-07 |
| AC114765.1 | 2.46 | 2.67 | 7.31E-08 | 2.44E-07 |
| RP11-3J1.1 | 3.78 | 3.58 | 7.36E-08 | 2.46E-07 |
| LINC01740 | 3.46 | 4.98 | 7.42E-08 | 2.48E-07 |
| RP11-398J5.1 | 4.12 | 2.92 | 7.49E-08 | 2.50E-07 |
| LINC01981 | 3.21 | 2.54 | 7.64E-08 | 2.55E-07 |
| RP11-449J1.1 | 3.22 | 3.33 | 7.85E-08 | 2.61E-07 |
| RP11-367F23.2 | 2.53 | 2.77 | 7.94E-08 | 2.64E-07 |
| RP11-689C9.1 | 2.94 | 3.74 | 7.96E-08 | 2.65E-07 |
| AC007389.3 | 2.22 | 2.71 | 7.97E-08 | 2.65E-07 |
| RP11-12M5.3 | 2.52 | 3.68 | 8.05E-08 | 2.67E-07 |
| KB-1184D12.1 | 2.26 | 2.59 | 8.10E-08 | 2.69E-07 |
| RP11-104E19.1 | 2.53 | 3.68 | 8.12E-08 | 2.69E-07 |
| RP11-66B24.1 | 2.62 | 3.23 | 8.21E-08 | 2.72E-07 |
| AC137723.5 | 2.07 | 2.62 | 8.89E-08 | 2.94E-07 |
| RP11-336A10.2 | 2.12 | 4.16 | 9.27E-08 | 3.07E-07 |
| CTC-241F20.4 | 2.34 | 3.41 | 9.31E-08 | 3.08E-07 |
| CTD-2587M23.1 | 2.94 | 3.29 | 9.33E-08 | 3.08E-07 |
| RP11-319E16.2 | 3.06 | 2.67 | 9.33E-08 | 3.08E-07 |
| RP11-54H7.4 | 3.04 | 8.78 | 9.58E-08 | 3.16E-07 |
| RP4-655C5.4 | 2.85 | 4.20 | 9.58E-08 | 3.16E-07 |
| LINC01681 | 3.63 | 3.09 | 9.69E-08 | 3.19E-07 |
| LINC01910 | 2.58 | 3.22 | 9.78E-08 | 3.22E-07 |
| CTD-2021J15.1 | 2.00 | 3.36 | 1.01E-07 | 3.30E-07 |
| LINC01037 | 3.28 | 2.98 | 1.01E-07 | 3.32E-07 |
| LINC02105 | 3.41 | 2.88 | 1.02E-07 | 3.34E-07 |
| RP11-52L5.6 | 2.98 | 2.82 | 1.02E-07 | 3.36E-07 |
| OVAAL | 3.09 | 3.81 | 1.03E-07 | 3.37E-07 |
| LINC01812 | 2.60 | 2.73 | 1.05E-07 | 3.42E-07 |
| CASC20 | 3.53 | 4.42 | 1.05E-07 | 3.42E-07 |
| RP11-346D14.1 | 2.26 | 2.65 | 1.06E-07 | 3.46E-07 |
| AC037445.1 | 2.28 | 3.52 | 1.08E-07 | 3.53E-07 |
| LINC02167 | 3.11 | 2.61 | 1.09E-07 | 3.57E-07 |
| LINC01204 | 2.52 | 3.89 | 1.13E-07 | 3.66E-07 |
| RP11-78A19.4 | 3.10 | 3.30 | 1.13E-07 | 3.68E-07 |
| LINC02111 | 2.83 | 2.90 | 1.15E-07 | 3.73E-07 |
| RP11-217E22.5 | 3.59 | 2.96 | 1.16E-07 | 3.76E-07 |
| LINC01210 | 4.56 | 3.68 | 1.19E-07 | 3.86E-07 |
| ARHGAP26-AS1 | 2.06 | 3.79 | 1.23E-07 | 3.99E-07 |
| LINC00308 | 3.22 | 2.55 | 1.24E-07 | 4.00E-07 |
| RP4-529N6.1 | 2.76 | 3.24 | 1.25E-07 | 4.03E-07 |
| HOTTIP | 3.57 | 3.66 | 1.27E-07 | 4.11E-07 |
| RP11-774I5.1 | 4.10 | 2.97 | 1.30E-07 | 4.19E-07 |
| AC025016.1 | 4.53 | 3.59 | 1.36E-07 | 4.37E-07 |
| RP11-11N5.1 | 2.85 | 3.17 | 1.37E-07 | 4.38E-07 |
| RP11-14C10.3 | 2.28 | 3.36 | 1.41E-07 | 4.51E-07 |
| LINC01608 | 5.23 | 4.09 | 1.44E-07 | 4.62E-07 |
| AF003625.3 | 4.45 | 3.65 | 1.46E-07 | 4.67E-07 |
| LINC01079 | 3.32 | 4.01 | 1.48E-07 | 4.74E-07 |
| RP11-269F21.3 | 4.51 | 4.95 | 1.51E-07 | 4.82E-07 |
| CTC-513N18.6 | 2.17 | 3.48 | 1.54E-07 | 4.92E-07 |
| LINC02196 | 3.65 | 2.96 | 1.55E-07 | 4.94E-07 |
| LINC00706 | 2.38 | 3.44 | 1.56E-07 | 4.97E-07 |
| RP11-314D7.1 | 3.38 | 2.60 | 1.56E-07 | 4.98E-07 |
| RP11-173A16.2 | 2.03 | 3.64 | 1.62E-07 | 5.16E-07 |
| RP11-555G19.1 | 4.23 | 3.95 | 1.62E-07 | 5.17E-07 |
| CTA-398F10.1 | 2.20 | 2.65 | 1.66E-07 | 5.26E-07 |
| RP11-435O5.6 | 2.51 | 3.28 | 1.67E-07 | 5.30E-07 |
| FGF12-AS2 | 2.39 | 3.24 | 1.76E-07 | 5.56E-07 |
| AC053503.11 | 2.24 | 2.70 | 1.84E-07 | 5.81E-07 |
| GPR1-AS | 4.80 | 4.86 | 1.85E-07 | 5.83E-07 |
| RP11-615J4.3 | 4.35 | 3.06 | 1.88E-07 | 5.93E-07 |
| RP11-397A16.1 | 3.64 | 3.90 | 1.90E-07 | 5.97E-07 |
| CTC-575I10.1 | 2.01 | 2.74 | 2.01E-07 | 6.28E-07 |
| AC106875.1 | 4.29 | 5.23 | 2.05E-07 | 6.43E-07 |
| RP11-89M20.2 | 3.92 | 2.85 | 2.13E-07 | 6.66E-07 |
| AC012594.1 | 2.29 | 2.89 | 2.16E-07 | 6.74E-07 |
| RP11-300M24.1 | 3.27 | 3.00 | 2.16E-07 | 6.75E-07 |
| FOXP1-IT1 | 2.36 | 4.74 | 2.21E-07 | 6.88E-07 |
| RP11-546O6.4 | 2.15 | 2.91 | 2.23E-07 | 6.95E-07 |
| RP11-118E18.2 | 3.13 | 2.62 | 2.25E-07 | 7.01E-07 |
| CTD-2128A3.2 | 3.84 | 3.15 | 2.28E-07 | 7.08E-07 |
| AC114730.5 | 2.27 | 3.34 | 2.41E-07 | 7.48E-07 |
| bP-2171C21.3 | 3.61 | 3.09 | 2.45E-07 | 7.58E-07 |
| CHODL-AS1 | 3.00 | 2.83 | 2.45E-07 | 7.58E-07 |
| RP11-235G24.3 | 4.48 | 3.31 | 2.46E-07 | 7.60E-07 |
| RP11-2A4.4 | 4.59 | 4.36 | 2.50E-07 | 7.74E-07 |
| CTC-420A11.2 | 2.92 | 4.36 | 2.55E-07 | 7.87E-07 |
| RP11-118E18.4 | 2.31 | 3.20 | 2.56E-07 | 7.91E-07 |
| LINC01346 | 4.51 | 4.15 | 2.56E-07 | 7.91E-07 |
| RP11-197K6.1 | 4.82 | 4.56 | 2.61E-07 | 8.06E-07 |
| NHS-AS1 | 2.42 | 2.74 | 2.64E-07 | 8.14E-07 |
| RP11-319E12.2 | 3.04 | 2.73 | 2.71E-07 | 8.32E-07 |
| RP5-1011O1.2 | 2.22 | 4.08 | 2.73E-07 | 8.40E-07 |
| RP11-109M17.2 | 3.17 | 4.21 | 2.73E-07 | 8.40E-07 |
| GS1-279B7.1 | 2.12 | 3.04 | 2.78E-07 | 8.54E-07 |
| RP11-416N4.4 | 2.44 | 2.64 | 2.80E-07 | 8.60E-07 |
| SCHLAP1 | 4.00 | 3.10 | 2.90E-07 | 8.90E-07 |
| C11orf44 | 2.34 | 3.20 | 2.93E-07 | 8.97E-07 |
| RP11-280G9.1 | 2.82 | 4.02 | 2.96E-07 | 9.07E-07 |
| AC006372.5 | 3.44 | 3.40 | 2.99E-07 | 9.15E-07 |
| RP11-1105O14.1 | 2.94 | 4.02 | 3.02E-07 | 9.22E-07 |
| RP11-81K13.1 | 3.02 | 3.34 | 3.13E-07 | 9.55E-07 |
| LINC01831 | 4.95 | 3.39 | 3.14E-07 | 9.57E-07 |
| AC011288.2 | 2.33 | 3.26 | 3.18E-07 | 9.68E-07 |
| LINC01992 | 4.28 | 4.04 | 3.21E-07 | 9.76E-07 |
| AC005550.3 | 2.98 | 5.54 | 3.30E-07 | 1.00E-06 |
| RP5-1170K4.7 | 2.42 | 3.05 | 3.32E-07 | 1.01E-06 |
| AC104088.1 | 3.02 | 3.95 | 3.44E-07 | 1.04E-06 |
| CTA-392C11.1 | 4.92 | 4.27 | 3.55E-07 | 1.07E-06 |
| C3orf67-AS1 | 2.80 | 2.56 | 3.58E-07 | 1.08E-06 |
| LINC01896 | 5.07 | 4.18 | 3.62E-07 | 1.09E-06 |
| LINC01312 | 3.32 | 3.26 | 3.74E-07 | 1.13E-06 |
| LINC02141 | 3.93 | 3.51 | 3.78E-07 | 1.14E-06 |
| RP11-394A14.2 | 2.78 | 2.66 | 3.91E-07 | 1.18E-06 |
| LINC01502 | 2.87 | 5.00 | 3.97E-07 | 1.20E-06 |
| LINC01411 | 2.72 | 3.03 | 4.02E-07 | 1.21E-06 |
| CTD-2587H24.5 | 2.08 | 3.32 | 4.07E-07 | 1.22E-06 |
| RP11-15G16.1 | 3.30 | 3.02 | 4.23E-07 | 1.27E-06 |
| LINC01391 | 3.47 | 3.21 | 4.26E-07 | 1.28E-06 |
| AC006000.5 | 2.83 | 3.20 | 4.50E-07 | 1.35E-06 |
| SC22CB-56B3.1 | 3.89 | 2.82 | 4.74E-07 | 1.41E-06 |
| AC016710.1 | 3.95 | 2.86 | 5.02E-07 | 1.50E-06 |
| LINC01221 | 3.52 | 2.69 | 5.18E-07 | 1.54E-06 |
| CTD-2384A14.1 | 4.04 | 4.03 | 5.22E-07 | 1.55E-06 |
| RP11-300E4.2 | 2.33 | 3.10 | 5.29E-07 | 1.57E-06 |
| AP000439.3 | 3.15 | 4.51 | 5.35E-07 | 1.59E-06 |
| RP11-1B20.1 | 2.62 | 3.13 | 5.38E-07 | 1.59E-06 |
| LINC01819 | 2.82 | 6.49 | 5.80E-07 | 1.71E-06 |
| AP000688.29 | 2.67 | 5.74 | 6.13E-07 | 1.80E-06 |
| AC005150.1 | 3.74 | 2.74 | 6.19E-07 | 1.81E-06 |
| RP11-527N22.2 | 2.10 | 3.84 | 6.36E-07 | 1.86E-06 |
| RP11-400D2.2 | 4.22 | 3.11 | 6.42E-07 | 1.88E-06 |
| GRM7-AS3 | 3.07 | 2.50 | 6.53E-07 | 1.91E-06 |
| RP11-15M15.1 | 3.07 | 2.73 | 6.79E-07 | 1.98E-06 |
| RP11-244B22.11 | 5.35 | 3.86 | 7.02E-07 | 2.04E-06 |
| RP11-123O10.3 | 2.96 | 2.54 | 7.18E-07 | 2.08E-06 |
| RP11-110L15.2 | 2.34 | 2.86 | 7.20E-07 | 2.09E-06 |
| RP11-161I2.1 | 3.26 | 3.84 | 7.43E-07 | 2.15E-06 |
| OTX2-AS1 | 3.21 | 3.42 | 7.48E-07 | 2.17E-06 |
| LINC01444 | 3.65 | 3.92 | 7.61E-07 | 2.20E-06 |
| RP11-311F12.1 | 2.27 | 5.38 | 7.75E-07 | 2.24E-06 |
| IL20RB-AS1 | 2.42 | 2.73 | 7.80E-07 | 2.25E-06 |
| WI2-85898F10.1 | 2.12 | 3.63 | 7.96E-07 | 2.30E-06 |
| TESC-AS1 | 2.22 | 3.75 | 8.00E-07 | 2.31E-06 |
| RP13-895J2.6 | 3.21 | 3.91 | 8.12E-07 | 2.34E-06 |
| CTC-338M12.9 | 2.30 | 3.91 | 8.15E-07 | 2.35E-06 |
| RP11-69C13.1 | 2.52 | 2.57 | 8.25E-07 | 2.37E-06 |
| AC104389.28 | 2.67 | 5.06 | 8.37E-07 | 2.41E-06 |
| RBAKDN | 2.01 | 4.06 | 8.56E-07 | 2.46E-06 |
| RP11-817J15.3 | 2.52 | 3.98 | 9.24E-07 | 2.65E-06 |
| RP1-209A6.1 | 2.35 | 3.41 | 9.48E-07 | 2.71E-06 |
| CTD-2374C24.1 | 3.71 | 3.01 | 9.57E-07 | 2.73E-06 |
| RP11-149B9.2 | 5.19 | 4.07 | 9.87E-07 | 2.81E-06 |
| LINC01885 | 3.14 | 2.52 | 1.05E-06 | 2.98E-06 |
| AC002511.2 | 2.44 | 4.04 | 1.05E-06 | 2.99E-06 |
| LINC01959 | 2.98 | 2.56 | 1.07E-06 | 3.03E-06 |
| RP11-702B10.2 | 2.38 | 4.37 | 1.10E-06 | 3.11E-06 |
| RP11-268G12.1 | 2.76 | 3.43 | 1.11E-06 | 3.15E-06 |
| RP11-297P16.4 | 4.60 | 3.74 | 1.12E-06 | 3.16E-06 |
| RP11-320G10.1 | 3.30 | 3.92 | 1.12E-06 | 3.17E-06 |
| RP5-921G16.1 | 2.94 | 3.33 | 1.13E-06 | 3.19E-06 |
| RP11-545D19.1 | 3.16 | 2.68 | 1.15E-06 | 3.24E-06 |
| LINC00705 | 2.46 | 3.02 | 1.18E-06 | 3.32E-06 |
| PAQR9-AS1 | 3.68 | 3.72 | 1.18E-06 | 3.32E-06 |
| NAALADL2-AS3 | 3.21 | 2.82 | 1.21E-06 | 3.38E-06 |
| RP11-501C14.5 | 3.65 | 2.77 | 1.21E-06 | 3.39E-06 |
| ZBTB46-AS1 | 2.54 | 3.44 | 1.26E-06 | 3.51E-06 |
| LINC01143 | 2.50 | 3.56 | 1.26E-06 | 3.54E-06 |
| RP11-78C3.1 | 2.21 | 2.92 | 1.34E-06 | 3.73E-06 |
| RP1-261G23.5 | 2.59 | 3.01 | 1.34E-06 | 3.75E-06 |
| RP11-14K3.7 | 3.35 | 2.59 | 1.39E-06 | 3.86E-06 |
| LINC01854 | 3.57 | 2.74 | 1.39E-06 | 3.87E-06 |
| MACC1-AS1 | 2.27 | 3.93 | 1.44E-06 | 3.99E-06 |
| RP11-157L3.12 | 2.12 | 2.86 | 1.44E-06 | 4.01E-06 |
| RP11-1250I15.3 | 2.44 | 3.32 | 1.46E-06 | 4.06E-06 |
| E2F3-IT1 | 2.88 | 2.87 | 1.49E-06 | 4.11E-06 |
| LINC01036 | 2.83 | 3.20 | 1.50E-06 | 4.15E-06 |
| RP11-382E9.1 | 2.98 | 2.48 | 1.50E-06 | 4.16E-06 |
| RP11-526P6.1 | 3.49 | 2.68 | 1.50E-06 | 4.16E-06 |
| CTC-264K15.6 | 2.97 | 3.08 | 1.55E-06 | 4.27E-06 |
| RP1-116K23.1 | 3.76 | 2.73 | 1.55E-06 | 4.29E-06 |
| RP11-319F12.2 | 3.91 | 3.28 | 1.60E-06 | 4.41E-06 |
| RP3-446N13.5 | 3.66 | 2.97 | 1.62E-06 | 4.46E-06 |
| RP11-269F21.2 | 3.26 | 2.77 | 1.64E-06 | 4.51E-06 |
| RP11-626P14.1 | 2.77 | 2.84 | 1.65E-06 | 4.54E-06 |
| RP11-52J3.3 | 3.70 | 3.38 | 1.67E-06 | 4.60E-06 |
| IGF2-AS | 2.59 | 4.60 | 1.68E-06 | 4.62E-06 |
| LINC00051 | 2.91 | 3.45 | 1.69E-06 | 4.63E-06 |
| RP11-420B22.1 | 3.00 | 2.99 | 1.70E-06 | 4.67E-06 |
| FGF12-AS3 | 2.39 | 2.84 | 1.72E-06 | 4.73E-06 |
| RP11-722M1.1 | 3.45 | 3.23 | 1.78E-06 | 4.86E-06 |
| MRGPRG-AS1 | 3.29 | 2.77 | 1.80E-06 | 4.92E-06 |
| RP11-70J12.1 | 3.52 | 3.55 | 1.83E-06 | 4.98E-06 |
| RP11-95P13.2 | 4.23 | 3.07 | 1.84E-06 | 5.03E-06 |
| AC074389.5 | 3.49 | 3.56 | 1.85E-06 | 5.04E-06 |
| RP11-308D13.3 | 2.65 | 3.03 | 1.92E-06 | 5.22E-06 |
| LINC01697 | 2.40 | 4.66 | 1.94E-06 | 5.27E-06 |
| RAPGEF4-AS1 | 2.41 | 3.04 | 1.95E-06 | 5.31E-06 |
| CTD-2552K11.2 | 2.32 | 2.52 | 2.01E-06 | 5.45E-06 |
| RP11-619J20.1 | 2.70 | 2.68 | 2.03E-06 | 5.52E-06 |
| RP11-19J5.2 | 2.10 | 2.73 | 2.06E-06 | 5.57E-06 |
| AC012065.4 | 3.60 | 2.69 | 2.09E-06 | 5.65E-06 |
| FLJ36000 | 3.96 | 3.30 | 2.10E-06 | 5.68E-06 |
| RP11-404J23.1 | 3.41 | 2.61 | 2.12E-06 | 5.75E-06 |
| PLCB1-IT1 | 3.54 | 2.71 | 2.15E-06 | 5.82E-06 |
| RP1-97J1.2 | 3.32 | 3.35 | 2.21E-06 | 5.96E-06 |
| LINC01593 | 2.29 | 2.67 | 2.23E-06 | 6.01E-06 |
| RP11-177F15.1 | 2.46 | 3.09 | 2.25E-06 | 6.06E-06 |
| RP11-364P22.2 | 2.11 | 2.86 | 2.25E-06 | 6.06E-06 |
| AF178030.2 | 3.29 | 3.33 | 2.28E-06 | 6.13E-06 |
| LINC01665 | 3.35 | 2.93 | 2.32E-06 | 6.23E-06 |
| RP11-838N2.5 | 2.21 | 3.76 | 2.34E-06 | 6.28E-06 |
| RP11-153K16.1 | 2.98 | 2.96 | 2.34E-06 | 6.29E-06 |
| RP11-655G22.1 | 3.16 | 2.53 | 2.35E-06 | 6.32E-06 |
| RP11-141A19.1 | 2.67 | 2.74 | 2.42E-06 | 6.50E-06 |
| RP11-69G7.1 | 3.11 | 3.64 | 2.63E-06 | 6.99E-06 |
| GACAT2 | 2.03 | 3.16 | 2.66E-06 | 7.08E-06 |
| AC006262.4 | 2.29 | 2.60 | 2.66E-06 | 7.08E-06 |
| AC133106.2 | 2.89 | 2.65 | 2.68E-06 | 7.11E-06 |
| RP11-412H8.2 | 2.52 | 5.63 | 2.69E-06 | 7.14E-06 |
| RP13-895J2.3 | 2.41 | 3.91 | 2.73E-06 | 7.24E-06 |
| AL078471.5 | 3.80 | 2.93 | 2.86E-06 | 7.56E-06 |
| RP11-61J19.2 | 2.25 | 2.77 | 2.87E-06 | 7.60E-06 |
| RP11-438B23.2 | 2.34 | 4.27 | 2.88E-06 | 7.62E-06 |
| RP5-944M2.2 | 2.40 | 2.52 | 2.93E-06 | 7.74E-06 |
| AF131216.5 | 2.59 | 3.72 | 3.15E-06 | 8.30E-06 |
| CTD-2515C13.2 | 3.50 | 2.75 | 3.20E-06 | 8.42E-06 |
| LINC01433 | 2.28 | 4.12 | 3.26E-06 | 8.56E-06 |
| CTA-992D9.6 | 4.31 | 3.14 | 3.27E-06 | 8.59E-06 |
| RP11-19C24.1 | 2.81 | 2.43 | 3.34E-06 | 8.76E-06 |
| AC006552.1 | 2.69 | 2.82 | 3.61E-06 | 9.42E-06 |
| LINC01250 | 2.21 | 2.80 | 3.63E-06 | 9.46E-06 |
| RP11-429B14.4 | 2.65 | 2.58 | 3.64E-06 | 9.49E-06 |
| AP000997.1 | 2.36 | 2.49 | 3.66E-06 | 9.53E-06 |
| AC008060.7 | 3.54 | 2.78 | 3.68E-06 | 9.58E-06 |
| AC022173.2 | 2.45 | 3.72 | 3.73E-06 | 9.70E-06 |
| RP11-344E13.4 | 2.85 | 3.05 | 3.74E-06 | 9.72E-06 |
| RP1-135L22.1 | 4.33 | 3.44 | 3.79E-06 | 9.84E-06 |
| RP3-359N14.2 | 2.09 | 2.52 | 3.79E-06 | 9.86E-06 |
| RP4-660H19.1 | 3.03 | 3.94 | 3.81E-06 | 9.90E-06 |
| AC105760.3 | 2.24 | 2.80 | 3.85E-06 | 1.00E-05 |
| RP11-142O6.1 | 2.02 | 4.15 | 3.86E-06 | 1.00E-05 |
| RP1-170O19.14 | 3.13 | 2.74 | 3.91E-06 | 1.01E-05 |
| RP11-488I20.9 | 4.42 | 4.39 | 4.06E-06 | 1.05E-05 |
| RP11-711K1.7 | 2.34 | 2.81 | 4.06E-06 | 1.05E-05 |
| GS1-72M22.1 | 2.32 | 3.92 | 4.08E-06 | 1.06E-05 |
| RP3-443C4.2 | 2.59 | 2.55 | 4.13E-06 | 1.07E-05 |
| RP11-142A22.4 | 2.79 | 4.47 | 4.20E-06 | 1.09E-05 |
| RP11-509E10.1 | 3.10 | 2.53 | 4.28E-06 | 1.10E-05 |
| RP11-818F20.4 | 3.45 | 3.86 | 4.43E-06 | 1.14E-05 |
| AP000998.2 | 2.92 | 2.64 | 4.43E-06 | 1.14E-05 |
| CTD-2315E11.1 | 2.33 | 3.32 | 4.45E-06 | 1.15E-05 |
| RP11-324L17.1 | 2.17 | 3.71 | 4.56E-06 | 1.17E-05 |
| CTD-2194D22.3 | 3.47 | 2.81 | 4.58E-06 | 1.18E-05 |
| PCAT18 | 2.31 | 4.83 | 4.82E-06 | 1.23E-05 |
| RP11-269G24.2 | 2.33 | 2.98 | 4.85E-06 | 1.24E-05 |
| CTD-2331D11.4 | 2.98 | 2.49 | 4.93E-06 | 1.26E-05 |
| RP11-369C8.1 | 3.61 | 3.97 | 4.98E-06 | 1.27E-05 |
| RP11-556I14.1 | 2.53 | 3.24 | 5.04E-06 | 1.29E-05 |
| RP11-65J3.3 | 2.13 | 3.87 | 5.06E-06 | 1.29E-05 |
| RP11-148E17.1 | 3.55 | 3.23 | 5.24E-06 | 1.33E-05 |
| RP11-410N8.3 | 2.04 | 2.93 | 5.27E-06 | 1.34E-05 |
| RP11-12K22.1 | 3.54 | 2.96 | 5.34E-06 | 1.36E-05 |
| RP11-614F17.2 | 3.22 | 3.30 | 5.38E-06 | 1.37E-05 |
| RP11-36N20.1 | 2.19 | 3.89 | 5.39E-06 | 1.37E-05 |
| LINC00456 | 2.48 | 2.51 | 5.50E-06 | 1.40E-05 |
| CTD-2021H9.2 | 2.51 | 2.62 | 5.71E-06 | 1.44E-05 |
| LINC01322 | 2.92 | 4.58 | 5.72E-06 | 1.45E-05 |
| RP11-583F2.5 | 2.56 | 3.65 | 5.84E-06 | 1.48E-05 |
| RP1-232L24.3 | 2.23 | 4.60 | 5.86E-06 | 1.48E-05 |
| RP11-319E16.1 | 2.34 | 2.68 | 5.99E-06 | 1.51E-05 |
| RP11-100N20.1 | 2.91 | 2.66 | 6.10E-06 | 1.53E-05 |
| AC093702.1 | 2.15 | 3.43 | 6.11E-06 | 1.54E-05 |
| RP11-802F5.1 | 2.53 | 2.86 | 6.19E-06 | 1.55E-05 |
| LINC01744 | 2.42 | 2.59 | 6.25E-06 | 1.57E-05 |
| CTD-2066L21.1 | 2.87 | 2.76 | 6.25E-06 | 1.57E-05 |
| LINC00879 | 4.16 | 3.14 | 6.28E-06 | 1.58E-05 |
| CTC-348L5.1 | 2.16 | 2.48 | 6.37E-06 | 1.60E-05 |
| LINC01886 | 3.83 | 4.19 | 6.44E-06 | 1.61E-05 |
| AC008063.3 | 2.13 | 3.10 | 6.45E-06 | 1.61E-05 |
| RP11-706D8.3 | 2.62 | 2.78 | 6.45E-06 | 1.61E-05 |
| LINC00867 | 2.17 | 3.27 | 6.49E-06 | 1.62E-05 |
| AC008088.4 | 2.02 | 2.58 | 6.53E-06 | 1.63E-05 |
| LINC01448 | 3.16 | 2.66 | 6.55E-06 | 1.64E-05 |
| TDRG1 | 2.77 | 2.74 | 6.65E-06 | 1.66E-05 |
| RP11-14C10.5 | 2.15 | 2.83 | 6.67E-06 | 1.66E-05 |
| AC091814.2 | 2.93 | 4.10 | 6.71E-06 | 1.67E-05 |
| RP11-274H24.1 | 2.45 | 3.73 | 6.94E-06 | 1.73E-05 |
| RP11-688I9.2 | 3.11 | 2.74 | 6.97E-06 | 1.73E-05 |
| RP11-355F22.1 | 2.81 | 2.55 | 7.33E-06 | 1.82E-05 |
| RP11-329J18.3 | 2.04 | 3.43 | 7.58E-06 | 1.88E-05 |
| RP11-12A16.3 | 2.48 | 3.03 | 7.59E-06 | 1.88E-05 |
| LINC02078 | 2.05 | 3.84 | 7.72E-06 | 1.91E-05 |
| RP11-749H17.2 | 3.08 | 2.75 | 7.73E-06 | 1.91E-05 |
| AL132709.1 | 3.39 | 2.79 | 7.87E-06 | 1.94E-05 |
| AC068535.2 | 3.89 | 2.78 | 8.07E-06 | 1.98E-05 |
| CTD-2251F13.1 | 3.10 | 2.94 | 8.19E-06 | 2.01E-05 |
| AC002463.3 | 3.31 | 2.89 | 8.23E-06 | 2.02E-05 |
| CYMP-AS1 | 2.82 | 4.46 | 8.42E-06 | 2.07E-05 |
| LINC01087 | 2.64 | 3.17 | 8.49E-06 | 2.08E-05 |
| C10orf126 | 3.10 | 3.34 | 8.51E-06 | 2.08E-05 |
| RP11-552M14.1 | 2.80 | 2.56 | 8.54E-06 | 2.09E-05 |
| RP11-631F7.1 | 2.32 | 2.89 | 8.65E-06 | 2.12E-05 |
| RP11-124N3.3 | 4.11 | 3.15 | 9.08E-06 | 2.22E-05 |
| RP11-685A21.1 | 2.91 | 2.86 | 9.15E-06 | 2.23E-05 |
| RP11-307P5.1 | 2.76 | 4.26 | 9.38E-06 | 2.28E-05 |
| CTB-147C22.9 | 2.62 | 2.78 | 9.54E-06 | 2.32E-05 |
| RP11-220C2.1 | 2.18 | 2.85 | 9.55E-06 | 2.32E-05 |
| RP11-527L4.6 | 2.98 | 3.82 | 9.58E-06 | 2.33E-05 |
| RP11-108E14.1 | 4.53 | 3.34 | 9.67E-06 | 2.35E-05 |
| CH507-528H12.1 | 2.54 | 3.19 | 9.73E-06 | 2.36E-05 |
| LINC02046 | 3.38 | 3.08 | 9.86E-06 | 2.39E-05 |
| AC008277.1 | 2.07 | 4.17 | 9.86E-06 | 2.39E-05 |
| MIR7-3HG | 2.97 | 4.05 | 1.01E-05 | 2.44E-05 |
| EPHA5-AS1 | 3.01 | 3.32 | 1.06E-05 | 2.55E-05 |
| AC004009.3 | 3.43 | 2.69 | 1.06E-05 | 2.55E-05 |
| RP11-206M11.7 | 3.77 | 5.19 | 1.08E-05 | 2.61E-05 |
| RP11-392B6.1 | 2.30 | 3.60 | 1.08E-05 | 2.61E-05 |
| RP11-129M6.1 | 2.12 | 5.98 | 1.11E-05 | 2.68E-05 |
| LINC00403 | 3.12 | 2.83 | 1.12E-05 | 2.69E-05 |
| RP11-91I20.4 | 2.38 | 3.03 | 1.15E-05 | 2.75E-05 |
| AC010145.4 | 3.78 | 2.76 | 1.20E-05 | 2.87E-05 |
| RP11-524H19.2 | 2.21 | 3.20 | 1.20E-05 | 2.87E-05 |
| RP1-290I10.5 | 3.25 | 3.15 | 1.23E-05 | 2.93E-05 |
| RP11-305B6.3 | 3.21 | 2.79 | 1.27E-05 | 3.04E-05 |
| RP11-268G13.1 | 2.89 | 2.67 | 1.28E-05 | 3.04E-05 |
| RP4-784A16.4 | 2.02 | 2.66 | 1.42E-05 | 3.37E-05 |
| AC096669.1 | 2.94 | 2.54 | 1.47E-05 | 3.49E-05 |
| CTB-1I21.1 | 2.93 | 2.93 | 1.47E-05 | 3.49E-05 |
| RP11-642D21.2 | 2.46 | 2.82 | 1.50E-05 | 3.55E-05 |
| RP11-6B19.1 | 2.51 | 3.74 | 1.53E-05 | 3.61E-05 |
| LINC01788 | 2.92 | 2.82 | 1.54E-05 | 3.64E-05 |
| MIR4300HG | 2.58 | 2.77 | 1.55E-05 | 3.66E-05 |
| RP11-61O11.1 | 3.95 | 3.42 | 1.62E-05 | 3.80E-05 |
| RP11-403I13.5 | 2.02 | 4.26 | 1.68E-05 | 3.93E-05 |
| RP11-445N18.5 | 2.24 | 3.54 | 1.72E-05 | 4.02E-05 |
| DPYD-AS2 | 2.71 | 3.73 | 1.73E-05 | 4.04E-05 |
| RP11-268G12.3 | 2.36 | 3.10 | 1.75E-05 | 4.09E-05 |
| AF127577.11 | 2.10 | 3.78 | 1.76E-05 | 4.11E-05 |
| RP11-1041F24.1 | 3.31 | 2.69 | 1.87E-05 | 4.37E-05 |
| ZBTB20-AS5 | 2.56 | 4.04 | 1.91E-05 | 4.44E-05 |
| CACNA1C-IT3 | 3.04 | 2.59 | 2.01E-05 | 4.66E-05 |
| RP11-449P1.1 | 2.59 | 2.62 | 2.04E-05 | 4.72E-05 |
| B3GALT5-AS1 | 2.18 | 5.81 | 2.22E-05 | 5.10E-05 |
| RP11-117L5.4 | 2.51 | 2.74 | 2.28E-05 | 5.24E-05 |
| LINC01551 | 2.92 | 2.96 | 2.34E-05 | 5.36E-05 |
| RP11-998D10.4 | 3.57 | 3.01 | 2.46E-05 | 5.62E-05 |
| RP11-264L1.4 | 2.09 | 3.19 | 2.48E-05 | 5.65E-05 |
| RP11-648K4.2 | 3.01 | 2.66 | 2.53E-05 | 5.78E-05 |
| RP5-944M2.1 | 2.25 | 2.62 | 2.54E-05 | 5.78E-05 |
| RP11-52J3.2 | 2.51 | 3.28 | 2.58E-05 | 5.87E-05 |
| RP11-1081M5.1 | 2.15 | 3.73 | 2.62E-05 | 5.95E-05 |
| RP11-11N5.3 | 2.26 | 2.54 | 2.65E-05 | 6.02E-05 |
| ANO1-AS2 | 2.03 | 3.07 | 2.68E-05 | 6.07E-05 |
| AC003958.2 | 2.39 | 2.82 | 2.70E-05 | 6.11E-05 |
| RP11-25C19.3 | 2.99 | 3.18 | 2.77E-05 | 6.27E-05 |
| DIO2-AS1 | 2.21 | 2.50 | 2.79E-05 | 6.31E-05 |
| PHACTR2-AS1 | 2.14 | 3.57 | 2.89E-05 | 6.51E-05 |
| RP1-276N6.2 | 3.91 | 3.11 | 3.10E-05 | 6.96E-05 |
| RP11-124N3.2 | 3.65 | 2.91 | 3.13E-05 | 7.04E-05 |
| AP000997.2 | 2.11 | 2.69 | 3.28E-05 | 7.35E-05 |
| RP11-25B7.1 | 2.13 | 2.69 | 3.34E-05 | 7.47E-05 |
| LINC01940 | 2.18 | 3.99 | 3.37E-05 | 7.53E-05 |
| RP11-311F12.2 | 2.22 | 3.61 | 3.41E-05 | 7.60E-05 |
| RP11-1D12.1 | 2.88 | 2.90 | 3.49E-05 | 7.77E-05 |
| LINC01976 | 2.21 | 2.61 | 3.52E-05 | 7.85E-05 |
| AC007731.1 | 2.25 | 2.83 | 3.59E-05 | 7.98E-05 |
| RP1-46F2.3 | 2.40 | 3.27 | 3.62E-05 | 8.05E-05 |
| RP11-301G7.1 | 2.84 | 2.69 | 3.76E-05 | 8.35E-05 |
| FER1L6-AS1 | 3.36 | 3.17 | 3.77E-05 | 8.38E-05 |
| CTB-4E7.1 | 2.14 | 2.67 | 3.81E-05 | 8.46E-05 |
| RP11-798K3.2 | 2.13 | 3.32 | 3.97E-05 | 8.78E-05 |
| RP11-669C19.1 | 2.05 | 2.62 | 3.99E-05 | 8.82E-05 |
| EGLN3-AS1 | 2.21 | 2.58 | 4.03E-05 | 8.92E-05 |
| RP11-474B12.1 | 2.04 | 3.13 | 4.05E-05 | 8.95E-05 |
| AC012506.2 | 2.62 | 2.60 | 4.16E-05 | 9.18E-05 |
| RP1-86D1.5 | 2.67 | 3.17 | 4.30E-05 | 9.50E-05 |
| RP11-1336O20.2 | 2.78 | 3.84 | 4.35E-05 | 9.59E-05 |
| LINC01192 | 2.76 | 2.88 | 4.45E-05 | 9.80E-05 |
| RP11-384C4.6 | 2.29 | 3.02 | 4.50E-05 | 9.89E-05 |
| C10orf71-AS1 | 2.16 | 3.21 | 4.52E-05 | 9.94E-05 |
| RP11-3B12.2 | 2.07 | 3.39 | 4.55E-05 | 1.00E-04 |
| RP11-305F18.1 | 2.72 | 3.45 | 4.76E-05 | 0.000104242 |
| RP11-704M14.1 | 2.58 | 3.57 | 4.80E-05 | 0.000105086 |
| RP11-486P11.1 | 2.02 | 3.19 | 5.00E-05 | 0.000109168 |
| LINC01470 | 2.12 | 2.76 | 5.09E-05 | 0.000110999 |
| ASTN2-AS1 | 2.32 | 2.87 | 5.31E-05 | 0.000115296 |
| RP11-31K23.2 | 3.16 | 2.72 | 5.34E-05 | 0.00011597 |
| RP11-1109M24.5 | 2.21 | 2.58 | 5.48E-05 | 0.000118742 |
| LINC00200 | 3.33 | 4.82 | 5.52E-05 | 0.00011947 |
| LINC02128 | 2.40 | 3.08 | 5.69E-05 | 0.00012275 |
| DSCR10 | 2.54 | 2.58 | 5.75E-05 | 0.000124006 |
| AC000111.6 | 2.28 | 4.16 | 5.94E-05 | 0.000128023 |
| DANT1 | 2.51 | 2.72 | 6.21E-05 | 0.000133516 |
| RP11-495K9.6 | 2.23 | 3.00 | 6.37E-05 | 0.000136822 |
| RP11-402L5.1 | 2.29 | 2.63 | 6.49E-05 | 0.000139045 |
| MTOR-AS1 | 2.05 | 2.78 | 6.59E-05 | 0.000141205 |
| LINC00488 | 2.94 | 3.03 | 6.75E-05 | 0.000144397 |
| RP11-342M21.2 | 2.64 | 2.99 | 6.93E-05 | 0.000147888 |
| LINC00440 | 2.48 | 2.82 | 6.97E-05 | 0.00014865 |
| LINC01179 | 2.20 | 2.76 | 7.07E-05 | 0.000150649 |
| CTA-392E5.1 | 2.32 | 3.31 | 7.09E-05 | 0.000150979 |
| RP11-549K20.1 | 2.22 | 3.40 | 7.29E-05 | 0.00015495 |
| RP11-966I7.2 | 3.02 | 2.56 | 7.50E-05 | 0.000158969 |
| RP11-255G12.3 | 2.35 | 2.64 | 7.63E-05 | 0.000161395 |
| CTD-2653B5.1 | 2.16 | 2.67 | 7.91E-05 | 0.000167015 |
| RP11-204N11.1 | 2.31 | 2.65 | 7.92E-05 | 0.000167169 |
| AC012363.4 | 2.70 | 2.61 | 7.96E-05 | 0.000167949 |
| RP11-92K15.1 | 2.09 | 2.74 | 8.18E-05 | 0.000172475 |
| RP11-433M22.2 | 2.59 | 4.05 | 8.25E-05 | 0.000173685 |
| ARHGEF3-AS1 | 2.50 | 2.54 | 8.36E-05 | 0.000175872 |
| LINC01574 | 2.19 | 2.66 | 8.53E-05 | 0.000179237 |
| RP11-162D9.3 | 2.57 | 3.48 | 8.54E-05 | 0.000179423 |
| RP11-401F2.4 | 2.19 | 2.80 | 8.58E-05 | 0.000180188 |
| CTB-61M7.1 | 3.49 | 3.26 | 8.94E-05 | 0.000186998 |
| RP1-269M15.3 | 2.70 | 4.13 | 9.02E-05 | 0.000188619 |
| LINC01425 | 3.07 | 2.91 | 9.20E-05 | 0.000192275 |
| RP11-274J7.2 | 2.14 | 2.84 | 9.53E-05 | 0.000198516 |
| RP3-428L16.1 | 2.56 | 3.64 | 9.64E-05 | 0.000200662 |
| CH507-513H4.6 | 2.06 | 3.11 | 9.86E-05 | 0.000204929 |
| RP11-338L22.2 | 2.08 | 3.05 | 0.000100364 | 0.000208193 |
| RP11-553K8.5 | 2.88 | 3.61 | 0.000102902 | 0.000213078 |
| CH507-513H4.3 | 3.71 | 4.35 | 0.000103976 | 0.000214994 |
| RP11-115H15.2 | 2.56 | 3.47 | 0.000104055 | 0.000215107 |
| AC010967.2 | 2.33 | 2.70 | 0.000106194 | 0.000219429 |
| AP000479.1 | 2.22 | 3.17 | 0.000109214 | 0.000225256 |
| RP11-122C21.1 | 2.71 | 2.94 | 0.000110617 | 0.000227835 |
| LINC01443 | 2.30 | 4.11 | 0.000113338 | 0.000232801 |
| RP11-448G4.4 | 2.64 | 2.75 | 0.000114436 | 0.000234896 |
| LINC00485 | 2.14 | 2.86 | 0.000115377 | 0.000236774 |
| AP004372.1 | 2.17 | 4.79 | 0.000116406 | 0.000238722 |
| RP11-302L19.1 | 2.44 | 2.72 | 0.000117076 | 0.000239954 |
| LINC02055 | 2.02 | 4.85 | 0.000120265 | 0.000245798 |
| MIR124-2HG | 2.80 | 2.44 | 0.000123141 | 0.000251503 |
| RP11-760D2.5 | 2.35 | 3.81 | 0.000126226 | 0.000257571 |
| AC106873.4 | 2.09 | 3.33 | 0.000127146 | 0.000259331 |
| LINC01580 | 2.67 | 2.56 | 0.000127562 | 0.000260121 |
| MIR3976HG | 3.34 | 2.86 | 0.000131606 | 0.0002677 |
| RP11-573N10.1 | 2.23 | 3.51 | 0.000132995 | 0.000270281 |
| RP11-712P20.2 | 2.46 | 3.30 | 0.00013424 | 0.000272503 |
| AC060834.3 | 2.70 | 2.67 | 0.000135684 | 0.000275311 |
| RP11-537P22.2 | 2.55 | 2.54 | 0.000140839 | 0.000285321 |
| AC016716.2 | 2.63 | 2.45 | 0.000157283 | 0.000316923 |
| CTC-458G6.4 | 2.98 | 2.67 | 0.000163261 | 0.000328087 |
| RP11-731N10.1 | 3.18 | 2.87 | 0.000166876 | 0.000334384 |
| RP11-417B4.2 | 2.17 | 2.85 | 0.00016897 | 0.000338052 |
| MIR670HG | 2.72 | 3.00 | 0.000175922 | 0.000350715 |
| AC009313.1 | 2.22 | 2.58 | 0.000180793 | 0.000359709 |
| RP11-286N22.10 | 2.20 | 3.80 | 0.000193016 | 0.000382759 |
| RP3-348I23.2 | 2.16 | 2.63 | 0.00020848 | 0.000411432 |
| RP11-436H11.5 | 3.08 | 2.81 | 0.000208551 | 0.000411482 |
| CTD-2050N2.1 | 2.27 | 2.53 | 0.000211763 | 0.000417272 |
| LINC01475 | 2.72 | 2.84 | 0.000217023 | 0.000426609 |
| AP000462.1 | 3.02 | 3.40 | 0.000226419 | 0.000443724 |
| RP11-327O17.2 | 2.58 | 3.77 | 0.000238163 | 0.000465523 |
| RP11-728G15.1 | 2.08 | 2.65 | 0.000241084 | 0.000470417 |
| LPP-AS1 | 2.59 | 3.21 | 0.00024449 | 0.00047665 |
| RP11-386B13.4 | 2.95 | 3.01 | 0.000248627 | 0.000483773 |
| RP11-733C7.1 | 2.44 | 4.04 | 0.000256611 | 0.000498123 |
| RP11-379J5.5 | 2.23 | 2.72 | 0.000263652 | 0.000510362 |
| AC022201.4 | 2.35 | 2.65 | 0.000290521 | 0.000558531 |
| LINC01878 | 2.19 | 2.42 | 0.000295661 | 0.000567324 |
| LINC01040 | 2.91 | 2.55 | 0.000296523 | 0.000568735 |
| RP11-120I21.2 | 2.48 | 2.57 | 0.000307519 | 0.000588323 |
| AC106876.2 | 2.36 | 4.59 | 0.000320613 | 0.000612333 |
| LINC01158 | 2.91 | 3.32 | 0.000340175 | 0.000646131 |
| BIRC6-AS1 | 2.28 | 3.01 | 0.000342951 | 0.00065113 |
| RP11-158G18.1 | 2.50 | 2.71 | 0.000348244 | 0.000660066 |
| RP11-774D14.1 | 2.60 | 5.68 | 0.000359685 | 0.000680605 |
| RP11-571I18.5 | 2.00 | 3.61 | 0.000395916 | 0.000744316 |
| CTD-3064C13.1 | 2.16 | 2.72 | 0.000454293 | 0.000847868 |
| ATG10-AS1 | 2.49 | 2.53 | 0.00046188 | 0.000860959 |
| LINC02008 | 2.53 | 2.82 | 0.000463902 | 0.000864012 |
| RP5-1119A7.17 | 2.81 | 3.05 | 0.000474309 | 0.000881937 |
| AP000462.2 | 2.22 | 3.46 | 0.000563452 | 0.001033607 |
| RP5-912I13.1 | 2.47 | 2.80 | 0.000578091 | 0.001057232 |
| RP11-545H22.1 | 2.40 | 2.62 | 0.000619223 | 0.001126972 |
| RP3-403L10.3 | 2.13 | 2.92 | 0.000648946 | 0.001177726 |
| RP11-318G21.4 | 2.67 | 3.21 | 0.000654845 | 0.001187475 |
| LINC02050 | 2.15 | 3.00 | 0.000669639 | 0.001212106 |
| HECW1-IT1 | 2.15 | 2.43 | 0.000675887 | 0.001222433 |
| MAGI1-AS1 | 2.06 | 3.42 | 0.000690028 | 0.001246756 |
| SLC9A9-AS1 | 2.10 | 2.47 | 0.000744521 | 0.001339037 |
| RP11-748C4.1 | 2.42 | 2.92 | 0.000807787 | 0.001446654 |
| CTD-2055G21.1 | 2.18 | 2.42 | 0.000816017 | 0.001460045 |
| RP11-58G13.1 | 2.33 | 2.86 | 0.000855712 | 0.001523803 |
| RP11-93B14.4 | 2.15 | 2.79 | 0.000860744 | 0.001532445 |
| LINC01205 | 2.27 | 3.04 | 0.000868641 | 0.001544995 |
| RP11-328K2.1 | 2.08 | 2.61 | 0.00088368 | 0.001570502 |
| RP1-310O13.13 | 2.57 | 3.03 | 0.00089585 | 0.001591503 |
| RP11-488I20.8 | 2.34 | 2.74 | 0.000907549 | 0.001610698 |
| RP11-578B16.1 | 2.13 | 2.68 | 0.000949902 | 0.001679804 |
| RP11-512N4.2 | 2.25 | 2.50 | 0.00095528 | 0.001688092 |
| AC023347.1 | 2.22 | 3.01 | 0.00096174 | 0.001698508 |
| RP11-328J6.1 | 2.26 | 5.07 | 0.001036976 | 0.001821381 |
| RP11-245A18.1 | 2.41 | 2.70 | 0.001052822 | 0.001846983 |
| CTD-2216M2.1 | 2.12 | 2.48 | 0.001062339 | 0.001862661 |
| RP11-152P17.3 | 2.31 | 2.67 | 0.001103561 | 0.001930053 |
| RP11-678G15.1 | 2.19 | 2.70 | 0.001112121 | 0.001943514 |
| CTC-546K23.1 | 2.51 | 2.87 | 0.001170453 | 0.002040307 |
| LINC01793 | 2.68 | 3.35 | 0.001200685 | 0.002090581 |
| RP11-662G23.1 | 2.24 | 3.22 | 0.001345076 | 0.002328931 |
| RP11-807H7.2 | 2.18 | 2.61 | 0.001437767 | 0.002478937 |
| RP11-701I24.3 | 2.07 | 2.69 | 0.001447285 | 0.002493438 |
| RP11-190J23.1 | 2.29 | 2.57 | 0.00157033 | 0.002690501 |
| CTB-51A17.1 | 2.12 | 2.59 | 0.001695636 | 0.002893085 |
| RP11-39E3.3 | 2.20 | 2.43 | 0.001745921 | 0.002972686 |
| CACNA1C-AS4 | 2.19 | 3.12 | 0.001851434 | 0.003139283 |
| ZBTB20-AS3 | 2.24 | 2.82 | 0.002125528 | 0.003563781 |
| RP11-539E19.2 | 2.19 | 2.67 | 0.002137314 | 0.003582236 |
| RP11-459O1.2 | 2.02 | 2.97 | 0.002142236 | 0.003589789 |
| GPC6-AS1 | 2.02 | 2.59 | 0.002265619 | 0.003778968 |
| RP4-564F22.6 | 2.18 | 2.71 | 0.002297735 | 0.003826868 |
| RP11-39M21.1 | 2.09 | 2.87 | 0.002734755 | 0.0044957 |
| RP11-40C11.2 | 2.44 | 2.74 | 0.002854761 | 0.004680225 |
| RP11-310J24.3 | 2.26 | 3.12 | 0.002896461 | 0.004743349 |
| RP11-369F10.2 | 2.06 | 2.50 | 0.004179728 | 0.006658439 |
| LINC02106 | 2.37 | 3.33 | 0.005513749 | 0.008611468 |
| CTD-2089N3.1 | 2.06 | 3.51 | 0.005690021 | 0.008862185 |
| RP1-53O8.2 | 2.00 | 2.57 | 0.006897419 | 0.010596121 |
| RP11-232D9.3 | 2.19 | 2.90 | 0.007076269 | 0.010839447 |

Table S7. The significantly dysregulated lncRNAs in lung squamous cell carcinoma in the TCGA database

| LncRNA | logFC | logCPM | P-value | FDR |
| --- | --- | --- | --- | --- |
| AC093110.3 | -3.83 | 7.53 | 9.50E-306 | 8.50E-302 |
| LANCL1-AS1 | -4.15 | 6.37 | 1.63E-287 | 7.31E-284 |
| RP11-371A19.2 | -5.43 | 5.51 | 3.84E-203 | 1.15E-199 |
| RP1-78O14.1 | -4.15 | 7.13 | 2.06E-194 | 4.62E-191 |
| AC090616.2 | -4.05 | 6.80 | 7.55E-185 | 1.35E-181 |
| RP11-434D9.1 | -3.73 | 5.56 | 1.06E-182 | 1.58E-179 |
| RP5-826L7.1 | -4.15 | 3.27 | 9.33E-177 | 1.19E-173 |
| LINC00968 | -4.36 | 7.00 | 6.10E-176 | 6.83E-173 |
| HSPC324 | -4.37 | 5.48 | 8.51E-175 | 8.47E-172 |
| ADAMTS9-AS2 | -3.56 | 6.00 | 5.62E-174 | 5.03E-171 |
| AP001189.4 | -3.95 | 6.09 | 6.65E-166 | 5.42E-163 |
| RP11-677M14.3 | -3.11 | 5.88 | 1.37E-165 | 1.02E-162 |
| RP11-354P11.2 | -4.60 | 3.80 | 2.20E-163 | 1.52E-160 |
| HID1-AS1 | -4.00 | 4.43 | 7.24E-161 | 4.63E-158 |
| MIR3945HG | -4.23 | 6.33 | 7.84E-151 | 4.68E-148 |
| RP11-805I24.3 | -5.04 | 4.54 | 4.58E-147 | 2.57E-144 |
| LINC01082 | -4.48 | 3.72 | 3.98E-144 | 2.10E-141 |
| LINC02016 | -6.33 | 5.10 | 1.35E-142 | 6.70E-140 |
| RP11-264B14.1 | -3.85 | 3.45 | 2.99E-140 | 1.41E-137 |
| ADAMTS9-AS1 | -3.99 | 6.56 | 1.26E-138 | 5.65E-136 |
| CTD-2369P2.8 | -4.12 | 7.49 | 3.46E-135 | 1.48E-132 |
| FENDRR | -4.06 | 9.81 | 1.61E-134 | 6.54E-132 |
| RP11-136H19.1 | -3.04 | 3.75 | 7.11E-132 | 2.77E-129 |
| RP11-613D13.8 | -3.80 | 5.59 | 5.42E-130 | 2.02E-127 |
| SFTA1P | -4.54 | 9.83 | 3.49E-128 | 1.25E-125 |
| RP11-1024P17.1 | -2.94 | 6.32 | 1.16E-127 | 4.01E-125 |
| AC018647.3 | -3.64 | 4.88 | 3.44E-127 | 1.14E-124 |
| RP11-582J16.4 | -2.16 | 6.56 | 2.62E-125 | 8.40E-123 |
| RP11-541N10.3 | -2.36 | 8.33 | 1.77E-122 | 5.46E-120 |
| RP4-575N6.5 | -3.41 | 3.51 | 1.03E-119 | 3.06E-117 |
| CTD-2530N21.5 | -4.42 | 3.36 | 2.98E-116 | 8.61E-114 |
| RP11-100L22.1 | -4.13 | 3.14 | 3.98E-116 | 1.11E-113 |
| RP11-714G18.1 | -3.15 | 4.19 | 3.63E-115 | 9.86E-113 |
| RP11-598F7.3 | -4.22 | 6.25 | 4.10E-115 | 1.08E-112 |
| RP11-336K24.5 | -4.26 | 3.87 | 9.58E-114 | 2.45E-111 |
| GATA6-AS1 | -3.22 | 5.73 | 1.27E-113 | 3.17E-111 |
| KCNMB2-AS1 | 7.48 | 9.94 | 1.06E-110 | 2.57E-108 |
| RP11-635O16.2 | -5.28 | 5.23 | 5.00E-110 | 1.18E-107 |
| RP3-523K23.2 | 6.64 | 11.42 | 4.61E-109 | 1.06E-106 |
| RP4-575N6.4 | -3.09 | 4.80 | 7.54E-109 | 1.69E-106 |
| AC079630.4 | -3.80 | 8.66 | 8.41E-109 | 1.84E-106 |
| PCAT19 | -2.93 | 9.20 | 2.80E-107 | 5.96E-105 |
| LINC01645 | -3.93 | 3.46 | 3.62E-106 | 7.53E-104 |
| SMIM25 | -2.97 | 10.25 | 4.53E-106 | 9.22E-104 |
| RP11-352D13.6 | -3.73 | 5.69 | 4.62E-103 | 9.19E-101 |
| LINC00607 | -3.01 | 6.19 | 1.01E-102 | 1.96E-100 |
| AC006273.4 | -2.96 | 4.42 | 6.47E-102 | 1.23E-99 |
| LINC01572 | 3.29 | 6.92 | 2.93E-101 | 5.47E-99 |
| LINC01936 | -3.43 | 8.09 | 4.37E-101 | 7.99E-99 |
| LINC00891 | -3.42 | 4.56 | 6.77E-101 | 1.21E-98 |
| ACOXL-AS1 | -2.66 | 4.87 | 7.28E-101 | 1.28E-98 |
| RP11-672A2.4 | -3.19 | 6.11 | 7.95E-101 | 1.37E-98 |
| LHFPL3-AS2 | -4.23 | 8.52 | 7.98E-100 | 1.35E-97 |
| LDLRAD4-AS1 | -3.76 | 3.12 | 3.14E-99 | 5.21E-97 |
| AC008268.1 | -6.39 | 7.80 | 5.37E-96 | 8.74E-94 |
| AC011899.9 | -2.98 | 8.08 | 4.03E-95 | 6.45E-93 |
| PACRG-AS3 | -4.48 | 3.46 | 5.45E-95 | 8.57E-93 |
| RP11-259K15.2 | -4.29 | 6.24 | 7.34E-94 | 1.13E-91 |
| BBOX1-AS1 | 6.87 | 9.42 | 6.07E-93 | 9.22E-91 |
| RP11-8L2.1 | 7.83 | 7.30 | 6.94E-93 | 1.04E-90 |
| MIR497HG | -2.08 | 6.36 | 2.22E-92 | 3.27E-90 |
| RP11-79H23.3 | -3.27 | 6.84 | 3.37E-92 | 4.87E-90 |
| AC007128.1 | 6.45 | 7.13 | 1.75E-91 | 2.49E-89 |
| RP11-475O23.2 | -4.15 | 3.30 | 2.28E-91 | 3.20E-89 |
| RP11-286H15.1 | -3.58 | 5.09 | 6.24E-91 | 8.59E-89 |
| RP1-18D14.7 | -3.30 | 4.54 | 1.28E-90 | 1.74E-88 |
| LINC01290 | -2.37 | 4.75 | 5.20E-90 | 6.95E-88 |
| LINC00702 | -3.06 | 7.29 | 1.91E-89 | 2.52E-87 |
| RP11-357P18.2 | -2.97 | 5.25 | 2.54E-89 | 3.30E-87 |
| RP11-389C8.2 | -2.41 | 8.10 | 2.46E-88 | 3.15E-86 |
| AC003991.3 | -3.58 | 4.93 | 1.23E-87 | 1.56E-85 |
| CTD-2515H24.2 | -3.55 | 4.06 | 3.34E-87 | 4.16E-85 |
| CASC9 | 7.58 | 10.41 | 2.49E-86 | 3.06E-84 |
| RP13-1016M1.2 | -2.92 | 4.26 | 1.39E-83 | 1.69E-81 |
| RP11-164O23.8 | -3.66 | 3.44 | 3.01E-83 | 3.60E-81 |
| RP4-594A5.1 | 7.17 | 5.70 | 1.95E-81 | 2.27E-79 |
| RBPMS-AS1 | -2.78 | 7.13 | 2.18E-81 | 2.51E-79 |
| PGM5-AS1 | -3.89 | 3.18 | 5.79E-81 | 6.56E-79 |
| MED4-AS1 | -2.32 | 4.26 | 8.16E-81 | 9.12E-79 |
| AP002856.5 | -5.67 | 5.03 | 8.25E-81 | 9.12E-79 |
| RP11-295M18.6 | -2.96 | 3.81 | 3.05E-80 | 3.33E-78 |
| LINC01197 | -2.64 | 6.11 | 7.12E-80 | 7.68E-78 |
| LINC01996 | -4.90 | 5.45 | 8.77E-80 | 9.35E-78 |
| RP1-251M9.3 | -5.02 | 3.25 | 2.11E-79 | 2.22E-77 |
| LINC00511 | 4.09 | 12.12 | 3.52E-79 | 3.66E-77 |
| AC133785.1 | 7.49 | 6.92 | 4.89E-79 | 5.03E-77 |
| SPRY4-IT1 | -3.06 | 4.39 | 8.91E-79 | 9.07E-77 |
| RP11-397A16.1 | 7.60 | 7.55 | 3.84E-78 | 3.87E-76 |
| AC004947.2 | -4.05 | 5.26 | 4.19E-78 | 4.17E-76 |
| RP11-793H13.3 | -2.89 | 3.62 | 1.30E-77 | 1.28E-75 |
| TBX5-AS1 | -2.64 | 9.02 | 5.24E-76 | 5.10E-74 |
| CTB-36H16.2 | -2.10 | 6.86 | 5.53E-76 | 5.33E-74 |
| RP11-335L23.5 | -2.77 | 3.50 | 6.28E-76 | 5.98E-74 |
| RP13-580F15.2 | -2.73 | 5.41 | 7.79E-76 | 7.34E-74 |
| RP11-111E14.1 | -2.83 | 4.13 | 2.11E-75 | 1.97E-73 |
| RP11-2N1.3 | -4.37 | 2.53 | 6.83E-75 | 6.31E-73 |
| LINC01352 | -2.80 | 4.52 | 1.88E-74 | 1.72E-72 |
| LINC01748 | 5.76 | 8.11 | 8.61E-74 | 7.79E-72 |
| RP11-312J18.6 | -4.10 | 3.53 | 1.05E-73 | 9.38E-72 |
| RP11-736K20.5 | -2.52 | 5.28 | 1.76E-73 | 1.56E-71 |
| C5orf64 | -2.85 | 3.17 | 2.82E-73 | 2.47E-71 |
| RP11-4B16.3 | -2.82 | 3.47 | 3.37E-73 | 2.93E-71 |
| RP11-287F9.2 | -4.82 | 3.67 | 5.24E-73 | 4.52E-71 |
| RP11-627G18.1 | -2.96 | 4.12 | 8.82E-73 | 7.53E-71 |
| CTD-2510F5.4 | 3.17 | 8.52 | 1.10E-72 | 9.32E-71 |
| LINC00656 | -3.38 | 4.33 | 2.15E-72 | 1.80E-70 |
| RP11-51B23.3 | -2.70 | 3.64 | 2.59E-72 | 2.13E-70 |
| RP11-95I16.2 | -3.86 | 3.78 | 2.59E-72 | 2.13E-70 |
| TFAP2A-AS1 | 3.37 | 6.77 | 2.09E-71 | 1.70E-69 |
| LINC02163 | 7.42 | 5.93 | 2.13E-71 | 1.72E-69 |
| RP11-359M6.1 | -3.90 | 5.19 | 3.99E-71 | 3.19E-69 |
| RP11-370I10.2 | -3.58 | 2.93 | 4.19E-71 | 3.33E-69 |
| RP11-796E10.1 | 5.69 | 5.73 | 5.99E-71 | 4.70E-69 |
| MIR205HG | 6.17 | 13.39 | 8.71E-71 | 6.79E-69 |
| RP11-325L12.6 | -2.46 | 4.48 | 4.06E-70 | 3.13E-68 |
| AP000438.2 | -3.18 | 3.59 | 5.06E-70 | 3.87E-68 |
| AC105053.3 | -3.64 | 3.50 | 5.44E-70 | 4.13E-68 |
| RP11-476D10.1 | -3.91 | 6.84 | 2.04E-69 | 1.53E-67 |
| RP11-191L9.4 | 8.08 | 6.90 | 3.96E-69 | 2.93E-67 |
| LINC01633 | 6.91 | 5.04 | 4.09E-69 | 3.00E-67 |
| RP13-577H12.2 | -4.57 | 2.86 | 1.30E-68 | 9.38E-67 |
| VIPR1-AS1 | -2.45 | 5.94 | 1.59E-68 | 1.14E-66 |
| PGM5P4-AS1 | -3.34 | 3.31 | 1.97E-68 | 1.40E-66 |
| LINC01863 | -4.59 | 3.83 | 4.37E-68 | 3.08E-66 |
| RP4-564M11.2 | -2.61 | 4.37 | 2.48E-67 | 1.73E-65 |
| TMPO-AS1 | 2.48 | 8.91 | 2.74E-67 | 1.90E-65 |
| RP11-108K3.1 | 5.52 | 5.71 | 2.82E-67 | 1.94E-65 |
| RP11-352D13.5 | -3.06 | 3.57 | 2.96E-67 | 2.02E-65 |
| RP11-354P11.4 | -3.53 | 3.33 | 2.98E-67 | 2.02E-65 |
| RP11-25H12.1 | 7.29 | 5.52 | 3.81E-67 | 2.57E-65 |
| CTC-296K1.4 | -2.78 | 3.49 | 4.21E-67 | 2.81E-65 |
| RP11-108L7.15 | 3.14 | 6.25 | 7.78E-67 | 5.16E-65 |
| POU6F2-AS2 | 7.35 | 6.50 | 8.66E-67 | 5.70E-65 |
| LINC01980 | 8.93 | 8.99 | 1.25E-66 | 8.17E-65 |
| CTD-2562J17.7 | -3.13 | 3.18 | 2.12E-66 | 1.37E-64 |
| LINC00491 | 7.33 | 7.57 | 4.00E-66 | 2.58E-64 |
| MAGI2-AS3 | -2.21 | 10.16 | 4.42E-65 | 2.83E-63 |
| AC005537.2 | 5.19 | 7.70 | 7.84E-65 | 4.98E-63 |
| CTC-296K1.3 | -2.53 | 3.92 | 1.48E-64 | 9.32E-63 |
| RP11-742B18.1 | 5.39 | 8.00 | 2.16E-64 | 1.36E-62 |
| RP11-501J20.5 | -3.00 | 3.21 | 2.60E-64 | 1.62E-62 |
| RP11-174G6.1 | 4.89 | 7.07 | 3.45E-64 | 2.13E-62 |
| C1orf140 | -3.22 | 3.10 | 4.13E-64 | 2.53E-62 |
| RP11-328K4.1 | 7.08 | 6.62 | 5.34E-64 | 3.25E-62 |
| RP11-77A13.1 | -4.88 | 7.05 | 1.12E-63 | 6.74E-62 |
| RP11-161I6.2 | 5.98 | 7.41 | 1.16E-63 | 6.91E-62 |
| CALML3-AS1 | 6.15 | 9.26 | 1.51E-63 | 8.95E-62 |
| RP11-356N1.2 | -2.80 | 4.03 | 2.70E-63 | 1.58E-61 |
| RP11-108K3.2 | 6.56 | 5.36 | 3.06E-63 | 1.78E-61 |
| RP11-624L4.1 | 3.78 | 8.21 | 3.56E-63 | 2.06E-61 |
| DDX11-AS1 | 2.96 | 7.04 | 1.70E-62 | 9.74E-61 |
| PCAT6 | 3.15 | 9.33 | 2.08E-62 | 1.18E-60 |
| CTC-480C2.1 | 7.69 | 5.91 | 2.35E-62 | 1.33E-60 |
| RP3-340B19.3 | -3.55 | 4.92 | 8.66E-62 | 4.88E-60 |
| AC005740.6 | -2.03 | 4.27 | 1.04E-61 | 5.82E-60 |
| RP11-512N21.3 | -3.22 | 3.32 | 2.70E-61 | 1.50E-59 |
| AC002398.12 | -3.30 | 2.80 | 3.52E-61 | 1.94E-59 |
| AC006129.1 | -2.48 | 4.92 | 1.17E-60 | 6.42E-59 |
| RP11-524H19.2 | 6.99 | 7.46 | 1.38E-60 | 7.54E-59 |
| MELTF-AS1 | 3.03 | 9.08 | 2.04E-60 | 1.11E-58 |
| RP11-254F19.2 | -3.23 | 2.69 | 2.42E-60 | 1.30E-58 |
| CTD-2523D13.2 | 4.63 | 7.23 | 2.47E-60 | 1.32E-58 |
| RP5-1159O4.2 | -2.21 | 5.42 | 2.75E-60 | 1.47E-58 |
| VPS9D1-AS1 | 3.76 | 10.18 | 6.29E-60 | 3.27E-58 |
| ADAMTSL4-AS1 | -2.01 | 5.67 | 7.35E-60 | 3.81E-58 |
| MGC27382 | -3.97 | 5.77 | 8.71E-60 | 4.48E-58 |
| STARD13-AS | -2.79 | 3.98 | 1.00E-59 | 5.14E-58 |
| LINC01836 | -2.60 | 6.29 | 1.12E-59 | 5.70E-58 |
| SNHG1 | 2.06 | 12.65 | 2.61E-59 | 1.31E-57 |
| RP3-512B11.3 | 3.28 | 8.47 | 6.31E-59 | 3.16E-57 |
| FOXD3-AS1 | 7.30 | 7.38 | 6.70E-59 | 3.33E-57 |
| RP11-673E11.2 | -3.81 | 2.56 | 6.99E-59 | 3.46E-57 |
| RP11-286E11.1 | -2.39 | 3.53 | 7.39E-59 | 3.64E-57 |
| RP11-1103G16.1 | 8.33 | 7.43 | 9.01E-59 | 4.41E-57 |
| RP11-408O19.5 | -2.24 | 4.29 | 1.38E-58 | 6.74E-57 |
| BCRP3 | -2.31 | 6.35 | 2.45E-58 | 1.18E-56 |
| LINC02038 | -3.07 | 5.65 | 2.93E-58 | 1.40E-56 |
| AP000251.3 | 4.74 | 6.22 | 3.64E-58 | 1.73E-56 |
| CTD-2589M5.4 | -3.81 | 5.04 | 3.97E-58 | 1.88E-56 |
| RP11-750H9.5 | -2.30 | 7.51 | 4.88E-58 | 2.30E-56 |
| RP11-800A3.7 | -2.15 | 5.34 | 5.30E-58 | 2.48E-56 |
| RP11-125O18.1 | -3.29 | 3.84 | 1.55E-57 | 7.22E-56 |
| LINC00163 | -3.68 | 4.18 | 1.60E-57 | 7.44E-56 |
| RP11-66N24.6 | -3.99 | 5.72 | 1.82E-57 | 8.39E-56 |
| CTC-499J9.1 | 6.64 | 7.10 | 2.19E-57 | 1.00E-55 |
| RP11-357D18.1 | -4.14 | 6.25 | 2.19E-57 | 1.00E-55 |
| ZEB2-AS1 | -2.03 | 4.70 | 2.38E-57 | 1.08E-55 |
| AC007405.4 | -2.73 | 5.86 | 4.00E-57 | 1.81E-55 |
| LINC00844 | -3.72 | 2.80 | 4.85E-57 | 2.18E-55 |
| KB-1448A5.1 | -3.11 | 2.80 | 6.42E-57 | 2.86E-55 |
| DLX6-AS1 | 7.06 | 9.51 | 6.48E-57 | 2.87E-55 |
| CADM3-AS1 | -3.24 | 5.64 | 9.04E-57 | 3.99E-55 |
| RP11-27M24.2 | -2.76 | 4.10 | 7.48E-56 | 3.27E-54 |
| RP11-589B3.6 | -2.67 | 2.71 | 1.12E-55 | 4.88E-54 |
| RP11-452C13.1 | -2.65 | 4.28 | 1.13E-55 | 4.89E-54 |
| RP11-532F6.3 | -2.32 | 6.86 | 2.03E-55 | 8.75E-54 |
| AC002066.1 | -2.68 | 5.03 | 2.75E-55 | 1.18E-53 |
| RP11-434H14.1 | -3.06 | 2.63 | 3.10E-55 | 1.32E-53 |
| LINC01985 | -3.33 | 2.77 | 5.06E-55 | 2.15E-53 |
| AF127577.8 | 7.77 | 6.11 | 1.40E-54 | 5.91E-53 |
| Z83851.4 | 2.64 | 8.42 | 1.78E-54 | 7.49E-53 |
| RP11-830F9.6 | -2.35 | 3.39 | 4.25E-54 | 1.78E-52 |
| CTB-193M12.5 | 2.41 | 10.55 | 9.10E-54 | 3.79E-52 |
| AC068831.16 | 4.94 | 5.37 | 9.54E-54 | 3.94E-52 |
| LINC02185 | -3.15 | 5.49 | 1.70E-53 | 6.99E-52 |
| LINC01412 | -2.81 | 2.84 | 4.60E-53 | 1.88E-51 |
| CTD-2373J6.1 | -2.41 | 3.49 | 1.14E-52 | 4.63E-51 |
| RP11-1038A11.3 | 4.79 | 8.40 | 2.25E-52 | 9.12E-51 |
| SFTPD-AS1 | -2.62 | 3.67 | 2.71E-52 | 1.09E-50 |
| RP5-823G15.5 | 3.75 | 5.47 | 3.12E-52 | 1.25E-50 |
| HHIP-AS1 | -2.66 | 7.75 | 5.38E-52 | 2.15E-50 |
| RP11-672A2.5 | -2.76 | 2.71 | 7.15E-52 | 2.85E-50 |
| SLC2A1-AS1 | 3.40 | 7.86 | 8.34E-52 | 3.30E-50 |
| RP11-439L18.1 | -2.25 | 4.00 | 8.50E-52 | 3.35E-50 |
| AC068831.6 | 2.05 | 5.56 | 1.32E-51 | 5.16E-50 |
| RP11-462G12.1 | -2.52 | 5.69 | 1.99E-51 | 7.74E-50 |
| OGFRP1 | 2.66 | 7.31 | 2.16E-51 | 8.37E-50 |
| RP11-244M2.1 | 4.79 | 7.80 | 4.62E-51 | 1.77E-49 |
| RP11-246K15.1 | -3.79 | 3.91 | 5.20E-51 | 1.98E-49 |
| CTD-2313J17.6 | -2.27 | 3.99 | 5.42E-51 | 2.06E-49 |
| RP11-473M20.9 | -2.10 | 6.64 | 6.26E-51 | 2.37E-49 |
| HLX-AS1 | -2.70 | 3.20 | 8.12E-51 | 3.06E-49 |
| RP11-236L14.2 | -2.23 | 4.70 | 9.53E-51 | 3.57E-49 |
| PVT1 | 2.47 | 10.79 | 1.10E-50 | 4.10E-49 |
| HOXC13-AS | 8.04 | 7.40 | 1.27E-50 | 4.72E-49 |
| CTD-2033D15.3 | -2.66 | 4.32 | 1.77E-50 | 6.54E-49 |
| AC092071.1 | -3.74 | 5.13 | 2.22E-50 | 8.14E-49 |
| SNHG4 | 2.65 | 8.78 | 2.63E-50 | 9.60E-49 |
| RP11-650L12.2 | 3.36 | 6.98 | 2.76E-50 | 1.00E-48 |
| RP11-696D21.2 | -2.25 | 3.47 | 4.49E-50 | 1.62E-48 |
| AC009264.1 | 7.85 | 8.06 | 6.04E-50 | 2.17E-48 |
| RP5-1103B4.3 | -4.29 | 2.49 | 7.35E-50 | 2.63E-48 |
| TYMSOS | 3.25 | 7.22 | 1.00E-49 | 3.58E-48 |
| AC135178.7 | -2.91 | 2.80 | 2.37E-49 | 8.42E-48 |
| RP11-322E11.5 | -2.11 | 5.72 | 2.60E-49 | 9.21E-48 |
| RP11-408B11.2 | 6.60 | 6.99 | 2.68E-49 | 9.45E-48 |
| RP11-49G2.3 | -2.50 | 4.03 | 3.03E-49 | 1.06E-47 |
| LINC00519 | 6.04 | 9.03 | 4.69E-49 | 1.64E-47 |
| LINC00982 | -2.62 | 6.60 | 4.86E-49 | 1.69E-47 |
| DARS-AS1 | 2.24 | 8.16 | 6.06E-49 | 2.10E-47 |
| RP11-758M4.4 | 7.87 | 8.86 | 8.65E-49 | 2.97E-47 |
| RP11-484L8.1 | -2.38 | 3.53 | 1.11E-48 | 3.79E-47 |
| RP11-544M22.1 | -3.31 | 5.15 | 1.45E-48 | 4.91E-47 |
| RP11-445O3.2 | 7.58 | 6.20 | 1.53E-48 | 5.16E-47 |
| RP11-346D19.1 | 7.36 | 5.45 | 1.95E-48 | 6.56E-47 |
| RP11-12G12.7 | 2.38 | 9.87 | 2.57E-48 | 8.63E-47 |
| RP11-354E11.2 | -2.40 | 5.76 | 5.34E-48 | 1.78E-46 |
| LINC01105 | -3.84 | 4.69 | 1.10E-47 | 3.66E-46 |
| LINC00472 | -2.18 | 6.18 | 1.52E-47 | 5.05E-46 |
| AC006159.5 | -2.95 | 2.89 | 1.54E-47 | 5.07E-46 |
| RP11-277P12.20 | 4.17 | 10.24 | 1.80E-47 | 5.91E-46 |
| CTD-2527I21.15 | 6.56 | 7.69 | 2.77E-47 | 9.05E-46 |
| AC069277.2 | 5.40 | 6.35 | 3.13E-47 | 1.02E-45 |
| RP11-384F7.2 | -4.01 | 3.95 | 3.66E-47 | 1.19E-45 |
| RP11-89B16.1 | -2.54 | 3.76 | 4.03E-47 | 1.30E-45 |
| LINC00670 | -3.45 | 2.89 | 4.84E-47 | 1.56E-45 |
| RP11-1090M7.1 | -2.78 | 2.75 | 5.79E-47 | 1.86E-45 |
| RP11-789C17.1 | -2.14 | 4.93 | 6.87E-47 | 2.20E-45 |
| LINC00958 | 5.80 | 11.07 | 7.30E-47 | 2.33E-45 |
| CTA-280A3.2 | 6.62 | 5.13 | 1.06E-46 | 3.37E-45 |
| LINC02033 | -2.03 | 3.64 | 1.16E-46 | 3.67E-45 |
| SLC14A2-AS1 | -2.75 | 2.85 | 1.41E-46 | 4.44E-45 |
| AC009262.2 | 5.47 | 3.97 | 1.48E-46 | 4.64E-45 |
| HAGLROS | 4.56 | 8.13 | 3.50E-46 | 1.10E-44 |
| RP11-35J10.7 | -2.77 | 3.67 | 4.44E-46 | 1.39E-44 |
| CTA-363E6.2 | -3.16 | 5.67 | 4.50E-46 | 1.40E-44 |
| RP11-434D9.2 | 5.08 | 4.84 | 8.33E-46 | 2.58E-44 |
| RP11-474N24.6 | -2.13 | 3.93 | 8.88E-46 | 2.74E-44 |
| AC123886.2 | -2.96 | 2.59 | 9.84E-46 | 3.03E-44 |
| RP11-513N24.1 | -2.48 | 5.09 | 1.49E-45 | 4.58E-44 |
| RP11-597M12.2 | -2.47 | 2.74 | 1.91E-45 | 5.83E-44 |
| NPSR1-AS1 | 6.58 | 5.72 | 2.41E-45 | 7.32E-44 |
| RP11-120M18.5 | -2.45 | 4.12 | 2.59E-45 | 7.80E-44 |
| LINC00671 | -2.34 | 3.36 | 2.76E-45 | 8.26E-44 |
| PCAT7 | 4.02 | 7.46 | 3.39E-45 | 1.01E-43 |
| RP11-823E8.3 | -2.24 | 5.81 | 3.49E-45 | 1.04E-43 |
| CTB-66B24.1 | -2.47 | 2.60 | 3.78E-45 | 1.12E-43 |
| RP11-320G24.1 | -2.51 | 4.07 | 3.96E-45 | 1.17E-43 |
| RP4-660H19.1 | 5.72 | 6.61 | 4.04E-45 | 1.19E-43 |
| RP11-35J10.6 | -2.93 | 3.33 | 4.21E-45 | 1.24E-43 |
| SCARNA15 | 2.38 | 6.44 | 5.04E-45 | 1.47E-43 |
| RP5-965F6.2 | -2.28 | 3.51 | 7.36E-45 | 2.14E-43 |
| LINC01096 | 6.24 | 5.69 | 8.51E-45 | 2.47E-43 |
| CASC8 | 4.75 | 7.97 | 1.23E-44 | 3.54E-43 |
| MEF2C-AS1 | -2.22 | 5.04 | 1.51E-44 | 4.35E-43 |
| MIR2052HG | 4.90 | 6.20 | 1.65E-44 | 4.73E-43 |
| RP4-758J18.13 | 2.05 | 8.28 | 1.94E-44 | 5.57E-43 |
| LINC00924 | -2.38 | 5.54 | 4.38E-44 | 1.25E-42 |
| HOXC-AS2 | 5.72 | 6.66 | 5.10E-44 | 1.45E-42 |
| RP11-560J1.2 | 2.65 | 6.35 | 6.77E-44 | 1.92E-42 |
| RP11-16K12.1 | -2.60 | 5.82 | 1.34E-43 | 3.78E-42 |
| DSG1-AS1 | 6.90 | 6.75 | 1.65E-43 | 4.64E-42 |
| RP11-21L23.3 | 3.24 | 6.21 | 2.60E-43 | 7.31E-42 |
| LINC01703 | 3.07 | 7.06 | 4.46E-43 | 1.25E-41 |
| RP11-546J1.1 | 2.54 | 5.42 | 5.06E-43 | 1.41E-41 |
| LINC00668 | 7.16 | 9.91 | 5.83E-43 | 1.62E-41 |
| RP11-783K16.5 | 3.71 | 7.82 | 7.42E-43 | 2.06E-41 |
| RP11-231D20.2 | 4.47 | 6.46 | 7.53E-43 | 2.08E-41 |
| CTD-2184D3.3 | 6.78 | 5.34 | 8.37E-43 | 2.31E-41 |
| RP11-1260E13.2 | -2.90 | 4.31 | 1.50E-42 | 4.09E-41 |
| RP11-499F3.2 | 4.33 | 6.36 | 1.50E-42 | 4.09E-41 |
| LINC01108 | -3.17 | 5.33 | 1.63E-42 | 4.43E-41 |
| RP11-365O16.3 | -2.70 | 4.23 | 1.66E-42 | 4.50E-41 |
| CTD-2340E1.2 | 5.54 | 4.42 | 1.79E-42 | 4.85E-41 |
| RP11-327J17.9 | -2.38 | 3.46 | 2.05E-42 | 5.52E-41 |
| LINC00898 | 7.38 | 6.53 | 2.15E-42 | 5.78E-41 |
| RP11-968A15.2 | 2.50 | 6.10 | 3.46E-42 | 9.29E-41 |
| LINC01967 | 6.25 | 5.18 | 4.44E-42 | 1.19E-40 |
| RP11-108M12.3 | 3.75 | 5.49 | 6.37E-42 | 1.70E-40 |
| RP6-191P20.4 | 5.22 | 5.58 | 8.81E-42 | 2.34E-40 |
| RP11-480A16.1 | 2.82 | 8.48 | 9.02E-42 | 2.39E-40 |
| RP11-213H15.1 | -2.14 | 4.73 | 1.23E-41 | 3.25E-40 |
| RP11-657O9.1 | 5.67 | 7.07 | 1.89E-41 | 4.97E-40 |
| LLNLR-470E3.1 | -2.28 | 5.71 | 3.15E-41 | 8.27E-40 |
| CTD-2008L17.2 | 4.97 | 8.09 | 3.16E-41 | 8.28E-40 |
| RP11-486L19.2 | 4.46 | 6.32 | 3.19E-41 | 8.34E-40 |
| AC079630.2 | -3.45 | 6.02 | 4.82E-41 | 1.25E-39 |
| LL22NC03-104C7.1 | -3.41 | 2.95 | 5.17E-41 | 1.34E-39 |
| RP11-199F11.2 | 2.05 | 7.21 | 6.59E-41 | 1.70E-39 |
| RP11-345J18.2 | -2.23 | 6.21 | 8.64E-41 | 2.22E-39 |
| RP11-445O3.1 | 6.19 | 4.78 | 9.05E-41 | 2.32E-39 |
| RP11-159F24.6 | 4.32 | 7.10 | 9.43E-41 | 2.41E-39 |
| RP11-2N1.2 | -3.85 | 4.26 | 1.04E-40 | 2.66E-39 |
| CTA-989H11.1 | 2.52 | 7.03 | 1.22E-40 | 3.10E-39 |
| RP11-245D16.4 | 2.76 | 6.77 | 1.34E-40 | 3.37E-39 |
| AC011288.2 | 7.03 | 6.96 | 1.47E-40 | 3.71E-39 |
| RP11-157F20.3 | 3.64 | 3.92 | 1.55E-40 | 3.90E-39 |
| RP11-10N16.3 | 4.00 | 4.73 | 1.63E-40 | 4.08E-39 |
| RP5-839B4.8 | -3.47 | 6.70 | 2.00E-40 | 5.00E-39 |
| LINC01165 | -2.67 | 3.14 | 2.32E-40 | 5.76E-39 |
| LINC02104 | -2.42 | 3.84 | 3.05E-40 | 7.54E-39 |
| RP11-44F21.5 | 3.51 | 9.48 | 3.11E-40 | 7.68E-39 |
| CTB-113P19.4 | 5.47 | 8.63 | 3.27E-40 | 8.06E-39 |
| LINC02147 | -2.65 | 3.15 | 3.90E-40 | 9.58E-39 |
| RP1-15D23.2 | -2.53 | 3.10 | 4.57E-40 | 1.12E-38 |
| FIRRE | 3.83 | 7.96 | 4.58E-40 | 1.12E-38 |
| RP11-284N8.3 | -2.28 | 9.04 | 5.62E-40 | 1.37E-38 |
| RP11-779O18.1 | -2.45 | 2.76 | 5.93E-40 | 1.43E-38 |
| KC6 | 5.03 | 8.76 | 6.52E-40 | 1.57E-38 |
| RP11-716O23.2 | -3.28 | 2.55 | 9.58E-40 | 2.29E-38 |
| TMEM108-AS1 | -3.03 | 2.52 | 9.71E-40 | 2.31E-38 |
| RP11-635N19.3 | 5.96 | 4.60 | 1.17E-39 | 2.77E-38 |
| RP11-64B16.4 | -3.16 | 3.21 | 1.60E-39 | 3.77E-38 |
| MYO16-AS1 | -3.07 | 5.57 | 2.07E-39 | 4.86E-38 |
| RP11-114B7.6 | 5.97 | 5.39 | 2.07E-39 | 4.86E-38 |
| RP11-485G7.6 | 3.22 | 5.06 | 3.30E-39 | 7.68E-38 |
| KDM4A-AS1 | 2.76 | 8.17 | 3.37E-39 | 7.82E-38 |
| RP11-738B7.1 | 4.04 | 4.47 | 4.00E-39 | 9.26E-38 |
| RP5-1056H1.2 | 3.16 | 7.48 | 4.14E-39 | 9.56E-38 |
| RP11-227F19.5 | -2.44 | 3.37 | 4.55E-39 | 1.05E-37 |
| HOXA11-AS | 5.92 | 6.96 | 5.29E-39 | 1.21E-37 |
| C1orf132 | -2.01 | 8.52 | 6.76E-39 | 1.54E-37 |
| CTD-2129N1.1 | 3.91 | 3.75 | 7.25E-39 | 1.65E-37 |
| RP1-27K12.4 | 4.49 | 6.91 | 7.83E-39 | 1.78E-37 |
| AC104088.1 | 4.83 | 5.51 | 8.35E-39 | 1.89E-37 |
| CTA-363E6.1 | -2.83 | 2.91 | 9.64E-39 | 2.17E-37 |
| LINC01873 | 4.94 | 4.67 | 9.78E-39 | 2.20E-37 |
| FEZF1-AS1 | 5.96 | 9.50 | 9.84E-39 | 2.21E-37 |
| RP11-594N15.3 | -2.19 | 5.38 | 1.57E-38 | 3.49E-37 |
| CTD-2021H9.3 | 6.46 | 8.12 | 1.72E-38 | 3.82E-37 |
| TRPM2-AS | 4.72 | 8.16 | 1.74E-38 | 3.86E-37 |
| KB-1460A1.1 | 3.03 | 5.76 | 1.88E-38 | 4.15E-37 |
| LINC00551 | -2.62 | 5.31 | 2.06E-38 | 4.53E-37 |
| CYP4A22-AS1 | 3.01 | 5.54 | 2.58E-38 | 5.66E-37 |
| RP11-429J17.7 | 2.54 | 6.51 | 2.91E-38 | 6.37E-37 |
| RP11-540A21.2 | 2.78 | 6.97 | 3.01E-38 | 6.58E-37 |
| CTD-3080P12.3 | -3.88 | 4.07 | 3.13E-38 | 6.82E-37 |
| LINC01807 | 7.26 | 6.70 | 3.85E-38 | 8.35E-37 |
| RP11-99J16__A.2 | -2.27 | 3.49 | 3.91E-38 | 8.46E-37 |
| RP11-7F17.3 | -2.23 | 5.63 | 4.00E-38 | 8.63E-37 |
| CTD-3010D24.3 | 4.79 | 6.69 | 6.63E-38 | 1.42E-36 |
| AC010148.1 | 3.08 | 6.34 | 6.89E-38 | 1.47E-36 |
| DUXAP8 | 3.46 | 9.44 | 7.58E-38 | 1.61E-36 |
| RP4-616B8.5 | 2.74 | 5.57 | 9.32E-38 | 1.97E-36 |
| RP11-519M16.1 | 6.34 | 5.23 | 1.21E-37 | 2.56E-36 |
| RP6-114E22.1 | 5.29 | 6.04 | 1.61E-37 | 3.37E-36 |
| LINC02126 | -2.44 | 4.22 | 1.61E-37 | 3.38E-36 |
| RP11-474G23.3 | 2.52 | 5.58 | 2.16E-37 | 4.51E-36 |
| RP5-1158E12.3 | 3.41 | 5.58 | 2.18E-37 | 4.54E-36 |
| CTD-2531D15.4 | -2.83 | 4.07 | 2.29E-37 | 4.75E-36 |
| AC116366.5 | -2.06 | 3.47 | 2.77E-37 | 5.72E-36 |
| CTD-2165H16.4 | 2.41 | 6.01 | 2.82E-37 | 5.83E-36 |
| LINC01305 | 6.71 | 6.67 | 2.89E-37 | 5.95E-36 |
| CTB-51J22.1 | -2.68 | 6.24 | 2.89E-37 | 5.95E-36 |
| LINC00940 | -2.98 | 2.86 | 3.44E-37 | 7.03E-36 |
| RP11-397O4.1 | -3.26 | 2.44 | 3.91E-37 | 7.98E-36 |
| CTD-2008L17.1 | 4.98 | 7.10 | 3.97E-37 | 8.07E-36 |
| FRMD6-AS1 | 2.41 | 6.64 | 4.96E-37 | 1.00E-35 |
| RP11-1C8.7 | 6.27 | 5.22 | 4.98E-37 | 1.00E-35 |
| RP11-710F7.2 | -2.30 | 3.81 | 5.26E-37 | 1.06E-35 |
| LINC02156 | 3.58 | 3.78 | 5.33E-37 | 1.07E-35 |
| RP11-32K4.1 | 7.18 | 5.89 | 6.09E-37 | 1.22E-35 |
| RP11-932O9.10 | 2.46 | 5.39 | 9.71E-37 | 1.94E-35 |
| RP11-459I19.1 | -2.13 | 4.06 | 9.82E-37 | 1.96E-35 |
| SOX21-AS1 | 4.16 | 9.99 | 1.31E-36 | 2.61E-35 |
| RP11-397A16.3 | 5.75 | 3.89 | 1.57E-36 | 3.11E-35 |
| TARID | -2.60 | 6.43 | 1.67E-36 | 3.30E-35 |
| CTD-2562G15.3 | 2.18 | 4.84 | 1.69E-36 | 3.33E-35 |
| RP11-411K7.1 | 4.89 | 8.58 | 2.67E-36 | 5.27E-35 |
| LINC01977 | 3.87 | 5.83 | 2.96E-36 | 5.79E-35 |
| RP13-463N16.6 | 6.34 | 6.80 | 4.36E-36 | 8.51E-35 |
| RP6-159A1.4 | -2.23 | 7.48 | 4.80E-36 | 9.35E-35 |
| RP11-214N9.1 | 2.10 | 6.17 | 5.64E-36 | 1.09E-34 |
| RP11-120D5.1 | 2.15 | 5.97 | 6.88E-36 | 1.33E-34 |
| LINC01451 | 4.26 | 8.81 | 9.56E-36 | 1.84E-34 |
| RP11-1C8.4 | 5.80 | 5.79 | 1.11E-35 | 2.14E-34 |
| RP11-739B23.1 | 3.09 | 4.58 | 1.12E-35 | 2.15E-34 |
| CTD-2256P15.2 | 2.34 | 8.24 | 1.26E-35 | 2.41E-34 |
| RP11-116N8.4 | -3.98 | 3.34 | 1.32E-35 | 2.53E-34 |
| RP11-96H17.1 | 5.88 | 5.85 | 1.35E-35 | 2.57E-34 |
| RP11-102G14.1 | 2.75 | 5.37 | 1.46E-35 | 2.78E-34 |
| RP11-90L1.8 | 2.97 | 7.42 | 1.47E-35 | 2.78E-34 |
| RP11-390P2.4 | 2.19 | 8.80 | 1.50E-35 | 2.83E-34 |
| LINC01234 | 7.22 | 9.34 | 1.60E-35 | 3.01E-34 |
| RP11-238K6.1 | -3.35 | 6.26 | 1.72E-35 | 3.23E-34 |
| AC002451.3 | -2.56 | 3.52 | 1.73E-35 | 3.25E-34 |
| SFTA3 | -3.60 | 3.64 | 2.25E-35 | 4.21E-34 |
| AC016910.1 | -2.06 | 2.60 | 2.55E-35 | 4.76E-34 |
| LINC01981 | 5.24 | 3.77 | 3.21E-35 | 5.96E-34 |
| RP11-1260E13.3 | -2.65 | 3.84 | 3.26E-35 | 6.04E-34 |
| AC004540.4 | -2.43 | 5.87 | 3.44E-35 | 6.36E-34 |
| AC002076.10 | 3.63 | 4.52 | 4.74E-35 | 8.74E-34 |
| HOXA10-AS | 5.57 | 5.70 | 5.64E-35 | 1.04E-33 |
| RP11-548P2.2 | -2.09 | 4.07 | 6.03E-35 | 1.11E-33 |
| GHET1 | 2.14 | 6.60 | 6.25E-35 | 1.14E-33 |
| RP5-1024N4.4 | -2.03 | 4.17 | 7.21E-35 | 1.32E-33 |
| DEPDC1-AS1 | 3.60 | 3.96 | 8.61E-35 | 1.57E-33 |
| RP11-445O3.3 | 6.51 | 6.10 | 1.08E-34 | 1.96E-33 |
| RP11-863P13.4 | -2.44 | 4.25 | 1.34E-34 | 2.41E-33 |
| RP5-908M14.10 | 2.14 | 6.51 | 1.46E-34 | 2.63E-33 |
| CCAT1 | 8.06 | 10.29 | 1.48E-34 | 2.65E-33 |
| LINC00640 | 3.63 | 7.07 | 1.88E-34 | 3.36E-33 |
| LINC01031 | -2.29 | 3.27 | 2.22E-34 | 3.96E-33 |
| RP11-544I20.2 | 2.00 | 5.41 | 2.39E-34 | 4.26E-33 |
| RP11-108K3.3 | 4.28 | 3.13 | 2.46E-34 | 4.37E-33 |
| PTCSC3 | -2.57 | 4.43 | 2.80E-34 | 4.96E-33 |
| C5orf66-AS1 | 7.82 | 7.45 | 3.50E-34 | 6.18E-33 |
| LINC01698 | 6.76 | 4.73 | 3.56E-34 | 6.26E-33 |
| CTD-2194D22.3 | 7.89 | 5.75 | 3.63E-34 | 6.37E-33 |
| RP11-218E20.3 | 4.75 | 6.70 | 5.55E-34 | 9.70E-33 |
| RP11-546O6.4 | 4.80 | 4.47 | 6.38E-34 | 1.11E-32 |
| RP11-293P20.2 | -3.20 | 3.73 | 7.01E-34 | 1.22E-32 |
| RUNDC3A-AS1 | 3.35 | 7.26 | 7.24E-34 | 1.26E-32 |
| LINC00261 | -3.37 | 8.97 | 7.27E-34 | 1.26E-32 |
| MIR2117HG | 5.55 | 6.05 | 8.49E-34 | 1.47E-32 |
| ZFPM2-AS1 | 5.23 | 8.85 | 9.16E-34 | 1.58E-32 |
| CTC-276P9.4 | 5.59 | 5.17 | 9.42E-34 | 1.62E-32 |
| CTC-537E7.2 | 7.08 | 6.42 | 9.78E-34 | 1.68E-32 |
| AP002954.4 | -2.23 | 4.95 | 1.12E-33 | 1.92E-32 |
| LMO7DN-IT1 | -2.34 | 3.10 | 1.45E-33 | 2.46E-32 |
| RP11-381N20.1 | -2.41 | 2.55 | 1.48E-33 | 2.51E-32 |
| AC114812.8 | 5.64 | 5.13 | 1.54E-33 | 2.59E-32 |
| FAM83A-AS1 | 6.44 | 8.33 | 1.62E-33 | 2.74E-32 |
| GS1-115G20.1 | -2.15 | 2.84 | 1.73E-33 | 2.91E-32 |
| RP11-368L12.1 | 5.58 | 5.95 | 1.99E-33 | 3.34E-32 |
| C8orf34-AS1 | -2.93 | 8.42 | 2.46E-33 | 4.11E-32 |
| RP1-154K9.2 | 4.05 | 5.37 | 2.83E-33 | 4.70E-32 |
| AC008984.2 | -2.15 | 3.50 | 2.97E-33 | 4.93E-32 |
| CTD-2034I4.2 | 3.34 | 4.35 | 3.31E-33 | 5.49E-32 |
| RP11-254F7.4 | 3.43 | 3.87 | 3.87E-33 | 6.41E-32 |
| CTD-2147F2.1 | 6.58 | 7.73 | 4.34E-33 | 7.18E-32 |
| RP6-65G23.3 | 2.51 | 8.22 | 4.79E-33 | 7.89E-32 |
| SLC12A9-AS1 | 2.45 | 5.96 | 5.48E-33 | 9.01E-32 |
| LINC00887 | 4.09 | 6.20 | 5.54E-33 | 9.09E-32 |
| DGUOK-AS1 | 2.00 | 8.41 | 6.28E-33 | 1.03E-31 |
| CTD-3216D2.5 | 2.43 | 5.22 | 7.49E-33 | 1.22E-31 |
| RP11-525G13.2 | 2.19 | 5.93 | 7.71E-33 | 1.26E-31 |
| RP11-481J2.3 | 3.25 | 5.23 | 8.17E-33 | 1.33E-31 |
| CTD-2591A6.2 | 7.18 | 5.45 | 9.35E-33 | 1.52E-31 |
| LINC00211 | -2.61 | 3.45 | 9.68E-33 | 1.57E-31 |
| RP11-286H14.8 | 3.23 | 5.09 | 9.88E-33 | 1.59E-31 |
| LINC01752 | 4.32 | 6.95 | 1.00E-32 | 1.61E-31 |
| RP11-1038A11.1 | 4.09 | 6.07 | 1.15E-32 | 1.86E-31 |
| RP11-286B14.2 | 6.17 | 4.65 | 1.33E-32 | 2.13E-31 |
| AC078942.1 | -2.68 | 2.89 | 1.56E-32 | 2.49E-31 |
| RP11-432I5.2 | 3.27 | 4.01 | 1.63E-32 | 2.60E-31 |
| RP11-159F24.5 | 3.38 | 6.11 | 2.67E-32 | 4.22E-31 |
| SMAD9-IT1 | -2.08 | 3.08 | 3.14E-32 | 4.96E-31 |
| RP13-455A7.1 | 4.76 | 3.45 | 3.32E-32 | 5.23E-31 |
| AC114803.3 | 5.36 | 3.83 | 3.55E-32 | 5.59E-31 |
| RP11-83M16.6 | 4.76 | 5.84 | 4.45E-32 | 6.97E-31 |
| RP11-367G18.1 | 3.36 | 8.95 | 4.96E-32 | 7.74E-31 |
| RP11-672A2.6 | -2.22 | 2.65 | 5.11E-32 | 7.95E-31 |
| RP11-452I5.2 | 2.67 | 8.00 | 5.60E-32 | 8.67E-31 |
| RP11-57A1.1 | 3.15 | 4.00 | 5.86E-32 | 9.06E-31 |
| LINC01624 | -2.29 | 3.97 | 7.46E-32 | 1.15E-30 |
| RP11-486G15.2 | 2.15 | 6.21 | 7.98E-32 | 1.23E-30 |
| AC195454.1 | -2.40 | 4.10 | 1.28E-31 | 1.96E-30 |
| RP11-646E18.4 | 6.51 | 6.19 | 1.34E-31 | 2.05E-30 |
| RP4-669H2.1 | 3.09 | 5.76 | 1.93E-31 | 2.94E-30 |
| LINC01605 | 4.50 | 8.78 | 2.28E-31 | 3.44E-30 |
| LINC01331 | -3.14 | 2.56 | 2.34E-31 | 3.53E-30 |
| RP3-323A16.1 | 4.02 | 8.38 | 2.41E-31 | 3.62E-30 |
| AC007743.1 | -2.14 | 7.48 | 2.74E-31 | 4.11E-30 |
| RP11-671P2.1 | -2.39 | 3.32 | 2.84E-31 | 4.26E-30 |
| CASC19 | 6.65 | 7.18 | 2.90E-31 | 4.34E-30 |
| RP11-535A5.1 | -2.10 | 3.01 | 3.42E-31 | 5.10E-30 |
| RP11-701H24.3 | -2.01 | 3.77 | 4.03E-31 | 5.97E-30 |
| CTD-2134A5.4 | 2.07 | 7.23 | 4.32E-31 | 6.39E-30 |
| RP5-836N17.4 | 3.15 | 4.40 | 4.35E-31 | 6.41E-30 |
| AC069513.4 | 3.33 | 5.08 | 4.91E-31 | 7.20E-30 |
| RP11-21L23.2 | 2.67 | 9.19 | 5.18E-31 | 7.57E-30 |
| PGM5P3-AS1 | -2.97 | 3.56 | 6.41E-31 | 9.34E-30 |
| AC013264.2 | -2.79 | 6.00 | 9.21E-31 | 1.34E-29 |
| RP11-63N8.3 | -2.51 | 2.46 | 1.01E-30 | 1.47E-29 |
| CTB-61M7.2 | -2.12 | 5.84 | 1.34E-30 | 1.95E-29 |
| RMST | -3.39 | 4.50 | 1.35E-30 | 1.95E-29 |
| CTD-2227E11.1 | 2.39 | 6.34 | 1.61E-30 | 2.32E-29 |
| RP11-544L8__B.4 | -2.40 | 3.97 | 1.67E-30 | 2.41E-29 |
| RP11-881M11.4 | 3.21 | 4.76 | 1.68E-30 | 2.43E-29 |
| C2orf48 | 2.89 | 6.45 | 1.73E-30 | 2.49E-29 |
| LINC00626 | 7.72 | 7.99 | 1.81E-30 | 2.60E-29 |
| HOTTIP | 5.96 | 4.71 | 1.96E-30 | 2.80E-29 |
| RP4-694A7.2 | 4.91 | 4.89 | 2.17E-30 | 3.09E-29 |
| LINC01063 | 2.69 | 6.00 | 2.34E-30 | 3.33E-29 |
| RP11-483F11.7 | 2.83 | 5.47 | 2.36E-30 | 3.35E-29 |
| BARX1-AS1 | 7.09 | 5.37 | 2.52E-30 | 3.57E-29 |
| AP000679.2 | 3.44 | 4.11 | 2.76E-30 | 3.90E-29 |
| LSAMP-AS1 | 4.86 | 5.51 | 2.83E-30 | 3.99E-29 |
| LINC02043 | 4.55 | 5.65 | 2.83E-30 | 3.99E-29 |
| RP11-1100L3.8 | -2.09 | 5.92 | 2.86E-30 | 4.03E-29 |
| RP11-429B14.4 | 5.18 | 4.11 | 3.15E-30 | 4.42E-29 |
| AC011738.4 | 4.72 | 5.67 | 3.23E-30 | 4.53E-29 |
| CTD-2626G11.2 | -2.81 | 7.26 | 4.24E-30 | 5.93E-29 |
| RP11-1M18.1 | 3.14 | 4.86 | 4.25E-30 | 5.93E-29 |
| RP1-272L16.1 | 6.36 | 5.88 | 4.57E-30 | 6.36E-29 |
| RP11-634H22.1 | 2.21 | 5.87 | 5.21E-30 | 7.21E-29 |
| CTD-2256P15.1 | 3.02 | 3.46 | 6.00E-30 | 8.29E-29 |
| RP11-390F4.3 | 3.05 | 8.14 | 6.28E-30 | 8.66E-29 |
| ATP2A1-AS1 | 2.33 | 6.40 | 7.31E-30 | 1.01E-28 |
| RP11-203F10.5 | -2.71 | 2.81 | 8.10E-30 | 1.11E-28 |
| RP11-98G7.1 | 3.86 | 5.32 | 1.74E-29 | 2.36E-28 |
| CTD-2532K18.2 | 4.69 | 4.99 | 1.88E-29 | 2.55E-28 |
| LINC00355 | 7.70 | 6.62 | 2.06E-29 | 2.79E-28 |
| CTA-363E6.6 | -2.26 | 4.01 | 2.12E-29 | 2.87E-28 |
| RP11-15K2.2 | -2.32 | 2.57 | 2.40E-29 | 3.25E-28 |
| LINC01468 | 7.47 | 7.06 | 2.54E-29 | 3.43E-28 |
| UMODL1-AS1 | -3.01 | 5.48 | 2.75E-29 | 3.70E-28 |
| RP11-394I13.2 | 2.13 | 5.72 | 4.62E-29 | 6.19E-28 |
| NAV2-AS2 | -2.51 | 4.04 | 5.74E-29 | 7.64E-28 |
| RP11-499F3.1 | 3.64 | 5.01 | 6.24E-29 | 8.28E-28 |
| RP11-276H19.2 | 4.30 | 9.50 | 6.52E-29 | 8.64E-28 |
| RP11-542G1.1 | 5.86 | 5.21 | 6.59E-29 | 8.72E-28 |
| AC013275.2 | -2.57 | 5.12 | 6.60E-29 | 8.72E-28 |
| RP11-9E17.1 | 2.37 | 9.58 | 8.37E-29 | 1.10E-27 |
| FAM83C-AS1 | 2.71 | 4.02 | 8.54E-29 | 1.12E-27 |
| RP11-351I24.1 | 2.71 | 5.41 | 1.04E-28 | 1.37E-27 |
| RP3-369A17.4 | -2.05 | 3.30 | 1.08E-28 | 1.42E-27 |
| LINC00892 | -2.08 | 5.39 | 1.24E-28 | 1.63E-27 |
| MIR9-3HG | 3.34 | 9.65 | 1.25E-28 | 1.63E-27 |
| HOXC-AS1 | 4.10 | 5.68 | 1.38E-28 | 1.79E-27 |
| AC015849.16 | 3.60 | 6.03 | 1.57E-28 | 2.05E-27 |
| LSINCT5 | -2.77 | 3.09 | 1.60E-28 | 2.07E-27 |
| RP11-527N22.2 | 4.73 | 6.53 | 1.62E-28 | 2.10E-27 |
| SATB2-AS1 | 3.44 | 4.97 | 1.63E-28 | 2.11E-27 |
| LEF1-AS1 | 2.15 | 5.85 | 1.77E-28 | 2.29E-27 |
| CTD-2292P10.4 | 2.74 | 6.87 | 2.01E-28 | 2.60E-27 |
| HHATL-AS1 | -3.02 | 3.45 | 2.15E-28 | 2.77E-27 |
| MYOSLID | 3.39 | 7.99 | 2.37E-28 | 3.05E-27 |
| AC004221.2 | 3.62 | 3.91 | 2.45E-28 | 3.15E-27 |
| RP11-909N17.2 | 5.05 | 5.90 | 2.46E-28 | 3.15E-27 |
| LINC01564 | 4.17 | 7.59 | 2.48E-28 | 3.18E-27 |
| TTC39A-AS1 | -2.10 | 4.92 | 3.03E-28 | 3.86E-27 |
| RP11-308D13.3 | 5.24 | 4.71 | 3.24E-28 | 4.13E-27 |
| RP11-295G20.2 | 2.76 | 10.06 | 3.52E-28 | 4.48E-27 |
| RP11-122M14.1 | -2.40 | 5.02 | 4.07E-28 | 5.15E-27 |
| CTC-575I10.1 | 3.70 | 4.04 | 4.87E-28 | 6.15E-27 |
| NAALADL2-AS2 | 5.99 | 6.24 | 5.12E-28 | 6.45E-27 |
| LINC01711 | 3.57 | 5.06 | 5.17E-28 | 6.50E-27 |
| LL22NC03-N64E9.1 | 4.45 | 4.47 | 5.97E-28 | 7.50E-27 |
| RP5-1063M23.2 | 3.20 | 5.29 | 6.30E-28 | 7.91E-27 |
| AC003958.2 | 6.31 | 5.68 | 6.61E-28 | 8.27E-27 |
| LINC01208 | 5.29 | 4.20 | 6.63E-28 | 8.29E-27 |
| RP4-539M6.14 | -2.87 | 5.69 | 6.84E-28 | 8.53E-27 |
| ABCA9-AS1 | 4.56 | 6.41 | 7.51E-28 | 9.35E-27 |
| RP11-319E16.1 | 4.04 | 3.44 | 9.15E-28 | 1.13E-26 |
| C10orf91 | 3.19 | 7.46 | 1.10E-27 | 1.36E-26 |
| CTC-251D13.1 | 2.32 | 7.13 | 1.39E-27 | 1.71E-26 |
| RP11-336A10.5 | 4.62 | 5.31 | 1.62E-27 | 1.99E-26 |
| RP11-314A20.2 | 2.60 | 4.87 | 1.66E-27 | 2.02E-26 |
| CTD-2228K2.7 | 2.78 | 11.09 | 1.85E-27 | 2.26E-26 |
| RP11-627G18.2 | -2.24 | 2.80 | 2.45E-27 | 2.98E-26 |
| LINC01012 | 2.11 | 6.44 | 2.53E-27 | 3.07E-26 |
| CTC-321K16.1 | 4.72 | 5.15 | 2.67E-27 | 3.24E-26 |
| RP11-567N4.3 | 5.97 | 5.01 | 2.83E-27 | 3.42E-26 |
| RP11-74M11.2 | -3.26 | 2.53 | 3.07E-27 | 3.71E-26 |
| LINC00840 | -2.48 | 3.63 | 3.13E-27 | 3.77E-26 |
| LINC00858 | 4.45 | 5.79 | 3.47E-27 | 4.17E-26 |
| LINC00592 | 3.06 | 5.62 | 4.55E-27 | 5.45E-26 |
| SOX2-OT | 5.51 | 11.27 | 4.71E-27 | 5.63E-26 |
| RP11-432J24.2 | -2.04 | 3.25 | 5.13E-27 | 6.11E-26 |
| AC114765.1 | 3.69 | 3.33 | 5.25E-27 | 6.25E-26 |
| CTD-2134A5.3 | 2.07 | 5.42 | 5.33E-27 | 6.34E-26 |
| RP11-95M15.1 | 5.38 | 5.97 | 6.41E-27 | 7.58E-26 |
| RP11-1007I13.4 | 5.44 | 4.67 | 6.43E-27 | 7.60E-26 |
| LINC01968 | 3.49 | 4.12 | 7.62E-27 | 8.99E-26 |
| RP11-357H14.17 | 5.49 | 8.01 | 8.29E-27 | 9.76E-26 |
| CTD-2139B15.5 | 7.28 | 5.19 | 8.54E-27 | 1.00E-25 |
| LINC00928 | 4.75 | 3.32 | 8.56E-27 | 1.01E-25 |
| XXbac-B444P24.8 | -2.25 | 3.68 | 8.96E-27 | 1.05E-25 |
| RP11-3B12.5 | 5.78 | 5.77 | 1.07E-26 | 1.25E-25 |
| RP5-968D22.3 | 2.95 | 3.54 | 1.16E-26 | 1.35E-25 |
| RP1-170O19.14 | 4.77 | 3.40 | 1.30E-26 | 1.51E-25 |
| RP11-132A1.4 | 3.11 | 8.49 | 1.31E-26 | 1.52E-25 |
| LINC01249 | 8.32 | 7.13 | 1.48E-26 | 1.71E-25 |
| LINC01385 | 5.54 | 4.13 | 1.49E-26 | 1.71E-25 |
| AC005753.1 | -2.07 | 3.27 | 1.56E-26 | 1.80E-25 |
| DLX2-AS1 | 5.51 | 3.99 | 1.78E-26 | 2.04E-25 |
| RP11-529G21.2 | 2.51 | 4.20 | 1.91E-26 | 2.20E-25 |
| ARNTL2-AS1 | 3.91 | 3.88 | 1.96E-26 | 2.25E-25 |
| SATB1-AS1 | 2.33 | 7.85 | 2.39E-26 | 2.72E-25 |
| RP11-395G23.3 | 2.45 | 8.63 | 2.73E-26 | 3.10E-25 |
| LL22NC03-63E9.3 | 4.60 | 4.87 | 2.75E-26 | 3.11E-25 |
| RP11-366F6.2 | 8.07 | 7.70 | 2.83E-26 | 3.21E-25 |
| FSIP2-AS1 | 2.87 | 4.97 | 2.96E-26 | 3.34E-25 |
| RP11-230B22.1 | 2.44 | 4.71 | 2.96E-26 | 3.34E-25 |
| RP11-394B2.6 | -2.06 | 3.01 | 3.23E-26 | 3.63E-25 |
| LINC01206 | 9.46 | 11.80 | 3.31E-26 | 3.71E-25 |
| RP11-387H17.6 | -2.38 | 3.14 | 3.76E-26 | 4.21E-25 |
| AC006262.6 | 3.57 | 7.29 | 3.81E-26 | 4.24E-25 |
| LINC01524 | 4.72 | 4.83 | 3.81E-26 | 4.24E-25 |
| RP11-366L20.2 | 2.69 | 6.42 | 4.85E-26 | 5.39E-25 |
| CTC-327F10.4 | 4.91 | 4.21 | 5.31E-26 | 5.88E-25 |
| RP1-111C20.3 | -2.11 | 3.38 | 5.69E-26 | 6.29E-25 |
| RP11-680H20.2 | 4.69 | 6.44 | 5.95E-26 | 6.58E-25 |
| CTD-2033A16.3 | 3.11 | 5.32 | 6.23E-26 | 6.88E-25 |
| CTC-558O2.2 | -2.14 | 2.98 | 6.28E-26 | 6.92E-25 |
| RP11-96B2.1 | 4.70 | 4.81 | 6.77E-26 | 7.44E-25 |
| RP11-574O7.1 | -2.32 | 2.56 | 7.92E-26 | 8.67E-25 |
| SRGAP3-AS2 | -3.29 | 7.49 | 7.92E-26 | 8.67E-25 |
| MAFA-AS1 | 4.96 | 5.48 | 8.17E-26 | 8.93E-25 |
| CTD-2330J20.2 | -2.65 | 2.51 | 1.34E-25 | 1.46E-24 |
| RP11-148B3.1 | 6.12 | 4.50 | 1.39E-25 | 1.51E-24 |
| LINC01765 | -3.22 | 4.63 | 1.92E-25 | 2.07E-24 |
| RP11-254F7.1 | 5.95 | 4.94 | 1.94E-25 | 2.09E-24 |
| HOXD-AS2 | 3.81 | 6.22 | 2.42E-25 | 2.59E-24 |
| LINC01099 | -2.03 | 3.16 | 2.47E-25 | 2.65E-24 |
| LINC00393 | 7.82 | 6.51 | 2.55E-25 | 2.72E-24 |
| LINC01518 | 8.23 | 6.24 | 3.01E-25 | 3.21E-24 |
| RP11-133K1.11 | 3.53 | 3.49 | 3.17E-25 | 3.38E-24 |
| RP11-493L12.5 | 2.65 | 4.40 | 3.19E-25 | 3.38E-24 |
| CTD-3035D6.2 | 2.95 | 5.17 | 3.19E-25 | 3.38E-24 |
| LINC00619 | -2.23 | 3.13 | 3.38E-25 | 3.58E-24 |
| RP11-356K23.2 | -2.46 | 2.91 | 3.60E-25 | 3.80E-24 |
| CTD-2309O5.3 | 5.03 | 4.16 | 3.64E-25 | 3.85E-24 |
| RP11-739L10.1 | 2.33 | 5.46 | 3.70E-25 | 3.90E-24 |
| LINC01876 | 2.38 | 7.09 | 3.82E-25 | 4.02E-24 |
| LINC01506 | -2.21 | 4.27 | 4.38E-25 | 4.60E-24 |
| RP5-1097P24.1 | 7.31 | 6.39 | 4.82E-25 | 5.04E-24 |
| SH3PXD2A-AS1 | 3.91 | 8.96 | 5.20E-25 | 5.43E-24 |
| LINC01625 | -2.21 | 3.14 | 6.22E-25 | 6.47E-24 |
| RP11-13E5.2 | 4.99 | 3.68 | 6.75E-25 | 7.02E-24 |
| RP11-408E5.5 | 6.22 | 5.02 | 7.98E-25 | 8.26E-24 |
| LINC02012 | 3.13 | 7.20 | 8.68E-25 | 8.96E-24 |
| RP11-568J23.8 | 2.92 | 5.59 | 1.09E-24 | 1.12E-23 |
| RP11-96H17.3 | 4.51 | 4.16 | 1.14E-24 | 1.17E-23 |
| LINC02187 | 5.60 | 5.62 | 1.28E-24 | 1.31E-23 |
| RP11-496D24.2 | 4.76 | 3.97 | 1.32E-24 | 1.35E-23 |
| RP11-390N6.1 | 3.34 | 3.72 | 1.33E-24 | 1.36E-23 |
| RP11-470P21.2 | 4.27 | 4.26 | 1.54E-24 | 1.57E-23 |
| RP11-778D9.12 | 2.06 | 5.76 | 1.59E-24 | 1.62E-23 |
| GACAT2 | 4.28 | 4.28 | 1.60E-24 | 1.63E-23 |
| RP11-255G12.2 | 2.98 | 4.39 | 1.61E-24 | 1.64E-23 |
| PKP4-AS1 | 3.00 | 7.71 | 1.66E-24 | 1.69E-23 |
| CTD-2650P22.2 | 2.10 | 6.08 | 1.76E-24 | 1.78E-23 |
| LINC00885 | 2.81 | 9.44 | 1.91E-24 | 1.94E-23 |
| RP11-713C5.1 | 3.88 | 5.92 | 2.13E-24 | 2.15E-23 |
| LINC01456 | 6.30 | 5.76 | 2.31E-24 | 2.32E-23 |
| CTD-3224I3.3 | -2.10 | 2.83 | 2.41E-24 | 2.43E-23 |
| LINC02014 | 2.74 | 6.19 | 2.45E-24 | 2.46E-23 |
| CTB-186G2.1 | 5.10 | 5.95 | 2.63E-24 | 2.64E-23 |
| RP11-353N14.1 | 4.34 | 3.80 | 2.64E-24 | 2.64E-23 |
| RP11-335K5.2 | 4.20 | 3.43 | 2.73E-24 | 2.74E-23 |
| RP11-277B15.3 | 2.19 | 4.88 | 2.77E-24 | 2.76E-23 |
| RP11-157I4.4 | 4.43 | 3.53 | 3.25E-24 | 3.25E-23 |
| RP11-80H5.2 | 3.85 | 3.66 | 3.28E-24 | 3.27E-23 |
| RP11-44B19.1 | -2.16 | 3.23 | 3.55E-24 | 3.53E-23 |
| CTD-2562J15.6 | -2.42 | 2.67 | 3.98E-24 | 3.94E-23 |
| RP3-332B22.1 | -3.12 | 3.03 | 4.64E-24 | 4.58E-23 |
| HOTAIR | 6.62 | 7.10 | 4.70E-24 | 4.63E-23 |
| AC011899.10 | -3.11 | 3.41 | 5.85E-24 | 5.75E-23 |
| CTD-2555C10.3 | 3.26 | 6.37 | 6.16E-24 | 6.05E-23 |
| HOXC-AS3 | 6.51 | 5.51 | 6.53E-24 | 6.40E-23 |
| RP11-84D1.1 | 2.25 | 4.90 | 6.57E-24 | 6.44E-23 |
| RP11-677M24.1 | 5.12 | 3.87 | 6.73E-24 | 6.58E-23 |
| PARD3-AS1 | 2.46 | 5.42 | 7.31E-24 | 7.13E-23 |
| RP11-789C1.2 | -3.03 | 2.69 | 8.83E-24 | 8.58E-23 |
| RP11-549B18.1 | 2.11 | 7.09 | 9.72E-24 | 9.40E-23 |
| RP11-424M24.5 | -3.21 | 4.11 | 1.04E-23 | 1.00E-22 |
| LINC02109 | 5.79 | 5.76 | 1.07E-23 | 1.03E-22 |
| RP11-573D15.8 | 3.11 | 5.53 | 1.08E-23 | 1.04E-22 |
| RP11-476M19.3 | -2.80 | 2.53 | 1.08E-23 | 1.04E-22 |
| RP11-104H15.10 | 3.16 | 3.32 | 1.12E-23 | 1.07E-22 |
| RP11-277P12.9 | 3.63 | 4.91 | 1.53E-23 | 1.46E-22 |
| LINC01905 | 3.75 | 5.14 | 1.67E-23 | 1.59E-22 |
| RP11-197K6.1 | 7.83 | 7.86 | 1.67E-23 | 1.59E-22 |
| LINC00930 | -2.50 | 5.12 | 1.72E-23 | 1.63E-22 |
| AC012501.2 | 6.02 | 4.62 | 1.74E-23 | 1.66E-22 |
| LINC00941 | 3.83 | 7.93 | 1.86E-23 | 1.76E-22 |
| RP11-259O2.2 | 5.17 | 4.46 | 1.94E-23 | 1.83E-22 |
| RP11-510J16.5 | 2.76 | 6.91 | 1.99E-23 | 1.88E-22 |
| LINC01561 | 3.88 | 5.12 | 2.06E-23 | 1.94E-22 |
| RP4-616B8.6 | 3.09 | 3.64 | 2.08E-23 | 1.96E-22 |
| RP1-163G9.2 | -2.85 | 3.59 | 2.10E-23 | 1.98E-22 |
| RP11-386M24.3 | -2.38 | 4.37 | 2.11E-23 | 1.98E-22 |
| LINC00337 | 2.64 | 5.91 | 2.22E-23 | 2.08E-22 |
| XXbac-BPG13B8.10 | -2.24 | 3.66 | 2.35E-23 | 2.20E-22 |
| RP11-503N18.4 | -2.53 | 3.66 | 2.47E-23 | 2.31E-22 |
| AC007278.2 | -2.27 | 2.72 | 2.50E-23 | 2.34E-22 |
| RP11-314B1.2 | -2.41 | 6.75 | 3.13E-23 | 2.91E-22 |
| RP11-723O4.9 | 2.52 | 6.63 | 3.15E-23 | 2.93E-22 |
| RP11-123K3.9 | 2.64 | 3.50 | 3.22E-23 | 2.99E-22 |
| LINC01322 | 4.60 | 5.56 | 3.27E-23 | 3.03E-22 |
| RP11-300J18.1 | -2.26 | 2.50 | 3.61E-23 | 3.34E-22 |
| RP11-310P5.1 | 3.36 | 3.39 | 4.01E-23 | 3.70E-22 |
| RP11-379B8.1 | 2.43 | 8.30 | 4.05E-23 | 3.73E-22 |
| AF131216.5 | 5.27 | 6.00 | 4.08E-23 | 3.76E-22 |
| RP11-190C22.8 | 2.27 | 4.18 | 4.26E-23 | 3.92E-22 |
| RP1-244F24.1 | 2.01 | 5.77 | 5.21E-23 | 4.77E-22 |
| RP11-380J14.1 | 5.36 | 6.30 | 5.24E-23 | 4.79E-22 |
| CTC-441N14.1 | -2.28 | 3.81 | 5.52E-23 | 5.04E-22 |
| U47924.29 | 2.46 | 3.75 | 5.73E-23 | 5.22E-22 |
| RP11-220C2.1 | 3.02 | 3.93 | 5.74E-23 | 5.23E-22 |
| CYP4F26P | 3.45 | 6.68 | 5.83E-23 | 5.31E-22 |
| RP11-423H2.3 | 2.34 | 6.66 | 5.95E-23 | 5.41E-22 |
| RP11-145A3.1 | 3.22 | 6.59 | 6.14E-23 | 5.58E-22 |
| CTD-2245E15.3 | -2.38 | 5.96 | 6.31E-23 | 5.73E-22 |
| RP5-984P4.6 | 6.35 | 5.60 | 6.37E-23 | 5.77E-22 |
| RP11-98D18.15 | -2.54 | 2.77 | 6.50E-23 | 5.89E-22 |
| RP11-465B22.8 | 2.20 | 8.23 | 6.78E-23 | 6.13E-22 |
| RP11-78F17.1 | 4.16 | 5.02 | 7.11E-23 | 6.41E-22 |
| LINC01559 | 5.97 | 8.50 | 7.21E-23 | 6.50E-22 |
| LINC01827 | -2.76 | 3.48 | 8.70E-23 | 7.80E-22 |
| LINC01705 | 3.72 | 5.83 | 8.98E-23 | 8.05E-22 |
| RP11-816J6.3 | 2.47 | 5.72 | 9.34E-23 | 8.35E-22 |
| RP11-884K10.6 | -2.16 | 2.76 | 9.84E-23 | 8.79E-22 |
| RP11-146I2.1 | 2.96 | 4.35 | 9.86E-23 | 8.80E-22 |
| RP11-963H4.3 | -2.00 | 2.90 | 1.04E-22 | 9.27E-22 |
| RP11-285C1.2 | 4.53 | 4.05 | 1.16E-22 | 1.04E-21 |
| LINC02076 | 2.93 | 4.51 | 1.22E-22 | 1.08E-21 |
| LINC01844 | -2.11 | 3.68 | 1.22E-22 | 1.08E-21 |
| AC012531.25 | 3.65 | 5.41 | 1.28E-22 | 1.14E-21 |
| RP11-337N6.1 | 5.12 | 7.49 | 1.31E-22 | 1.16E-21 |
| AC009501.4 | 2.52 | 7.92 | 1.34E-22 | 1.19E-21 |
| AC107072.2 | -2.26 | 2.74 | 1.45E-22 | 1.28E-21 |
| KCNQ5-IT1 | 4.25 | 3.56 | 1.47E-22 | 1.29E-21 |
| RP11-114G22.1 | 5.78 | 6.08 | 1.49E-22 | 1.31E-21 |
| LINC01833 | 6.93 | 6.98 | 1.60E-22 | 1.41E-21 |
| LINC01901 | 4.90 | 6.17 | 1.62E-22 | 1.42E-21 |
| MNX1-AS1 | 4.87 | 7.03 | 1.73E-22 | 1.52E-21 |
| LINC00942 | 7.15 | 10.70 | 1.99E-22 | 1.74E-21 |
| RP4-781K5.5 | 5.45 | 4.37 | 2.14E-22 | 1.87E-21 |
| RP1-101G11.3 | 6.10 | 5.43 | 2.18E-22 | 1.90E-21 |
| RP11-467P9.1 | 2.39 | 4.49 | 2.30E-22 | 2.00E-21 |
| LINC01133 | 4.30 | 10.86 | 2.40E-22 | 2.08E-21 |
| RP1-27K12.2 | 7.85 | 11.52 | 2.47E-22 | 2.14E-21 |
| RP11-70F11.7 | 6.55 | 5.03 | 2.51E-22 | 2.18E-21 |
| RP11-499O7.7 | 3.93 | 6.57 | 2.54E-22 | 2.20E-21 |
| LINC01611 | 6.22 | 5.00 | 2.68E-22 | 2.31E-21 |
| LINC01612 | -2.69 | 4.94 | 2.84E-22 | 2.45E-21 |
| LINC01393 | 2.26 | 5.40 | 2.85E-22 | 2.45E-21 |
| LINC01971 | 3.42 | 3.93 | 2.85E-22 | 2.45E-21 |
| RP11-177F15.1 | 5.02 | 5.06 | 2.87E-22 | 2.46E-21 |
| RP11-6O2.2 | 2.74 | 4.83 | 2.87E-22 | 2.47E-21 |
| BLACAT1 | 2.84 | 8.87 | 2.92E-22 | 2.51E-21 |
| RP11-539E17.5 | 4.87 | 4.06 | 3.09E-22 | 2.65E-21 |
| IGFL2-AS1 | 5.38 | 9.27 | 3.19E-22 | 2.72E-21 |
| AC108676.1 | 4.96 | 8.39 | 3.28E-22 | 2.79E-21 |
| C20orf166-AS1 | -2.05 | 3.77 | 3.44E-22 | 2.93E-21 |
| RP11-65D17.1 | 4.75 | 4.83 | 3.45E-22 | 2.94E-21 |
| RP11-383I23.2 | 2.05 | 5.25 | 3.67E-22 | 3.12E-21 |
| RP11-417L19.2 | 4.04 | 4.10 | 3.84E-22 | 3.26E-21 |
| LINC01615 | 3.54 | 7.21 | 3.85E-22 | 3.27E-21 |
| RP11-44N22.3 | 2.23 | 4.94 | 3.89E-22 | 3.29E-21 |
| RP4-785G19.5 | 2.36 | 4.79 | 3.90E-22 | 3.30E-21 |
| RP11-685G9.4 | 3.73 | 3.19 | 4.25E-22 | 3.59E-21 |
| LINC01775 | 2.51 | 3.82 | 4.54E-22 | 3.83E-21 |
| CASC20 | 5.17 | 6.29 | 4.57E-22 | 3.84E-21 |
| RP11-536G4.2 | -2.49 | 4.05 | 4.59E-22 | 3.86E-21 |
| LINC01280 | 4.89 | 3.47 | 5.52E-22 | 4.63E-21 |
| LL22NC03-N14H11.1 | 2.64 | 5.30 | 5.67E-22 | 4.75E-21 |
| RP11-553A10.1 | 4.05 | 7.65 | 5.75E-22 | 4.80E-21 |
| CTD-2308L22.1 | 2.01 | 6.87 | 5.93E-22 | 4.96E-21 |
| SMILR | 3.93 | 5.03 | 6.04E-22 | 5.04E-21 |
| PART1 | 4.58 | 9.01 | 6.11E-22 | 5.10E-21 |
| AC005256.1 | 5.62 | 4.29 | 6.50E-22 | 5.40E-21 |
| AC097713.3 | 6.32 | 6.27 | 6.76E-22 | 5.61E-21 |
| RP11-120K18.2 | 2.87 | 4.87 | 7.21E-22 | 5.98E-21 |
| RP11-193M21.1 | -2.44 | 4.73 | 7.24E-22 | 5.99E-21 |
| AC073321.4 | 4.06 | 3.41 | 7.48E-22 | 6.19E-21 |
| RP11-54H7.4 | 5.46 | 10.93 | 7.68E-22 | 6.33E-21 |
| LINC00871 | 5.51 | 5.86 | 8.24E-22 | 6.77E-21 |
| RP11-964E11.2 | 3.57 | 7.08 | 8.50E-22 | 6.97E-21 |
| CTB-50L17.5 | 2.80 | 3.52 | 8.90E-22 | 7.28E-21 |
| AC010731.2 | 4.67 | 5.51 | 1.12E-21 | 9.17E-21 |
| LA16c-329F2.2 | 2.06 | 4.42 | 1.16E-21 | 9.45E-21 |
| LINC02003 | 3.59 | 3.58 | 1.35E-21 | 1.10E-20 |
| RP11-107M16.2 | 4.10 | 4.41 | 1.49E-21 | 1.21E-20 |
| LINC01361 | 2.95 | 4.11 | 1.60E-21 | 1.30E-20 |
| RP11-62I21.1 | -2.19 | 3.77 | 1.67E-21 | 1.35E-20 |
| RP11-831A10.1 | 7.88 | 6.57 | 1.89E-21 | 1.53E-20 |
| RP4-813D12.3 | 3.62 | 5.20 | 2.20E-21 | 1.76E-20 |
| RP5-827C21.6 | 3.05 | 5.55 | 2.71E-21 | 2.16E-20 |
| RP11-384O8.1 | 2.70 | 7.62 | 2.94E-21 | 2.34E-20 |
| AP000696.2 | 4.90 | 4.06 | 2.97E-21 | 2.36E-20 |
| LINC01842 | 4.09 | 6.03 | 2.97E-21 | 2.36E-20 |
| LINC02041 | 2.73 | 6.99 | 3.17E-21 | 2.52E-20 |
| AP000697.6 | 4.14 | 3.07 | 3.84E-21 | 3.04E-20 |
| RP11-802D6.1 | 2.63 | 4.82 | 3.97E-21 | 3.14E-20 |
| LINC01116 | 2.92 | 8.76 | 4.15E-21 | 3.28E-20 |
| RP5-940J5.3 | 2.13 | 4.46 | 4.20E-21 | 3.32E-20 |
| CTD-2091N23.1 | 3.48 | 4.16 | 4.54E-21 | 3.59E-20 |
| RP11-332J15.4 | 3.41 | 4.74 | 4.83E-21 | 3.81E-20 |
| MLIP-IT1 | 3.44 | 4.28 | 4.90E-21 | 3.86E-20 |
| LINC00896 | 2.48 | 5.38 | 4.95E-21 | 3.90E-20 |
| IL20RB-AS1 | 4.75 | 3.93 | 5.31E-21 | 4.17E-20 |
| AF131217.1 | 3.78 | 9.84 | 5.45E-21 | 4.27E-20 |
| LINC01460 | 3.04 | 6.38 | 5.68E-21 | 4.45E-20 |
| LINC01732 | -2.23 | 2.82 | 5.75E-21 | 4.50E-20 |
| NKX2-1-AS1 | -2.45 | 6.99 | 5.94E-21 | 4.64E-20 |
| AF003625.3 | 7.58 | 6.59 | 6.02E-21 | 4.70E-20 |
| AC005863.1 | 5.65 | 5.31 | 6.43E-21 | 5.02E-20 |
| AC092669.6 | 4.49 | 3.22 | 8.45E-21 | 6.54E-20 |
| PTCSC2 | 4.58 | 4.84 | 8.92E-21 | 6.90E-20 |
| RP11-3J1.1 | 5.70 | 4.25 | 9.33E-21 | 7.20E-20 |
| RP11-545D19.1 | 5.67 | 3.99 | 1.12E-20 | 8.60E-20 |
| CTD-3032H12.1 | -2.36 | 2.86 | 1.15E-20 | 8.85E-20 |
| RP11-129M6.1 | 3.94 | 7.44 | 1.23E-20 | 9.42E-20 |
| RP11-276M12.1 | 2.36 | 4.51 | 1.25E-20 | 9.57E-20 |
| CTD-2066L21.3 | 5.22 | 5.58 | 1.30E-20 | 9.95E-20 |
| RP11-110A12.2 | 6.53 | 6.88 | 1.37E-20 | 1.05E-19 |
| LINC01391 | 5.67 | 4.65 | 1.38E-20 | 1.05E-19 |
| RP11-661A12.9 | 2.87 | 5.46 | 1.39E-20 | 1.07E-19 |
| RP11-343H19.1 | 2.31 | 4.07 | 1.45E-20 | 1.11E-19 |
| LINC01513 | -2.46 | 3.74 | 1.45E-20 | 1.11E-19 |
| RP11-360L9.7 | 3.17 | 3.17 | 1.46E-20 | 1.11E-19 |
| RP4-663N10.1 | -2.59 | 2.71 | 1.83E-20 | 1.39E-19 |
| RP4-781K5.9 | 4.37 | 3.53 | 1.85E-20 | 1.40E-19 |
| RP11-300E4.2 | 3.57 | 3.42 | 1.87E-20 | 1.42E-19 |
| RP11-104E19.1 | 5.37 | 6.70 | 1.92E-20 | 1.46E-19 |
| RP11-297P16.3 | 6.07 | 4.85 | 1.99E-20 | 1.51E-19 |
| AC005324.6 | -3.11 | 2.94 | 2.14E-20 | 1.62E-19 |
| RP11-112L6.3 | 2.25 | 5.48 | 2.26E-20 | 1.70E-19 |
| LINC01166 | -2.77 | 3.50 | 2.27E-20 | 1.71E-19 |
| RP11-327J17.2 | -2.10 | 2.59 | 2.28E-20 | 1.72E-19 |
| RP3-525N10.2 | -2.13 | 4.71 | 2.63E-20 | 1.98E-19 |
| RAPGEF4-AS1 | 3.16 | 3.31 | 2.88E-20 | 2.17E-19 |
| UCA1 | 4.83 | 8.66 | 3.29E-20 | 2.46E-19 |
| CTD-2023N9.1 | -2.66 | 2.73 | 3.65E-20 | 2.72E-19 |
| LINC01202 | 5.72 | 4.04 | 3.71E-20 | 2.77E-19 |
| LINC00173 | 2.33 | 7.08 | 3.87E-20 | 2.88E-19 |
| RP11-360O19.4 | 2.85 | 4.83 | 3.97E-20 | 2.95E-19 |
| RP11-259O2.1 | 3.30 | 7.18 | 4.09E-20 | 3.03E-19 |
| RP11-794G24.1 | 2.75 | 5.56 | 4.13E-20 | 3.06E-19 |
| AC123023.1 | -2.75 | 3.59 | 4.90E-20 | 3.62E-19 |
| RP11-215P8.4 | 5.76 | 6.23 | 4.91E-20 | 3.62E-19 |
| CTD-2619J13.13 | 2.45 | 6.83 | 4.92E-20 | 3.63E-19 |
| CTD-2544H17.1 | -3.05 | 4.06 | 4.99E-20 | 3.68E-19 |
| RP11-268G12.1 | 4.85 | 4.79 | 5.01E-20 | 3.69E-19 |
| AC114765.2 | 3.64 | 3.30 | 5.30E-20 | 3.89E-19 |
| AC012360.4 | 2.17 | 3.89 | 5.31E-20 | 3.90E-19 |
| LINC00466 | 4.41 | 3.82 | 5.86E-20 | 4.29E-19 |
| AC008088.4 | 3.78 | 3.62 | 6.14E-20 | 4.49E-19 |
| LINC00645 | 3.73 | 3.44 | 6.32E-20 | 4.61E-19 |
| RP11-734K21.2 | 3.51 | 4.77 | 6.54E-20 | 4.77E-19 |
| LINC00615 | 6.75 | 5.20 | 8.69E-20 | 6.30E-19 |
| STEAP2-AS1 | 2.74 | 3.82 | 9.04E-20 | 6.54E-19 |
| RP11-676J12.6 | -2.35 | 3.79 | 9.62E-20 | 6.94E-19 |
| LINC01607 | 2.04 | 6.16 | 1.04E-19 | 7.47E-19 |
| RP11-268F1.3 | -3.00 | 4.16 | 1.09E-19 | 7.81E-19 |
| KB-1440D3.13 | 2.32 | 4.28 | 1.15E-19 | 8.26E-19 |
| RP11-863P13.3 | 2.56 | 6.29 | 1.16E-19 | 8.33E-19 |
| RP11-608O21.1 | 5.36 | 4.68 | 1.28E-19 | 9.13E-19 |
| RP4-777L9.2 | 2.31 | 4.62 | 1.32E-19 | 9.44E-19 |
| KCNH1-IT1 | 5.16 | 4.20 | 1.33E-19 | 9.46E-19 |
| RP11-789C17.3 | -2.39 | 2.88 | 1.45E-19 | 1.03E-18 |
| RP11-616M22.7 | 5.12 | 4.94 | 1.45E-19 | 1.04E-18 |
| RP11-445P17.8 | 3.85 | 7.55 | 1.47E-19 | 1.05E-18 |
| RP11-57A19.2 | 3.34 | 6.60 | 1.57E-19 | 1.12E-18 |
| RP11-874J12.4 | 3.95 | 6.03 | 1.62E-19 | 1.15E-18 |
| RP11-204L24.2 | 2.09 | 5.26 | 1.72E-19 | 1.22E-18 |
| RP11-685G9.2 | 3.19 | 3.46 | 1.80E-19 | 1.27E-18 |
| RP4-621N11.2 | 2.44 | 3.69 | 1.80E-19 | 1.27E-18 |
| AC073043.1 | 2.27 | 3.98 | 1.94E-19 | 1.37E-18 |
| RP11-356K23.1 | -2.44 | 6.44 | 2.10E-19 | 1.48E-18 |
| CTB-189B5.3 | 3.01 | 3.46 | 2.19E-19 | 1.54E-18 |
| RP11-211G23.2 | 5.79 | 5.91 | 2.19E-19 | 1.54E-18 |
| RP11-62L18.3 | 4.94 | 3.42 | 2.38E-19 | 1.67E-18 |
| LINC01614 | 2.95 | 7.87 | 2.63E-19 | 1.84E-18 |
| CTA-384D8.35 | 2.21 | 8.47 | 2.71E-19 | 1.90E-18 |
| LINC01168 | -2.56 | 2.99 | 2.83E-19 | 1.98E-18 |
| RP11-734K21.5 | 3.50 | 6.30 | 2.83E-19 | 1.98E-18 |
| CTC-441N14.2 | -2.04 | 3.74 | 3.08E-19 | 2.14E-18 |
| LINC02159 | 3.59 | 7.25 | 3.25E-19 | 2.26E-18 |
| LINC00461 | 5.25 | 5.40 | 3.28E-19 | 2.28E-18 |
| SALRNA1 | 2.41 | 4.55 | 3.29E-19 | 2.28E-18 |
| RP4-735C1.4 | -2.13 | 3.32 | 3.50E-19 | 2.42E-18 |
| CTC-526N19.1 | 2.26 | 9.85 | 3.53E-19 | 2.44E-18 |
| RP4-784A16.3 | 3.38 | 3.13 | 3.62E-19 | 2.50E-18 |
| LINC02178 | 6.35 | 5.60 | 3.92E-19 | 2.71E-18 |
| LA16c-321D4.2 | 2.52 | 5.39 | 3.95E-19 | 2.72E-18 |
| RP11-123B3.2 | 4.12 | 4.60 | 4.04E-19 | 2.78E-18 |
| LINC01992 | 6.99 | 5.91 | 4.47E-19 | 3.07E-18 |
| RP11-1038A11.2 | 3.93 | 3.41 | 4.56E-19 | 3.13E-18 |
| RP11-311F12.1 | 3.75 | 6.94 | 4.71E-19 | 3.23E-18 |
| IGF2BP2-AS1 | 3.23 | 5.27 | 4.84E-19 | 3.31E-18 |
| RP1-296L11.1 | 3.69 | 3.03 | 5.08E-19 | 3.47E-18 |
| LINC01527 | 5.21 | 6.25 | 5.13E-19 | 3.51E-18 |
| RP11-59D5__B.2 | 3.87 | 8.32 | 5.28E-19 | 3.60E-18 |
| RP11-338H14.1 | 4.60 | 5.36 | 5.39E-19 | 3.67E-18 |
| CTA-384D8.31 | 4.41 | 7.81 | 5.82E-19 | 3.96E-18 |
| SMCR2 | 2.43 | 3.35 | 6.05E-19 | 4.12E-18 |
| AC074286.1 | 2.41 | 10.33 | 6.21E-19 | 4.21E-18 |
| RP11-85G21.2 | -2.80 | 3.85 | 6.30E-19 | 4.27E-18 |
| AC018890.6 | 4.03 | 7.49 | 6.31E-19 | 4.28E-18 |
| RP5-1120P11.1 | 2.54 | 8.46 | 6.42E-19 | 4.34E-18 |
| RP11-225N10.1 | 3.94 | 4.67 | 6.47E-19 | 4.37E-18 |
| LINC01297 | 6.12 | 4.42 | 6.80E-19 | 4.59E-18 |
| AC007966.1 | 2.67 | 5.06 | 7.66E-19 | 5.15E-18 |
| RP11-1012E15.2 | 4.54 | 5.93 | 7.81E-19 | 5.25E-18 |
| AC007193.6 | 4.24 | 5.65 | 8.29E-19 | 5.56E-18 |
| HOXB-AS4 | 5.10 | 5.20 | 8.31E-19 | 5.57E-18 |
| RP11-143E21.3 | 5.88 | 5.88 | 8.40E-19 | 5.62E-18 |
| CTC-327F10.5 | 3.74 | 3.45 | 9.22E-19 | 6.16E-18 |
| CTC-232P5.3 | -2.39 | 3.00 | 1.01E-18 | 6.71E-18 |
| RP11-422J15.1 | 3.72 | 2.73 | 1.11E-18 | 7.35E-18 |
| XXYLT1-AS1 | 2.93 | 3.99 | 1.20E-18 | 7.94E-18 |
| LINC00392 | 9.38 | 7.92 | 1.23E-18 | 8.17E-18 |
| LINC01416 | 3.58 | 3.32 | 1.28E-18 | 8.48E-18 |
| RP11-17A4.2 | -2.31 | 3.73 | 1.34E-18 | 8.84E-18 |
| RP11-34D15.2 | -2.09 | 2.49 | 1.49E-18 | 9.86E-18 |
| KB-1836B5.4 | 2.45 | 4.58 | 1.57E-18 | 1.04E-17 |
| LINC00470 | 4.17 | 7.66 | 1.66E-18 | 1.09E-17 |
| RP11-895M11.3 | 2.31 | 3.64 | 1.72E-18 | 1.13E-17 |
| PTGES2-AS1 | 2.11 | 4.56 | 1.78E-18 | 1.17E-17 |
| DGCR9 | 2.27 | 6.75 | 1.85E-18 | 1.21E-17 |
| AL163953.2 | 6.38 | 4.76 | 1.91E-18 | 1.25E-17 |
| RP4-734C18.1 | 5.77 | 5.31 | 2.40E-18 | 1.56E-17 |
| RP11-495P10.5 | 3.70 | 3.81 | 2.70E-18 | 1.76E-17 |
| RP11-366L20.3 | 3.69 | 3.50 | 2.73E-18 | 1.77E-17 |
| RP5-827C21.2 | 2.91 | 4.98 | 2.82E-18 | 1.83E-17 |
| LINC02137 | 3.58 | 5.70 | 2.92E-18 | 1.89E-17 |
| ATP11A-AS1 | -2.02 | 2.51 | 3.07E-18 | 1.99E-17 |
| LINC00462 | 5.12 | 5.63 | 3.15E-18 | 2.03E-17 |
| RP11-284F21.10 | 3.85 | 10.37 | 3.24E-18 | 2.09E-17 |
| AC007389.3 | 4.14 | 4.63 | 3.26E-18 | 2.10E-17 |
| RP11-255G12.3 | 4.47 | 4.06 | 3.43E-18 | 2.21E-17 |
| RP11-398G24.2 | 4.70 | 3.49 | 3.73E-18 | 2.40E-17 |
| APCDD1L-AS1 | 3.88 | 6.65 | 4.00E-18 | 2.57E-17 |
| RP11-557H15.3 | 4.42 | 8.64 | 4.02E-18 | 2.58E-17 |
| MKRN3-AS1 | 3.74 | 3.87 | 4.15E-18 | 2.65E-17 |
| CTD-2066L21.2 | 5.10 | 3.88 | 4.43E-18 | 2.83E-17 |
| PCAT1 | 2.05 | 6.78 | 4.51E-18 | 2.88E-17 |
| LINC01592 | 3.78 | 4.13 | 4.61E-18 | 2.94E-17 |
| LINP1 | 4.28 | 7.12 | 5.80E-18 | 3.69E-17 |
| DSCR9 | 2.34 | 5.05 | 5.95E-18 | 3.78E-17 |
| LINC00536 | 5.75 | 4.78 | 5.99E-18 | 3.81E-17 |
| RP1-202O8.2 | 3.10 | 4.28 | 6.55E-18 | 4.16E-17 |
| DLG1-AS1 | 2.07 | 5.89 | 6.56E-18 | 4.16E-17 |
| RP13-497K6.1 | -2.42 | 2.88 | 6.57E-18 | 4.17E-17 |
| AC091729.7 | 2.95 | 5.00 | 6.97E-18 | 4.42E-17 |
| RP11-429J17.5 | 2.84 | 4.65 | 7.01E-18 | 4.44E-17 |
| AC007182.6 | -2.18 | 4.04 | 7.03E-18 | 4.45E-17 |
| RP11-484K9.4 | 2.01 | 4.18 | 7.25E-18 | 4.58E-17 |
| RP11-169F17.1 | 7.63 | 8.62 | 7.40E-18 | 4.67E-17 |
| TM4SF1-AS1 | 2.72 | 7.12 | 7.89E-18 | 4.97E-17 |
| RP11-417E7.2 | 2.61 | 5.84 | 8.69E-18 | 5.47E-17 |
| RP11-120K24.5 | 2.94 | 5.19 | 9.44E-18 | 5.93E-17 |
| RP11-674N23.4 | 2.46 | 3.71 | 9.73E-18 | 6.11E-17 |
| CTB-60B18.12 | 2.54 | 3.77 | 9.93E-18 | 6.23E-17 |
| RP11-1020M18.10 | 5.31 | 4.21 | 1.03E-17 | 6.45E-17 |
| ATP6V1B1-AS1 | 3.09 | 4.12 | 1.05E-17 | 6.55E-17 |
| CTB-33O18.1 | 5.34 | 4.60 | 1.05E-17 | 6.57E-17 |
| TBL1XR1-AS1 | 2.56 | 3.79 | 1.09E-17 | 6.79E-17 |
| RP11-133K1.7 | 2.61 | 2.76 | 1.10E-17 | 6.88E-17 |
| LINC01429 | 3.36 | 3.34 | 1.11E-17 | 6.95E-17 |
| CTD-2314B22.1 | 5.92 | 4.03 | 1.20E-17 | 7.50E-17 |
| LINC01117 | 2.59 | 5.28 | 1.27E-17 | 7.88E-17 |
| RP3-388N13.5 | 2.22 | 3.57 | 1.31E-17 | 8.16E-17 |
| RP11-150O12.6 | 2.93 | 7.48 | 1.35E-17 | 8.39E-17 |
| GDNF-AS1 | 3.74 | 6.62 | 1.48E-17 | 9.17E-17 |
| RP11-6N13.1 | 4.83 | 3.25 | 1.54E-17 | 9.52E-17 |
| RP11-20G13.1 | 3.38 | 5.09 | 1.62E-17 | 1.00E-16 |
| MIR31HG | 4.28 | 6.55 | 1.75E-17 | 1.08E-16 |
| RP5-1011O1.2 | 3.53 | 5.32 | 1.79E-17 | 1.10E-16 |
| RP11-1069G10.2 | 3.86 | 3.97 | 2.09E-17 | 1.28E-16 |
| LINC01697 | 4.71 | 7.22 | 2.10E-17 | 1.29E-16 |
| AC022201.4 | 5.40 | 3.99 | 2.14E-17 | 1.31E-16 |
| RP11-722P11.4 | 3.54 | 3.65 | 2.32E-17 | 1.42E-16 |
| RP11-203H2.2 | -2.57 | 2.90 | 2.34E-17 | 1.43E-16 |
| LINC00665 | 2.26 | 11.28 | 2.40E-17 | 1.47E-16 |
| F11-AS1 | -2.33 | 5.22 | 2.67E-17 | 1.63E-16 |
| RP11-839D17.3 | 2.32 | 5.26 | 2.81E-17 | 1.71E-16 |
| RP1-46F2.3 | 6.14 | 5.50 | 3.07E-17 | 1.86E-16 |
| RP11-230L22.4 | -2.42 | 2.99 | 3.29E-17 | 2.00E-16 |
| AC002310.7 | 2.00 | 3.52 | 3.32E-17 | 2.01E-16 |
| AP000688.14 | 2.26 | 6.20 | 3.48E-17 | 2.10E-16 |
| RP11-256I23.3 | 3.41 | 3.46 | 3.71E-17 | 2.24E-16 |
| RP11-391H12.8 | 2.81 | 4.41 | 3.73E-17 | 2.25E-16 |
| AC068196.1 | 2.21 | 3.18 | 3.98E-17 | 2.40E-16 |
| RP11-465L10.10 | 2.23 | 6.00 | 4.18E-17 | 2.52E-16 |
| RP11-586K2.1 | 3.03 | 3.92 | 4.46E-17 | 2.68E-16 |
| CTA-390C10.9 | 3.74 | 3.07 | 4.49E-17 | 2.69E-16 |
| AC079610.1 | 3.27 | 4.57 | 5.11E-17 | 3.06E-16 |
| RP11-467L19.16 | 5.26 | 5.92 | 5.13E-17 | 3.07E-16 |
| RP11-367F23.2 | 5.42 | 5.06 | 5.13E-17 | 3.07E-16 |
| WNT5A-AS1 | 2.31 | 7.44 | 5.31E-17 | 3.17E-16 |
| RP11-284F21.7 | 3.29 | 7.83 | 5.41E-17 | 3.23E-16 |
| AC091801.1 | 5.32 | 4.80 | 5.46E-17 | 3.26E-16 |
| RP11-659E9.4 | 3.15 | 2.80 | 5.52E-17 | 3.29E-16 |
| KB-1930G5.4 | 3.48 | 3.10 | 6.11E-17 | 3.63E-16 |
| RP11-829H16.3 | 2.73 | 4.96 | 6.38E-17 | 3.79E-16 |
| LA16c-312E8.2 | 2.53 | 3.19 | 6.56E-17 | 3.89E-16 |
| RP11-284F21.9 | 4.36 | 8.62 | 6.69E-17 | 3.96E-16 |
| CASC11 | 3.15 | 5.44 | 6.69E-17 | 3.96E-16 |
| RP5-965G21.4 | 2.16 | 6.72 | 6.78E-17 | 4.01E-16 |
| CTD-2066L21.1 | 5.02 | 3.66 | 6.83E-17 | 4.03E-16 |
| CTA-246H3.12 | 3.26 | 3.52 | 6.87E-17 | 4.06E-16 |
| RP11-336A10.4 | 4.80 | 6.77 | 6.92E-17 | 4.08E-16 |
| RP11-354I13.1 | 5.09 | 3.59 | 7.35E-17 | 4.33E-16 |
| RP11-728G15.1 | 4.33 | 3.80 | 7.51E-17 | 4.42E-16 |
| CTC-420A11.2 | 3.36 | 4.31 | 7.61E-17 | 4.47E-16 |
| RP11-54O7.1 | 2.96 | 5.39 | 7.91E-17 | 4.64E-16 |
| TEX41 | 2.25 | 8.04 | 8.18E-17 | 4.79E-16 |
| RP11-235G24.3 | 5.24 | 3.69 | 8.83E-17 | 5.16E-16 |
| ELDR | 4.92 | 5.73 | 8.98E-17 | 5.25E-16 |
| RP11-186F10.2 | 3.60 | 3.66 | 9.36E-17 | 5.46E-16 |
| LINC00184 | 2.70 | 5.00 | 9.40E-17 | 5.48E-16 |
| FLJ42969 | 2.70 | 4.97 | 9.43E-17 | 5.50E-16 |
| CH507-42P11.6 | 2.81 | 3.49 | 9.90E-17 | 5.77E-16 |
| MIR137HG | 6.28 | 5.90 | 1.06E-16 | 6.14E-16 |
| AC005330.2 | 2.86 | 5.38 | 1.31E-16 | 7.60E-16 |
| LINC02141 | 5.39 | 4.00 | 1.35E-16 | 7.80E-16 |
| RP11-10A14.4 | 2.16 | 5.66 | 1.48E-16 | 8.55E-16 |
| CTD-2021H9.2 | 3.36 | 3.09 | 1.62E-16 | 9.30E-16 |
| RP11-1055B8.9 | 2.36 | 4.18 | 1.66E-16 | 9.54E-16 |
| RP11-65M17.3 | 3.91 | 4.62 | 1.82E-16 | 1.04E-15 |
| AFAP1-AS1 | 4.71 | 10.99 | 1.83E-16 | 1.05E-15 |
| RP11-25L3.3 | 3.53 | 4.03 | 1.87E-16 | 1.07E-15 |
| RP11-432J24.5 | -2.16 | 3.24 | 2.04E-16 | 1.16E-15 |
| RP11-150C16.1 | 2.60 | 5.23 | 2.15E-16 | 1.22E-15 |
| AC093702.1 | 3.48 | 4.41 | 2.20E-16 | 1.25E-15 |
| LINC00492 | 3.03 | 2.57 | 2.26E-16 | 1.28E-15 |
| RP3-428L16.1 | 4.02 | 3.73 | 2.55E-16 | 1.45E-15 |
| LINC02081 | 2.41 | 6.85 | 2.68E-16 | 1.52E-15 |
| RP11-993B23.3 | 3.04 | 6.79 | 2.71E-16 | 1.53E-15 |
| LINC01194 | 8.06 | 6.70 | 2.78E-16 | 1.57E-15 |
| RP11-319E16.2 | 3.30 | 2.69 | 2.85E-16 | 1.61E-15 |
| RP11-561P12.5 | -2.53 | 2.63 | 3.00E-16 | 1.69E-15 |
| AC069257.8 | 2.01 | 4.01 | 3.01E-16 | 1.70E-15 |
| LINC01956 | 5.18 | 5.00 | 3.07E-16 | 1.73E-15 |
| RP11-124O11.1 | -2.36 | 3.06 | 3.41E-16 | 1.92E-15 |
| RP11-404J23.1 | 5.83 | 4.09 | 3.54E-16 | 1.99E-15 |
| RP11-114H21.2 | 6.01 | 4.43 | 3.55E-16 | 1.99E-15 |
| RP1-45C12.1 | 2.35 | 5.38 | 3.59E-16 | 2.01E-15 |
| RP1-97J1.2 | 3.86 | 4.95 | 3.61E-16 | 2.02E-15 |
| RP11-616M22.5 | -2.05 | 4.47 | 3.84E-16 | 2.15E-15 |
| RP11-395E19.6 | -2.83 | 3.35 | 3.97E-16 | 2.21E-15 |
| RP11-642C5.1 | 3.37 | 3.65 | 3.99E-16 | 2.22E-15 |
| CH507-154B10.2 | 2.40 | 3.45 | 4.25E-16 | 2.37E-15 |
| AC092484.1 | 5.40 | 4.87 | 4.31E-16 | 2.40E-15 |
| LINC00391 | 3.75 | 3.34 | 4.55E-16 | 2.53E-15 |
| CTD-2083E4.7 | 4.33 | 4.46 | 4.88E-16 | 2.71E-15 |
| RP11-1000B6.2 | 2.69 | 2.83 | 4.88E-16 | 2.71E-15 |
| RP11-123O10.4 | 2.92 | 3.60 | 5.10E-16 | 2.82E-15 |
| LINC01629 | 4.56 | 6.19 | 5.33E-16 | 2.95E-15 |
| RP11-124N19.3 | 3.22 | 4.78 | 5.60E-16 | 3.09E-15 |
| AC053503.4 | 2.24 | 4.31 | 5.64E-16 | 3.11E-15 |
| RP11-486O13.2 | -2.38 | 2.62 | 5.82E-16 | 3.21E-15 |
| RP11-567M16.1 | 3.27 | 8.00 | 5.98E-16 | 3.29E-15 |
| RP11-370A5.1 | 2.04 | 4.60 | 6.62E-16 | 3.63E-15 |
| FLJ12825 | 2.09 | 5.10 | 7.14E-16 | 3.91E-15 |
| RP4-794I6.4 | 2.17 | 8.88 | 7.19E-16 | 3.94E-15 |
| RP11-10A14.9 | 2.62 | 3.84 | 7.25E-16 | 3.97E-15 |
| LINC01583 | 3.21 | 4.47 | 7.30E-16 | 3.99E-15 |
| STEAP3-AS1 | 2.20 | 6.56 | 7.55E-16 | 4.12E-15 |
| AC068580.7 | 3.75 | 3.55 | 7.96E-16 | 4.34E-15 |
| RP11-776A13.1 | 4.92 | 3.57 | 7.97E-16 | 4.35E-15 |
| ATP13A4-AS1 | -2.59 | 5.55 | 8.00E-16 | 4.36E-15 |
| LINC02086 | 3.18 | 6.82 | 8.14E-16 | 4.43E-15 |
| RP11-13K12.2 | 5.05 | 5.83 | 8.53E-16 | 4.64E-15 |
| CTD-2008P7.8 | 6.46 | 5.39 | 8.95E-16 | 4.86E-15 |
| WASIR2 | 2.56 | 4.98 | 9.94E-16 | 5.38E-15 |
| RP11-706C16.8 | 4.22 | 3.34 | 1.12E-15 | 6.03E-15 |
| RP11-68I3.10 | 2.51 | 3.52 | 1.14E-15 | 6.13E-15 |
| RP11-21C17.1 | 3.99 | 3.05 | 1.14E-15 | 6.14E-15 |
| CTD-2021H9.1 | 3.44 | 3.28 | 1.15E-15 | 6.19E-15 |
| RP11-425D17.2 | 2.88 | 5.34 | 1.17E-15 | 6.29E-15 |
| LINC00520 | 3.42 | 7.15 | 1.17E-15 | 6.29E-15 |
| RP11-399K21.13 | 2.18 | 3.29 | 1.29E-15 | 6.93E-15 |
| ELFN1-AS1 | 3.84 | 6.46 | 1.31E-15 | 7.01E-15 |
| RP11-280K24.4 | 3.71 | 4.88 | 1.37E-15 | 7.34E-15 |
| CTD-2013M15.1 | 4.91 | 4.59 | 1.39E-15 | 7.44E-15 |
| RP11-38M8.1 | 2.25 | 6.34 | 1.40E-15 | 7.48E-15 |
| RP11-445P17.3 | 2.75 | 3.75 | 1.43E-15 | 7.65E-15 |
| AC004920.3 | 3.85 | 2.82 | 1.47E-15 | 7.83E-15 |
| AC003986.6 | 2.73 | 3.64 | 1.65E-15 | 8.78E-15 |
| CTD-2194D22.4 | 4.67 | 3.16 | 1.77E-15 | 9.42E-15 |
| RP11-297P16.4 | 7.88 | 8.73 | 1.83E-15 | 9.74E-15 |
| LHFPL3-AS1 | -2.29 | 3.87 | 1.83E-15 | 9.74E-15 |
| RP11-26L20.3 | 3.47 | 3.95 | 1.85E-15 | 9.84E-15 |
| RP11-565P22.2 | 5.78 | 5.41 | 1.92E-15 | 1.02E-14 |
| NOVA1-AS1 | 5.24 | 5.17 | 2.28E-15 | 1.21E-14 |
| CTD-2587M23.1 | 4.60 | 4.47 | 2.31E-15 | 1.22E-14 |
| ZFHX4-AS1 | 5.32 | 5.68 | 2.31E-15 | 1.22E-14 |
| RP11-430H10.2 | 5.00 | 5.08 | 2.39E-15 | 1.26E-14 |
| LINC01811 | -2.43 | 3.05 | 2.43E-15 | 1.28E-14 |
| FMR1-AS1 | 2.20 | 3.26 | 2.46E-15 | 1.29E-14 |
| LINC00992 | 2.76 | 6.92 | 2.47E-15 | 1.30E-14 |
| RP11-370A5.2 | 3.18 | 4.60 | 2.48E-15 | 1.30E-14 |
| CTB-49A3.2 | 2.54 | 4.35 | 2.78E-15 | 1.45E-14 |
| RP4-536B24.4 | 2.64 | 3.43 | 2.83E-15 | 1.48E-14 |
| LINC00862 | 2.23 | 4.70 | 2.87E-15 | 1.50E-14 |
| RP13-631K18.3 | 3.13 | 4.17 | 3.16E-15 | 1.64E-14 |
| RP11-498M15.1 | 4.18 | 3.62 | 3.41E-15 | 1.77E-14 |
| LINC01419 | 9.26 | 7.83 | 3.47E-15 | 1.80E-14 |
| AC016723.4 | 5.57 | 5.21 | 3.60E-15 | 1.87E-14 |
| AC005042.5 | 4.32 | 3.26 | 3.65E-15 | 1.89E-14 |
| AC091814.3 | 3.24 | 5.48 | 3.92E-15 | 2.03E-14 |
| RP11-320G10.1 | 5.14 | 5.19 | 3.95E-15 | 2.04E-14 |
| RP3-395M20.3 | 2.97 | 4.48 | 4.16E-15 | 2.15E-14 |
| RP11-771K4.1 | 3.22 | 4.24 | 4.31E-15 | 2.22E-14 |
| RP11-78C3.1 | 5.17 | 4.37 | 4.67E-15 | 2.41E-14 |
| CTB-32H22.1 | 2.74 | 2.86 | 5.06E-15 | 2.60E-14 |
| AC007249.3 | 2.68 | 5.92 | 5.29E-15 | 2.72E-14 |
| LINC00518 | 5.02 | 4.40 | 5.32E-15 | 2.73E-14 |
| MIR548XHG | 7.72 | 6.39 | 5.56E-15 | 2.85E-14 |
| CTC-273B12.10 | 2.25 | 3.78 | 5.88E-15 | 3.01E-14 |
| AC068138.1 | 6.19 | 4.93 | 6.02E-15 | 3.08E-14 |
| RP11-568A7.3 | -2.81 | 3.41 | 6.05E-15 | 3.09E-14 |
| RP11-386B13.4 | 4.69 | 3.50 | 6.12E-15 | 3.13E-14 |
| RP11-305F18.1 | 4.42 | 5.08 | 6.23E-15 | 3.18E-14 |
| RP1-193H18.3 | -2.08 | 2.59 | 7.60E-15 | 3.87E-14 |
| RP11-109M17.2 | 4.07 | 5.53 | 7.74E-15 | 3.94E-14 |
| LINC01994 | 4.22 | 3.86 | 7.79E-15 | 3.96E-14 |
| RP4-547N15.3 | -2.63 | 4.61 | 8.27E-15 | 4.19E-14 |
| RP11-573J24.1 | 4.33 | 2.95 | 8.32E-15 | 4.22E-14 |
| RP11-283G6.5 | 4.28 | 4.75 | 8.72E-15 | 4.41E-14 |
| RP3-522D1.1 | 2.97 | 4.45 | 9.06E-15 | 4.57E-14 |
| LINC00629 | 2.14 | 3.97 | 9.69E-15 | 4.88E-14 |
| RP1-86C11.7 | 2.09 | 6.22 | 9.74E-15 | 4.90E-14 |
| RP11-493L12.3 | 3.67 | 4.84 | 9.90E-15 | 4.98E-14 |
| RP11-180M15.3 | 3.03 | 2.82 | 1.02E-14 | 5.10E-14 |
| PCAT2 | 3.01 | 3.19 | 1.03E-14 | 5.17E-14 |
| RP11-148B18.1 | -2.29 | 2.57 | 1.11E-14 | 5.57E-14 |
| RP11-311F12.2 | 4.64 | 5.40 | 1.14E-14 | 5.68E-14 |
| LINC01854 | 7.15 | 5.75 | 1.14E-14 | 5.71E-14 |
| OVOL1-AS1 | 2.41 | 4.00 | 1.16E-14 | 5.81E-14 |
| RP11-286B14.1 | 3.74 | 4.15 | 1.23E-14 | 6.12E-14 |
| SAMD12-AS1 | 2.32 | 6.82 | 1.24E-14 | 6.20E-14 |
| AC005162.5 | 3.12 | 3.90 | 1.31E-14 | 6.50E-14 |
| LINC01896 | 6.99 | 5.44 | 1.33E-14 | 6.63E-14 |
| RP6-24A23.7 | 5.79 | 9.57 | 1.44E-14 | 7.11E-14 |
| RP11-35O15.1 | 2.02 | 4.34 | 1.44E-14 | 7.12E-14 |
| RP11-66B24.7 | 2.36 | 6.59 | 1.45E-14 | 7.20E-14 |
| AC024560.2 | 3.08 | 4.91 | 1.46E-14 | 7.21E-14 |
| RP11-304L19.3 | 2.12 | 5.89 | 1.50E-14 | 7.40E-14 |
| RP11-681L8.1 | 4.44 | 3.92 | 1.51E-14 | 7.43E-14 |
| RP1-118J21.5 | 2.84 | 3.81 | 1.57E-14 | 7.72E-14 |
| RP11-81H3.2 | 5.64 | 5.78 | 1.60E-14 | 7.86E-14 |
| LINC01918 | 3.02 | 3.74 | 1.61E-14 | 7.92E-14 |
| FLJ37505 | 6.35 | 5.75 | 1.65E-14 | 8.09E-14 |
| LINC01287 | 6.64 | 8.41 | 1.68E-14 | 8.24E-14 |
| CTD-2116N20.1 | 2.06 | 3.94 | 1.71E-14 | 8.37E-14 |
| RP11-217E22.5 | 4.33 | 3.32 | 1.78E-14 | 8.69E-14 |
| RP11-498C9.12 | 2.59 | 6.70 | 1.82E-14 | 8.90E-14 |
| RP11-476K15.1 | 5.35 | 5.57 | 1.84E-14 | 8.95E-14 |
| LINC01571 | -2.77 | 3.84 | 1.85E-14 | 9.03E-14 |
| RP11-21L23.4 | 4.26 | 5.90 | 1.94E-14 | 9.42E-14 |
| RP11-329E24.6 | 4.83 | 3.70 | 2.14E-14 | 1.04E-13 |
| RP11-153K16.1 | 5.29 | 4.43 | 2.15E-14 | 1.04E-13 |
| RP11-128P10.1 | 3.09 | 2.52 | 2.19E-14 | 1.06E-13 |
| FAM222A-AS1 | 2.44 | 5.30 | 2.19E-14 | 1.06E-13 |
| AL109761.5 | 2.54 | 4.45 | 2.25E-14 | 1.09E-13 |
| RP11-378I6.1 | 3.53 | 4.29 | 2.26E-14 | 1.09E-13 |
| RP6-24A23.3 | 4.42 | 6.70 | 2.53E-14 | 1.22E-13 |
| AC112721.2 | 2.52 | 4.41 | 2.54E-14 | 1.23E-13 |
| RP11-314D7.2 | 4.29 | 3.39 | 2.56E-14 | 1.23E-13 |
| RRM1-AS1 | 2.24 | 2.72 | 2.62E-14 | 1.26E-13 |
| LINC01214 | 4.44 | 3.94 | 2.68E-14 | 1.29E-13 |
| RP11-10A14.5 | 3.47 | 6.17 | 2.79E-14 | 1.34E-13 |
| CTD-2566J3.1 | 6.24 | 5.80 | 2.81E-14 | 1.35E-13 |
| MUC2 | 5.94 | 9.06 | 2.81E-14 | 1.35E-13 |
| KB-1460A1.2 | 2.62 | 2.76 | 2.89E-14 | 1.39E-13 |
| RP11-180I4.4 | -2.48 | 3.22 | 2.91E-14 | 1.40E-13 |
| RP11-69G7.1 | 6.62 | 6.56 | 2.94E-14 | 1.41E-13 |
| RP11-304L19.1 | 2.01 | 6.08 | 3.18E-14 | 1.52E-13 |
| RP11-30P6.6 | 3.28 | 5.19 | 3.44E-14 | 1.64E-13 |
| AC007405.8 | 3.44 | 3.18 | 3.56E-14 | 1.69E-13 |
| LINC00704 | 3.08 | 6.88 | 3.65E-14 | 1.73E-13 |
| LINC01448 | 4.89 | 3.42 | 3.80E-14 | 1.80E-13 |
| MIAT | 2.29 | 11.82 | 3.82E-14 | 1.81E-13 |
| AC009955.8 | 3.00 | 2.67 | 3.86E-14 | 1.83E-13 |
| LINC01639 | 5.71 | 4.03 | 3.86E-14 | 1.83E-13 |
| RP1-122P22.4 | 2.58 | 7.33 | 4.07E-14 | 1.92E-13 |
| RP4-583K8.1 | 4.19 | 2.88 | 4.09E-14 | 1.93E-13 |
| RP11-436D23.1 | 4.21 | 3.49 | 4.10E-14 | 1.94E-13 |
| RP11-26J3.1 | 2.08 | 4.77 | 4.14E-14 | 1.95E-13 |
| POU6F2-AS1 | 3.99 | 2.78 | 4.22E-14 | 1.99E-13 |
| ESRG | 7.14 | 9.16 | 4.47E-14 | 2.10E-13 |
| RP3-395M20.2 | 2.73 | 4.45 | 4.51E-14 | 2.12E-13 |
| RP11-675F6.3 | 4.35 | 4.62 | 4.56E-14 | 2.14E-13 |
| CTC-338M12.9 | 3.35 | 5.14 | 4.66E-14 | 2.18E-13 |
| AC006000.5 | 3.49 | 3.50 | 4.82E-14 | 2.25E-13 |
| RP11-85O21.2 | 4.57 | 3.33 | 4.86E-14 | 2.28E-13 |
| RP11-662I13.2 | 3.20 | 4.47 | 4.88E-14 | 2.28E-13 |
| CTD-2523D13.1 | 2.80 | 3.01 | 5.04E-14 | 2.35E-13 |
| RP11-314N14.1 | 7.43 | 5.33 | 5.10E-14 | 2.38E-13 |
| UNC5B-AS1 | 2.09 | 6.67 | 5.15E-14 | 2.40E-13 |
| EWSAT1 | 3.26 | 6.92 | 5.22E-14 | 2.44E-13 |
| RP11-1070A24.2 | 3.62 | 3.24 | 5.26E-14 | 2.45E-13 |
| WI2-85898F10.1 | 2.80 | 4.08 | 5.39E-14 | 2.51E-13 |
| RP11-259O2.3 | 3.61 | 3.54 | 5.62E-14 | 2.61E-13 |
| RP11-962G15.1 | 6.61 | 4.82 | 5.65E-14 | 2.62E-13 |
| AC003088.1 | 3.11 | 3.41 | 5.85E-14 | 2.71E-13 |
| RP11-573D15.3 | 3.62 | 3.16 | 5.99E-14 | 2.77E-13 |
| RP11-445F12.1 | 6.16 | 5.10 | 6.01E-14 | 2.78E-13 |
| RP11-348J24.2 | 2.97 | 4.32 | 6.18E-14 | 2.86E-13 |
| TLR8-AS1 | -2.10 | 3.39 | 6.51E-14 | 3.00E-13 |
| LINC02068 | 2.33 | 6.01 | 6.71E-14 | 3.09E-13 |
| GRM5-AS1 | 3.74 | 3.58 | 7.14E-14 | 3.28E-13 |
| RP11-66D17.3 | 2.74 | 3.06 | 7.39E-14 | 3.39E-13 |
| RP11-142A23.1 | 2.37 | 3.78 | 7.92E-14 | 3.62E-13 |
| KIAA0087 | -2.10 | 3.14 | 8.25E-14 | 3.77E-13 |
| RP11-146E13.4 | 2.96 | 3.50 | 8.38E-14 | 3.82E-13 |
| RP11-669N7.2 | 7.65 | 6.50 | 9.02E-14 | 4.11E-13 |
| RP11-85B7.2 | 2.03 | 5.04 | 9.72E-14 | 4.43E-13 |
| LINC01399 | 3.01 | 3.44 | 1.01E-13 | 4.61E-13 |
| LINC01549 | 4.89 | 4.82 | 1.01E-13 | 4.61E-13 |
| RP11-478J18.2 | 3.63 | 4.31 | 1.01E-13 | 4.61E-13 |
| RP11-3L21.2 | 4.54 | 4.71 | 1.03E-13 | 4.66E-13 |
| LINC00332 | -2.52 | 3.11 | 1.03E-13 | 4.69E-13 |
| AP003900.6 | 6.13 | 4.45 | 1.09E-13 | 4.95E-13 |
| CTD-2210P24.1 | 5.06 | 3.61 | 1.13E-13 | 5.10E-13 |
| RP11-626E13.1 | -2.07 | 2.56 | 1.13E-13 | 5.11E-13 |
| AF212831.2 | 3.46 | 2.62 | 1.16E-13 | 5.23E-13 |
| AC093390.1 | -2.29 | 3.09 | 1.17E-13 | 5.27E-13 |
| SAMMSON | 2.98 | 3.59 | 1.19E-13 | 5.36E-13 |
| AC084149.2 | 4.06 | 2.95 | 1.22E-13 | 5.48E-13 |
| CTD-2529O21.1 | 2.43 | 3.33 | 1.23E-13 | 5.53E-13 |
| RP11-332J15.3 | 2.53 | 3.86 | 1.24E-13 | 5.58E-13 |
| LINC00557 | 4.00 | 3.65 | 1.28E-13 | 5.74E-13 |
| LINC02208 | 3.60 | 3.98 | 1.35E-13 | 6.07E-13 |
| CTD-2147F2.2 | 3.15 | 3.62 | 1.49E-13 | 6.64E-13 |
| LINC02031 | 3.78 | 6.65 | 1.54E-13 | 6.86E-13 |
| RP11-187E13.1 | 3.31 | 3.70 | 1.90E-13 | 8.45E-13 |
| LINC01356 | 2.63 | 5.21 | 1.91E-13 | 8.46E-13 |
| LINC01908 | -2.24 | 3.65 | 2.08E-13 | 9.22E-13 |
| RP11-587P21.2 | 7.46 | 6.12 | 2.12E-13 | 9.37E-13 |
| AC011298.2 | 4.94 | 4.60 | 2.15E-13 | 9.48E-13 |
| KB-1460A1.3 | 2.04 | 3.07 | 2.15E-13 | 9.50E-13 |
| TLX1NB | 5.08 | 3.68 | 2.16E-13 | 9.51E-13 |
| LINC01127 | 3.53 | 9.08 | 2.24E-13 | 9.84E-13 |
| AC005329.7 | 2.39 | 4.65 | 2.31E-13 | 1.02E-12 |
| RP11-818F20.5 | 3.54 | 6.05 | 2.43E-13 | 1.07E-12 |
| LINC00707 | 3.09 | 6.67 | 2.44E-13 | 1.07E-12 |
| RP11-644C3.1 | 3.22 | 3.20 | 2.48E-13 | 1.09E-12 |
| CTD-2377O17.1 | 2.13 | 4.24 | 2.52E-13 | 1.10E-12 |
| LINC00974 | 3.17 | 2.99 | 2.53E-13 | 1.11E-12 |
| RP11-67L3.2 | 2.04 | 3.54 | 2.54E-13 | 1.11E-12 |
| LINC00501 | 4.58 | 5.14 | 2.59E-13 | 1.13E-12 |
| LINC00504 | 2.81 | 8.52 | 2.70E-13 | 1.18E-12 |
| RP11-556I14.2 | 2.46 | 3.21 | 2.72E-13 | 1.19E-12 |
| RP11-462P6.1 | 2.47 | 2.64 | 2.75E-13 | 1.20E-12 |
| FAM181A-AS1 | -2.02 | 5.10 | 2.81E-13 | 1.23E-12 |
| RP11-734K21.3 | 3.82 | 3.48 | 2.82E-13 | 1.23E-12 |
| RP11-412P11.1 | 5.63 | 4.43 | 2.89E-13 | 1.26E-12 |
| RP11-449P1.1 | 4.53 | 4.13 | 3.02E-13 | 1.31E-12 |
| RP11-290K4.2 | 4.01 | 2.79 | 3.08E-13 | 1.34E-12 |
| RP11-220I1.2 | 2.52 | 3.17 | 3.35E-13 | 1.45E-12 |
| RP11-697M17.2 | 2.48 | 2.72 | 3.82E-13 | 1.65E-12 |
| LINC00601 | 4.02 | 4.05 | 3.85E-13 | 1.66E-12 |
| CHODL-AS1 | 3.29 | 2.93 | 3.90E-13 | 1.69E-12 |
| RP11-221N13.3 | 3.35 | 5.20 | 4.15E-13 | 1.79E-12 |
| RP13-192B19.2 | 3.20 | 2.97 | 4.30E-13 | 1.86E-12 |
| RP11-94M14.2 | 4.15 | 3.47 | 4.34E-13 | 1.87E-12 |
| RP11-314D7.1 | 4.05 | 3.02 | 4.39E-13 | 1.89E-12 |
| RP11-687D19.1 | -2.65 | 2.49 | 4.54E-13 | 1.95E-12 |
| LINC02197 | -2.57 | 2.92 | 4.56E-13 | 1.96E-12 |
| SPATA3-AS1 | 2.32 | 3.93 | 4.58E-13 | 1.96E-12 |
| CTD-2535I10.1 | 5.14 | 4.36 | 4.58E-13 | 1.97E-12 |
| LINC01269 | 2.65 | 6.05 | 4.82E-13 | 2.06E-12 |
| RP11-44F14.2 | 2.09 | 7.45 | 4.86E-13 | 2.08E-12 |
| RP11-538D16.3 | 2.09 | 4.45 | 4.86E-13 | 2.08E-12 |
| RP11-218I7.2 | 3.22 | 2.53 | 5.04E-13 | 2.15E-12 |
| LINC01979 | 2.59 | 5.52 | 5.32E-13 | 2.27E-12 |
| RP11-44N12.5 | 2.25 | 4.39 | 5.37E-13 | 2.29E-12 |
| LINC01587 | 3.05 | 4.81 | 5.44E-13 | 2.32E-12 |
| GS1-24F4.2 | 2.64 | 4.27 | 5.63E-13 | 2.40E-12 |
| AF121898.3 | 4.03 | 3.00 | 5.85E-13 | 2.49E-12 |
| KCNIP4-IT1 | 3.74 | 2.76 | 6.20E-13 | 2.63E-12 |
| RP11-308B16.2 | 6.18 | 4.80 | 6.27E-13 | 2.66E-12 |
| CTD-2251F13.1 | 3.92 | 3.35 | 6.41E-13 | 2.72E-12 |
| RP11-337N6.2 | 2.22 | 5.32 | 6.53E-13 | 2.77E-12 |
| RP4-737E23.2 | 3.40 | 8.24 | 6.58E-13 | 2.79E-12 |
| AC079135.1 | 3.85 | 2.89 | 6.78E-13 | 2.86E-12 |
| RP11-647P12.1 | -2.20 | 2.63 | 7.35E-13 | 3.10E-12 |
| CTD-2162K18.4 | 2.81 | 5.59 | 7.47E-13 | 3.14E-12 |
| CDKN2A-AS1 | 3.21 | 3.93 | 7.52E-13 | 3.16E-12 |
| CASC21 | 2.14 | 4.65 | 8.15E-13 | 3.42E-12 |
| RP11-54O7.16 | 2.37 | 3.95 | 8.34E-13 | 3.49E-12 |
| RP11-1070N10.7 | 4.67 | 4.58 | 8.39E-13 | 3.51E-12 |
| RP11-3B12.2 | 2.92 | 3.64 | 8.75E-13 | 3.66E-12 |
| CTC-353G13.1 | 5.04 | 4.07 | 8.77E-13 | 3.67E-12 |
| RP11-1070N10.5 | 4.56 | 5.23 | 8.80E-13 | 3.68E-12 |
| OTX2-AS1 | 4.05 | 4.36 | 9.03E-13 | 3.78E-12 |
| CTC-492K19.7 | 2.33 | 3.21 | 9.59E-13 | 4.00E-12 |
| AC067956.1 | 2.31 | 3.31 | 1.02E-12 | 4.27E-12 |
| RP11-675F6.4 | 4.02 | 4.35 | 1.04E-12 | 4.31E-12 |
| RP11-190J1.3 | 4.90 | 4.40 | 1.05E-12 | 4.36E-12 |
| AC025016.1 | 5.51 | 3.86 | 1.08E-12 | 4.49E-12 |
| RP11-167H9.4 | 4.29 | 4.31 | 1.14E-12 | 4.72E-12 |
| MYHAS | 2.17 | 4.23 | 1.18E-12 | 4.89E-12 |
| RP11-401O9.3 | 3.29 | 4.15 | 1.19E-12 | 4.93E-12 |
| RP11-730G20.1 | 3.23 | 2.64 | 1.30E-12 | 5.38E-12 |
| RP11-123K19.1 | 3.94 | 3.91 | 1.36E-12 | 5.63E-12 |
| RP1-35C21.2 | 3.82 | 4.03 | 1.44E-12 | 5.95E-12 |
| RP11-484N16.1 | 2.74 | 4.47 | 1.49E-12 | 6.14E-12 |
| RP11-114H23.1 | 2.96 | 5.51 | 1.53E-12 | 6.30E-12 |
| LINC01093 | -2.02 | 3.03 | 1.57E-12 | 6.46E-12 |
| AC013463.2 | 3.01 | 7.51 | 1.58E-12 | 6.50E-12 |
| TCF4-AS1 | 3.23 | 4.89 | 1.60E-12 | 6.54E-12 |
| FGF10-AS1 | -2.35 | 2.94 | 1.65E-12 | 6.77E-12 |
| LINC01964 | 4.69 | 4.37 | 1.69E-12 | 6.90E-12 |
| RP11-144A16.8 | -2.59 | 3.18 | 1.70E-12 | 6.96E-12 |
| RP11-352B15.2 | 5.27 | 3.93 | 1.72E-12 | 7.04E-12 |
| CTD-2265O21.3 | 3.48 | 2.69 | 1.89E-12 | 7.69E-12 |
| RP4-736L20.3 | 2.15 | 2.85 | 1.95E-12 | 7.94E-12 |
| FAM230C | 6.71 | 4.99 | 2.21E-12 | 8.97E-12 |
| LINC01995 | 4.24 | 4.23 | 2.22E-12 | 9.03E-12 |
| TDRG1 | 5.46 | 4.45 | 2.40E-12 | 9.71E-12 |
| RP11-88H10.3 | 5.05 | 3.53 | 2.41E-12 | 9.75E-12 |
| LINC01494 | 2.83 | 2.69 | 2.47E-12 | 9.98E-12 |
| RP11-502N13.2 | 2.18 | 3.23 | 2.48E-12 | 1.00E-11 |
| RP11-746B8.1 | 3.05 | 2.84 | 2.55E-12 | 1.03E-11 |
| RP4-781K5.4 | 2.47 | 5.47 | 2.59E-12 | 1.04E-11 |
| LINC00165 | -2.51 | 3.78 | 2.66E-12 | 1.07E-11 |
| FLJ16779 | 2.68 | 6.22 | 2.68E-12 | 1.08E-11 |
| RP11-488I20.8 | 5.78 | 4.65 | 2.92E-12 | 1.17E-11 |
| LINC00659 | 3.30 | 4.49 | 3.02E-12 | 1.21E-11 |
| LINC00525 | 2.27 | 5.28 | 3.04E-12 | 1.21E-11 |
| RP11-817J15.2 | 4.37 | 6.11 | 3.09E-12 | 1.23E-11 |
| RP11-338L18.1 | 3.44 | 2.60 | 3.16E-12 | 1.26E-11 |
| RP11-260A9.6 | 6.39 | 4.52 | 3.27E-12 | 1.30E-11 |
| LINC02128 | 3.76 | 3.89 | 3.36E-12 | 1.34E-11 |
| CTA-520D8.2 | 4.38 | 4.64 | 3.40E-12 | 1.35E-11 |
| LINC01250 | 3.38 | 3.54 | 3.50E-12 | 1.39E-11 |
| RP11-309M7.1 | 4.47 | 3.38 | 3.54E-12 | 1.41E-11 |
| RP11-297D21.2 | 2.39 | 2.59 | 3.70E-12 | 1.47E-11 |
| KB-1615E4.2 | 2.98 | 3.92 | 3.91E-12 | 1.55E-11 |
| RP4-715N11.2 | 3.18 | 3.26 | 4.17E-12 | 1.65E-11 |
| RP11-430H10.3 | 4.47 | 3.47 | 4.18E-12 | 1.65E-11 |
| RP11-88H10.2 | 4.95 | 3.71 | 4.45E-12 | 1.75E-11 |
| RP4-784A16.4 | 2.53 | 2.82 | 4.47E-12 | 1.76E-11 |
| RP11-8P13.5 | 2.07 | 4.37 | 4.84E-12 | 1.91E-11 |
| RP5-1121A15.3 | 4.42 | 3.60 | 5.00E-12 | 1.97E-11 |
| RP11-269F21.3 | 5.03 | 4.92 | 5.19E-12 | 2.04E-11 |
| RP3-495K2.2 | 2.75 | 3.26 | 5.42E-12 | 2.13E-11 |
| RP11-280O1.2 | -2.02 | 3.63 | 5.60E-12 | 2.20E-11 |
| RP4-806M20.3 | 2.66 | 3.86 | 5.67E-12 | 2.22E-11 |
| LINC01910 | 3.03 | 3.76 | 5.98E-12 | 2.34E-11 |
| LEMD1-AS1 | 2.03 | 4.35 | 6.26E-12 | 2.45E-11 |
| LINC00302 | 4.67 | 3.14 | 6.53E-12 | 2.55E-11 |
| RP11-376O6.2 | 2.46 | 2.61 | 6.73E-12 | 2.62E-11 |
| SSTR5-AS1 | 4.18 | 5.09 | 7.07E-12 | 2.75E-11 |
| RP11-1042B17.3 | 3.74 | 4.13 | 7.28E-12 | 2.82E-11 |
| CTA-126B4.7 | 2.26 | 5.18 | 7.51E-12 | 2.91E-11 |
| LINC01687 | 5.45 | 3.83 | 7.52E-12 | 2.91E-11 |
| RP11-831A10.2 | 5.13 | 3.77 | 7.94E-12 | 3.07E-11 |
| CTD-2339L15.3 | 2.22 | 2.57 | 7.96E-12 | 3.08E-11 |
| RP11-838N2.5 | 3.59 | 5.19 | 8.70E-12 | 3.36E-11 |
| RP11-456H18.2 | 2.15 | 5.55 | 8.89E-12 | 3.43E-11 |
| PKIA-AS1 | 2.00 | 4.58 | 9.01E-12 | 3.47E-11 |
| LVCAT1 | 3.55 | 5.46 | 9.45E-12 | 3.63E-11 |
| LINC01224 | 3.46 | 8.16 | 9.45E-12 | 3.63E-11 |
| RP11-14C10.5 | 2.43 | 2.82 | 9.46E-12 | 3.64E-11 |
| CTD-2616J11.16 | 2.63 | 3.30 | 9.72E-12 | 3.73E-11 |
| FLJ36000 | 6.61 | 5.29 | 9.79E-12 | 3.76E-11 |
| C15orf59-AS1 | 3.47 | 5.25 | 1.00E-11 | 3.85E-11 |
| RP11-90P5.2 | 2.66 | 3.76 | 1.01E-11 | 3.88E-11 |
| RP11-503C24.6 | 4.03 | 4.50 | 1.01E-11 | 3.88E-11 |
| LINC01546 | 2.38 | 4.43 | 1.08E-11 | 4.14E-11 |
| RP11-443C10.1 | 2.27 | 3.43 | 1.10E-11 | 4.21E-11 |
| RP11-264E20.2 | 2.09 | 2.77 | 1.12E-11 | 4.27E-11 |
| AJ003147.9 | 3.62 | 2.92 | 1.12E-11 | 4.28E-11 |
| AF015262.2 | 3.46 | 3.18 | 1.13E-11 | 4.32E-11 |
| LINC00051 | 4.71 | 4.26 | 1.19E-11 | 4.53E-11 |
| C5orf60 | 2.07 | 3.55 | 1.23E-11 | 4.68E-11 |
| RP11-104J23.1 | -2.29 | 2.54 | 1.32E-11 | 5.00E-11 |
| RP11-17E2.2 | 2.92 | 4.46 | 1.35E-11 | 5.10E-11 |
| AL121578.2 | 4.52 | 3.30 | 1.38E-11 | 5.22E-11 |
| RP11-625H11.2 | 4.50 | 3.70 | 1.45E-11 | 5.46E-11 |
| RP11-471M2.3 | 5.41 | 3.91 | 1.57E-11 | 5.91E-11 |
| RP11-806L2.5 | 2.06 | 2.77 | 1.58E-11 | 5.96E-11 |
| RP5-866L20.1 | 3.59 | 3.88 | 1.61E-11 | 6.05E-11 |
| RP1-313L4.3 | 2.33 | 4.55 | 1.63E-11 | 6.14E-11 |
| RP11-85G21.3 | -2.12 | 4.95 | 1.66E-11 | 6.22E-11 |
| RP11-568J23.4 | 2.16 | 2.66 | 1.67E-11 | 6.26E-11 |
| FER1L6-AS2 | 5.40 | 4.58 | 1.73E-11 | 6.48E-11 |
| RP11-15M15.2 | 2.36 | 2.79 | 1.76E-11 | 6.59E-11 |
| RP11-101E5.1 | -2.46 | 2.94 | 1.81E-11 | 6.78E-11 |
| OSTM1-AS1 | 4.65 | 3.84 | 1.85E-11 | 6.94E-11 |
| RP11-576D8.4 | 3.02 | 4.15 | 1.87E-11 | 6.98E-11 |
| RP11-290F24.3 | 2.58 | 3.56 | 1.94E-11 | 7.27E-11 |
| RP11-883A18.3 | 2.06 | 4.35 | 1.99E-11 | 7.43E-11 |
| LINC01491 | 4.45 | 3.23 | 2.00E-11 | 7.47E-11 |
| RP11-73M14.1 | 2.50 | 2.70 | 2.17E-11 | 8.10E-11 |
| RP5-827E24.1 | 3.58 | 3.61 | 2.19E-11 | 8.14E-11 |
| LINC01192 | 4.23 | 3.42 | 2.29E-11 | 8.53E-11 |
| LINC02167 | 7.97 | 6.44 | 2.43E-11 | 9.02E-11 |
| LINC01213 | 3.43 | 3.81 | 2.44E-11 | 9.04E-11 |
| RP11-46A10.2 | 2.02 | 3.76 | 2.46E-11 | 9.12E-11 |
| LVCAT5 | 6.22 | 4.65 | 2.48E-11 | 9.20E-11 |
| LINC02111 | 3.47 | 3.19 | 2.50E-11 | 9.25E-11 |
| RP1-269M15.3 | 5.12 | 5.78 | 2.60E-11 | 9.62E-11 |
| CH17-335B8.4 | 2.52 | 3.69 | 2.61E-11 | 9.63E-11 |
| RP11-700N1.1 | 2.80 | 3.53 | 2.67E-11 | 9.86E-11 |
| AC073316.2 | 2.07 | 3.95 | 2.77E-11 | 1.02E-10 |
| AC093627.7 | 4.19 | 3.53 | 2.78E-11 | 1.03E-10 |
| RP11-317N12.1 | 6.38 | 5.57 | 2.80E-11 | 1.03E-10 |
| RP11-161D15.1 | 3.58 | 3.18 | 2.83E-11 | 1.04E-10 |
| AC004158.3 | 4.38 | 4.28 | 3.20E-11 | 1.17E-10 |
| LINC00701 | 3.63 | 2.72 | 3.53E-11 | 1.29E-10 |
| RP11-416N4.1 | 3.23 | 2.99 | 3.84E-11 | 1.40E-10 |
| RP11-398B16.2 | 6.92 | 5.15 | 3.90E-11 | 1.42E-10 |
| FOXCUT | 2.44 | 4.37 | 3.92E-11 | 1.43E-10 |
| RP11-680F20.10 | 2.33 | 3.34 | 4.10E-11 | 1.49E-10 |
| RP4-799P18.2 | 2.71 | 4.48 | 4.26E-11 | 1.55E-10 |
| AC008278.3 | 3.74 | 3.34 | 4.29E-11 | 1.56E-10 |
| CTA-392E5.1 | 6.24 | 5.97 | 4.39E-11 | 1.59E-10 |
| LINC01370 | 7.56 | 6.02 | 4.50E-11 | 1.63E-10 |
| RP11-135A1.3 | 4.12 | 3.56 | 4.51E-11 | 1.63E-10 |
| RP3-333B15.5 | 2.88 | 3.04 | 4.54E-11 | 1.64E-10 |
| RP4-806M20.4 | 2.17 | 4.11 | 4.57E-11 | 1.66E-10 |
| RP11-802F5.1 | 4.34 | 3.90 | 4.63E-11 | 1.67E-10 |
| RP3-359N14.2 | 3.04 | 2.96 | 4.64E-11 | 1.68E-10 |
| ERVMER61-1 | 5.94 | 4.20 | 4.69E-11 | 1.69E-10 |
| RP11-184I16.4 | 3.67 | 4.81 | 4.83E-11 | 1.74E-10 |
| RP1-290I10.5 | 3.62 | 3.02 | 5.12E-11 | 1.84E-10 |
| LINC01511 | 5.03 | 5.75 | 5.33E-11 | 1.92E-10 |
| RP11-402J6.1 | 3.74 | 3.09 | 5.40E-11 | 1.94E-10 |
| RP5-916L7.2 | 3.13 | 3.22 | 5.41E-11 | 1.94E-10 |
| LINC01120 | 2.87 | 2.51 | 5.43E-11 | 1.95E-10 |
| RP11-148M9.1 | 3.43 | 2.53 | 5.44E-11 | 1.95E-10 |
| RP5-1029K10.2 | 2.38 | 3.72 | 5.62E-11 | 2.01E-10 |
| RP5-978I12.1 | -2.35 | 3.77 | 5.63E-11 | 2.02E-10 |
| RP11-689C9.1 | 2.69 | 3.71 | 5.73E-11 | 2.05E-10 |
| LINC01535 | 2.01 | 6.50 | 5.84E-11 | 2.08E-10 |
| RP11-817J15.3 | 4.09 | 5.51 | 5.89E-11 | 2.10E-10 |
| MIR5689HG | 2.82 | 2.80 | 6.18E-11 | 2.20E-10 |
| CTA-392C11.1 | 8.87 | 7.20 | 6.20E-11 | 2.21E-10 |
| RP11-454P21.1 | 3.60 | 4.01 | 6.20E-11 | 2.21E-10 |
| RP4-797C5.2 | 2.93 | 2.85 | 6.23E-11 | 2.22E-10 |
| AC073957.15 | 3.57 | 4.34 | 6.24E-11 | 2.22E-10 |
| RP11-115C10.1 | 3.31 | 4.57 | 6.55E-11 | 2.33E-10 |
| RP11-282A11.3 | 3.24 | 5.04 | 6.96E-11 | 2.47E-10 |
| RP11-503C24.2 | 4.19 | 4.37 | 7.05E-11 | 2.50E-10 |
| LINC01143 | 3.18 | 4.21 | 7.15E-11 | 2.53E-10 |
| RP11-126K1.9 | 2.21 | 2.66 | 7.19E-11 | 2.55E-10 |
| RP11-54O7.18 | 2.27 | 4.42 | 7.21E-11 | 2.55E-10 |
| AC096669.1 | 4.28 | 2.94 | 7.74E-11 | 2.73E-10 |
| CTD-2354A18.1 | 6.20 | 6.97 | 7.99E-11 | 2.82E-10 |
| RP11-346D14.1 | 3.10 | 3.13 | 8.41E-11 | 2.96E-10 |
| RP11-1085N6.3 | 3.75 | 3.16 | 8.89E-11 | 3.12E-10 |
| LINC00381 | 2.70 | 3.36 | 8.94E-11 | 3.14E-10 |
| LINC01344 | 2.41 | 4.10 | 9.28E-11 | 3.25E-10 |
| LINC00698 | 2.60 | 3.11 | 9.31E-11 | 3.26E-10 |
| U95743.1 | 4.33 | 3.09 | 9.40E-11 | 3.28E-10 |
| RP11-890B15.2 | 2.49 | 5.16 | 9.40E-11 | 3.28E-10 |
| RP11-438D14.3 | 3.73 | 3.00 | 9.68E-11 | 3.38E-10 |
| LINC01920 | 3.61 | 2.84 | 9.70E-11 | 3.38E-10 |
| RP1-140J1.1 | 3.77 | 4.55 | 9.81E-11 | 3.42E-10 |
| RBAKDN | 2.89 | 4.37 | 9.83E-11 | 3.42E-10 |
| RP1-90K10.4 | 3.55 | 3.26 | 1.03E-10 | 3.59E-10 |
| DDR1-AS1 | 2.21 | 3.17 | 1.04E-10 | 3.63E-10 |
| RP11-615J4.3 | 6.66 | 4.83 | 1.06E-10 | 3.69E-10 |
| LINC01405 | 3.71 | 3.32 | 1.09E-10 | 3.79E-10 |
| AJ003147.8 | 3.55 | 2.92 | 1.20E-10 | 4.14E-10 |
| RP11-110H1.8 | 4.26 | 4.43 | 1.20E-10 | 4.15E-10 |
| RP11-1263C18.1 | 4.22 | 3.29 | 1.22E-10 | 4.22E-10 |
| AE000662.93 | 2.04 | 3.55 | 1.24E-10 | 4.29E-10 |
| RP5-928E24.2 | 2.79 | 2.57 | 1.28E-10 | 4.42E-10 |
| B3GALT5-AS1 | 3.19 | 6.30 | 1.31E-10 | 4.49E-10 |
| RP11-501C14.5 | 4.37 | 3.20 | 1.32E-10 | 4.53E-10 |
| RP11-68I3.7 | 2.62 | 2.66 | 1.33E-10 | 4.59E-10 |
| AC024592.9 | 2.18 | 5.01 | 1.35E-10 | 4.62E-10 |
| CLSTN2-AS1 | 2.49 | 3.10 | 1.36E-10 | 4.67E-10 |
| RP11-496N12.6 | 2.71 | 5.35 | 1.36E-10 | 4.68E-10 |
| RP11-488I20.9 | 6.80 | 4.98 | 1.39E-10 | 4.75E-10 |
| AC006946.16 | 2.30 | 6.27 | 1.40E-10 | 4.79E-10 |
| SCHLAP1 | 5.93 | 4.19 | 1.45E-10 | 4.94E-10 |
| AC010967.2 | 4.31 | 3.15 | 1.45E-10 | 4.95E-10 |
| RP4-651E10.4 | 2.23 | 3.63 | 1.47E-10 | 5.00E-10 |
| CTD-2128A3.2 | 3.28 | 3.22 | 1.47E-10 | 5.01E-10 |
| RP11-785D18.3 | 2.94 | 5.92 | 1.49E-10 | 5.09E-10 |
| H19 | 3.40 | 13.95 | 1.51E-10 | 5.12E-10 |
| LNX1-AS1 | 3.23 | 2.87 | 1.53E-10 | 5.19E-10 |
| RP11-314P15.2 | 3.52 | 2.57 | 1.54E-10 | 5.23E-10 |
| TTTY16 | -2.14 | 3.22 | 1.54E-10 | 5.24E-10 |
| RP4-761J14.9 | 2.45 | 2.60 | 1.58E-10 | 5.37E-10 |
| RP11-353N14.3 | 3.00 | 2.57 | 1.59E-10 | 5.39E-10 |
| RP11-722M1.1 | 4.81 | 3.94 | 1.60E-10 | 5.42E-10 |
| RP11-324L17.1 | 2.64 | 3.75 | 1.60E-10 | 5.44E-10 |
| RP11-625L16.1 | -2.18 | 2.79 | 1.66E-10 | 5.61E-10 |
| RP11-538I12.3 | 3.42 | 3.71 | 1.66E-10 | 5.61E-10 |
| AP000473.6 | 2.94 | 2.86 | 1.81E-10 | 6.11E-10 |
| AC007365.1 | 2.08 | 4.20 | 1.85E-10 | 6.26E-10 |
| RP1-170O19.24 | 2.22 | 2.84 | 1.90E-10 | 6.42E-10 |
| RP11-145G20.1 | 6.55 | 5.07 | 1.95E-10 | 6.59E-10 |
| CYYR1-AS1 | 2.33 | 4.53 | 2.13E-10 | 7.15E-10 |
| RP11-324D17.2 | 2.82 | 3.10 | 2.19E-10 | 7.34E-10 |
| RP5-1185I7.1 | 2.54 | 7.44 | 2.28E-10 | 7.64E-10 |
| LINC00319 | 2.60 | 2.96 | 2.34E-10 | 7.84E-10 |
| AC087491.2 | 2.35 | 6.67 | 2.34E-10 | 7.84E-10 |
| RP11-398J5.1 | 4.98 | 3.62 | 2.36E-10 | 7.89E-10 |
| AC006548.28 | 2.45 | 5.39 | 2.49E-10 | 8.32E-10 |
| AC078842.3 | 3.21 | 2.59 | 2.57E-10 | 8.57E-10 |
| LINC01543 | 3.42 | 3.54 | 2.61E-10 | 8.72E-10 |
| RP11-140A10.3 | 2.46 | 3.51 | 2.64E-10 | 8.81E-10 |
| ATP13A5-AS1 | 3.06 | 2.85 | 2.66E-10 | 8.88E-10 |
| RP11-78A19.4 | 3.07 | 3.19 | 2.71E-10 | 9.04E-10 |
| AC010745.2 | 4.16 | 2.98 | 2.73E-10 | 9.08E-10 |
| RP11-749H20.1 | 4.27 | 3.09 | 3.05E-10 | 1.01E-09 |
| AE000661.50 | 2.56 | 3.76 | 3.05E-10 | 1.01E-09 |
| RP11-279N8.1 | 2.75 | 3.27 | 3.07E-10 | 1.02E-09 |
| RP11-478P10.1 | 2.70 | 2.49 | 3.08E-10 | 1.02E-09 |
| LINC00682 | 5.09 | 3.68 | 3.09E-10 | 1.02E-09 |
| LINC00460 | 2.95 | 6.99 | 3.16E-10 | 1.05E-09 |
| LINC02071 | 2.72 | 2.56 | 3.28E-10 | 1.08E-09 |
| AC012363.4 | 4.80 | 3.65 | 3.31E-10 | 1.09E-09 |
| RP11-109D24.1 | 2.66 | 2.53 | 3.32E-10 | 1.10E-09 |
| CTB-178M22.1 | 2.82 | 2.65 | 3.35E-10 | 1.11E-09 |
| LINC00113 | -2.10 | 2.70 | 3.61E-10 | 1.19E-09 |
| RP11-119K6.6 | 2.13 | 2.64 | 4.05E-10 | 1.33E-09 |
| RP11-254I22.2 | 2.90 | 2.77 | 4.06E-10 | 1.33E-09 |
| RP11-565A3.2 | 5.95 | 4.05 | 4.11E-10 | 1.35E-09 |
| RP11-320M16.2 | 3.12 | 3.35 | 4.13E-10 | 1.35E-09 |
| LHX5-AS1 | 3.40 | 2.62 | 4.14E-10 | 1.36E-09 |
| RP11-435D7.3 | 2.75 | 2.79 | 4.65E-10 | 1.52E-09 |
| RP11-334G22.1 | 2.46 | 2.60 | 4.67E-10 | 1.53E-09 |
| LINC00648 | 3.39 | 7.23 | 4.80E-10 | 1.56E-09 |
| RP1-140K8.1 | -2.09 | 2.49 | 5.00E-10 | 1.63E-09 |
| LINC01289 | 2.87 | 2.49 | 5.27E-10 | 1.71E-09 |
| AC093843.1 | 2.84 | 2.82 | 5.50E-10 | 1.78E-09 |
| LINC01205 | 4.99 | 4.09 | 6.16E-10 | 1.99E-09 |
| AC106875.1 | 5.40 | 5.79 | 6.18E-10 | 1.99E-09 |
| LINC01616 | -2.56 | 3.12 | 6.26E-10 | 2.02E-09 |
| CTD-2210P24.2 | 4.66 | 3.30 | 6.52E-10 | 2.10E-09 |
| RP11-227H15.4 | 3.85 | 4.85 | 6.78E-10 | 2.18E-09 |
| RP11-863K10.2 | 3.75 | 3.38 | 6.79E-10 | 2.18E-09 |
| RP11-626P14.2 | 3.86 | 3.07 | 7.00E-10 | 2.25E-09 |
| LINC01395 | 2.81 | 3.77 | 7.12E-10 | 2.28E-09 |
| RP11-12K11.2 | 3.69 | 4.45 | 7.35E-10 | 2.35E-09 |
| LINC01850 | 3.12 | 2.93 | 7.51E-10 | 2.40E-09 |
| AC008060.7 | 4.59 | 3.56 | 7.56E-10 | 2.42E-09 |
| AC112721.1 | 2.08 | 3.84 | 7.57E-10 | 2.42E-09 |
| RP11-406A9.2 | 4.35 | 4.77 | 7.79E-10 | 2.49E-09 |
| CTC-551A13.1 | 2.09 | 3.37 | 8.00E-10 | 2.55E-09 |
| RP11-108K14.12 | 2.47 | 4.17 | 8.07E-10 | 2.57E-09 |
| CACNA2D3-AS1 | 2.59 | 3.49 | 8.26E-10 | 2.63E-09 |
| LINC01139 | 2.90 | 8.54 | 8.29E-10 | 2.64E-09 |
| AC018685.1 | 3.58 | 2.90 | 8.36E-10 | 2.66E-09 |
| RP11-663N22.1 | 2.16 | 4.56 | 8.43E-10 | 2.68E-09 |
| LINC01475 | 3.81 | 3.62 | 8.52E-10 | 2.71E-09 |
| KB-1991G8.1 | 2.10 | 5.08 | 8.55E-10 | 2.71E-09 |
| MTUS2-AS1 | 2.13 | 3.51 | 8.69E-10 | 2.76E-09 |
| TBX18-AS1 | 2.69 | 3.34 | 8.81E-10 | 2.79E-09 |
| AC003092.1 | 4.16 | 4.77 | 8.82E-10 | 2.79E-09 |
| RP11-1085N6.5 | 3.24 | 2.55 | 8.89E-10 | 2.81E-09 |
| RP11-332K15.1 | 3.44 | 3.67 | 9.13E-10 | 2.89E-09 |
| RP11-418I22.2 | -2.15 | 2.54 | 9.22E-10 | 2.92E-09 |
| RP11-80F22.15 | 4.67 | 3.16 | 9.24E-10 | 2.92E-09 |
| LINC01929 | 2.23 | 7.27 | 9.28E-10 | 2.93E-09 |
| RP11-1109M24.5 | 6.56 | 5.31 | 9.33E-10 | 2.95E-09 |
| RP11-485F13.1 | 5.72 | 4.35 | 9.52E-10 | 3.01E-09 |
| RP11-383J24.1 | 3.00 | 3.69 | 9.55E-10 | 3.01E-09 |
| AC012354.6 | 3.12 | 3.95 | 1.09E-09 | 3.42E-09 |
| RP11-554D15.3 | 3.31 | 3.56 | 1.20E-09 | 3.75E-09 |
| RP11-552M14.1 | 4.02 | 2.87 | 1.21E-09 | 3.79E-09 |
| RP11-809H16.5 | 3.79 | 3.05 | 1.26E-09 | 3.95E-09 |
| RP11-61J19.2 | 2.88 | 3.18 | 1.29E-09 | 4.03E-09 |
| RP11-172F10.1 | 2.86 | 3.25 | 1.36E-09 | 4.22E-09 |
| LINC01667 | 5.94 | 5.91 | 1.36E-09 | 4.23E-09 |
| RP11-568A7.2 | -2.24 | 2.70 | 1.40E-09 | 4.35E-09 |
| RP11-548L20.1 | 5.95 | 4.14 | 1.41E-09 | 4.39E-09 |
| SLC7A11-AS1 | 2.40 | 6.41 | 1.41E-09 | 4.39E-09 |
| RP5-912I13.1 | 5.19 | 3.63 | 1.46E-09 | 4.51E-09 |
| WFDC21P | 2.06 | 10.99 | 1.50E-09 | 4.64E-09 |
| LINC00705 | 2.46 | 3.68 | 1.52E-09 | 4.69E-09 |
| PCAT5 | 3.19 | 3.26 | 1.53E-09 | 4.73E-09 |
| RP11-390F4.6 | 2.44 | 4.74 | 1.54E-09 | 4.75E-09 |
| LARS2-AS1 | 2.04 | 3.30 | 1.57E-09 | 4.83E-09 |
| LINC01470 | 2.81 | 2.83 | 1.60E-09 | 4.93E-09 |
| AC074389.5 | 4.95 | 5.03 | 1.66E-09 | 5.12E-09 |
| LINC01254 | 3.50 | 3.99 | 1.68E-09 | 5.17E-09 |
| LINC00556 | 2.90 | 2.77 | 1.77E-09 | 5.44E-09 |
| LINC01160 | 2.18 | 6.83 | 1.78E-09 | 5.45E-09 |
| CTD-2306M5.1 | 3.26 | 2.55 | 1.81E-09 | 5.54E-09 |
| RP11-170M17.1 | 2.98 | 3.23 | 1.87E-09 | 5.72E-09 |
| LINC00308 | 4.07 | 2.83 | 1.92E-09 | 5.85E-09 |
| RP11-497G19.7 | 2.67 | 3.92 | 1.99E-09 | 6.07E-09 |
| C3orf67-AS1 | 2.62 | 2.46 | 2.04E-09 | 6.22E-09 |
| RP11-326N17.2 | 2.25 | 3.11 | 2.04E-09 | 6.22E-09 |
| RP11-91H12.3 | 3.60 | 2.68 | 2.05E-09 | 6.25E-09 |
| CTC-458G6.4 | 5.31 | 3.90 | 2.07E-09 | 6.29E-09 |
| PLUT | 4.02 | 2.87 | 2.27E-09 | 6.86E-09 |
| RP11-804N13.1 | 3.88 | 4.44 | 2.28E-09 | 6.89E-09 |
| EGLN3-AS1 | 3.42 | 2.86 | 2.28E-09 | 6.89E-09 |
| LINC00383 | 5.61 | 3.88 | 2.29E-09 | 6.90E-09 |
| RP1-40E16.9 | 6.18 | 5.37 | 2.34E-09 | 7.07E-09 |
| RP11-221N13.4 | 3.99 | 2.99 | 2.38E-09 | 7.18E-09 |
| RP11-275I4.2 | 2.05 | 3.71 | 2.46E-09 | 7.43E-09 |
| LINC01162 | 3.99 | 2.85 | 2.55E-09 | 7.68E-09 |
| RP4-753M9.1 | 3.19 | 2.79 | 2.56E-09 | 7.71E-09 |
| RP4-738P15.1 | 2.93 | 4.07 | 2.64E-09 | 7.93E-09 |
| RP11-269F21.2 | 3.86 | 3.06 | 2.68E-09 | 8.06E-09 |
| FGF12-AS3 | 2.76 | 3.17 | 2.72E-09 | 8.18E-09 |
| RP11-649A16.1 | 4.61 | 3.25 | 2.76E-09 | 8.28E-09 |
| AC019185.4 | 2.92 | 2.76 | 2.81E-09 | 8.44E-09 |
| ADARB2-AS1 | 2.77 | 4.24 | 2.88E-09 | 8.63E-09 |
| RP11-367F23.1 | 2.36 | 4.51 | 2.93E-09 | 8.76E-09 |
| RP11-115I9.1 | 4.22 | 2.91 | 3.05E-09 | 9.11E-09 |
| PAUPAR | 3.46 | 2.55 | 3.19E-09 | 9.52E-09 |
| RP11-560A15.3 | 3.42 | 3.00 | 3.20E-09 | 9.55E-09 |
| AC084193.1 | 5.20 | 3.51 | 3.28E-09 | 9.78E-09 |
| RP11-65M17.1 | 3.01 | 2.91 | 3.41E-09 | 1.02E-08 |
| RP11-290L1.5 | 3.40 | 2.68 | 3.60E-09 | 1.07E-08 |
| LINC02005 | 3.11 | 4.24 | 3.64E-09 | 1.08E-08 |
| CTC-261N6.1 | 4.19 | 2.88 | 3.69E-09 | 1.10E-08 |
| LINC02048 | 2.41 | 3.23 | 3.88E-09 | 1.15E-08 |
| RP11-63P12.7 | 2.72 | 2.60 | 3.97E-09 | 1.17E-08 |
| RP11-488P3.1 | 2.35 | 7.25 | 4.00E-09 | 1.18E-08 |
| XXbac-BPG308K3.5 | 2.12 | 3.88 | 4.01E-09 | 1.18E-08 |
| LINC01608 | 5.96 | 4.16 | 4.03E-09 | 1.19E-08 |
| RP5-1024C24.1 | 3.31 | 3.99 | 4.07E-09 | 1.20E-08 |
| RP13-60M5.2 | 5.25 | 3.54 | 4.19E-09 | 1.23E-08 |
| bP-2171C21.3 | 3.85 | 3.45 | 4.54E-09 | 1.33E-08 |
| CTB-107G13.1 | 3.00 | 5.93 | 4.63E-09 | 1.36E-08 |
| MYCNUT | 4.75 | 3.34 | 4.69E-09 | 1.37E-08 |
| RP11-103J17.2 | 5.65 | 5.06 | 4.81E-09 | 1.41E-08 |
| XX-C2158C6.3 | 2.31 | 2.90 | 4.89E-09 | 1.43E-08 |
| RP11-114H23.2 | 2.36 | 4.32 | 4.97E-09 | 1.45E-08 |
| LINC02170 | 2.21 | 5.22 | 4.98E-09 | 1.45E-08 |
| RP11-196H14.2 | 2.09 | 2.53 | 5.09E-09 | 1.48E-08 |
| LINC01098 | 3.03 | 3.16 | 5.31E-09 | 1.55E-08 |
| GS1-72M22.1 | 3.56 | 5.21 | 5.33E-09 | 1.55E-08 |
| RP11-335E6.3 | 2.72 | 2.51 | 5.38E-09 | 1.56E-08 |
| RP11-237N19.3 | 2.68 | 2.90 | 5.41E-09 | 1.57E-08 |
| AC128709.4 | 2.27 | 6.14 | 5.45E-09 | 1.58E-08 |
| LINC01887 | 2.20 | 3.24 | 5.62E-09 | 1.63E-08 |
| ELOVL2-AS1 | 2.84 | 3.31 | 5.64E-09 | 1.64E-08 |
| RP11-180C1.1 | 5.34 | 3.90 | 5.69E-09 | 1.65E-08 |
| CTD-2374C24.1 | 4.52 | 3.46 | 5.81E-09 | 1.69E-08 |
| RP11-392O17.1 | 3.71 | 5.65 | 5.82E-09 | 1.69E-08 |
| FGF12-AS2 | 2.47 | 3.69 | 6.09E-09 | 1.76E-08 |
| RP11-805L22.3 | 3.56 | 2.59 | 6.18E-09 | 1.79E-08 |
| RP11-120I21.2 | 4.69 | 3.58 | 6.25E-09 | 1.81E-08 |
| RP11-136B18.2 | 3.33 | 2.58 | 6.43E-09 | 1.86E-08 |
| AC003986.7 | 2.63 | 2.73 | 6.45E-09 | 1.86E-08 |
| LINC02105 | 3.01 | 2.88 | 6.62E-09 | 1.91E-08 |
| RP11-202K23.1 | 5.07 | 3.40 | 6.67E-09 | 1.92E-08 |
| RP11-332J15.2 | 4.61 | 3.41 | 6.68E-09 | 1.92E-08 |
| LINC01792 | 2.70 | 3.19 | 6.81E-09 | 1.96E-08 |
| BCAR4 | 4.36 | 4.48 | 7.20E-09 | 2.07E-08 |
| LINC00973 | 3.91 | 5.15 | 7.70E-09 | 2.21E-08 |
| RP11-384F7.1 | 3.96 | 3.61 | 7.96E-09 | 2.28E-08 |
| RP11-1114I9.1 | 3.87 | 2.90 | 8.43E-09 | 2.41E-08 |
| RP11-11N5.1 | 4.08 | 4.49 | 8.69E-09 | 2.48E-08 |
| LINC01343 | 3.60 | 3.46 | 8.72E-09 | 2.49E-08 |
| RP11-16L21.7 | 2.13 | 3.44 | 8.97E-09 | 2.55E-08 |
| MIR3681HG | 2.24 | 4.88 | 9.17E-09 | 2.61E-08 |
| RP11-272B17.2 | 3.82 | 2.87 | 9.28E-09 | 2.63E-08 |
| RP11-502M1.2 | 2.82 | 3.42 | 9.35E-09 | 2.65E-08 |
| LINC01666 | 2.88 | 3.65 | 9.57E-09 | 2.71E-08 |
| RP11-348J12.5 | 2.53 | 3.18 | 9.90E-09 | 2.80E-08 |
| AC092625.1 | 3.69 | 2.72 | 1.02E-08 | 2.88E-08 |
| RP11-15M15.1 | 2.25 | 2.53 | 1.03E-08 | 2.90E-08 |
| AC000032.2 | 3.92 | 3.77 | 1.04E-08 | 2.94E-08 |
| RP11-578B16.1 | 3.68 | 3.29 | 1.05E-08 | 2.97E-08 |
| RP11-401O9.4 | 2.41 | 4.39 | 1.06E-08 | 2.98E-08 |
| RP4-745K6.1 | 3.75 | 2.67 | 1.06E-08 | 2.99E-08 |
| RP11-268G12.3 | 3.03 | 3.88 | 1.07E-08 | 3.01E-08 |
| RP11-509A17.3 | 2.34 | 3.81 | 1.15E-08 | 3.23E-08 |
| CTB-1I21.1 | 3.97 | 3.70 | 1.16E-08 | 3.26E-08 |
| LINC01087 | 3.68 | 4.19 | 1.17E-08 | 3.28E-08 |
| RP11-128P17.4 | 4.05 | 3.76 | 1.19E-08 | 3.32E-08 |
| RP11-374A4.1 | 2.51 | 2.91 | 1.19E-08 | 3.33E-08 |
| RP11-322J23.1 | 3.65 | 3.59 | 1.24E-08 | 3.48E-08 |
| LINC01193 | 5.61 | 4.00 | 1.27E-08 | 3.54E-08 |
| LINC01251 | 2.87 | 3.65 | 1.27E-08 | 3.55E-08 |
| RP11-400D2.2 | 5.11 | 3.43 | 1.29E-08 | 3.59E-08 |
| RP11-419C23.1 | 2.40 | 3.32 | 1.33E-08 | 3.70E-08 |
| RP4-536B24.3 | 2.17 | 3.04 | 1.36E-08 | 3.79E-08 |
| RP11-315A17.1 | 6.20 | 5.79 | 1.37E-08 | 3.80E-08 |
| RP11-1081M5.1 | 3.50 | 4.78 | 1.37E-08 | 3.80E-08 |
| RP11-122C21.1 | 4.20 | 4.02 | 1.39E-08 | 3.87E-08 |
| LINC01258 | 2.67 | 3.51 | 1.51E-08 | 4.18E-08 |
| LA16c-352F7.1 | 2.08 | 3.62 | 1.52E-08 | 4.19E-08 |
| RP11-64C12.6 | 2.29 | 2.94 | 1.54E-08 | 4.27E-08 |
| CTB-147C22.9 | 2.92 | 2.95 | 1.55E-08 | 4.28E-08 |
| RP11-302L19.1 | 3.40 | 3.56 | 1.58E-08 | 4.36E-08 |
| LINC02152 | 4.10 | 3.96 | 1.61E-08 | 4.45E-08 |
| LINC01812 | 2.48 | 2.88 | 1.69E-08 | 4.65E-08 |
| LINC01036 | 3.35 | 3.65 | 1.69E-08 | 4.66E-08 |
| TTLL11-IT1 | 2.11 | 3.91 | 1.72E-08 | 4.72E-08 |
| AC137723.5 | 2.06 | 2.56 | 1.72E-08 | 4.73E-08 |
| RP11-395N3.1 | 2.20 | 3.92 | 1.73E-08 | 4.77E-08 |
| RP11-557H15.5 | 3.31 | 2.81 | 1.78E-08 | 4.88E-08 |
| RP11-438B23.2 | 2.59 | 4.76 | 1.79E-08 | 4.91E-08 |
| RP11-440G9.1 | 3.14 | 3.62 | 1.82E-08 | 4.98E-08 |
| LINC01602 | 4.11 | 6.26 | 1.92E-08 | 5.26E-08 |
| LINC01805 | 2.66 | 3.97 | 1.95E-08 | 5.33E-08 |
| RP11-21A7A.2 | 2.08 | 2.65 | 1.98E-08 | 5.40E-08 |
| RP11-22H5.2 | 2.30 | 3.11 | 2.14E-08 | 5.83E-08 |
| PLCH1-AS2 | 2.15 | 2.90 | 2.15E-08 | 5.85E-08 |
| AC005197.2 | 2.02 | 2.93 | 2.17E-08 | 5.89E-08 |
| RP11-434I12.3 | 3.66 | 6.67 | 2.19E-08 | 5.95E-08 |
| AC016710.1 | 5.32 | 3.66 | 2.37E-08 | 6.40E-08 |
| LINC01598 | 2.27 | 3.35 | 2.45E-08 | 6.60E-08 |
| AC098828.2 | 2.45 | 3.50 | 2.55E-08 | 6.87E-08 |
| KIRREL3-AS1 | 3.74 | 3.81 | 2.57E-08 | 6.93E-08 |
| RP11-474D1.3 | 5.89 | 5.85 | 2.60E-08 | 7.01E-08 |
| MIR4300HG | 3.24 | 3.43 | 2.62E-08 | 7.05E-08 |
| RP11-536I6.2 | 3.61 | 3.56 | 2.67E-08 | 7.18E-08 |
| CTD-2555A7.2 | 2.42 | 3.22 | 2.73E-08 | 7.32E-08 |
| AC015933.2 | 3.06 | 3.04 | 2.76E-08 | 7.41E-08 |
| LINC01647 | 3.80 | 3.61 | 2.87E-08 | 7.70E-08 |
| RP11-319F12.2 | 4.70 | 3.64 | 2.89E-08 | 7.75E-08 |
| RP11-94P11.4 | 3.57 | 2.67 | 2.92E-08 | 7.82E-08 |
| CTD-2008P7.1 | 2.23 | 3.28 | 3.04E-08 | 8.14E-08 |
| RP11-332J15.1 | 2.05 | 2.77 | 3.28E-08 | 8.74E-08 |
| RP11-109E24.1 | 2.04 | 3.27 | 3.42E-08 | 9.09E-08 |
| RP11-431M3.1 | 4.20 | 2.98 | 3.48E-08 | 9.25E-08 |
| LINC01707 | 2.67 | 2.51 | 3.49E-08 | 9.27E-08 |
| RP11-395D3.1 | 2.88 | 3.21 | 3.55E-08 | 9.43E-08 |
| ERVH48-1 | 3.06 | 7.35 | 3.56E-08 | 9.44E-08 |
| RP11-416N4.4 | 2.38 | 2.55 | 3.74E-08 | 9.91E-08 |
| AC004870.4 | 3.44 | 4.89 | 3.79E-08 | 1.01E-07 |
| RP11-326A13.1 | 2.56 | 2.67 | 3.95E-08 | 1.04E-07 |
| LINC01885 | 3.71 | 2.66 | 3.98E-08 | 1.05E-07 |
| AC027119.1 | 3.57 | 2.77 | 3.98E-08 | 1.05E-07 |
| LINC00851 | 3.32 | 2.72 | 4.13E-08 | 1.09E-07 |
| AC016730.1 | 4.59 | 3.35 | 4.21E-08 | 1.11E-07 |
| LINC00628 | 2.65 | 3.26 | 4.26E-08 | 1.12E-07 |
| RP3-400B16.3 | 3.95 | 3.50 | 4.38E-08 | 1.15E-07 |
| RP11-323C15.2 | 2.48 | 7.26 | 4.64E-08 | 1.22E-07 |
| LINC02188 | 2.35 | 6.57 | 4.75E-08 | 1.24E-07 |
| LINC02119 | 4.23 | 3.22 | 4.79E-08 | 1.25E-07 |
| RP6-159A1.3 | 2.55 | 4.47 | 4.88E-08 | 1.28E-07 |
| NRG1-IT1 | 3.83 | 2.81 | 4.97E-08 | 1.30E-07 |
| RP11-78L16.1 | 5.13 | 3.53 | 4.97E-08 | 1.30E-07 |
| RP11-776A13.3 | 4.43 | 3.12 | 5.09E-08 | 1.33E-07 |
| RP6-91H8.5 | 2.45 | 3.33 | 5.13E-08 | 1.34E-07 |
| CTD-2315E11.1 | 2.63 | 3.06 | 5.44E-08 | 1.41E-07 |
| RP11-35L17.3 | 2.43 | 2.62 | 5.53E-08 | 1.44E-07 |
| ST8SIA6-AS1 | 3.22 | 6.94 | 5.65E-08 | 1.47E-07 |
| LINC01173 | 2.72 | 2.51 | 5.72E-08 | 1.48E-07 |
| CTD-2384A14.1 | 5.17 | 4.92 | 5.77E-08 | 1.50E-07 |
| AC023481.1 | 2.30 | 2.67 | 5.82E-08 | 1.51E-07 |
| RP11-315F22.1 | 5.63 | 4.51 | 5.94E-08 | 1.54E-07 |
| PAQR9-AS1 | 2.54 | 3.34 | 6.04E-08 | 1.57E-07 |
| LINC01115 | 2.10 | 3.47 | 6.40E-08 | 1.65E-07 |
| RP11-126K15.1 | 2.50 | 2.59 | 6.43E-08 | 1.66E-07 |
| RP11-774D14.1 | 3.94 | 6.50 | 6.53E-08 | 1.68E-07 |
| LINC01411 | 3.24 | 2.91 | 6.62E-08 | 1.71E-07 |
| CTD-2320G14.2 | 2.31 | 2.62 | 6.65E-08 | 1.71E-07 |
| RP11-705O24.1 | 2.28 | 2.70 | 7.10E-08 | 1.83E-07 |
| RP11-479J7.2 | 2.21 | 2.81 | 7.13E-08 | 1.83E-07 |
| RP11-706C16.7 | 2.22 | 4.09 | 7.18E-08 | 1.84E-07 |
| AC009236.2 | 2.19 | 2.71 | 7.70E-08 | 1.97E-07 |
| LINC01181 | 2.03 | 3.00 | 7.90E-08 | 2.02E-07 |
| RP1-261G23.5 | 2.60 | 2.89 | 8.08E-08 | 2.07E-07 |
| RP11-567M16.2 | 2.21 | 3.84 | 8.15E-08 | 2.08E-07 |
| RP11-897M7.1 | 2.97 | 2.97 | 8.64E-08 | 2.20E-07 |
| AC133680.1 | 2.29 | 3.19 | 9.18E-08 | 2.34E-07 |
| RP11-430H10.4 | 3.57 | 2.84 | 9.19E-08 | 2.34E-07 |
| LINC01446 | 4.26 | 6.39 | 9.42E-08 | 2.39E-07 |
| RP11-631F7.1 | 2.75 | 3.06 | 9.67E-08 | 2.46E-07 |
| CTD-3007L5.1 | 5.93 | 4.07 | 9.96E-08 | 2.53E-07 |
| AC002463.3 | 4.16 | 3.26 | 1.02E-07 | 2.59E-07 |
| CTD-2620I22.3 | 2.73 | 4.18 | 1.03E-07 | 2.61E-07 |
| RP11-739N10.1 | 2.64 | 3.10 | 1.06E-07 | 2.67E-07 |
| LINC01326 | 4.54 | 3.30 | 1.06E-07 | 2.68E-07 |
| RP11-79E3.3 | 4.40 | 3.00 | 1.07E-07 | 2.70E-07 |
| RP11-136I14.5 | 3.79 | 2.95 | 1.10E-07 | 2.78E-07 |
| RP11-538D16.2 | 2.06 | 4.05 | 1.10E-07 | 2.78E-07 |
| RP5-1028L10.2 | 2.81 | 2.55 | 1.11E-07 | 2.79E-07 |
| RP11-347D21.2 | 3.23 | 2.53 | 1.17E-07 | 2.93E-07 |
| KB-68A7.2 | 3.56 | 4.30 | 1.19E-07 | 2.98E-07 |
| RP11-702F3.3 | 3.64 | 3.35 | 1.19E-07 | 2.99E-07 |
| RP11-713M6.2 | 3.65 | 2.73 | 1.20E-07 | 3.00E-07 |
| CTD-2587H24.5 | 2.35 | 3.62 | 1.22E-07 | 3.06E-07 |
| RP11-227D2.3 | 3.73 | 2.67 | 1.23E-07 | 3.08E-07 |
| AL078471.5 | 4.46 | 3.03 | 1.25E-07 | 3.13E-07 |
| RP11-95P13.2 | 4.41 | 3.22 | 1.26E-07 | 3.16E-07 |
| RP4-529N6.2 | 3.46 | 2.79 | 1.33E-07 | 3.33E-07 |
| RP11-16L14.2 | 2.93 | 2.57 | 1.35E-07 | 3.36E-07 |
| LINC01976 | 2.71 | 2.84 | 1.35E-07 | 3.37E-07 |
| CTD-2311B13.1 | 3.86 | 2.73 | 1.37E-07 | 3.41E-07 |
| RP11-234O6.2 | 5.61 | 4.09 | 1.38E-07 | 3.44E-07 |
| RP11-193H5.1 | 3.89 | 3.28 | 1.39E-07 | 3.45E-07 |
| AC141930.2 | 2.32 | 2.96 | 1.40E-07 | 3.49E-07 |
| LINC00303 | 3.12 | 4.19 | 1.41E-07 | 3.50E-07 |
| RP3-340N1.2 | 2.57 | 6.46 | 1.45E-07 | 3.61E-07 |
| RP11-490G2.2 | 3.24 | 2.75 | 1.46E-07 | 3.62E-07 |
| C10orf126 | 5.15 | 4.50 | 1.49E-07 | 3.70E-07 |
| LINC00221 | 5.62 | 7.63 | 1.50E-07 | 3.70E-07 |
| CTD-2311M21.3 | 2.23 | 4.73 | 1.52E-07 | 3.77E-07 |
| RP11-536K17.1 | 4.42 | 3.02 | 1.54E-07 | 3.81E-07 |
| RP11-176N18.2 | 2.43 | 4.46 | 1.57E-07 | 3.88E-07 |
| LINC01731 | 2.74 | 3.29 | 1.58E-07 | 3.90E-07 |
| RP5-1139I1.1 | 3.33 | 3.64 | 1.60E-07 | 3.95E-07 |
| AC128709.3 | 2.03 | 6.65 | 1.67E-07 | 4.11E-07 |
| RP11-706O15.3 | 2.14 | 7.60 | 1.68E-07 | 4.14E-07 |
| RP11-52L5.6 | 2.95 | 2.60 | 1.69E-07 | 4.16E-07 |
| RP11-482M8.3 | 3.98 | 3.09 | 1.70E-07 | 4.19E-07 |
| AC092635.1 | 3.80 | 2.79 | 1.73E-07 | 4.26E-07 |
| AC019064.1 | 3.50 | 2.56 | 1.79E-07 | 4.39E-07 |
| IGF2-AS | 2.52 | 4.72 | 1.80E-07 | 4.41E-07 |
| AP000439.1 | 3.80 | 4.67 | 1.84E-07 | 4.50E-07 |
| RP11-101E14.3 | 4.10 | 2.82 | 1.85E-07 | 4.52E-07 |
| LINC01077 | 2.92 | 2.70 | 1.85E-07 | 4.52E-07 |
| DIO2-AS1 | 3.07 | 2.75 | 1.93E-07 | 4.71E-07 |
| RP11-385M4.1 | 3.07 | 3.02 | 1.97E-07 | 4.80E-07 |
| LINC01033 | 2.77 | 4.93 | 1.99E-07 | 4.87E-07 |
| AC006262.10 | 3.47 | 3.14 | 2.02E-07 | 4.94E-07 |
| LINC02196 | 3.25 | 3.00 | 2.05E-07 | 4.99E-07 |
| RP11-865I6.2 | 3.53 | 4.70 | 2.06E-07 | 5.01E-07 |
| LINC00972 | 3.49 | 2.72 | 2.12E-07 | 5.15E-07 |
| RP11-293F5.1 | 3.43 | 2.54 | 2.23E-07 | 5.40E-07 |
| U91319.1 | 3.94 | 3.60 | 2.26E-07 | 5.49E-07 |
| RP11-34F13.2 | 2.24 | 3.64 | 2.32E-07 | 5.62E-07 |
| RP11-788H18.1 | 4.06 | 3.19 | 2.33E-07 | 5.64E-07 |
| ANO1-AS2 | 2.58 | 3.39 | 2.45E-07 | 5.91E-07 |
| RP11-973F15.2 | 2.54 | 3.26 | 2.45E-07 | 5.92E-07 |
| CTD-2587H19.2 | 2.14 | 3.21 | 2.46E-07 | 5.92E-07 |
| RP11-1191J2.2 | 2.06 | 4.27 | 2.48E-07 | 5.98E-07 |
| RP11-17M24.3 | 2.15 | 3.12 | 2.57E-07 | 6.19E-07 |
| RP11-89M20.2 | 3.73 | 2.65 | 2.61E-07 | 6.28E-07 |
| RP11-665I14.1 | 5.20 | 3.62 | 2.63E-07 | 6.32E-07 |
| SC22CB-56B3.1 | 5.01 | 3.49 | 2.71E-07 | 6.51E-07 |
| EVX1-AS | 2.76 | 2.43 | 2.88E-07 | 6.91E-07 |
| RP11-11K13.1 | 4.37 | 2.98 | 2.91E-07 | 6.98E-07 |
| C12orf77 | 3.30 | 4.03 | 2.96E-07 | 7.08E-07 |
| LINC01574 | 2.99 | 2.89 | 2.98E-07 | 7.14E-07 |
| AC060834.3 | 5.01 | 3.97 | 3.04E-07 | 7.27E-07 |
| RP11-875H7.5 | 4.28 | 6.23 | 3.15E-07 | 7.52E-07 |
| MAPT-IT1 | 3.42 | 2.68 | 3.21E-07 | 7.66E-07 |
| RP11-479O16.1 | 3.51 | 4.77 | 3.41E-07 | 8.11E-07 |
| RP3-446N13.5 | 3.51 | 2.94 | 3.46E-07 | 8.22E-07 |
| RP11-1145L24.1 | 3.68 | 3.25 | 3.47E-07 | 8.25E-07 |
| RP11-295G12.1 | 3.71 | 2.65 | 3.74E-07 | 8.84E-07 |
| CTD-2168K21.1 | 2.78 | 2.44 | 3.81E-07 | 9.00E-07 |
| RP11-498B4.5 | 2.84 | 2.71 | 3.83E-07 | 9.03E-07 |
| AC006262.4 | 2.41 | 3.69 | 3.86E-07 | 9.12E-07 |
| LINC01895 | 3.06 | 2.47 | 3.86E-07 | 9.12E-07 |
| RP11-114M1.2 | 2.68 | 2.83 | 3.90E-07 | 9.20E-07 |
| LINC01623 | 2.16 | 3.07 | 4.17E-07 | 9.79E-07 |
| CTD-2377D24.4 | 4.17 | 3.23 | 4.19E-07 | 9.83E-07 |
| KCNAB1-AS2 | 2.73 | 2.53 | 4.19E-07 | 9.85E-07 |
| LINC02008 | 4.28 | 3.03 | 4.25E-07 | 9.98E-07 |
| RP11-676J15.1 | 2.37 | 2.52 | 4.37E-07 | 1.03E-06 |
| CTD-2210P24.3 | 2.80 | 2.66 | 4.38E-07 | 1.03E-06 |
| KIRREL3-AS3 | 3.40 | 2.61 | 4.39E-07 | 1.03E-06 |
| AP001476.4 | 3.87 | 2.83 | 4.40E-07 | 1.03E-06 |
| AP001476.2 | 2.33 | 4.19 | 4.79E-07 | 1.12E-06 |
| C11orf44 | 2.60 | 3.19 | 4.80E-07 | 1.12E-06 |
| TCL6 | 2.17 | 6.44 | 4.80E-07 | 1.12E-06 |
| RP11-284G10.1 | 4.19 | 3.18 | 4.81E-07 | 1.12E-06 |
| LINC02042 | 2.20 | 3.80 | 4.90E-07 | 1.14E-06 |
| UPK1A-AS1 | 2.69 | 4.40 | 5.23E-07 | 1.22E-06 |
| LINC00408 | 4.35 | 2.97 | 5.33E-07 | 1.24E-06 |
| RP11-644L4.1 | 2.19 | 2.76 | 5.38E-07 | 1.25E-06 |
| XXyac-YR29IB3.1 | 3.53 | 2.95 | 5.47E-07 | 1.27E-06 |
| RP4-668E10.4 | 3.89 | 2.73 | 5.52E-07 | 1.28E-06 |
| AC007091.1 | 3.17 | 2.90 | 5.65E-07 | 1.31E-06 |
| RP11-475A13.1 | 4.07 | 3.62 | 5.93E-07 | 1.37E-06 |
| LINC01749 | 2.44 | 2.66 | 5.93E-07 | 1.37E-06 |
| RP11-624C23.1 | 2.27 | 4.71 | 6.04E-07 | 1.39E-06 |
| AC007126.1 | 3.23 | 3.32 | 6.13E-07 | 1.41E-06 |
| RP11-476M19.2 | 2.32 | 2.96 | 6.14E-07 | 1.42E-06 |
| RP11-753N8.1 | 2.53 | 2.61 | 6.14E-07 | 1.42E-06 |
| AC006145.4 | 3.72 | 3.32 | 6.47E-07 | 1.49E-06 |
| GPR1-AS | 3.88 | 4.52 | 6.50E-07 | 1.49E-06 |
| RP5-827O9.1 | 3.34 | 2.50 | 6.58E-07 | 1.51E-06 |
| LINC00534 | 2.25 | 3.69 | 6.72E-07 | 1.54E-06 |
| FOXC2-AS1 | 2.43 | 2.96 | 6.88E-07 | 1.58E-06 |
| GATA3-AS1 | 2.24 | 4.09 | 7.02E-07 | 1.60E-06 |
| LINC02065 | 2.44 | 3.06 | 7.14E-07 | 1.63E-06 |
| AP000997.2 | 3.88 | 2.95 | 7.37E-07 | 1.68E-06 |
| AC104389.28 | 2.76 | 5.23 | 7.48E-07 | 1.71E-06 |
| RP11-414H23.3 | 2.11 | 2.61 | 7.53E-07 | 1.72E-06 |
| LINC01630 | 2.59 | 3.40 | 7.62E-07 | 1.74E-06 |
| LINC01681 | 2.60 | 2.69 | 7.68E-07 | 1.75E-06 |
| RP11-161D15.2 | 2.52 | 3.09 | 7.71E-07 | 1.76E-06 |
| LINC00661 | 3.88 | 3.38 | 8.07E-07 | 1.84E-06 |
| PCAT18 | 3.10 | 4.88 | 8.14E-07 | 1.85E-06 |
| KB-1930G5.3 | 3.18 | 2.50 | 8.29E-07 | 1.88E-06 |
| RP11-11N5.3 | 3.26 | 3.04 | 8.31E-07 | 1.89E-06 |
| RP13-895J2.3 | 2.73 | 3.82 | 8.57E-07 | 1.94E-06 |
| LINC01345 | 4.18 | 3.06 | 9.09E-07 | 2.06E-06 |
| GPR50-AS1 | 3.07 | 2.51 | 9.34E-07 | 2.11E-06 |
| RP11-19O2.2 | 3.57 | 2.71 | 9.35E-07 | 2.11E-06 |
| LINC00488 | 4.00 | 3.43 | 9.41E-07 | 2.12E-06 |
| RP11-415C15.2 | 2.49 | 4.03 | 9.57E-07 | 2.16E-06 |
| AC011294.3 | 2.02 | 5.06 | 1.00E-06 | 2.25E-06 |
| RP11-13K12.1 | 2.15 | 6.21 | 1.09E-06 | 2.45E-06 |
| FTCD-AS1 | 2.88 | 2.66 | 1.09E-06 | 2.45E-06 |
| RP11-545A16.1 | 2.34 | 2.62 | 1.13E-06 | 2.53E-06 |
| LINC01228 | 3.41 | 4.71 | 1.13E-06 | 2.53E-06 |
| XXyac-YM21GA2.3 | -2.17 | 3.57 | 1.19E-06 | 2.67E-06 |
| LINC02120 | 2.06 | 2.83 | 1.21E-06 | 2.70E-06 |
| LINC00052 | 3.04 | 2.96 | 1.23E-06 | 2.74E-06 |
| AC108868.6 | 5.37 | 4.32 | 1.24E-06 | 2.77E-06 |
| LINC00237 | 2.91 | 4.01 | 1.25E-06 | 2.78E-06 |
| RP11-66B24.1 | 2.65 | 3.07 | 1.25E-06 | 2.79E-06 |
| RP1-287H17.1 | 2.01 | 2.74 | 1.26E-06 | 2.82E-06 |
| CTD-2314G24.2 | 2.26 | 5.56 | 1.27E-06 | 2.84E-06 |
| OVAAL | 3.03 | 4.46 | 1.29E-06 | 2.88E-06 |
| RP11-209K10.2 | 3.29 | 3.15 | 1.33E-06 | 2.95E-06 |
| RP11-554D14.6 | 2.33 | 4.15 | 1.37E-06 | 3.05E-06 |
| AC012506.2 | 2.55 | 2.54 | 1.38E-06 | 3.07E-06 |
| PSG8-AS1 | 2.69 | 2.67 | 1.43E-06 | 3.17E-06 |
| RP11-124N3.2 | 3.98 | 2.77 | 1.44E-06 | 3.18E-06 |
| RP4-704D23.1 | 2.77 | 2.73 | 1.50E-06 | 3.30E-06 |
| LINC00867 | 2.59 | 3.62 | 1.52E-06 | 3.36E-06 |
| RP11-734I18.1 | 3.33 | 4.99 | 1.55E-06 | 3.42E-06 |
| RP11-445P19.3 | 2.88 | 2.69 | 1.57E-06 | 3.46E-06 |
| RP11-622A1.2 | 3.17 | 2.51 | 1.59E-06 | 3.50E-06 |
| AC064834.1 | 6.07 | 5.91 | 1.78E-06 | 3.91E-06 |
| RP11-445N18.5 | 2.25 | 4.79 | 1.80E-06 | 3.95E-06 |
| LINC00540 | 2.04 | 7.44 | 1.86E-06 | 4.06E-06 |
| LINC00970 | 2.23 | 4.15 | 1.87E-06 | 4.10E-06 |
| AP001042.1 | 2.21 | 2.74 | 1.93E-06 | 4.22E-06 |
| RP11-344E13.4 | 3.28 | 3.60 | 2.04E-06 | 4.45E-06 |
| DPP10-AS1 | 2.66 | 6.52 | 2.06E-06 | 4.48E-06 |
| LINC01467 | 5.05 | 3.67 | 2.06E-06 | 4.49E-06 |
| LINC01998 | 3.33 | 4.28 | 2.11E-06 | 4.59E-06 |
| RP11-206M11.7 | 3.63 | 4.21 | 2.17E-06 | 4.70E-06 |
| RP11-171N4.1 | 4.62 | 3.17 | 2.26E-06 | 4.89E-06 |
| CTA-992D9.6 | 5.21 | 3.95 | 2.32E-06 | 5.02E-06 |
| LINC00939 | 2.10 | 5.59 | 2.47E-06 | 5.34E-06 |
| RP11-702B10.2 | 2.50 | 4.41 | 2.48E-06 | 5.35E-06 |
| LINC01179 | 3.16 | 3.44 | 2.48E-06 | 5.35E-06 |
| RP11-736E3.1 | 2.38 | 2.53 | 2.50E-06 | 5.39E-06 |
| MGC15885 | 2.24 | 2.57 | 2.54E-06 | 5.48E-06 |
| RP11-10H3.1 | 3.47 | 4.67 | 2.54E-06 | 5.48E-06 |
| LINC02046 | 3.37 | 3.29 | 2.54E-06 | 5.48E-06 |
| RP5-856G1.1 | 2.13 | 3.32 | 2.60E-06 | 5.58E-06 |
| AC002511.3 | 2.11 | 4.50 | 2.61E-06 | 5.61E-06 |
| LINC01425 | 4.43 | 3.41 | 2.70E-06 | 5.80E-06 |
| RP11-274M17.3 | 3.51 | 2.56 | 2.83E-06 | 6.07E-06 |
| LINC02200 | 3.43 | 2.83 | 2.84E-06 | 6.09E-06 |
| RP11-775H9.3 | 6.19 | 5.48 | 2.91E-06 | 6.23E-06 |
| RP11-1041F24.1 | 4.18 | 3.05 | 3.06E-06 | 6.53E-06 |
| LINC01037 | 2.82 | 3.15 | 3.28E-06 | 6.97E-06 |
| AC010907.2 | 2.46 | 2.61 | 3.34E-06 | 7.10E-06 |
| LINC00320 | 3.54 | 2.61 | 3.36E-06 | 7.13E-06 |
| RP11-1022B3.1 | 3.40 | 2.60 | 3.37E-06 | 7.15E-06 |
| AC116614.1 | 2.26 | 4.96 | 3.43E-06 | 7.27E-06 |
| AC079466.1 | 4.61 | 5.69 | 3.43E-06 | 7.28E-06 |
| LINC02002 | 3.61 | 2.60 | 3.57E-06 | 7.55E-06 |
| DANT1 | 3.59 | 2.95 | 3.65E-06 | 7.72E-06 |
| RP11-31K23.2 | 3.68 | 2.86 | 3.72E-06 | 7.85E-06 |
| MRGPRG-AS1 | 3.13 | 2.78 | 3.80E-06 | 8.02E-06 |
| LINC00404 | 4.17 | 3.52 | 3.94E-06 | 8.30E-06 |
| LINC01080 | 2.48 | 2.53 | 4.00E-06 | 8.42E-06 |
| LINC01346 | 4.88 | 4.59 | 4.07E-06 | 8.56E-06 |
| LINC01819 | 2.68 | 6.48 | 4.17E-06 | 8.77E-06 |
| RP11-138E9.2 | 3.04 | 2.73 | 4.31E-06 | 9.04E-06 |
| MIR7-3HG | 3.54 | 3.77 | 4.35E-06 | 9.11E-06 |
| RP11-117L5.4 | 2.36 | 2.47 | 4.36E-06 | 9.14E-06 |
| LINC00403 | 4.16 | 4.04 | 4.36E-06 | 9.15E-06 |
| LINC00836 | 4.14 | 3.06 | 4.47E-06 | 9.35E-06 |
| AC006372.5 | 2.73 | 3.09 | 4.48E-06 | 9.38E-06 |
| CTD-2540L5.5 | 2.54 | 2.73 | 4.59E-06 | 9.61E-06 |
| RP11-510C10.3 | 4.09 | 3.14 | 4.73E-06 | 9.89E-06 |
| LINC01153 | 2.99 | 2.52 | 4.78E-06 | 9.99E-06 |
| RP1-205F14P.1 | 2.43 | 2.57 | 4.86E-06 | 1.01E-05 |
| LINC01603 | 2.12 | 2.66 | 4.94E-06 | 1.03E-05 |
| RP11-12K22.1 | 3.22 | 2.53 | 5.01E-06 | 1.04E-05 |
| AP001065.15 | 2.18 | 8.05 | 5.19E-06 | 1.08E-05 |
| LINC02095 | 2.98 | 3.21 | 5.21E-06 | 1.08E-05 |
| RP11-554D14.8 | 2.15 | 3.59 | 5.22E-06 | 1.09E-05 |
| RP11-162D9.3 | 2.32 | 3.07 | 5.31E-06 | 1.10E-05 |
| XXyac-YX65C7_A.3 | 2.25 | 3.58 | 5.39E-06 | 1.12E-05 |
| RP11-481J2.1 | 2.39 | 2.53 | 5.73E-06 | 1.19E-05 |
| TTTY20 | 5.60 | 3.80 | 5.77E-06 | 1.19E-05 |
| RP11-569G13.3 | 3.11 | 5.32 | 5.78E-06 | 1.20E-05 |
| RP11-437J19.1 | 2.17 | 2.56 | 5.79E-06 | 1.20E-05 |
| LINC01804 | 4.23 | 3.40 | 5.84E-06 | 1.21E-05 |
| RP11-474D1.4 | 4.44 | 3.02 | 5.89E-06 | 1.22E-05 |
| LINC01198 | 2.83 | 3.50 | 6.67E-06 | 1.37E-05 |
| RP11-30L8.1 | 3.27 | 2.49 | 6.97E-06 | 1.43E-05 |
| RP11-703M24.5 | 2.22 | 3.36 | 7.00E-06 | 1.44E-05 |
| LINC01701 | 2.08 | 2.77 | 7.03E-06 | 1.44E-05 |
| RP5-855F14.2 | 2.65 | 2.98 | 7.04E-06 | 1.45E-05 |
| RP11-148E17.1 | 3.95 | 3.68 | 7.05E-06 | 1.45E-05 |
| RP11-466I1.1 | 3.67 | 2.70 | 7.12E-06 | 1.46E-05 |
| RP11-69I8.2 | 2.34 | 2.47 | 7.14E-06 | 1.46E-05 |
| CTD-2194D22.1 | 2.90 | 2.85 | 7.18E-06 | 1.47E-05 |
| LINC01665 | 3.82 | 3.30 | 7.20E-06 | 1.48E-05 |
| RP11-362K2.2 | 2.18 | 2.94 | 7.21E-06 | 1.48E-05 |
| RP11-44K6.3 | 2.40 | 3.39 | 8.20E-06 | 1.67E-05 |
| RP11-431J17.1 | 4.71 | 3.28 | 8.85E-06 | 1.80E-05 |
| RP11-20J15.2 | 3.32 | 2.63 | 8.91E-06 | 1.81E-05 |
| CTC-339O9.1 | 2.78 | 4.61 | 8.96E-06 | 1.82E-05 |
| LINC01159 | 4.23 | 3.03 | 9.60E-06 | 1.94E-05 |
| AC010969.1 | 2.33 | 4.11 | 9.63E-06 | 1.95E-05 |
| RP11-407A16.3 | 3.15 | 3.03 | 9.76E-06 | 1.98E-05 |
| AF064860.7 | 2.06 | 3.13 | 9.83E-06 | 1.99E-05 |
| RP11-966I7.2 | 3.70 | 2.75 | 9.86E-06 | 2.00E-05 |
| RP11-554A11.8 | 4.10 | 4.67 | 9.95E-06 | 2.01E-05 |
| LINC00911 | 3.11 | 2.91 | 1.04E-05 | 2.10E-05 |
| AC145123.2 | 3.39 | 2.92 | 1.12E-05 | 2.26E-05 |
| RP11-244B22.11 | 3.79 | 2.69 | 1.22E-05 | 2.44E-05 |
| RP11-184D12.1 | 4.27 | 3.41 | 1.22E-05 | 2.44E-05 |
| LINC01776 | 2.97 | 2.96 | 1.28E-05 | 2.57E-05 |
| RP11-359E19.2 | 2.55 | 6.14 | 1.37E-05 | 2.73E-05 |
| CTD-2515C13.2 | 3.24 | 2.59 | 1.40E-05 | 2.79E-05 |
| RP13-539F13.3 | 2.24 | 2.65 | 1.42E-05 | 2.84E-05 |
| RP3-410C9.2 | 3.18 | 4.46 | 1.51E-05 | 3.00E-05 |
| LINC01989 | 2.60 | 3.89 | 1.54E-05 | 3.05E-05 |
| SIX3-AS1 | 2.35 | 3.69 | 1.65E-05 | 3.26E-05 |
| LINC00613 | 4.41 | 2.99 | 1.66E-05 | 3.27E-05 |
| RP11-857B24.1 | 2.16 | 3.51 | 1.66E-05 | 3.28E-05 |
| RP3-326I13.1 | 3.40 | 3.04 | 1.78E-05 | 3.51E-05 |
| AC004009.3 | 3.17 | 2.44 | 1.78E-05 | 3.51E-05 |
| RP11-429A20.3 | 2.66 | 4.51 | 1.79E-05 | 3.52E-05 |
| CTD-2140G10.2 | 3.33 | 3.41 | 1.85E-05 | 3.64E-05 |
| LINC01203 | 2.35 | 2.59 | 1.92E-05 | 3.76E-05 |
| RP11-3G21.1 | 3.87 | 2.75 | 1.93E-05 | 3.78E-05 |
| RP11-384J4.2 | 2.54 | 3.24 | 1.95E-05 | 3.82E-05 |
| RP11-1C1.6 | 4.18 | 3.30 | 1.98E-05 | 3.87E-05 |
| RP11-124N3.3 | 3.83 | 2.90 | 1.99E-05 | 3.88E-05 |
| LINC01182 | 2.12 | 2.70 | 2.00E-05 | 3.91E-05 |
| RP11-794A8.1 | 5.10 | 4.63 | 2.00E-05 | 3.92E-05 |
| LINC01886 | 2.97 | 3.30 | 2.10E-05 | 4.10E-05 |
| AC116609.2 | 2.46 | 3.68 | 2.14E-05 | 4.17E-05 |
| CTB-78F1.2 | 2.62 | 3.60 | 2.18E-05 | 4.24E-05 |
| RP11-80F22.14 | 3.06 | 2.41 | 2.23E-05 | 4.33E-05 |
| LINC01029 | 4.49 | 4.00 | 2.29E-05 | 4.45E-05 |
| MIR124-2HG | 3.17 | 2.51 | 2.35E-05 | 4.55E-05 |
| RP11-318G21.4 | 3.84 | 3.80 | 2.50E-05 | 4.83E-05 |
| LINC02203 | 3.52 | 2.56 | 2.53E-05 | 4.88E-05 |
| LINC01551 | 3.87 | 3.53 | 2.57E-05 | 4.96E-05 |
| AC147651.1 | 2.04 | 4.14 | 2.79E-05 | 5.37E-05 |
| RP11-1081M5.2 | 2.15 | 3.39 | 2.81E-05 | 5.40E-05 |
| RP11-2A4.4 | 4.00 | 3.36 | 2.84E-05 | 5.47E-05 |
| AC112518.3 | 2.54 | 2.57 | 2.90E-05 | 5.56E-05 |
| RP11-204E9.1 | 3.71 | 3.77 | 3.12E-05 | 5.98E-05 |
| LINC02122 | 2.36 | 3.59 | 3.12E-05 | 5.98E-05 |
| LINC01924 | 3.26 | 2.61 | 3.25E-05 | 6.21E-05 |
| C8orf49 | 2.25 | 3.49 | 3.38E-05 | 6.44E-05 |
| PEX5L-AS1 | 4.84 | 4.88 | 3.44E-05 | 6.55E-05 |
| RP11-145E17.2 | 2.86 | 2.56 | 3.51E-05 | 6.68E-05 |
| AC005307.4 | 2.90 | 5.07 | 3.55E-05 | 6.74E-05 |
| AC018866.1 | 3.51 | 2.82 | 3.57E-05 | 6.78E-05 |
| THRA1/BTR | 2.95 | 3.84 | 3.66E-05 | 6.95E-05 |
| LINC00458 | 3.00 | 2.62 | 3.69E-05 | 7.01E-05 |
| RP11-614F17.2 | 3.13 | 3.16 | 3.78E-05 | 7.16E-05 |
| AP000997.1 | 3.35 | 2.72 | 3.91E-05 | 7.40E-05 |
| CLDN10-AS1 | 2.90 | 3.99 | 4.08E-05 | 7.68E-05 |
| RP11-275H4.1 | 2.31 | 2.52 | 4.09E-05 | 7.71E-05 |
| RP11-707A18.1 | 2.49 | 4.33 | 4.21E-05 | 7.92E-05 |
| RP11-34C15.2 | 4.63 | 3.46 | 4.21E-05 | 7.92E-05 |
| AC006019.3 | 4.13 | 3.35 | 4.28E-05 | 8.05E-05 |
| RP11-760D2.5 | 2.89 | 3.81 | 4.55E-05 | 8.53E-05 |
| TUSC7 | 3.55 | 4.47 | 4.68E-05 | 8.78E-05 |
| LINC01320 | 2.88 | 4.82 | 4.69E-05 | 8.79E-05 |
| RP11-203E8.1 | 2.02 | 3.30 | 5.28E-05 | 9.85E-05 |
| RP11-366H4.1 | 3.69 | 3.84 | 5.44E-05 | 0.000101206 |
| CTD-3064C13.1 | 2.70 | 3.01 | 5.63E-05 | 0.000104657 |
| RP11-91P17.1 | 3.60 | 2.64 | 5.69E-05 | 0.000105728 |
| RP11-307P5.1 | 2.23 | 4.08 | 5.80E-05 | 0.000107613 |
| LINC01440 | 2.99 | 2.57 | 6.07E-05 | 0.000112618 |
| EPHA5-AS1 | 2.48 | 3.63 | 6.42E-05 | 0.000118738 |
| LINC01541 | 3.06 | 2.55 | 6.55E-05 | 0.000121084 |
| RP11-567C20.2 | 3.79 | 2.73 | 6.64E-05 | 0.000122735 |
| EMX2OS | 2.11 | 5.83 | 7.04E-05 | 0.000129671 |
| AC005808.3 | 2.07 | 2.73 | 7.29E-05 | 0.000134196 |
| RP11-510C10.4 | 3.36 | 2.66 | 7.60E-05 | 0.000139539 |
| RP1-310O13.13 | 2.39 | 2.90 | 7.75E-05 | 0.000142157 |
| LINC01324 | 3.16 | 3.74 | 7.95E-05 | 0.00014553 |
| RP11-279O17.2 | 2.61 | 2.54 | 7.97E-05 | 0.000145971 |
| RP11-2L8.2 | 3.99 | 3.35 | 8.29E-05 | 0.000151492 |
| RP11-438D14.2 | 2.31 | 2.73 | 8.89E-05 | 0.000161867 |
| LINC01445 | 4.33 | 3.02 | 9.12E-05 | 0.000165809 |
| RP11-116O18.1 | 2.16 | 6.63 | 9.57E-05 | 0.000173674 |
| RP11-429E11.2 | 2.07 | 2.85 | 9.91E-05 | 0.000179527 |
| LINC00445 | 2.74 | 2.72 | 9.93E-05 | 0.00017984 |
| VENTXP1 | 3.05 | 2.41 | 0.000100371 | 0.00018173 |
| TUSC8 | 2.63 | 4.33 | 0.00010347 | 0.000187077 |
| RP11-526F3.1 | 2.51 | 5.07 | 0.000104048 | 0.000187971 |
| RP11-500B12.1 | 2.26 | 4.02 | 0.000105763 | 0.000190876 |
| AC005150.1 | 2.92 | 2.82 | 0.000107436 | 0.000193778 |
| RP11-531A24.3 | 2.13 | 5.50 | 0.00011183 | 0.000201419 |
| RP1-200K18.1 | 2.58 | 2.61 | 0.000112474 | 0.000202539 |
| RP11-693J15.6 | 2.93 | 5.01 | 0.000120919 | 0.000216918 |
| LINC02063 | 2.98 | 2.47 | 0.000122336 | 0.000219283 |
| LINC02055 | 2.42 | 5.44 | 0.000124725 | 0.000223298 |
| RP11-279F6.2 | 3.45 | 3.97 | 0.000125698 | 0.00022495 |
| RP11-91J3.3 | 2.87 | 2.79 | 0.000128567 | 0.000229856 |
| RP11-298E9.5 | 2.01 | 2.56 | 0.000131875 | 0.000235487 |
| LINC01158 | 3.01 | 3.14 | 0.00013346 | 0.000238081 |
| AC007731.1 | 2.50 | 2.59 | 0.000136784 | 0.000243718 |
| LINC00676 | 4.75 | 3.72 | 0.000146158 | 0.000259956 |
| AL773572.7 | 2.16 | 3.81 | 0.00015896 | 0.000281607 |
| RP11-563N12.2 | 2.53 | 2.77 | 0.000161025 | 0.000284984 |
| CTB-180C19.1 | 4.49 | 3.33 | 0.000165145 | 0.000292102 |
| RP11-240M16.1 | 2.70 | 3.94 | 0.000177574 | 0.000313036 |
| CTB-78F1.1 | 2.57 | 2.80 | 0.000177806 | 0.000313383 |
| FAM230B | 2.24 | 2.65 | 0.000181232 | 0.000319233 |
| LINC01021 | 2.15 | 5.03 | 0.000184055 | 0.000323887 |
| C17orf77 | 2.47 | 3.06 | 0.00018457 | 0.000324666 |
| LINC01210 | 3.23 | 2.98 | 0.000189564 | 0.000332992 |
| AC096570.2 | 3.14 | 2.79 | 0.000194611 | 0.000341455 |
| RP11-60A8.1 | 2.04 | 3.13 | 0.000209889 | 0.000366538 |
| CTC-490G23.4 | 2.01 | 2.45 | 0.000221504 | 0.000385694 |
| CTC-286N12.1 | 3.94 | 3.39 | 0.000235321 | 0.000408732 |
| AC006050.2 | 3.38 | 4.09 | 0.000236776 | 0.000411167 |
| HTR5A-AS1 | 3.60 | 2.79 | 0.000236822 | 0.000411167 |
| RP11-402N8.1 | 2.86 | 2.46 | 0.000237978 | 0.000413015 |
| LINC02212 | 3.16 | 4.67 | 0.000242259 | 0.000419875 |
| LINC01054 | 2.66 | 3.45 | 0.000254016 | 0.000439656 |
| LINC01606 | 2.32 | 5.29 | 0.000263783 | 0.000455681 |
| RP13-895J2.6 | 2.57 | 3.32 | 0.000273276 | 0.000471443 |
| LINC01312 | 2.03 | 2.77 | 0.000279209 | 0.000481123 |
| LINC01497 | 2.65 | 3.89 | 0.000282015 | 0.000485677 |
| CTD-2552K11.2 | 2.19 | 2.58 | 0.000305855 | 0.000524616 |
| RP1-32I10.10 | 2.03 | 2.54 | 0.000308022 | 0.00052803 |
| LINC00581 | 2.88 | 3.22 | 0.000311529 | 0.000533428 |
| RP13-895J2.2 | 2.10 | 3.18 | 0.000335935 | 0.000573247 |
| AC005307.1 | 2.50 | 3.30 | 0.000343239 | 0.000584485 |
| RP11-643A5.2 | 3.96 | 3.14 | 0.000358904 | 0.000610233 |
| LINC02125 | 2.48 | 2.87 | 0.000377126 | 0.000640606 |
| CTD-2130O13.1 | 2.86 | 4.22 | 0.00038959 | 0.0006609 |
| LINC01514 | 2.67 | 2.60 | 0.000392251 | 0.000664911 |
| LINC01822 | 2.26 | 2.59 | 0.000395305 | 0.000669709 |
| RP5-1119A7.17 | 2.56 | 2.55 | 0.000405068 | 0.0006856 |
| RP11-369C8.1 | 2.68 | 2.77 | 0.000406878 | 0.000688403 |
| RP11-463C8.7 | 2.16 | 2.45 | 0.000433219 | 0.000731864 |
| RP11-21B23.3 | 3.63 | 2.74 | 0.000454124 | 0.000765303 |
| LINC01257 | 2.36 | 3.32 | 0.000467107 | 0.000786001 |
| LINC00379 | 3.62 | 3.46 | 0.000484224 | 0.000813733 |
| RP11-323H21.3 | 2.83 | 2.78 | 0.000497204 | 0.000834763 |
| RP11-132E11.2 | 2.38 | 2.57 | 0.000525959 | 0.000881223 |
| RP11-415C15.1 | 3.09 | 2.64 | 0.000544504 | 0.000910931 |
| RP11-433M22.2 | 2.10 | 3.32 | 0.000565543 | 0.000944717 |
| RP11-297L17.2 | 3.46 | 2.66 | 0.000582627 | 0.000971805 |
| RP11-720L8.1 | 2.74 | 2.64 | 0.000585143 | 0.00097582 |
| RP11-61O11.1 | 3.02 | 2.95 | 0.000602972 | 0.001004244 |
| ADIPOQ-AS1 | 3.09 | 2.86 | 0.000651939 | 0.001081373 |
| LINC00200 | 3.16 | 4.40 | 0.000673698 | 0.001116639 |
| LINC01632 | 2.62 | 2.54 | 0.000703939 | 0.001163962 |
| LINC02050 | 2.52 | 2.83 | 0.0007162 | 0.001183361 |
| LINC00523 | 2.57 | 2.50 | 0.000732199 | 0.001208905 |
| LINC00456 | 2.36 | 2.47 | 0.0007574 | 0.001247294 |
| CTD-2234N14.2 | 2.53 | 2.60 | 0.000844051 | 0.001380599 |
| RP11-94B19.7 | 2.19 | 3.35 | 0.000856165 | 0.001399496 |
| RP11-449J10.1 | 2.21 | 2.40 | 0.000884352 | 0.00144283 |
| LINC01413 | 2.86 | 3.49 | 0.000897161 | 0.001462663 |
| AC023347.1 | 2.77 | 3.03 | 0.000926923 | 0.001508236 |
| RP11-459O1.2 | 2.39 | 3.69 | 0.001016511 | 0.001646751 |
| RP11-706O15.7 | 2.21 | 3.99 | 0.001081414 | 0.001746889 |
| CTC-525D6.1 | 2.55 | 2.55 | 0.001126644 | 0.001815317 |
| LINC02066 | 2.45 | 3.59 | 0.001255987 | 0.002017191 |
| DSCAM-AS1 | 2.72 | 3.94 | 0.001323168 | 0.002119758 |
| CH507-513H4.3 | 2.94 | 4.26 | 0.001369626 | 0.002189495 |
| RP11-489D6.2 | 2.13 | 3.74 | 0.001380554 | 0.002204211 |
| RP11-305B6.3 | 2.33 | 2.57 | 0.001408914 | 0.002246287 |
| RP11-510C10.2 | 2.69 | 2.49 | 0.001451651 | 0.002311134 |
| LINC00678 | 2.07 | 2.70 | 0.001536738 | 0.002440525 |
| LINC00967 | 3.04 | 3.25 | 0.001631379 | 0.002583954 |
| RP11-543H12.1 | 2.40 | 2.80 | 0.001786322 | 0.002815924 |
| RP11-481E4.2 | 2.88 | 2.55 | 0.001791707 | 0.002823916 |
| LINC01015 | 2.97 | 2.60 | 0.001830811 | 0.002883012 |
| LINC00837 | 2.47 | 2.60 | 0.001993325 | 0.00312082 |
| RP11-138I17.1 | 2.40 | 3.56 | 0.002077297 | 0.003247181 |
| LINC00703 | 2.63 | 3.84 | 0.002183633 | 0.003403306 |
| RP3-438O4.4 | 2.28 | 2.80 | 0.002246455 | 0.003498783 |
| AP000439.3 | 2.19 | 3.80 | 0.0023099 | 0.003592598 |
| AC007682.1 | 2.13 | 2.44 | 0.002321841 | 0.003608037 |
| RP1-293L8.2 | 2.03 | 2.63 | 0.002340346 | 0.003634902 |
| RP11-497G19.1 | 2.28 | 5.11 | 0.002520576 | 0.003901409 |
| RP11-232D9.3 | 2.71 | 2.74 | 0.002788307 | 0.004294165 |
| RP11-1E6.1 | 2.46 | 2.45 | 0.002984286 | 0.004580235 |
| LINC01793 | 2.57 | 3.59 | 0.003323363 | 0.005071101 |
| LINC01935 | 2.06 | 2.40 | 0.003537766 | 0.005379927 |
| PEX5L-AS2 | 2.13 | 2.43 | 0.004334189 | 0.006532278 |
| RP11-598D14.1 | 2.24 | 2.60 | 0.004429821 | 0.006660719 |
| RP11-280H21.1 | 2.51 | 2.89 | 0.004782867 | 0.007151897 |
| RP11-1026M7.3 | 2.44 | 2.41 | 0.005013238 | 0.007480189 |
| RP11-53B5.1 | 2.04 | 3.71 | 0.005093174 | 0.0075944 |
| AF241725.6 | 2.58 | 2.58 | 0.008055491 | 0.011738057 |
| CH507-513H4.4 | 2.07 | 3.32 | 0.018818289 | 0.026136675 |


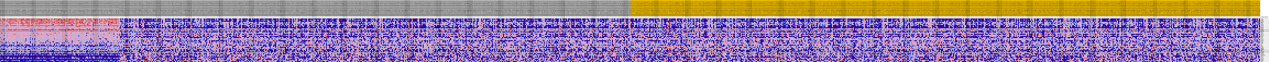


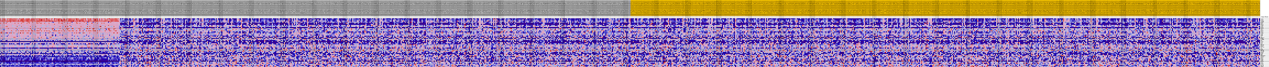


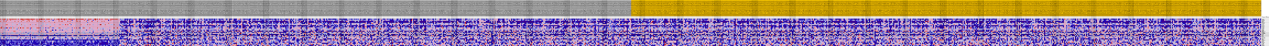


Figure S1-3. Gene set enrichment analyses of LINC01614 in NSCLC (enrichment heatmap).
